# Supplementary material for: Design and Synthesis of New Pyrimidine-Quinolone Hybrids as Novel hLDHA Inhibitors
Source: Pharmaceuticals (Basel). 2022 Jun 24;15(7):792. doi: 10.3390/ph15070792 (PMC9322123; doi:10.3390/ph15070792)
Supplement: Supplementary file 1 [file pharmaceuticals-15-00792-s001.zip › pharmaceuticals-1762849-supplementary.pdf]

Supplementary Materials

Design and Synthesis of New Pyrimidine-Quinolone Hybrids as Novel *h*LDHA Inhibitors

Iván Díaz, Sofia Salido, Manuel Nogueras and Justo Cobo *

Facultad de Ciencias Experimentales, Departamento de Química Inorgánica y Orgánica, Universidad de Jaén, E-23071 Jaén, Spain; idc00005@red.ujaen.es (I.D.); ssalido@ujaen.es (S.S.); mmontiel@ujaen.es (M.N.)
* Correspondence: jcobo@ujaen.es

Table of contents.

1. *In-silico* studies 4

2. Chemistry 7

3. LDHA biological assays. 8

3.1 Dose-response inhibition curves 8

4. NMR, MS and HRMS spectra. 14

4.1 Compound 8 14

4.2 Compound 9 17

4.3 Compound 15 20

4.4 Compound 19 23

4.5 Compound 22a 26

4.6 Compound 22b 29

4.7 Compound 22c 32

4.8 Compound 23a 35

4.9 Compound 23b 38

4.10 Compound 23c 41

4.11 Compound 24a 44

4.12 Compound 24b 47

4.13 Compound 24c 50

4.14 Compound 25a 53

4.15 Compound 25b 56

4.16 Compound 25c 59

4.17 Compound 26a 62

4.18 Compound 26b 65

4.19 Compound 26c 68

4.20 Compound 27a 71

4.21 Compound 27b 74

4.22 Compound 27c 77

4.23 Compound 28a 80

4.24 Compound 28b 83

4.25 Compound 28c 86

4.26 Compound 29a 89

4.27 Compound 29b 92

4.28 Compound 29c 95

4.29 Compound 30a 98
4.30 Compound 30b 101
4.31 Compound 30c 104
4.32 Compound 31a 107
4.33 Compound 31b 110
4.34 Compound 31c 113
4.35 Compound 32a 116
4.36 Compound 32b 119
4.37 Compound 32c 122
4.38 Compound 35a 125
4.39 Compound 35b 128
4.40 Compound 35c 131

1. *In-silico* studies

Table S1. Affinity and energy values (kcal/mol) involved in the interaction of the screening compounds (**10**–**21**) and reference **W31** when docked in the LDHA active site.

Compound	S	Arg^{168}	His^{192}	Asn^{137}	Asp^{194}
**W31**	-10,444	-8,7	-6	-3,6	-3,5
**10**	-9,950	-10,3	0	0	-7,7
**11**	-9,301	0	-2,7	-4,5	0
**12**	-7.467	0	0	0	-10.1
**13**	-9.601	-7.1	0	0	-4.7
**14**	-9.983	-5.7	-3.2	0	-3.5
**15**	-9,231	-1,6	0	0	-5
**16**	-9,372	0	-2,6	-4,4	0
**17**	-9,463	-6,5	0	0	0
**18**	-9,917	-6	0	0	-3,4
**19**	-8,130	0	0	0	0
**20**	-8,70	0	0	0	-2,5
**21**	-8,399	0	-2,5	-4,5	0

Table S2. Affinity and energy values (kcal/mol) involved in the interaction of the screening compounds (**10**–**21**) and reference **NAD** when docked in the NAD site.

Compound	S	Asp^{51}	Val^{135}	Arg^{98}	Val^{30}
**NAD**	-13,280	-7,7	-3,7	-16,9	-5,6
**10**	-9,847	0	-3,9	-4,3	0
**11**	-8,446	0	-3,3	-5,9	0
**12**	-8.668	0	0	0	0
**13**	-9.782	0	0	0	0
**14**	-8.431	-3.4	0	0	0
**15**	-8,001	0	0	-2,4	0
**16**	-8,499	-0,7	0	0	0
**17**	-8,597	-0,6	0	0	0
**18**	-8,588	0	0	0	0
**19**	-8,486	0	0	0	0
**20**	-8,829	0	0	0	0
**21**	-9,073	0	0	0	0

Table S3. Affinity and energy values (kcal/mol) involved in the interaction of the screening compounds (**10**–**21**) when docked in the extended site.

Compound	S	Arg^{168}	His^{192}	Asn^{137}	Asp^{194}	Asp^{51}	Val^{135}	Arg^{98}	Val^{30}
**10**	-8,652	-3,1	0	-1,3	0	0	0	0	0
**11**	-7,608	-9,3	-7	-3,4	-3,4	0	0	0	0
**12**	-8.727	-0.7	0	0	0	0	0	0	0
**13**	-8.239	0	0	0	0	0	0	0	0
**14**	-8.121	0	-5.8	0	-4.8	0	0	0	0
**15**	-8,098	0	0	-0,7	0	0	0	0	0
**16**	-7,857	0	-3,1	-4,9	0	0	0	0	0
**17**	-8,172	0	0	0	-1,6	0	0	0	0
**18**	-8,658	0	0	0	0	0	0	0	0
**19**	-8,341	-2,9	0	-3,5	0	0	0	0	0
**20**	-7,826	0	-4,4	-0,5	0	0	0	0	0
**21**	-7,385	-3,7	0	-8,1	0	0	0	0	0

Table S4. Affinity and energy values (kcal/mol) involved in the interaction of compounds **24**–**31**(**a**–**c**) with the main aminoacid residues. If empty, that compound did not pass some of the filtering stages.

Quinolone	Aryl	Comp.	Arg^{168}	His^{192}	Asn^{137}	Asp^{194}	Affinity
6-H	4-ClPh	**24a**	-7,8	0	0	-4,8	-9,240
	4-CF_3Ph	**25a**	-4,5	0	-0,5	-2	-9,781
	Naphthalene-2-yl	**26a**	-5,1	0	-0,7	-3,1	-10,254
	3,4-(OCH_2O)Ph	**27a**					
6-Cl	4-ClPh	**24b**	-4,7	0	-0,5	-5,1	-9,971
	4-CF_3Ph	**25b**	-4,3	0	-0,6	-4	-10,115
	Naphthalene-2-yl	**26b**	-5,9	0	0	-3,1	-10,355
	3,4-(OCH_2O)Ph	**27b**	-5,8	-2	-4,2	0	-10,066
6-OCH_3	4-ClPh	**24c**	-8,2	-0,6	-0,6	0	-9,215
	4-CF_3Ph	**25c**	-2,2	0	0	0	-9,442
	Naphthalene-2-yl	**26c**	-6,6	0	0	-3,2	-10,765
	3,4-(OCH_2O)Ph	**27c**	-3,7	0	0	0	-9,353
6-H	4-ClPh	**28a**					
	4-CF_3Ph	**29a**	-11,1	0	0	0	-9,217
	Naphthalene-2-yl	**30a**					
	3,4-(OCH_2O)Ph	**31a**					
6-Cl	4-ClPh	**28b**	-8,7	0	0	0	-9,145
	4-CF_3Ph	**29b**	-4,4	0	0	0	-9,506
	Naphthalene-2-yl	**30b**					
	3,4-(OCH_2O)Ph	**31b**	-2,5	-0,8	0	0	-9,024
6-OCH_3	4-ClPh	**28c**					
	4-CF_3Ph	**29c**					
	Naphthalene-2-yl	**30c**	-5,9	0	0	0	-9,357
	3,4-(OCH_2O)Ph	**31c**	-4,8	0	0	0	-9,268

Table S5. Affinity and energy values (kcal/mol) involved in the interaction of the different pyrimidine-quinolone hybrids **24**–**31**(**a**–**c**) and **33**–**36**(**a**–**c**) with the main aminoacid residues. If empty, that compound did not pass some of the filtering stages.

Quinolone	Aryl	Comp.	Arg^{168}	His^{192}	Asn^{137}	Asp^{194}	Affinity
6-H	4-ClPh	**24a**	-7,8	0	0	-4,8	-9,240
	4-CF_3Ph	**25a**	-4,5	0	-0,5	-2	-9,781
	Naphthalene-2-yl	**26a**	-5,1	0	-0,7	-3,1	-10,254
	3,4-(OCH_2O)Ph	**27a**					
6-Cl	4-ClPh	**24b**	-4,7	0	-0,5	-5,1	-9,971
	4-CF_3Ph	**25b**	-4,3	0	-0,6	-4	-10,115
	Naphthalene-2-yl	**26b**	-5,9	0	0	-3,1	-10,355
	3,4-(OCH_2O)Ph	**27b**	-5,8	-2	-4,2	0	-10,066
6-OCH_3	4-ClPh	**24c**	-8,2	-0,6	-0,6	0	-9,215
	4-CF_3Ph	**25c**	-2,2	0	0	0	-9,442
	Naphthalene-2-yl	**26c**	-6,6	0	0	-3,2	-10,765
	3,4-(OCH_2O)Ph	**27c**	-3,7	0	0	0	-9,353
6-H	4-ClPh	**28a**					
	4-CF_3Ph	**29a**	-11,1	0	0	0	-9,217
	Naphthalene-2-yl	**30a**					
	3,4-(OCH_2O)Ph	**31a**					
6-Cl	4-ClPh	**28b**	-8,7	0	0	0	-9,145
	4-CF_3Ph	**29b**	-4,4	0	0	0	-9,506
	Naphthalene-2-yl	**30b**					
	3,4-(OCH_2O)Ph	**31b**	-2,5	-0,8	0	0	-9,024
6-OCH_3	4-ClPh	**28c**					
	4-CF_3Ph	**29c**					
	Naphthalene-2-yl	**30c**	-5,9	0	0	0	-9,357
	3,4-(OCH_2O)Ph	**31c**	-4,8	0	0	0	-9,268
6-H	4-ClPh	**33a**	-6,5	0	0	0	-9,278
	4-CF_3Ph	**34a**	-6,1	0	0	-0,9	-10,134
	Naphthalene-2-yl	**35a**	-6	0	0	-0,9	-10,324
	3,4-(OCH_2O)Ph	**36a**					
6-Cl	4-ClPh	**33b**	-2,8	0	0	-0,7	-9,616
	4-CF_3Ph	**34b**	-5,6	0	0	-6	-10,218
	Naphthalene-2-yl	**35b**	-5,4	-1,5	0	-0,5	-9,693
	3,4-(OCH_2O)Ph	**36b**	-5,2	0	0	-5,3	-9,820
6-OCH_3	4-ClPh	**33c**	-6,6	0	0	0	-9,386
	4-CF_3Ph	**34c**	-11,2	0	-1,5	0	-9,251
	Naphthalene-2-yl	**35c**	-6,7	0	0	0	-9,329
	3,4-(OCH_2O)Ph	**36c**					

Table S6. Mean energy values (kcal/mol) involved in the interaction of hybrids **24**–**31**(**a**–**c**) and **33**–**36**(**a**–**c**) with the main aminoacid residues

Substitution	Compounds	Arg^{168}	Asp^{194}	His^{192}	Asn^{137}	Affinity
Ortho	**24**-**27**(**a**-**c**)	-5,3	-3,6	-1,3	-1,2	-9,869
Para	**28**-**31**(**a**-**c**)	-6,2	0,0	-0,8	0,0	-9,253
Meta	**33**-**36**(**a**-**c**)	-6,2	-2,4	-1,5	-1,5	-9,705

2. **Chemistry**

Table S7. Conditions used in different trials for reaction between intermediates **24**–**25** and **1**–**4**.

Solvent	Acid	Base	Catalyst	Heating
EtOH				MW (120°C)
EtOH		Et_3N		MW (120°C)
EtOH		K_2CO_3		MW (120°C)
EtOH		K_2CO_3	KI	MW (120°C)
EtOH	pTSA			MW (120°C)
EtOH	HCl			MW (120°C)
DMF		K_2CO_3	KI	MW (160°C)
DMF		K_2CO_3	KI	MW (180°C)
DMF		K_2CO_3	$Pd(OAc)_2$ + BINAP	80°C
DMSO		K_2CO_3	$AgNO_3$	80°C →100°C
CH_3CN				MW (120°C)
CH_3CN		Et3N		MW (120°C)
CH_3CN		K_2CO_3	$Pd(OAc)_2$	MW (120°C)

3. LDHA biological assays.

3.1 Dose-response inhibition curves

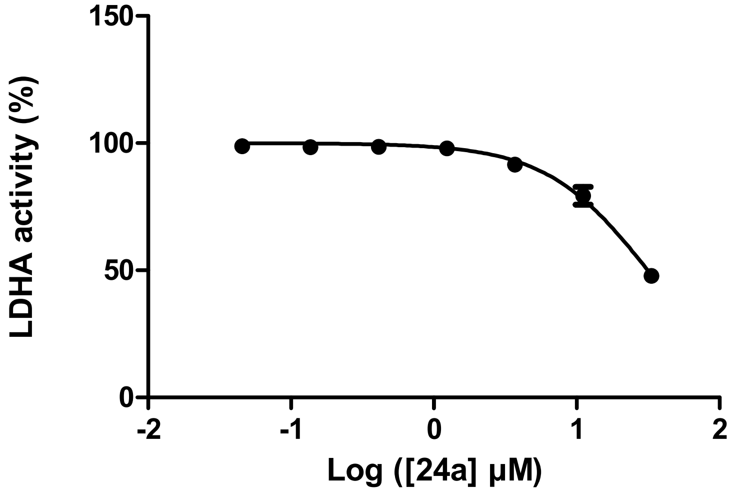

Figure S1. LDHA inhibition curve of compound **24a** (mean ± SD of *n* = 3 replicates)

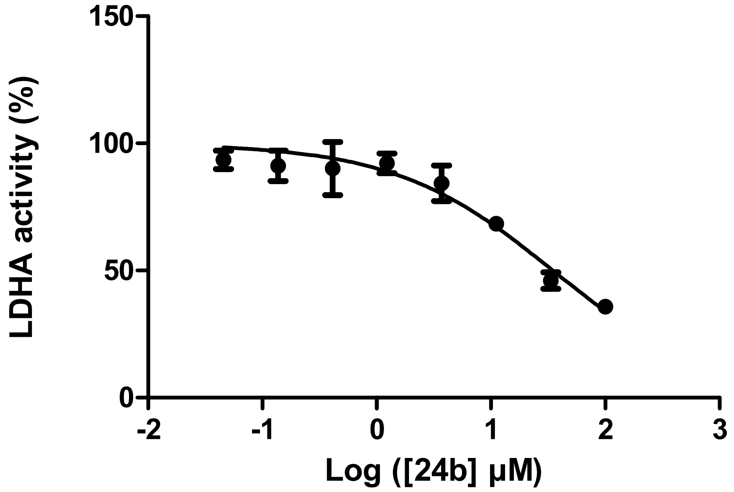

Figure S2. LDHA inhibition curve of compound **24b** (mean ± SD of *n* = 3 replicates)

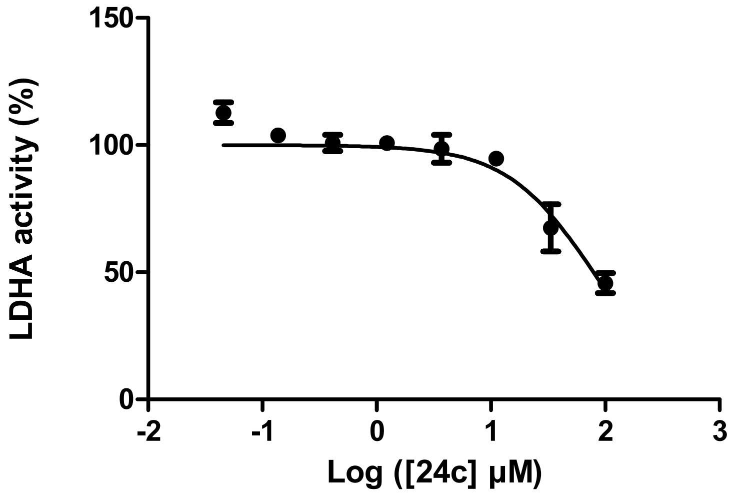

Figure S3. LDHA inhibition curve of compound **24c** (mean ± SD of *n* = 3 replicates)

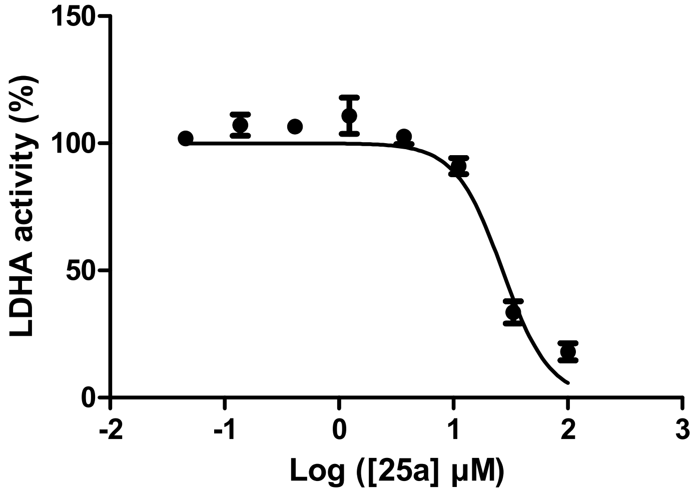

Figure S4. LDHA inhibition curve of compound **25a** (mean ± SD of *n* = 3 replicates)

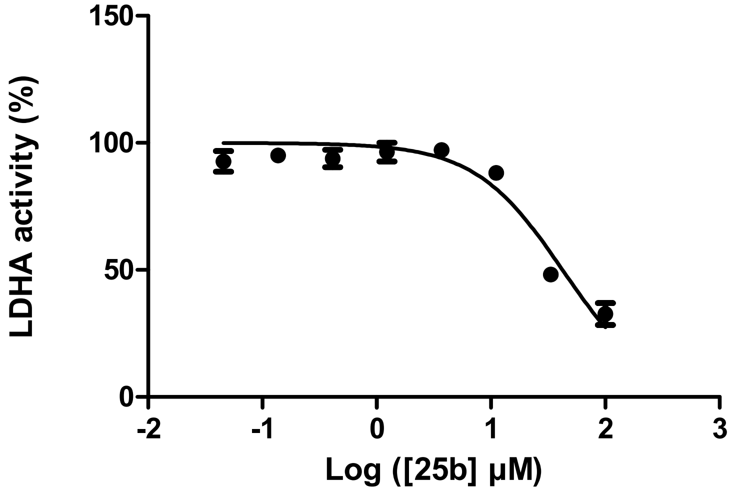

Figure S5. LDHA inhibition curve of compound **25b** (mean ± SD of *n* = 3 replicates)

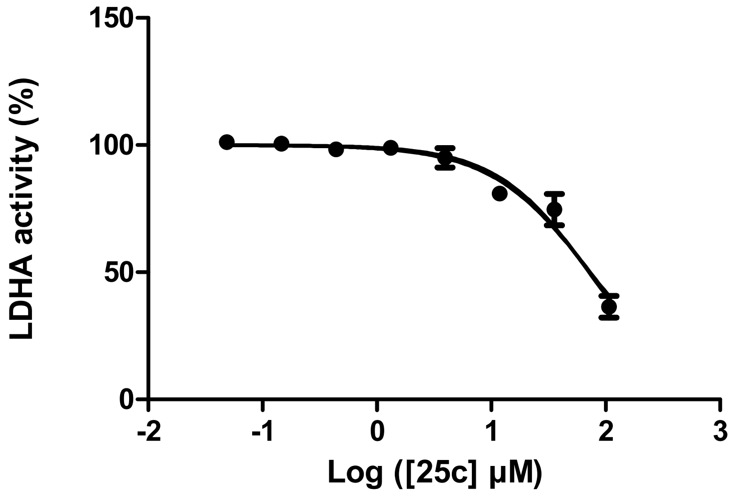

Figure S6. LDHA inhibition curve of compound **25c** (mean ± SD of *n* = 3 replicates)

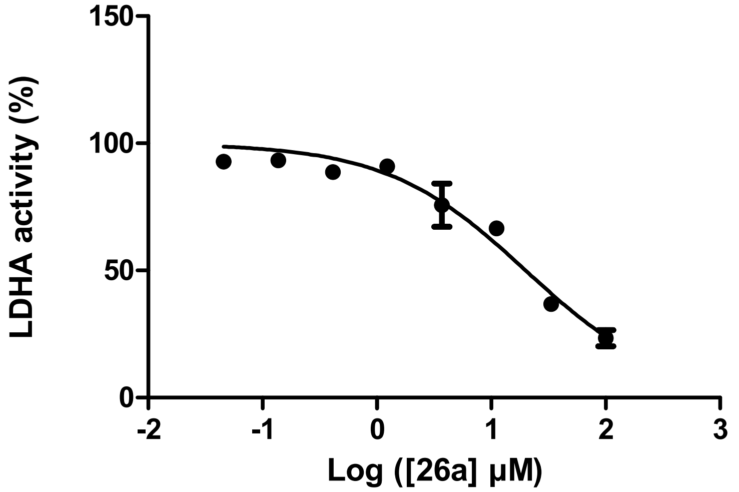

Figure S7. LDHA inhibition curve of compound **26a** (mean ± SD of *n* = 3 replicates)

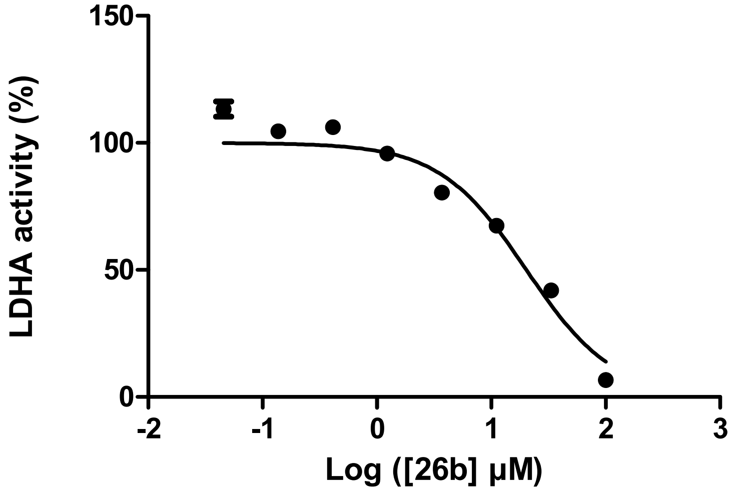

Figure S8. LDHA inhibition curve of compound **26b** (mean ± SD of *n* = 3 replicates)

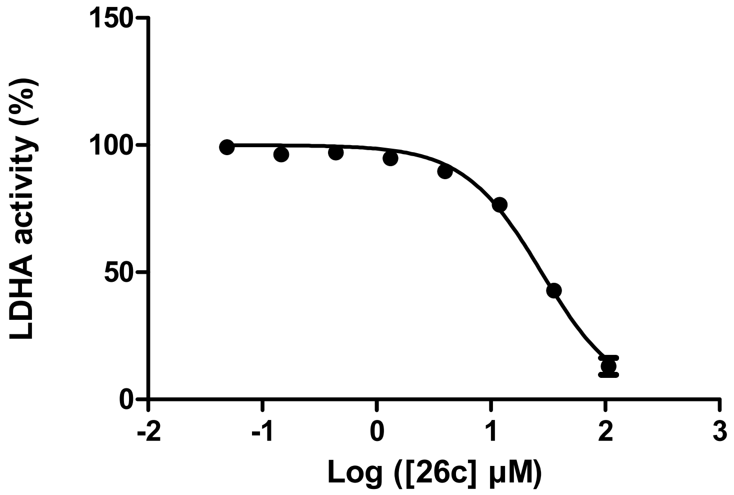

Figure S9. LDHA inhibition curve of compound **26c** (mean ± SD of *n* = 3 replicates)

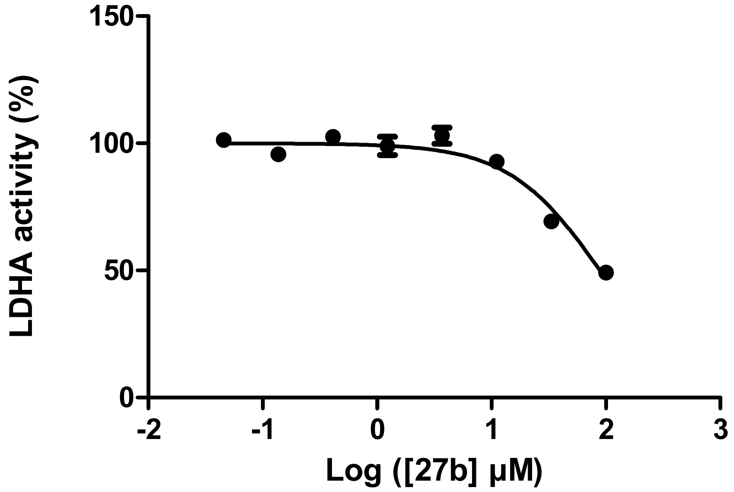

Figure S10. LDHA inhibition curve of compound **27b** (mean ± SD of *n* = 3 replicates)

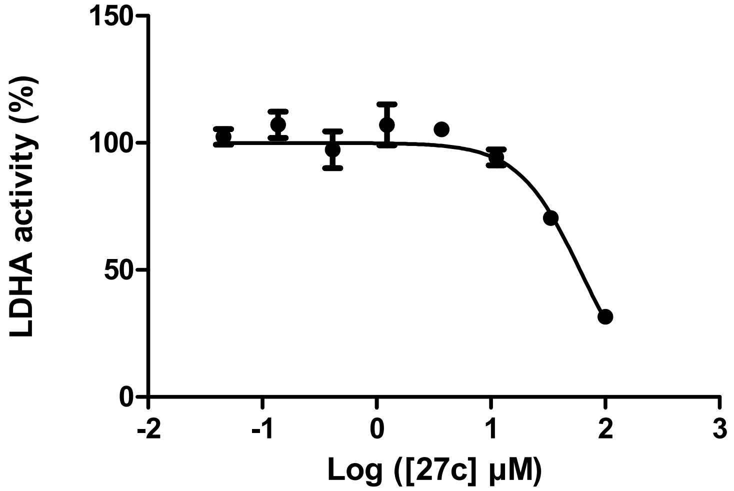

Figure S11. LDHA inhibition curve of compound **27c** (mean ± SD of *n* = 3 replicates)

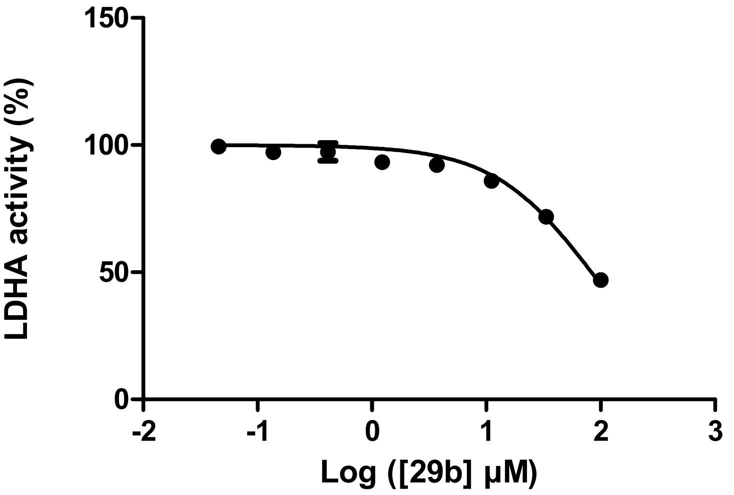

Figure S12. LDHA inhibition curve of compound **29b** (mean ± SD of *n* = 3 replicates)

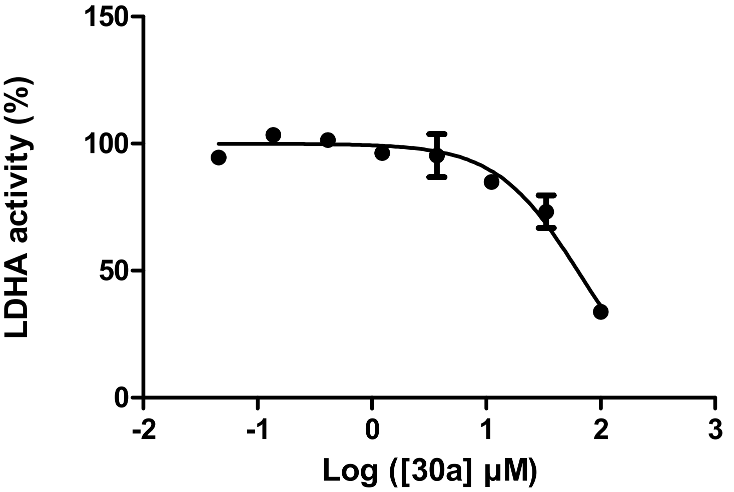

Figure S13. LDHA inhibition curve of compound **30a** (mean ± SD of *n* = 3 replicates)

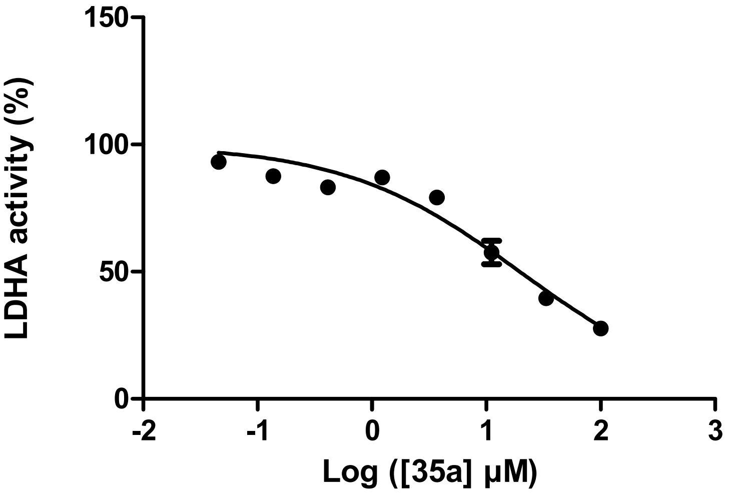

Figure S14. LDHA inhibition curve of compound **35a** (mean ± SD of *n* = 3 replicates)

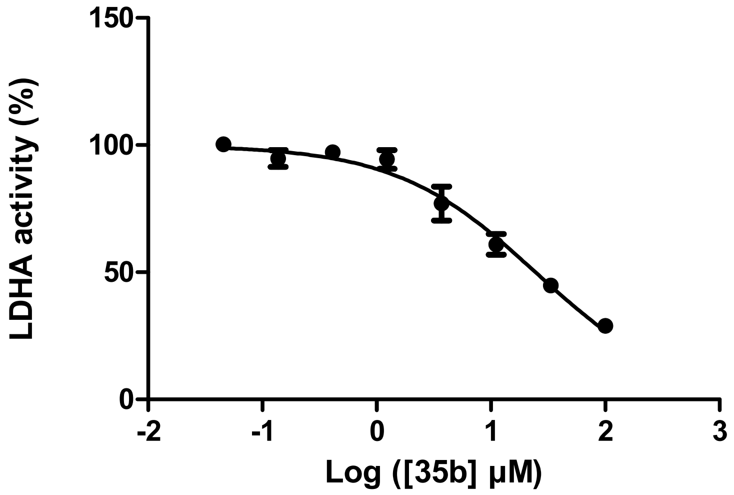

Figure S15. LDHA inhibition curve of compound **35b** (mean ± SD of *n* = 3 replicates)

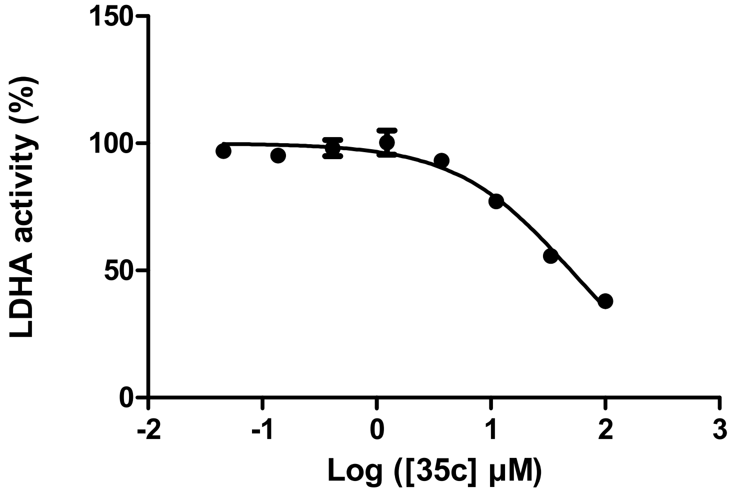

Figure S16. LDHA inhibition curve of compound **35c** (mean ± SD of *n* = 3 replicates)

4. NMR, MS and HRMS spectra.

4.1 Compound 8

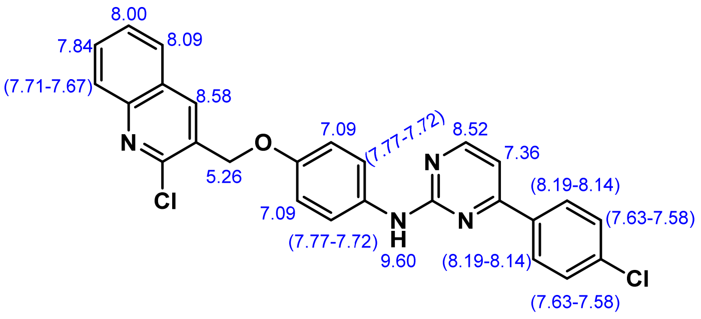

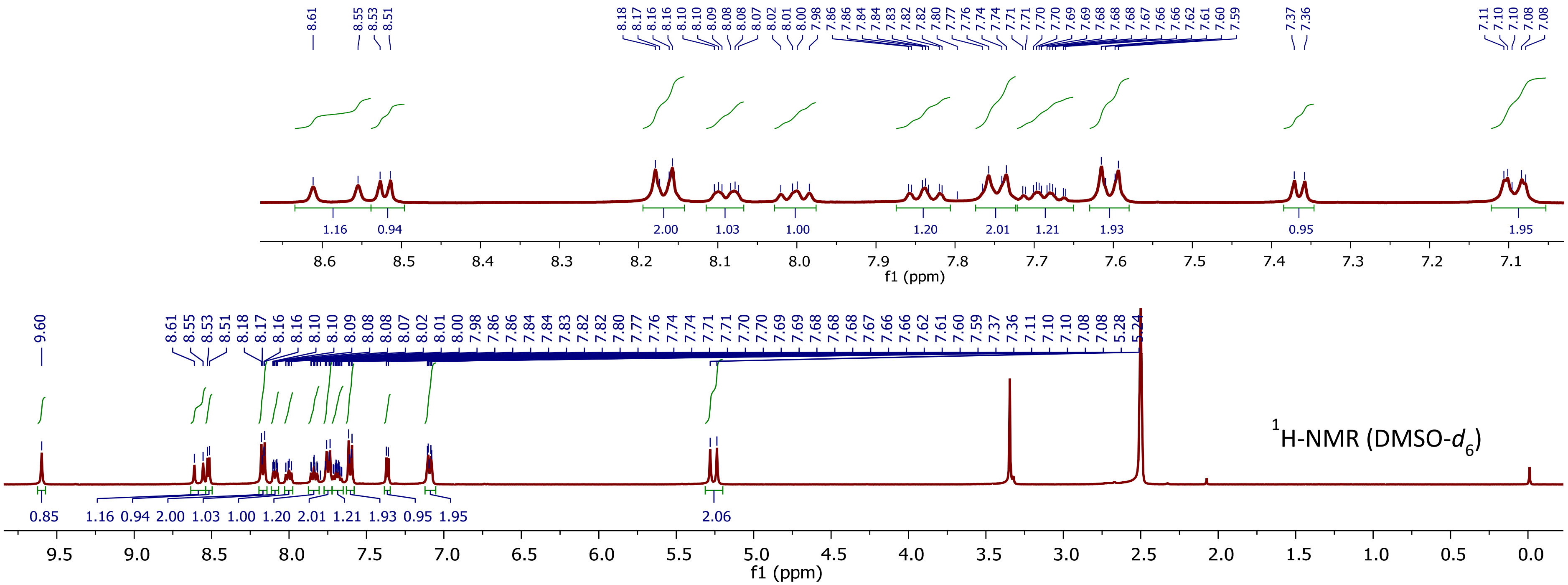

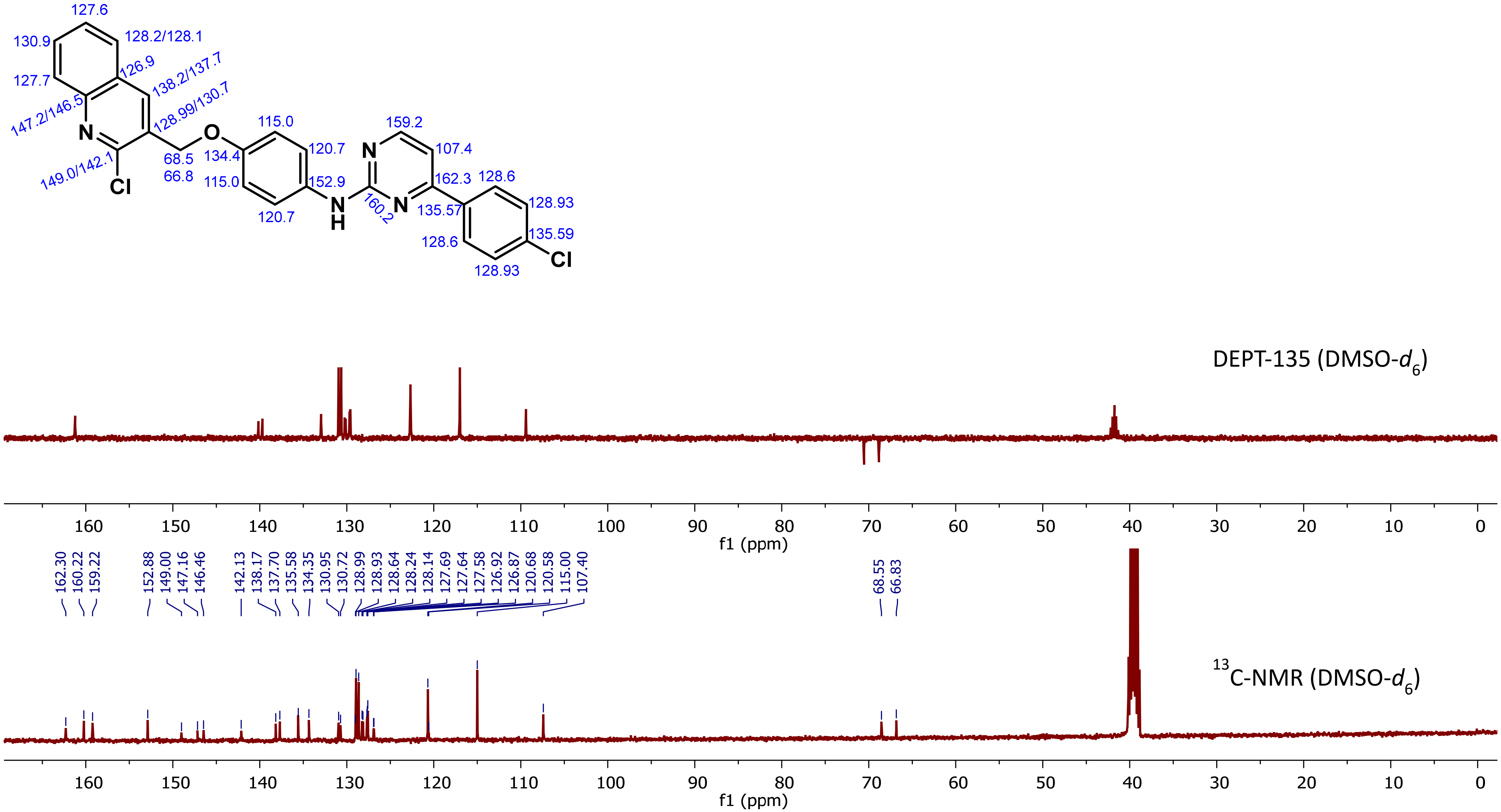

DEPT-135 (DMSO-d_6)
^{13}C-NMR (DMSO-d_6)
f1 (ppm)
162.30 160.22 159.22 152.88 149.00 147.16 146.46 142.13 138.17 137.70 135.58 134.35 130.95 130.72 128.99 128.93 128.64 128.24 128.14 127.69 127.64 127.58 126.92 126.87 120.68 120.58 115.00 107.40 68.55 66.83

EI MS (70eV)

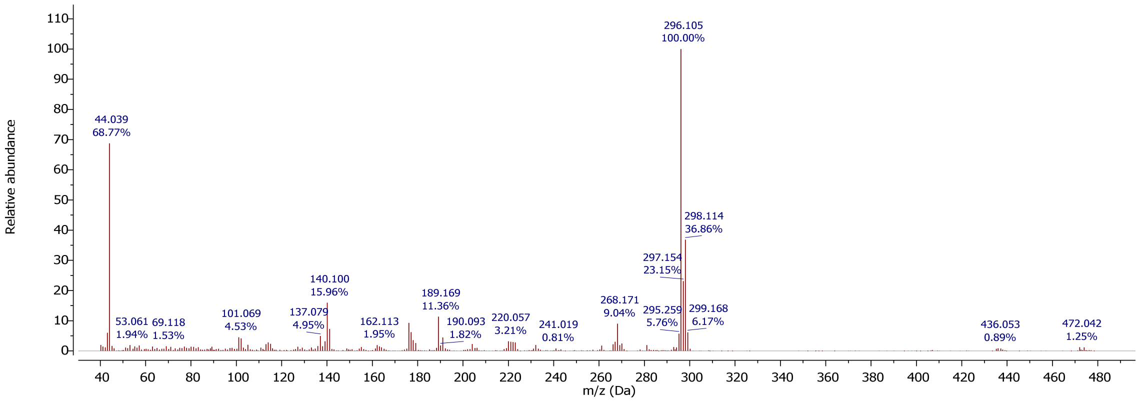

ESI-QTOF (positive ionization)

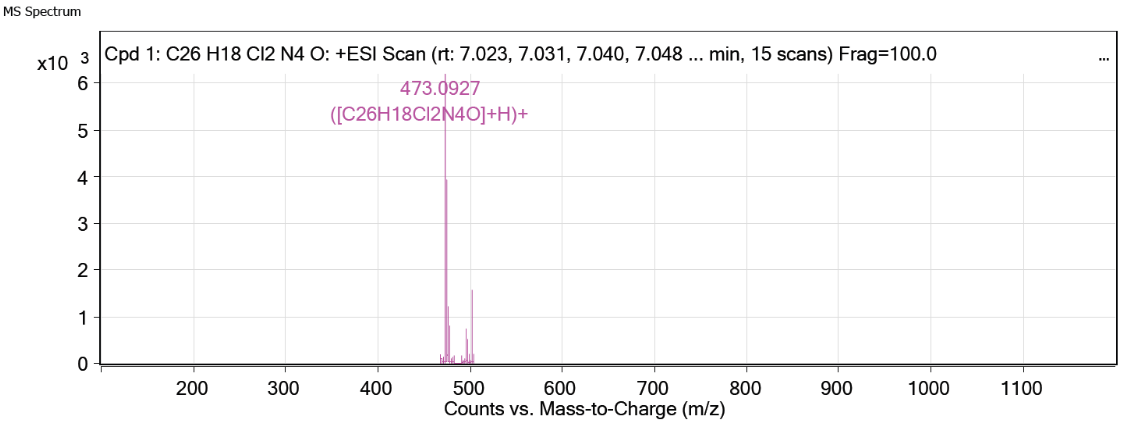

MS Spectrum Peak List

m/z	Calc *m/z*	Diff(ppm)	z	Abund	Formula	Ion
473.0927	473.093	0.76	1	6219.32	C26H18Cl2N4O	(M+H)+
474.0961	474.0961	0.12	1	1828.22	C26H18Cl2N4O	(M+H)+
475.0904	475.0907	0.57	1	4097	C26H18Cl2N4O	(M+H)+
476.0932	476.0934	0.26	1	1239.43	C26H18Cl2N4O	(M+H)+
477.09	477.0892	-1.84	1	857.27	C26H18Cl2N4O	(M+H)+
478.0927	478.091	-3.67	1	181.88	C26H18Cl2N4O	(M+H)+
495.0751	495.075	-0.15	1	756.54	C26H18Cl2N4O	(M+Na)+
496.0769	496.0781	2.29	1	195.74	C26H18Cl2N4O	(M+Na)+
497.0737	497.0726	-2.1	1	510.59	C26H18Cl2N4O	(M+Na)+
499.0814	499.0711	-20.67	1	153.04	C26H18Cl2N4O	(M+Na)+

4.2 Compound 9

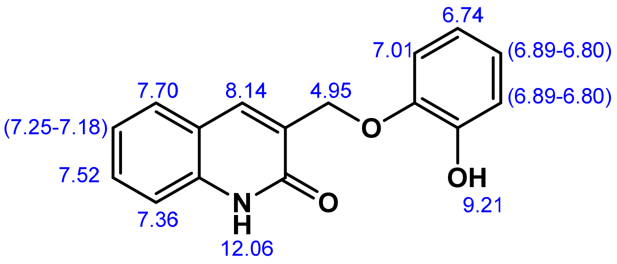

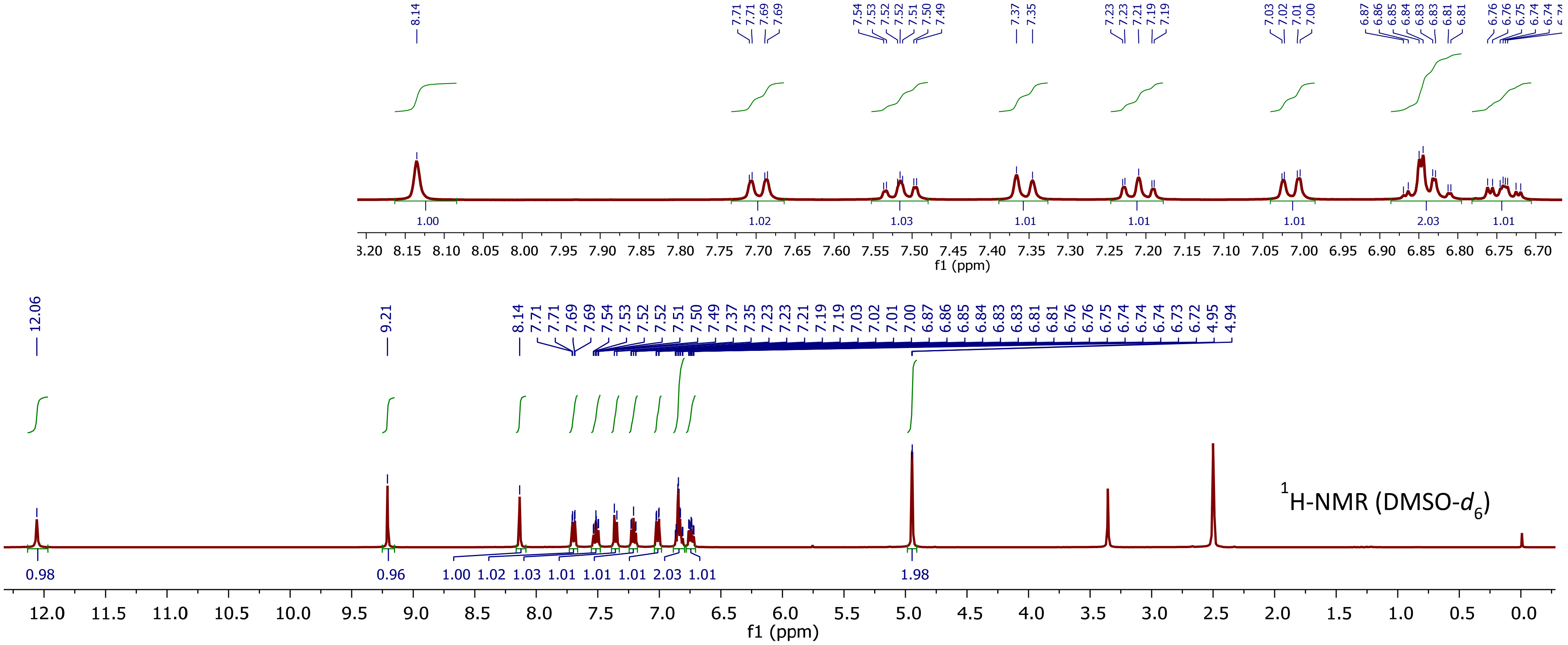

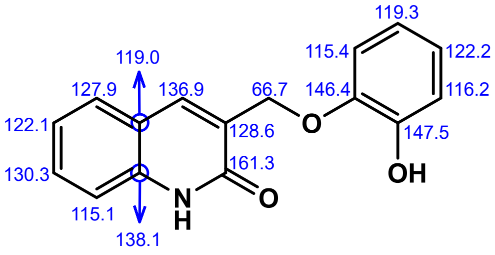
119.3
115.4
122.2
119.0
127.9
136.9
66.7
146.4
116.2
122.1
128.6
147.5
130.3
161.3
OH
O
N
H
115.1
138.1

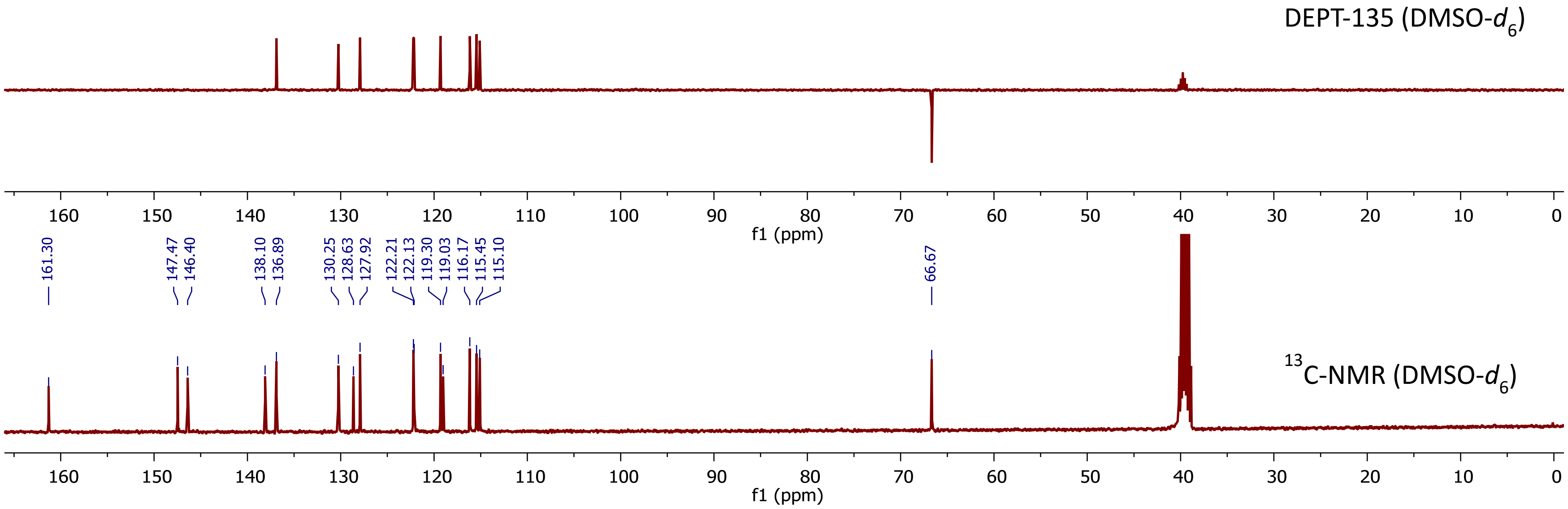
DEPT-135 (DMSO-d_6)
160
150
140
130
120
110
100
90
80
70
60
50
40
30
20
10
0
f1 (ppm)
161.30
147.47
146.40
138.10
136.89
130.25
128.63
127.92
122.21
122.13
119.30
119.03
116.17
115.45
115.10
66.67
^{13}C-NMR (DMSO-d_6)

EI MS (70eV)

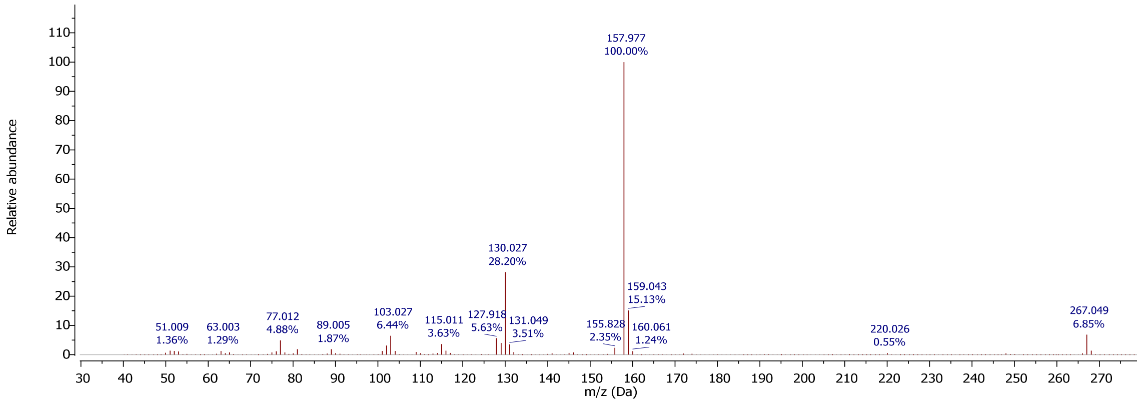

ESI-QTOF (positive ionization)

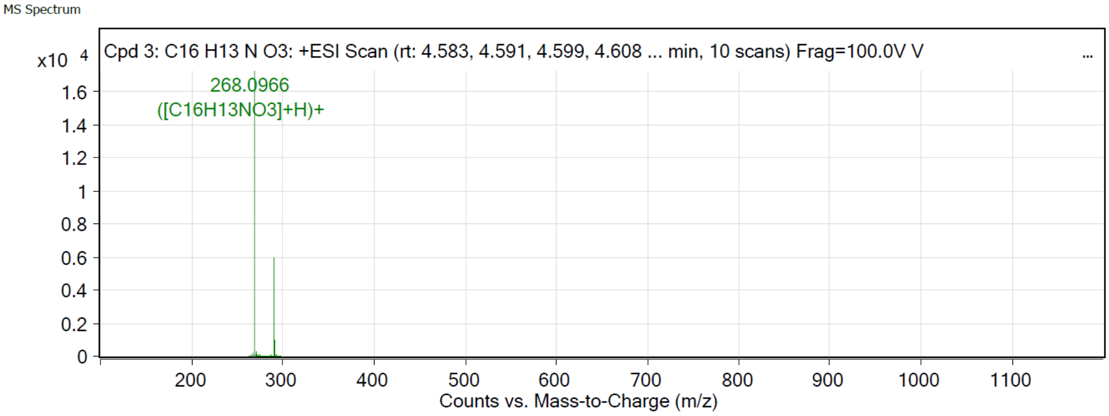

MS Spectrum Peak List

m/z	Calc *m/z*	Diff(ppm)	z	Abund	Formula	Ion
267.088	267.089	3.55	1	127.47	C16H13NO3	M+
268.0966	268.0968	0.73	1	17277.36	C16H13NO3	(M+H)+
269.1	269.1001	0.37	1	3074.52	C16H13NO3	(M+H)+
270.1029	270.1027	-0.75	1	357.6	C16H13NO3	(M+H)+
290.0779	290.0788	2.93	1	6088.36	C16H13NO3	(M+Na)+
291.0816	291.082	1.48	1	1041.68	C16H13NO3	(M+Na)+
292.082	292.0846	9.11	1	89.78	C16H13NO3	(M+Na)+

4.3 Compound 15

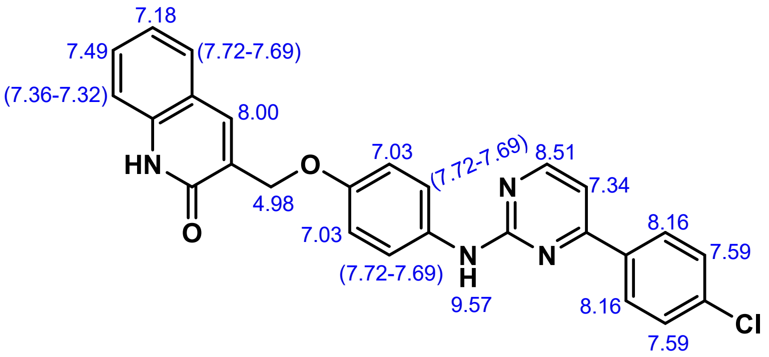

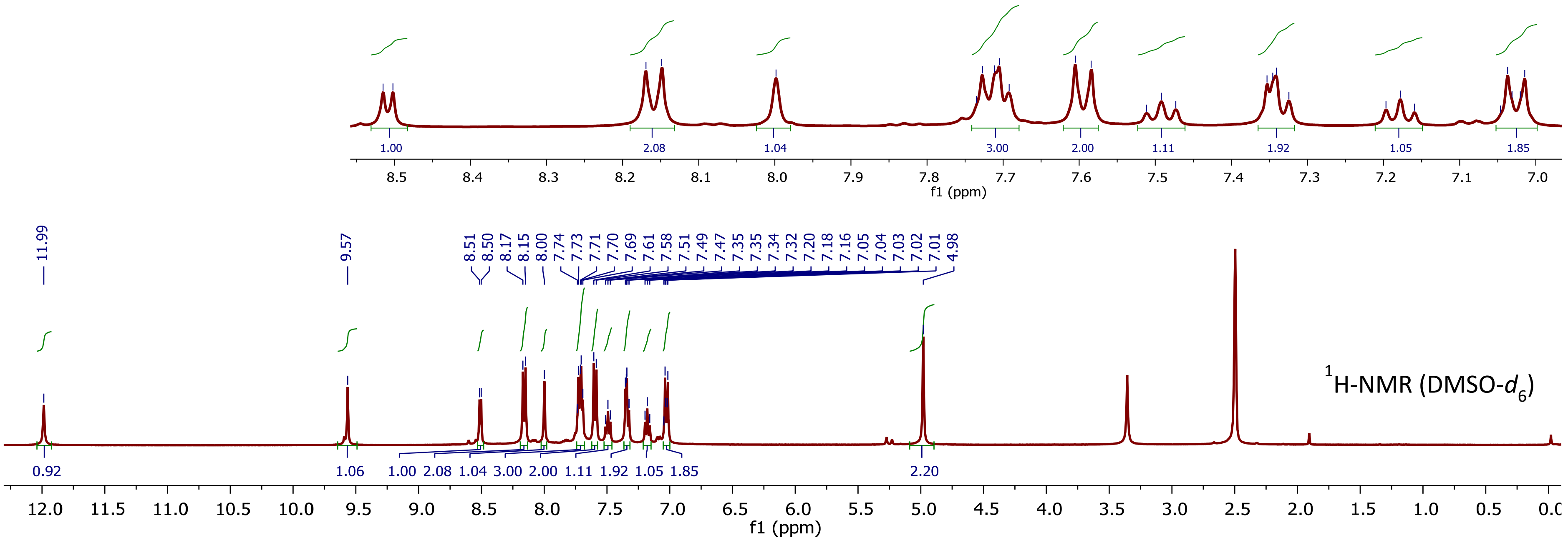

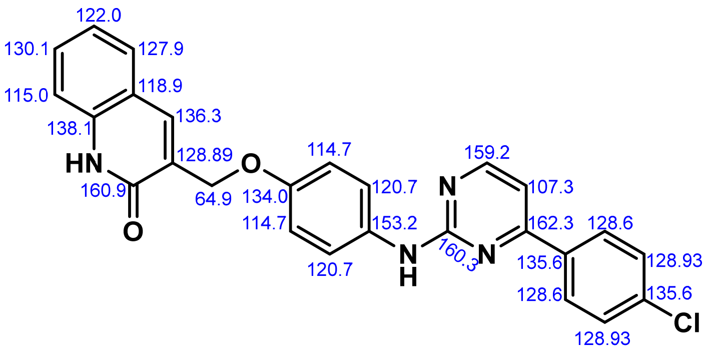
122.0
130.1
127.9
115.0
118.9
136.3
138.1
HN
128.89
O
114.7
159.2
160.9
64.9
134.0
120.7
N
107.3
O
114.7
153.2
162.3
128.6
N
160.3
N
H
135.6
128.93
120.7
128.6
135.6
128.93
Cl

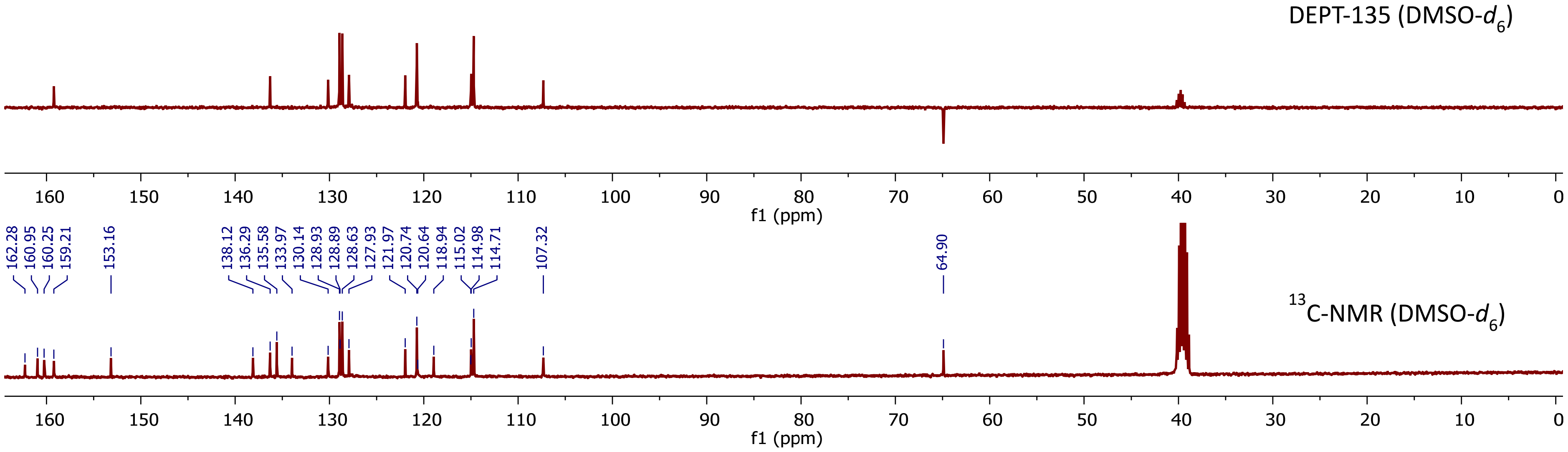
DEPT-135 (DMSO-d_6)
160 150 140 130 120 110 100 90 80 70 60 50 40 30 20 10 0
f1 (ppm)
162.28
160.95
160.25
159.21
153.16
138.12
136.29
135.58
133.97
130.14
128.93
128.89
128.63
127.93
121.97
120.74
120.64
118.94
115.02
114.98
114.71
107.32
64.90
^{13}C-NMR (DMSO-d_6)
160 150 140 130 120 110 100 90 80 70 60 50 40 30 20 10 0
f1 (ppm)

EI MS (70eV)

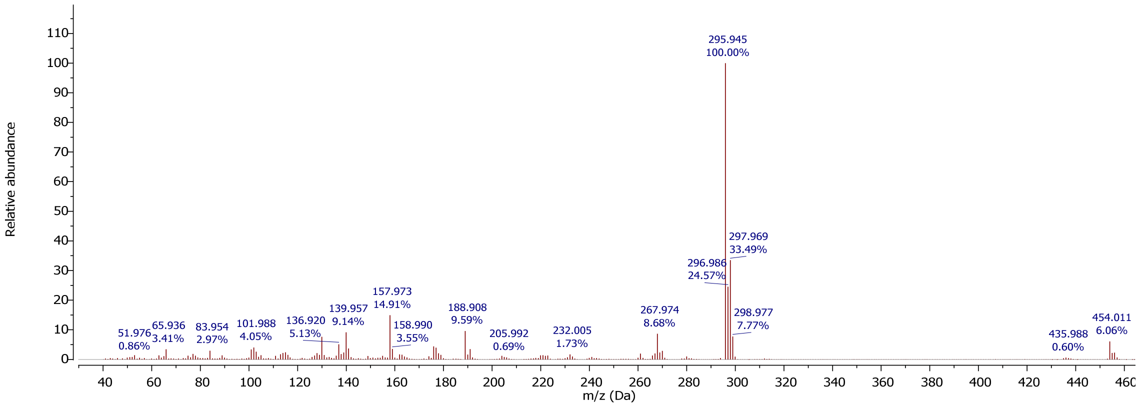

ESI-QTOF (positive ionization)

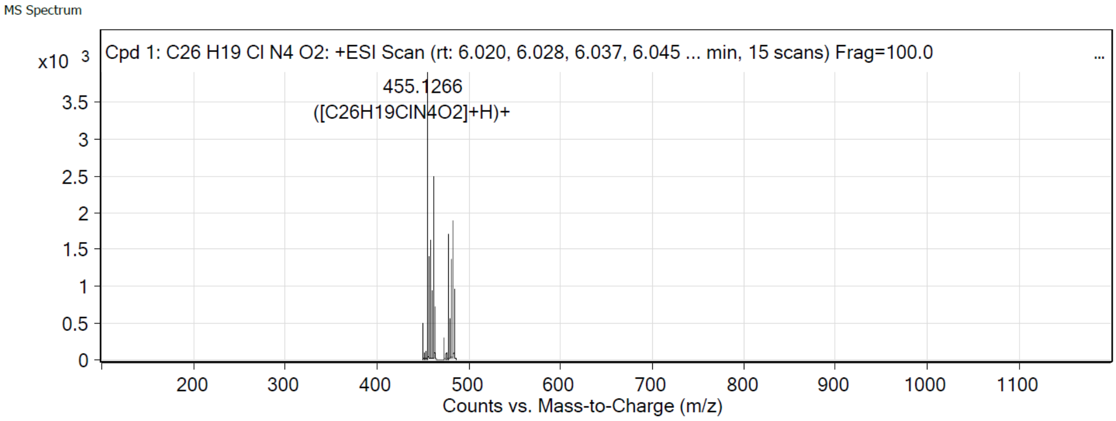

MS Spectrum Peak List

m/z	Calc *m/z*	Diff(ppm)	z	Abund	Formula	Ion
455.1266	455.1269	0.7	1	3951.15	C26H19ClN4O2	(M+H)+
456.1289	456.13	2.41	1	1176.77	C26H19ClN4O2	(M+H)+
457.1247	457.1251	0.9	1	1488.47	C26H19ClN4O2	(M+H)+
458.1277	458.1275	-0.53	1	395.03	C26H19ClN4O2	(M+H)+
459.1327	459.1302	-5.56	1	40.66	C26H19ClN4O2	(M+H)+
477.1082	477.1089	1.34	1	1772.62	C26H19ClN4O2	(M+Na)+
478.1116	478.1119	0.66	1	450.63	C26H19ClN4O2	(M+Na)+
479.1066	479.1071	1.08	1	540.46	C26H19ClN4O2	(M+Na)+
480.109	480.1094	0.9	1	127.29	C26H19ClN4O2	(M+Na)+

4.4 Compound 19

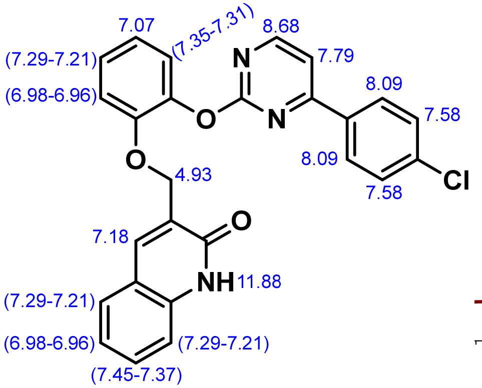

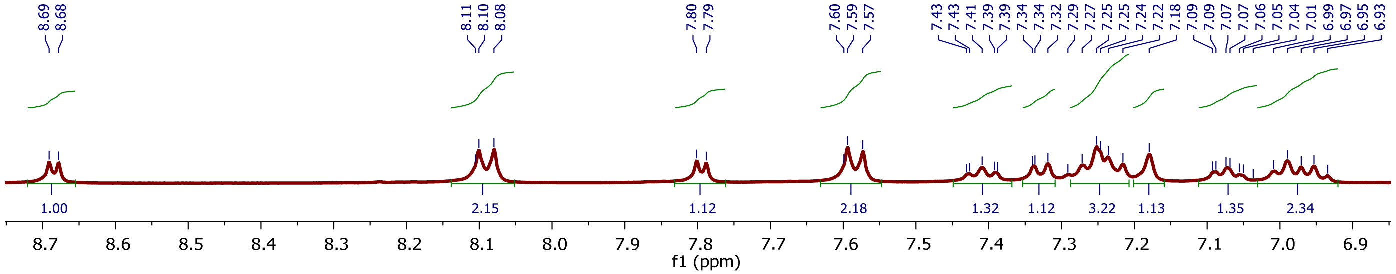

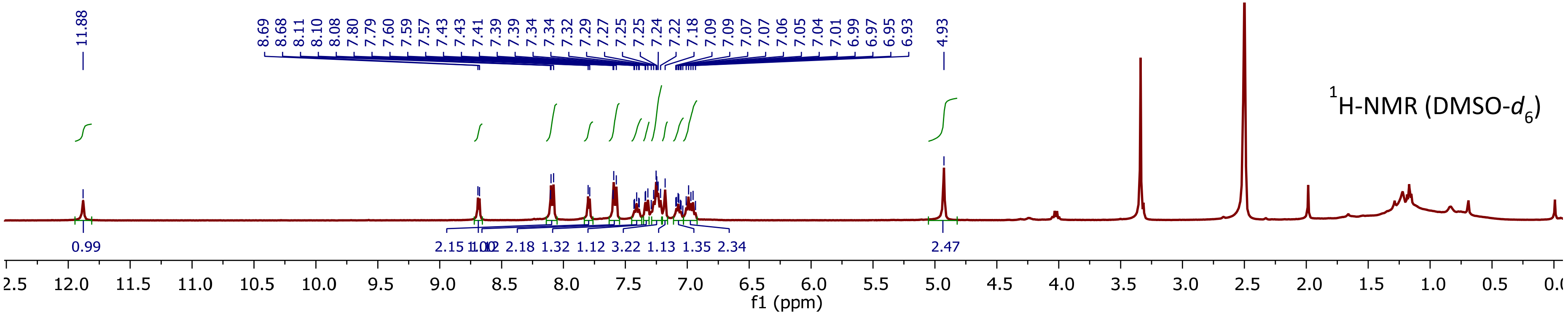

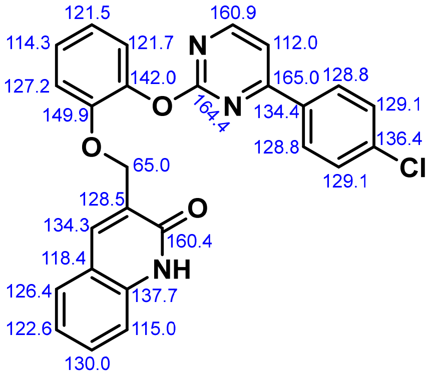
121.5
160.9
114.3
121.7
N
112.0
127.2
142.0
165.0
128.8
O
164.4
N
134.4
129.1
149.9
136.4
128.8
Cl
O
129.1
65.0
128.5
O
134.3
160.4
118.4
NH
126.4
137.7
122.6
115.0
130.0

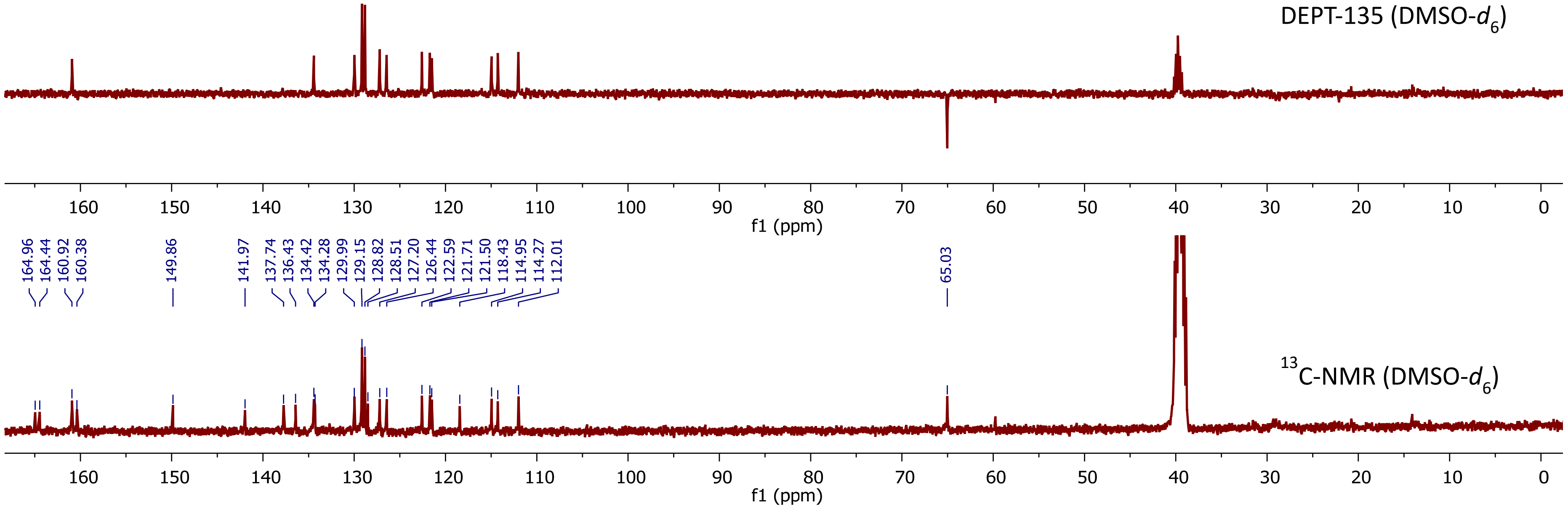
DEPT-135 (DMSO-d_6)
160
150
140
130
120
110
100
90
80
70
60
50
40
30
20
10
0
f1 (ppm)
164.96
164.44
160.92
160.38
149.86
141.97
137.74
136.43
134.42
134.28
129.99
129.15
128.82
128.51
127.20
126.44
122.59
121.71
121.50
118.43
114.95
114.27
112.01
65.03
^{13}C-NMR (DMSO-d_6)
160
150
140
130
120
110
100
90
80
70
60
50
40
30
20
10
0
f1 (ppm)

EI MS (70eV)

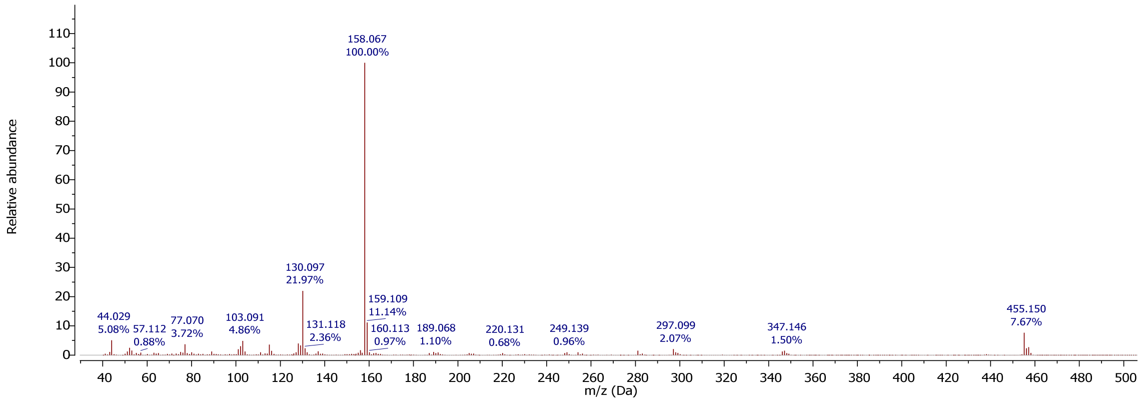

ESI-QTOF (positive ionization)

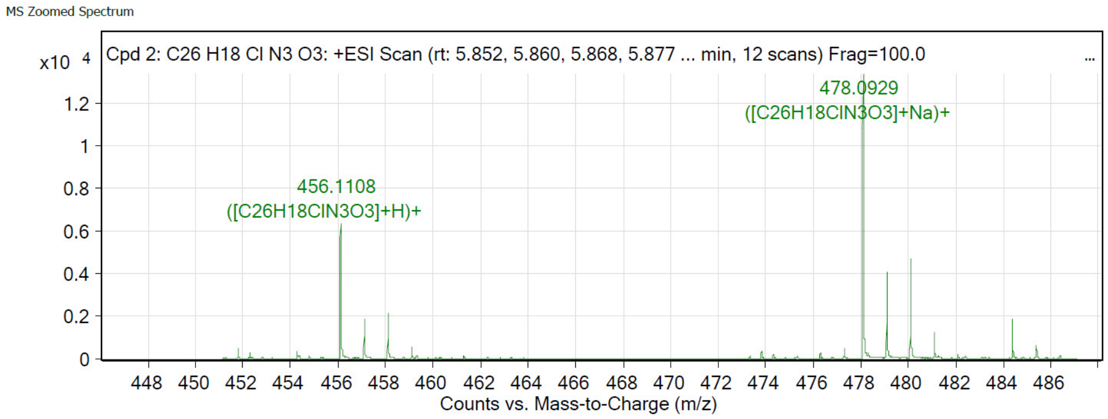

MS Spectrum Peak List

m/z	Calc *m/z*	Diff(ppm)	z	Abund	Formula	Ion
456.1108	456.1109	0.21	1	6375.96	C26H18ClN3O3	(M+H)+
457.1138	457.1141	0.65	1	1920.26	C26H18ClN3O3	(M+H)+
458.1088	458.1092	0.86	1	2208.63	C26H18ClN3O3	(M+H)+
459.1117	459.1116	-0.2	1	576.29	C26H18ClN3O3	(M+H)+
460.1171	460.1143	-6.16	1	20.83	C26H18ClN3O3	(M+H)+
478.0929	478.0929	-0.05	1	13461.33	C26H18ClN3O3	(M+Na)+
479.0956	479.096	0.83	1	4169.89	C26H18ClN3O3	(M+Na)+
480.091	480.0911	0.23	1	4700.13	C26H18ClN3O3	(M+Na)+
481.0925	481.0936	2.24	1	1299.51	C26H18ClN3O3	(M+Na)+
482.0944	482.0962	3.73	1	194.51	C26H18ClN3O3	(M+Na)+

4.5 Compound 22a

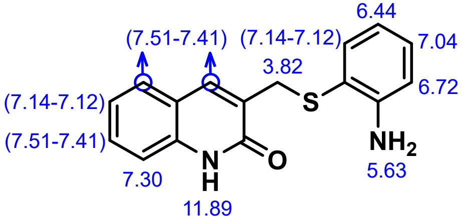

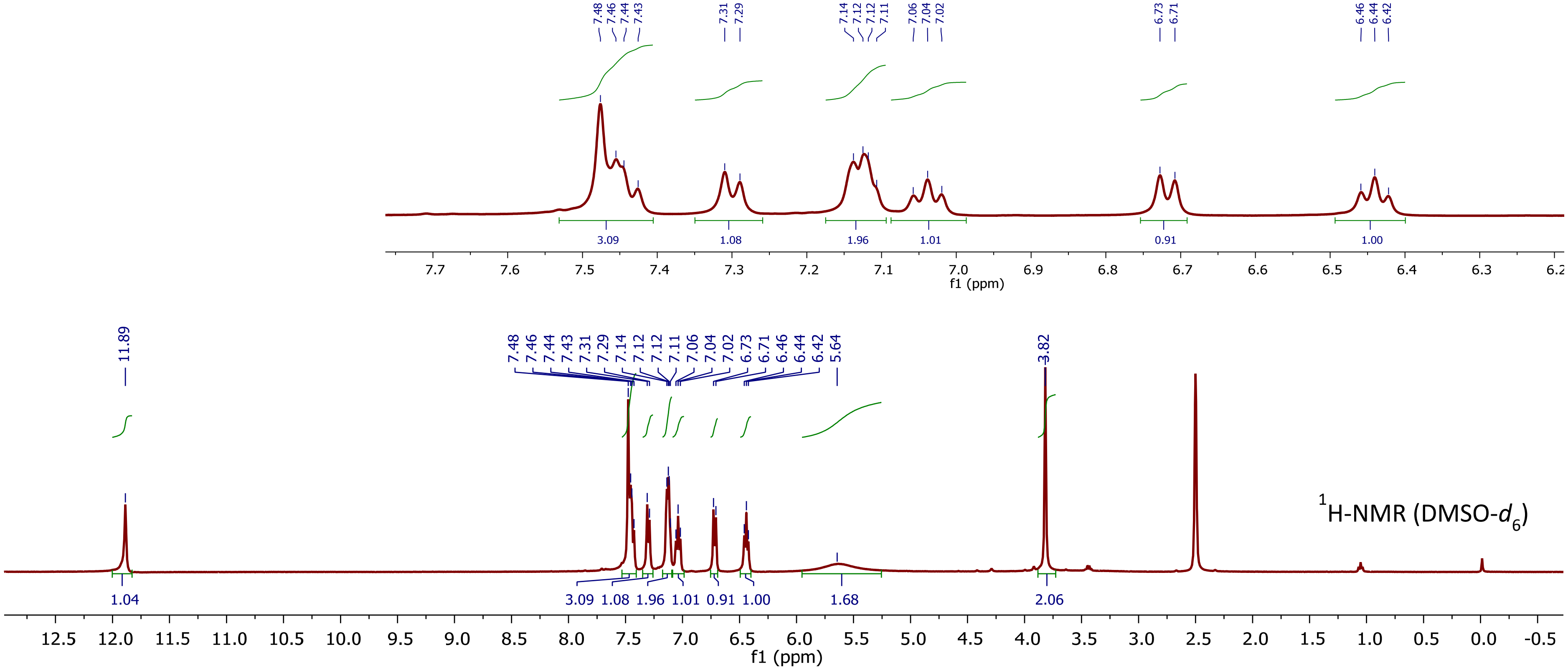

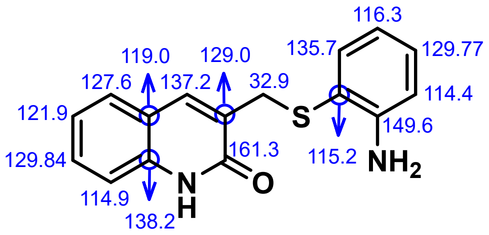
116.3
135.7
129.77
119.0
129.0
127.6
137.2
32.9
114.4
121.9
S
149.6
161.3
129.84
115.2
NH2
N
O
114.9
H
138.2

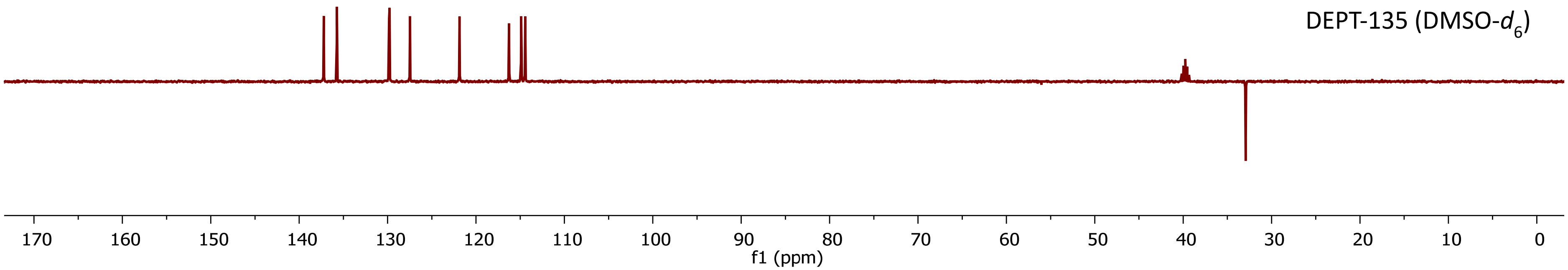
DEPT-135 (DMSO-d6)
170 160 150 140 130 120 110 100 90 80 70 60 50 40 30 20 10 0
f1 (ppm)

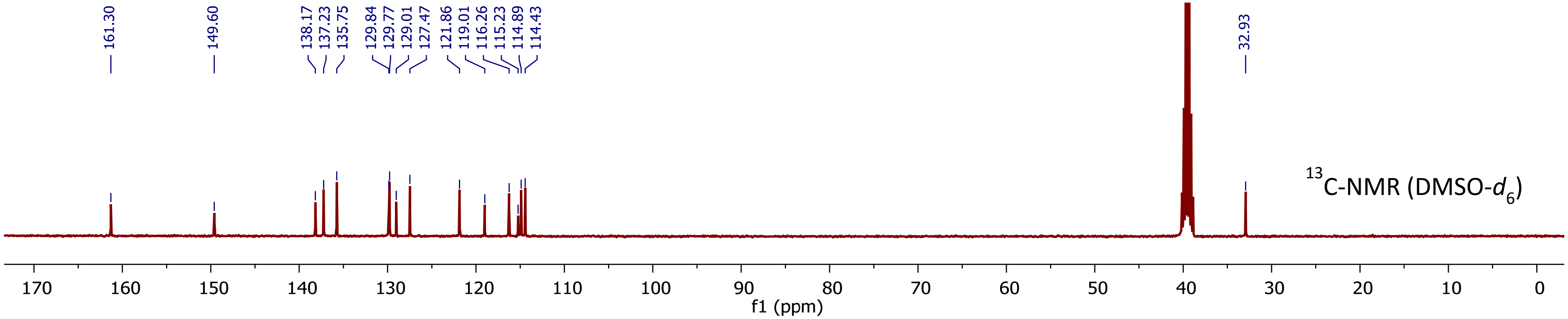
161.30
149.60
138.17
137.23
135.75
129.84
129.77
129.01
127.47
121.86
119.01
116.26
115.23
114.89
114.43
32.93
13C-NMR (DMSO-d6)
170 160 150 140 130 120 110 100 90 80 70 60 50 40 30 20 10 0
f1 (ppm)

EI MS (70eV)

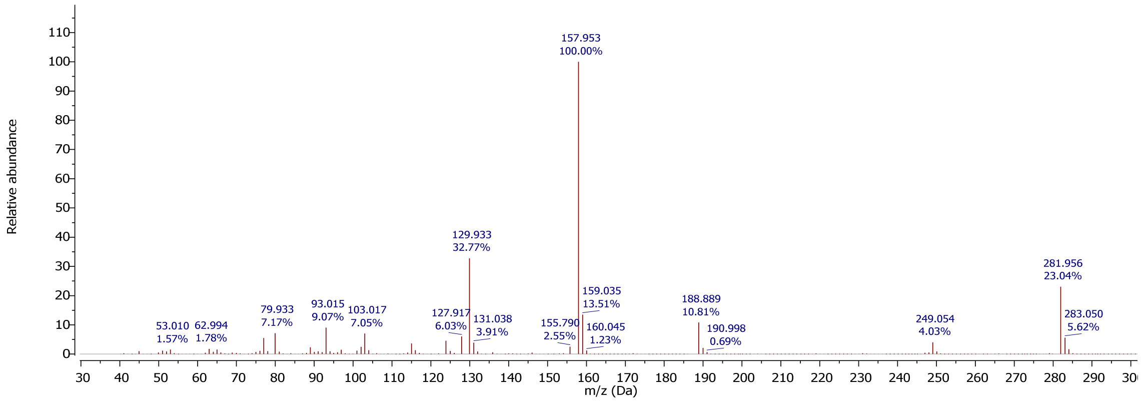

ESI-QTOF (positive ionization)

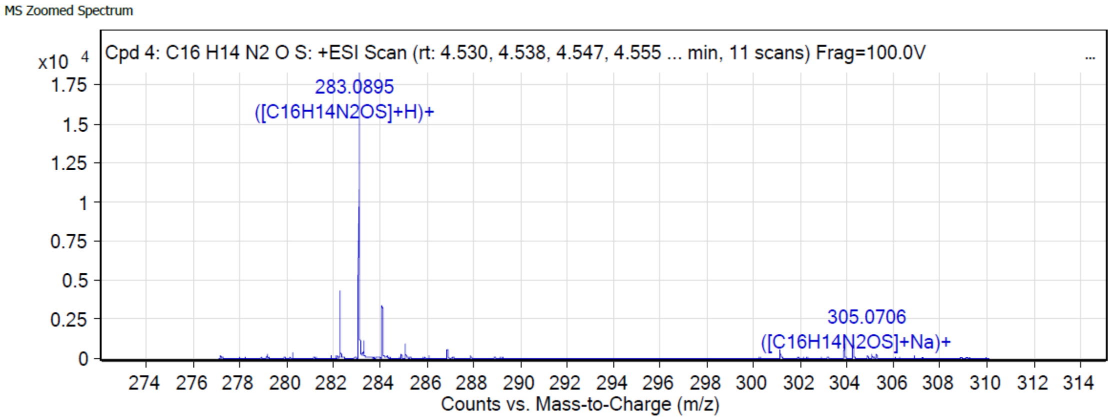

MS Spectrum Peak List

m/z	Calc *m/z*	Diff(ppm)	z	Abund	Formula	Ion
282.0818	282.0821	1.11	1	137.98	C16H14N2OS	M+
283.0895	283.09	1.54	1	18691.6	C16H14N2OS	(M+H)+
284.0925	284.0929	1.36	1	3518.1	C16H14N2OS	(M+H)+
285.0894	285.0887	-2.3	1	1059.53	C16H14N2OS	(M+H)+
305.0706	305.0719	4.29	1	309.66	C16H14N2OS	(M+Na)+

4.6 Compound 22b

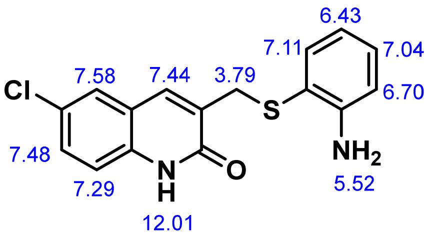

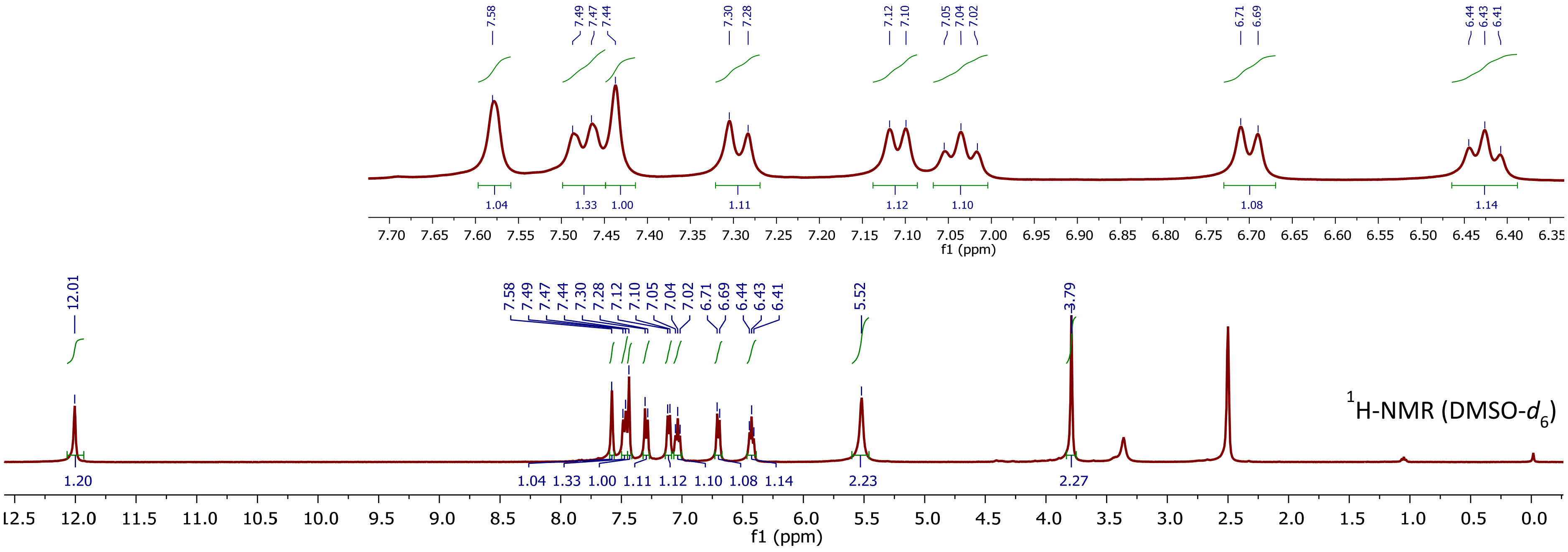

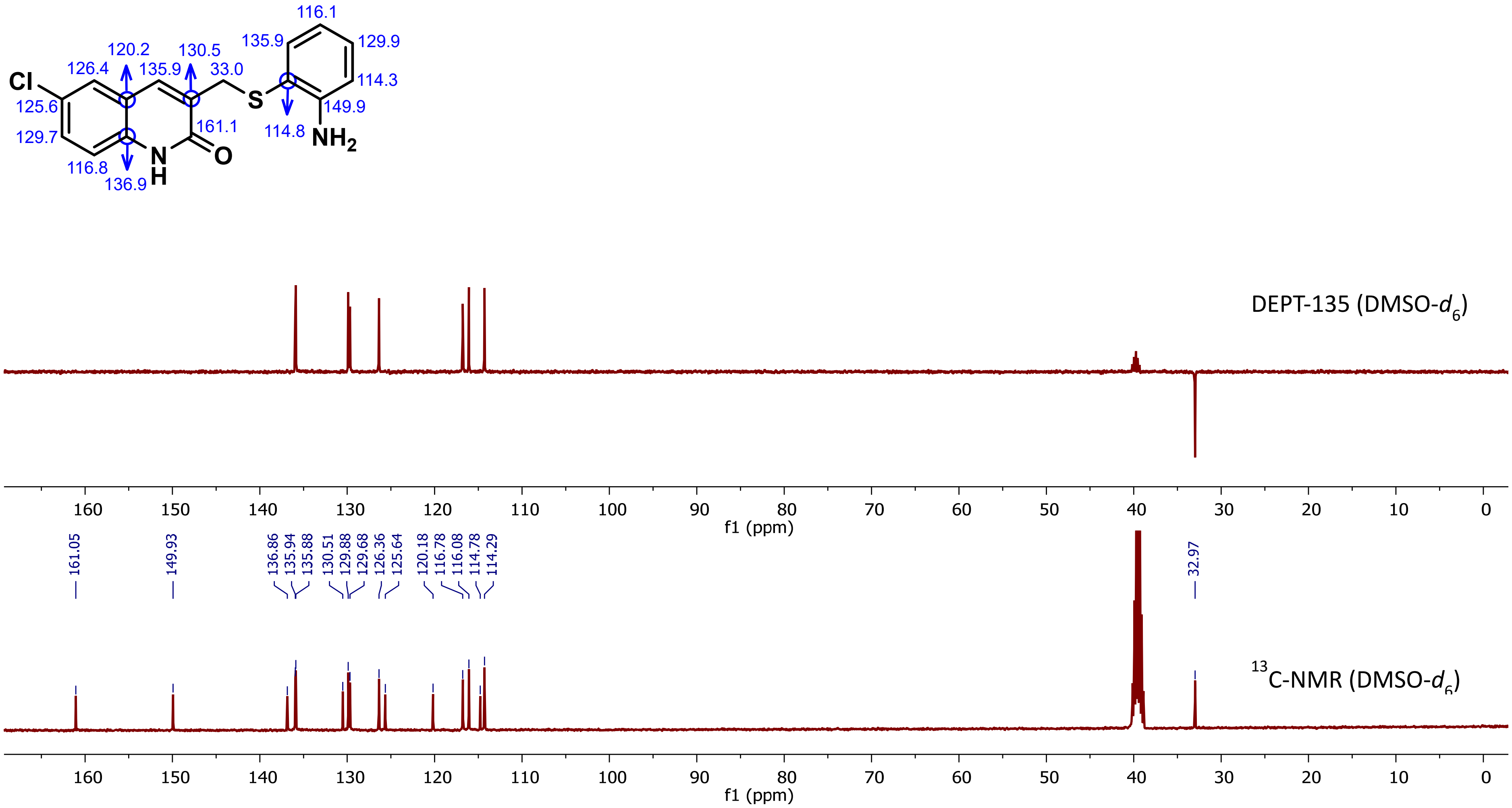

120.2
130.5
116.1
135.9
129.9
126.4
135.9
33.0
Cl
125.6
114.3
149.9
129.7
161.1
114.8
NH2
116.8
N
H
O
S
136.9
DEPT-135 (DMSO-d6)
f1 (ppm)
161.05
149.93
136.86
135.94
135.88
130.51
129.88
129.68
126.36
125.64
120.18
116.78
116.08
114.78
114.29
32.97
13C-NMR (DMSO-d6)

EI MS (70eV)

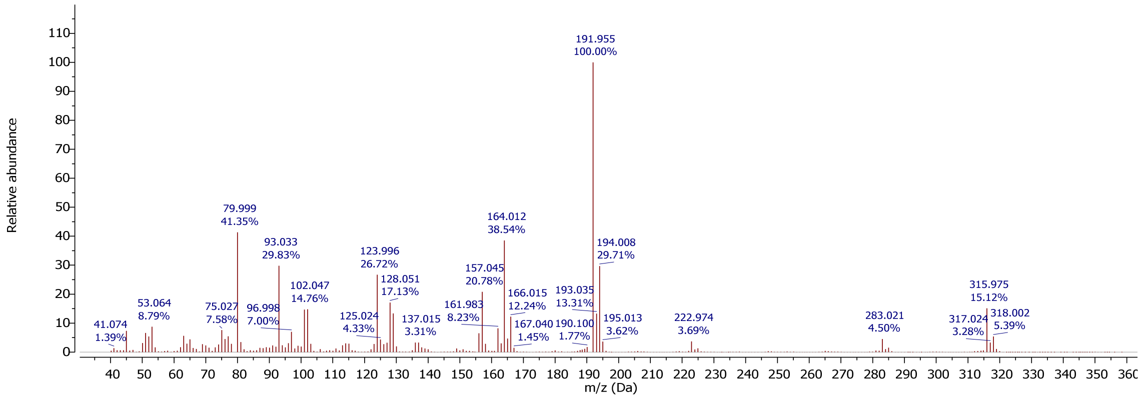

ESI-QTOF (positive ionization)

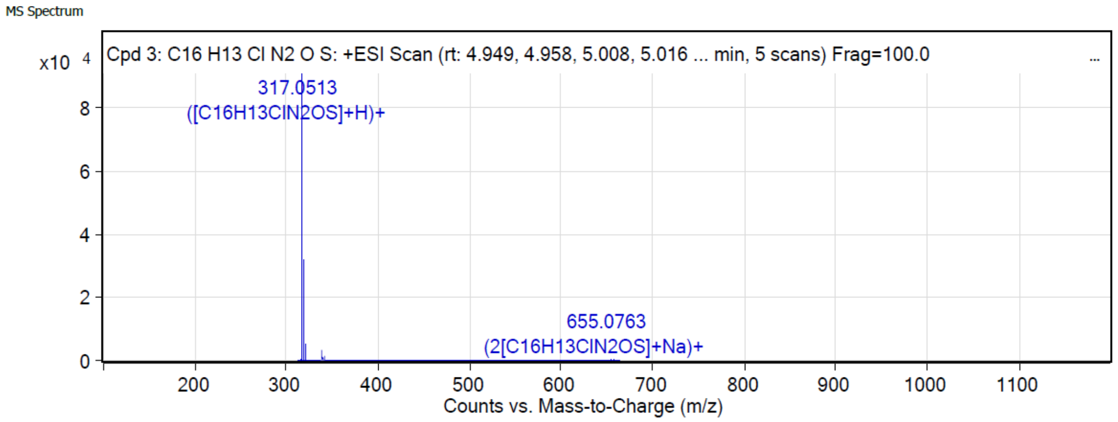

MS Spectrum Peak List

m/z	Calc *m/z*	Diff(ppm)	z	Abund	Formula	Ion
317.0513	317.051	-1.12	1	91054.87	C16H13ClN2OS	(M+H)+
318.0546	318.054	-1.99	1	15869.8	C16H13ClN2OS	(M+H)+
319.0487	319.0483	-1.11	1	31865.69	C16H13ClN2OS	(M+H)+
320.051	320.051	0.26	1	5519.68	C16H13ClN2OS	(M+H)+
321.0472	321.0471	-0.25	1	1600.66	C16H13ClN2OS	(M+H)+
339.0331	339.0329	-0.36	1	3268.07	C16H13ClN2OS	(M+Na)+
340.035	340.0359	2.52	1	831.13	C16H13ClN2OS	(M+Na)+
341.0312	341.0303	-2.87	1	1435.39	C16H13ClN2OS	(M+Na)+
655.0763	655.0766	0.5	1	451.57	C16H13ClN2OS	(2M+Na)+
657.0732	657.0744	1.72	1	361.43	C16H13ClN2OS	(2M+Na)+

4.7 Compound 22c

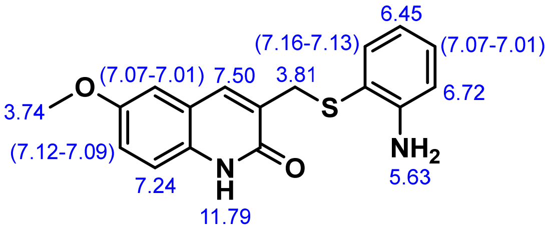

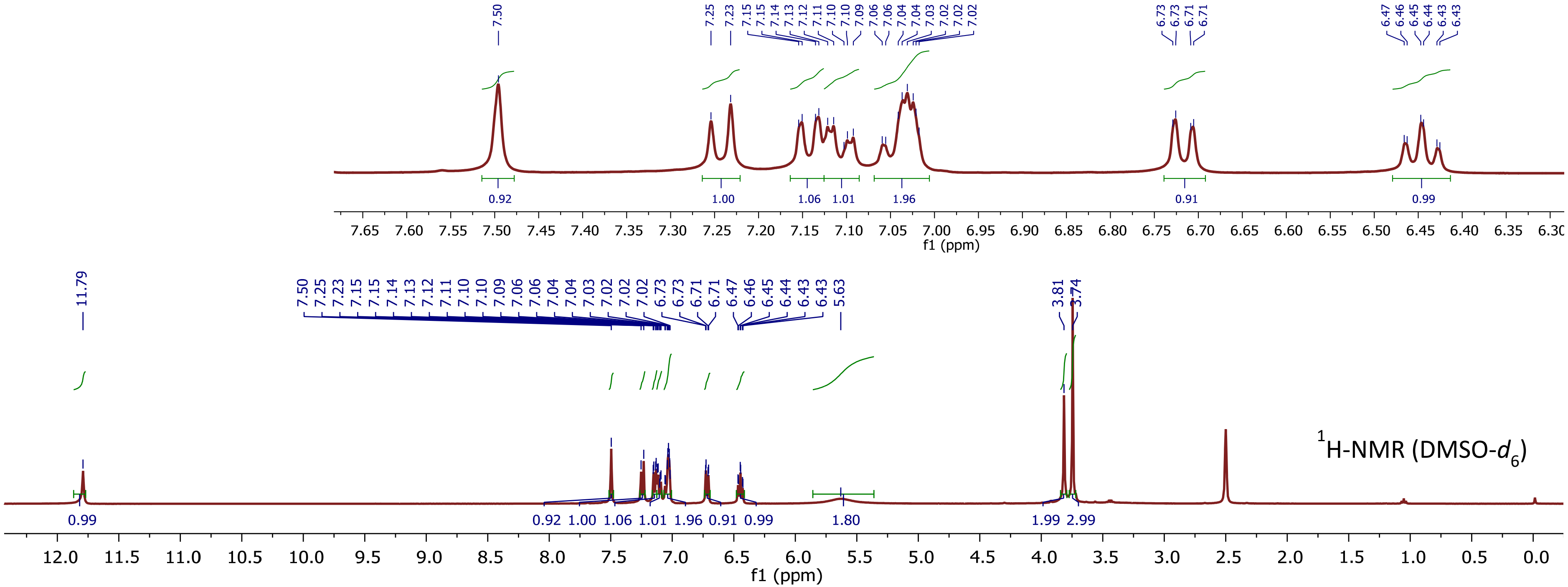

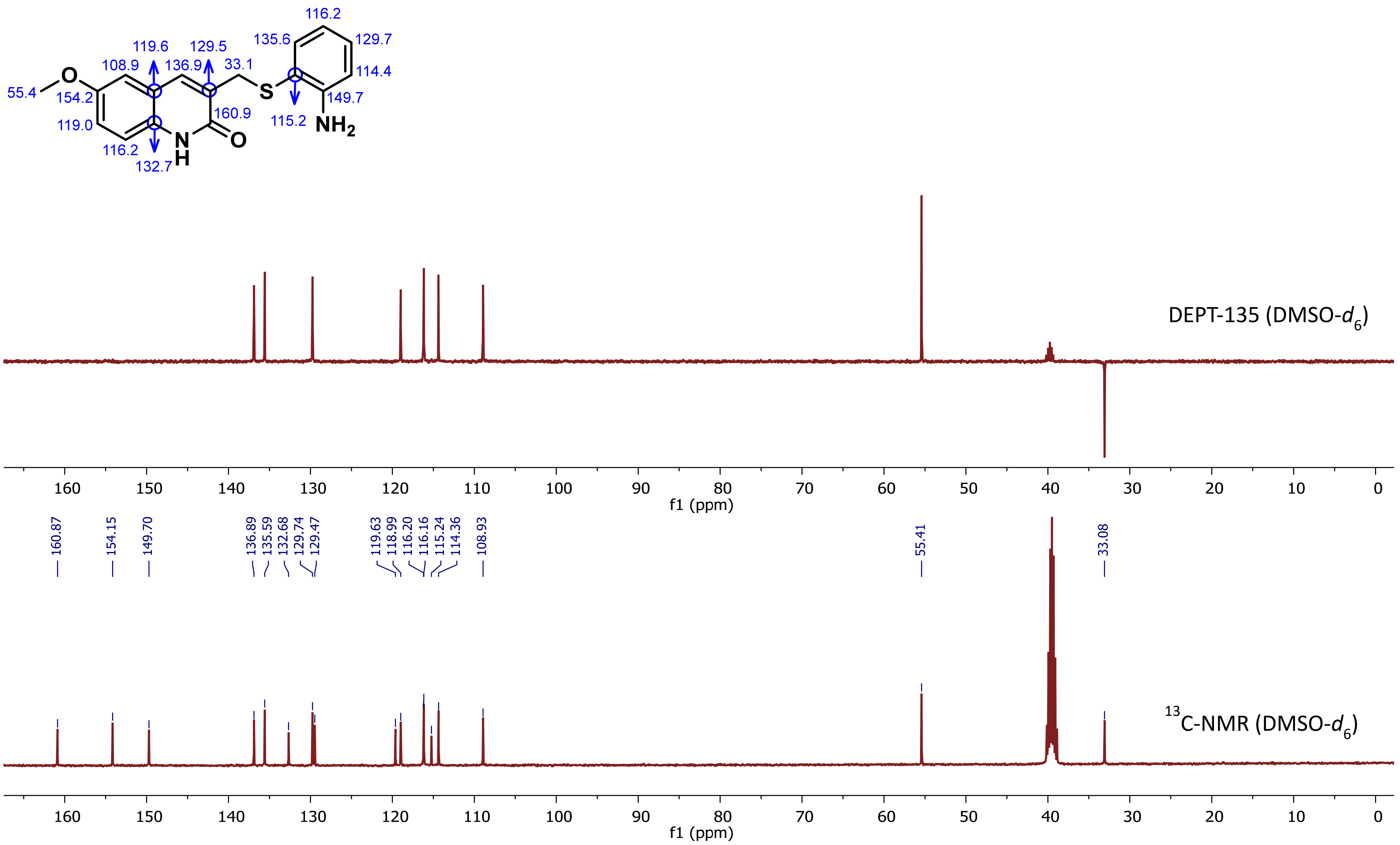
116.2
135.6
129.7
119.6
129.5
108.9
136.9
33.1
114.4
55.4
154.2
149.7
160.9
119.0
115.2
116.2
132.7
DEPT-135 (DMSO-d_6)
f1 (ppm)
160.87
154.15
149.70
136.89
135.59
132.68
129.74
129.47
119.63
118.99
116.20
116.16
115.24
114.36
108.93
55.41
33.08
^{13}C-NMR (DMSO-d_6)
f1 (ppm)

EI MS (70eV)

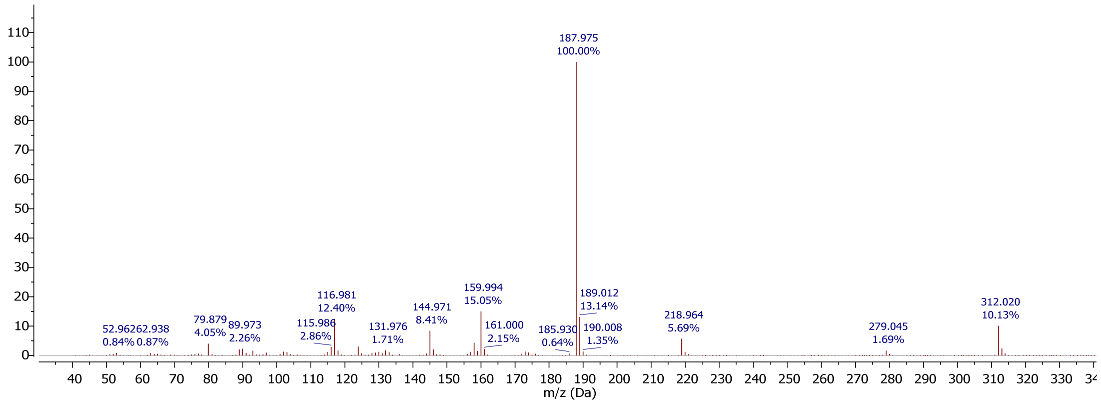

ESI-QTOF (positive ionization)

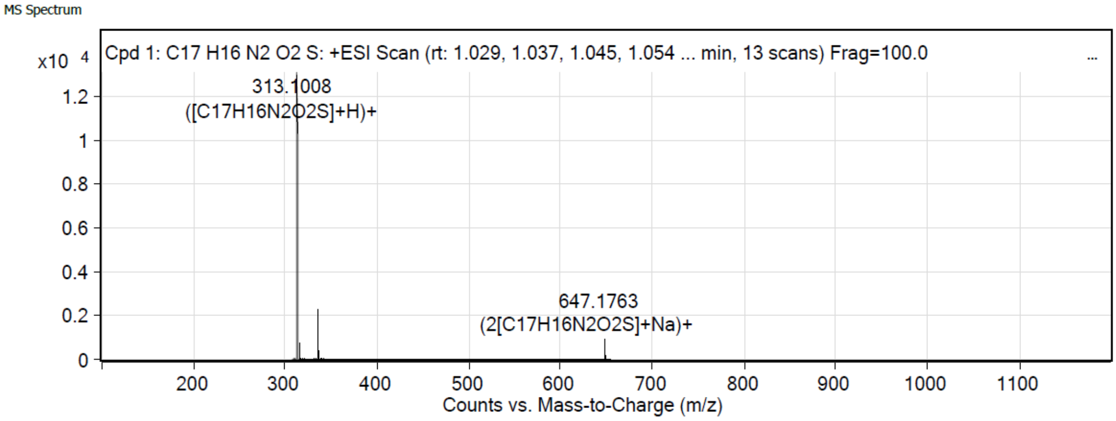

MS Spectrum Peak List

m/z	Calc *m/z*	Diff(ppm)	z	Abund	Formula	Ion
313.1008	313.1005	-1.02	1	13382.66	C17H16N2O2S	(M+H)+
314.1039	314.1035	-1.22	1	2447.19	C17H16N2O2S	(M+H)+
315.0996	315.0997	0.44	1	729.27	C17H16N2O2S	(M+H)+
316.0981	316.1012	9.69	1	89.5	C17H16N2O2S	(M+H)+
335.0826	335.0825	-0.45	1	2424.13	C17H16N2O2S	(M+Na)+
336.085	336.0855	1.39	1	462.95	C17H16N2O2S	(M+Na)+
337.0844	337.0817	-8.1	1	175.52	C17H16N2O2S	(M+Na)+
647.1763	647.1757	-0.84	1	988.01	C17H16N2O2S	(2M+Na)+
648.1807	648.1787	-3.08	1	355.91	C17H16N2O2S	(2M+Na)+
649.1746	649.1765	2.89	1	205.42	C17H16N2O2S	(2M+Na)+

4.8 Compound 23a

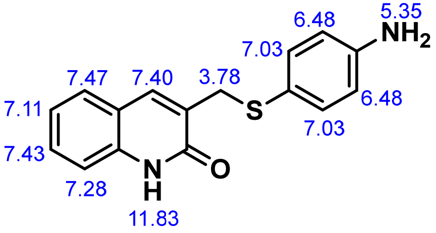

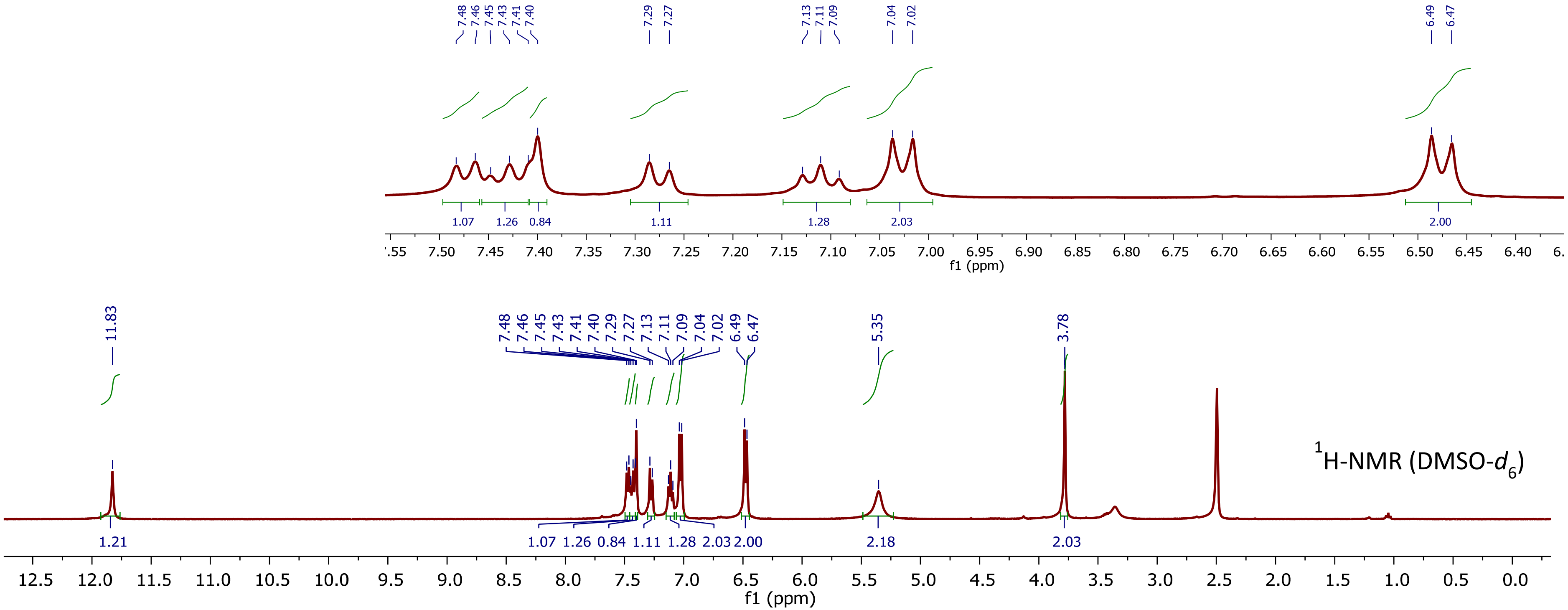

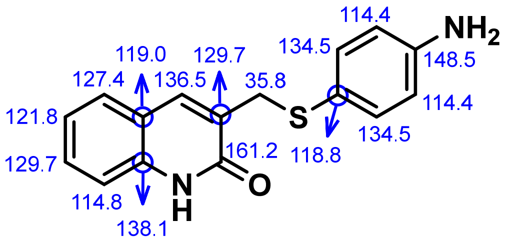

114.4
NH2
134.5
148.5
119.0
129.7
127.4
136.5
35.8
121.8
S
114.4
161.2
118.8
134.5
129.7
N
O
114.8
H
138.1

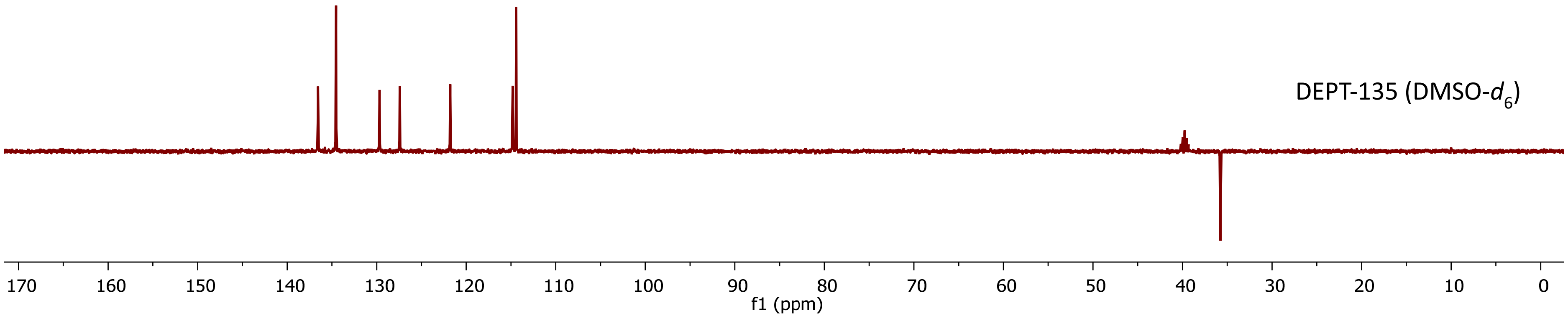

DEPT-135 (DMSO-d6)
170 160 150 140 130 120 110 100 90 80 70 60 50 40 30 20 10 0
f1 (ppm)

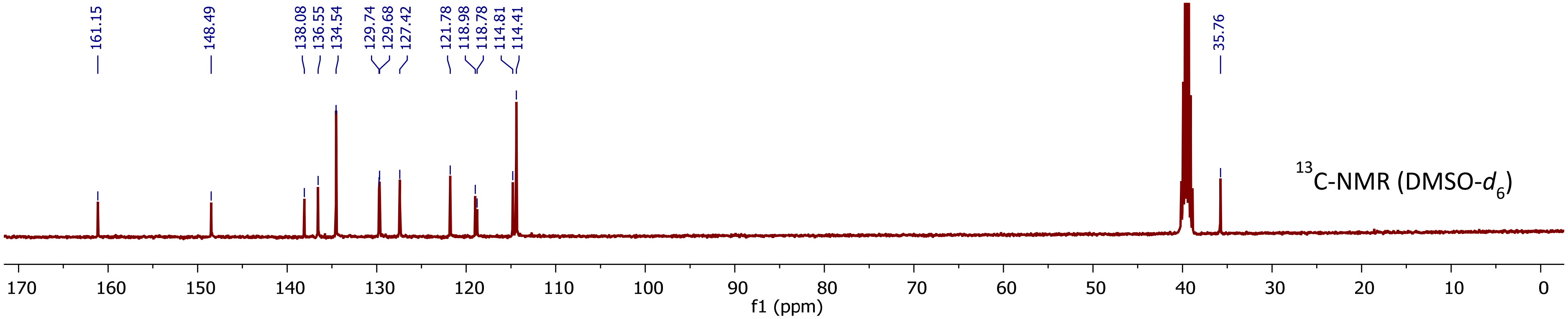

161.15
148.49
138.08
136.55
134.54
129.74
129.68
127.42
121.78
118.98
118.78
114.81
114.41
35.76
13C-NMR (DMSO-d6)
170 160 150 140 130 120 110 100 90 80 70 60 50 40 30 20 10 0
f1 (ppm)

EI MS (70eV)

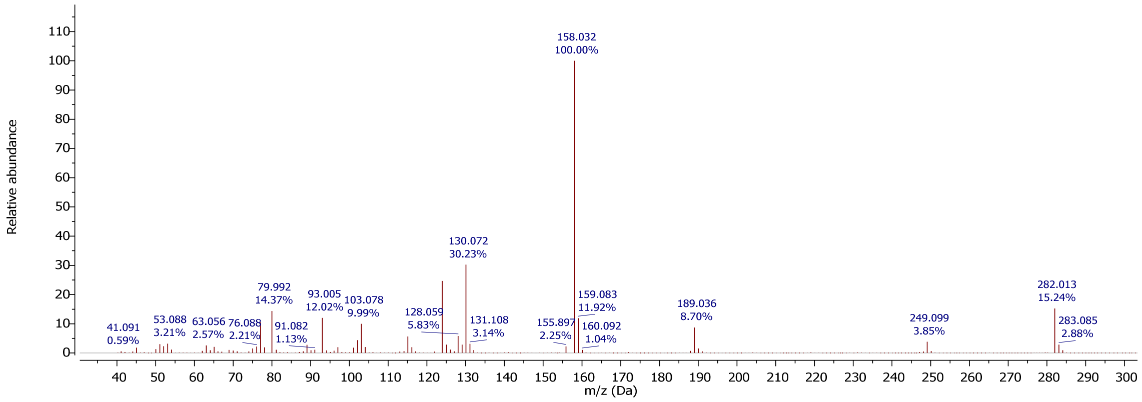

ESI-QTOF (positive ionization)

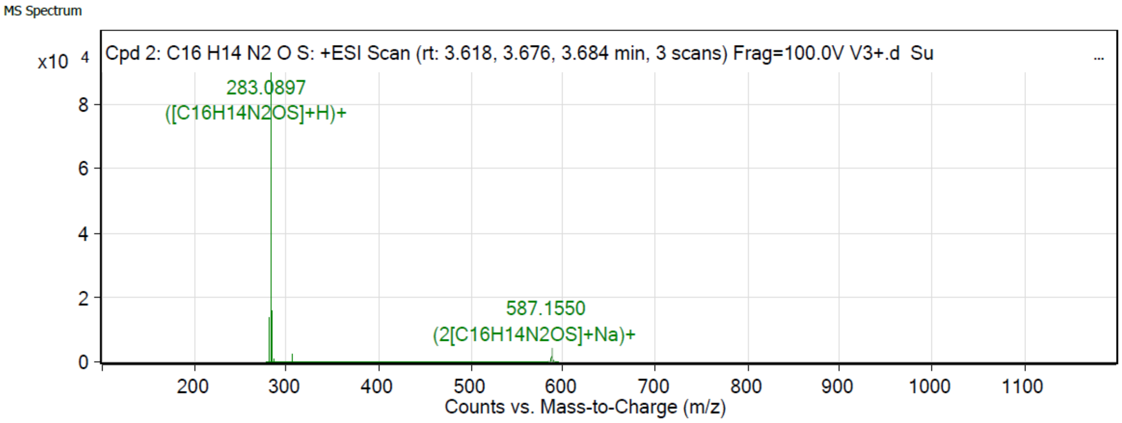

MS Spectrum Peak List

m/z	Calc *m/z*	Diff(ppm)	z	Abund	Formula	Ion
283.0897	283.09	0.78	1	90184.47	C16H14N2OS	(M+H)+
284.0929	284.0929	0.17	1	15839.54	C16H14N2OS	(M+H)+
285.0884	285.0887	1.25	1	4394.17	C16H14N2OS	(M+H)+
305.0717	305.0719	0.82	1	2215.43	C16H14N2OS	(M+Na)+
306.0746	306.0749	0.94	1	412.94	C16H14N2OS	(M+Na)+
307.0649	307.0707	18.86	1	152.5	C16H14N2OS	(M+Na)+
587.155	587.1546	-0.65	1	4516.79	C16H14N2OS	(2M+Na)+
588.1582	588.1576	-1.16	1	1762.98	C16H14N2OS	(2M+Na)+
589.1559	589.155	-1.66	1	727.91	C16H14N2OS	(2M+Na)+

4.9 Compound 23b

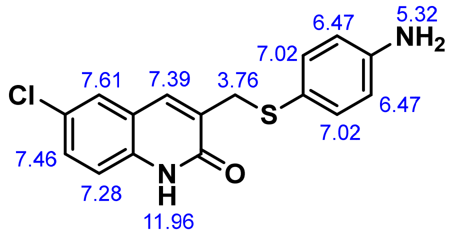

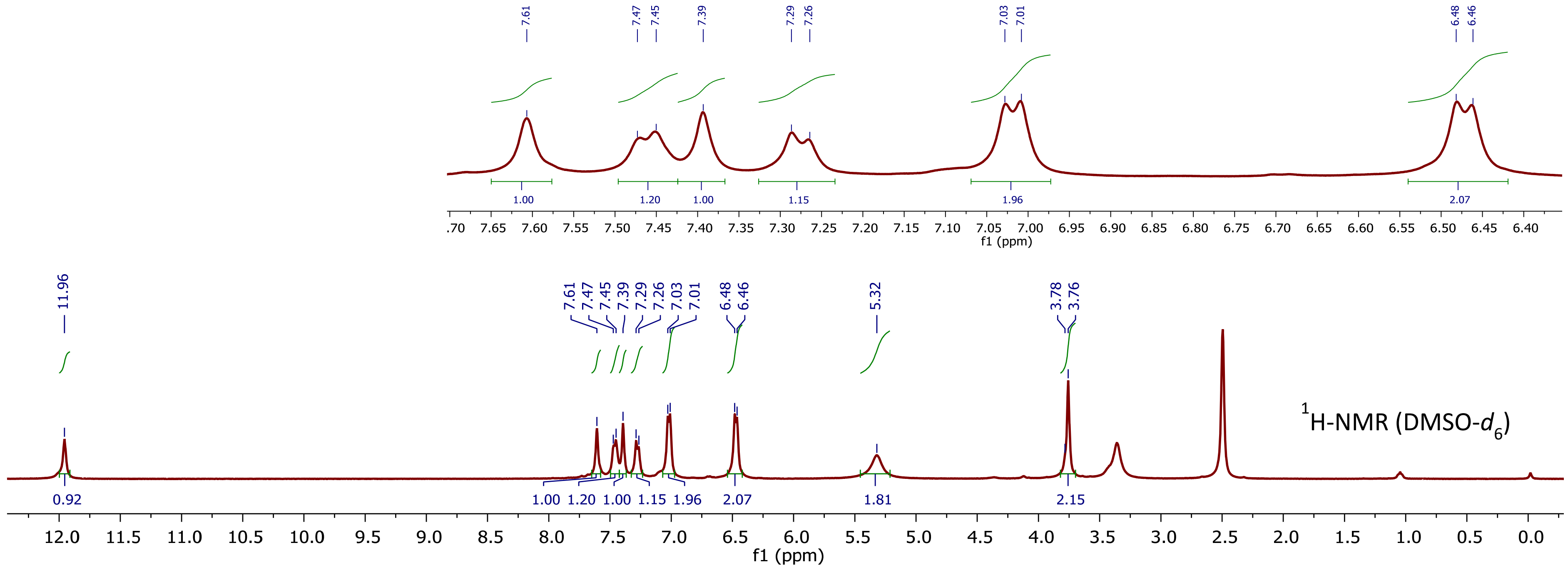

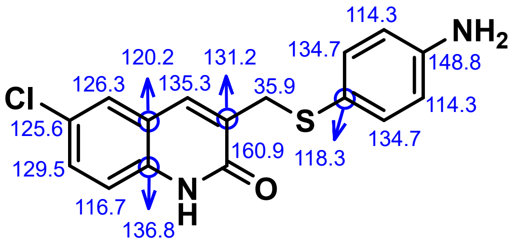

Cl
NH2
N
H
O
S
120.2
131.2
126.3
135.3
35.9
114.3
134.7
148.8
114.3
134.7
125.6
129.5
116.7
136.8
160.9
118.3

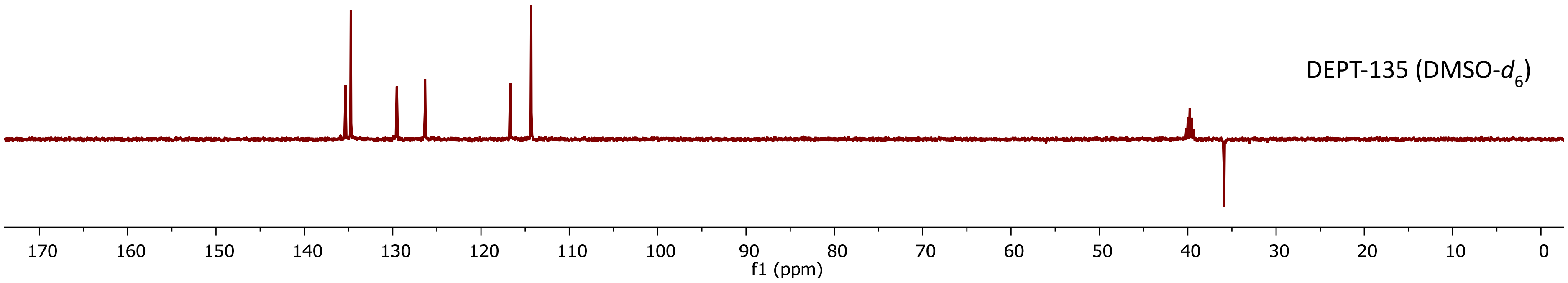

DEPT-135 (DMSO-d6)
170
160
150
140
130
120
110
100
90
80
70
60
50
40
30
20
10
0
f1 (ppm)

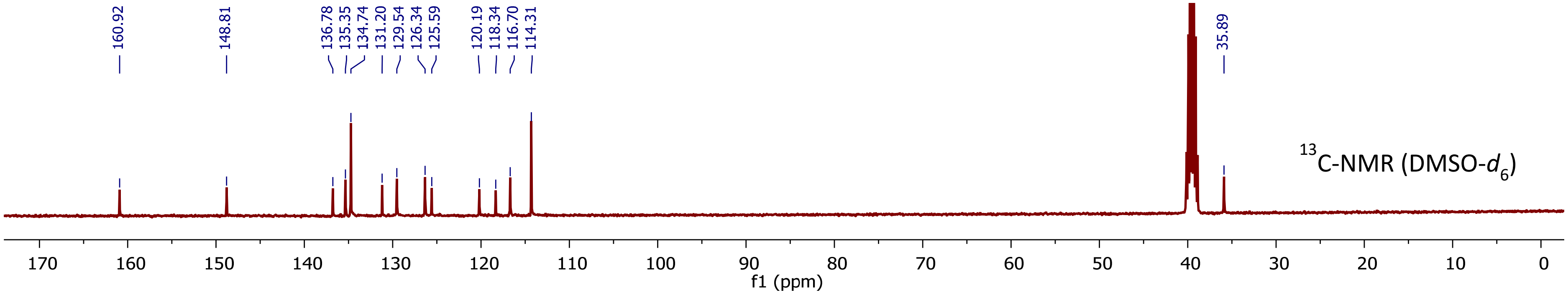

160.92
148.81
136.78
135.35
134.74
131.20
129.54
126.34
125.59
120.19
118.34
116.70
114.31
35.89
13C-NMR (DMSO-d6)
170
160
150
140
130
120
110
100
90
80
70
60
50
40
30
20
10
0
f1 (ppm)

EI MS (70eV)

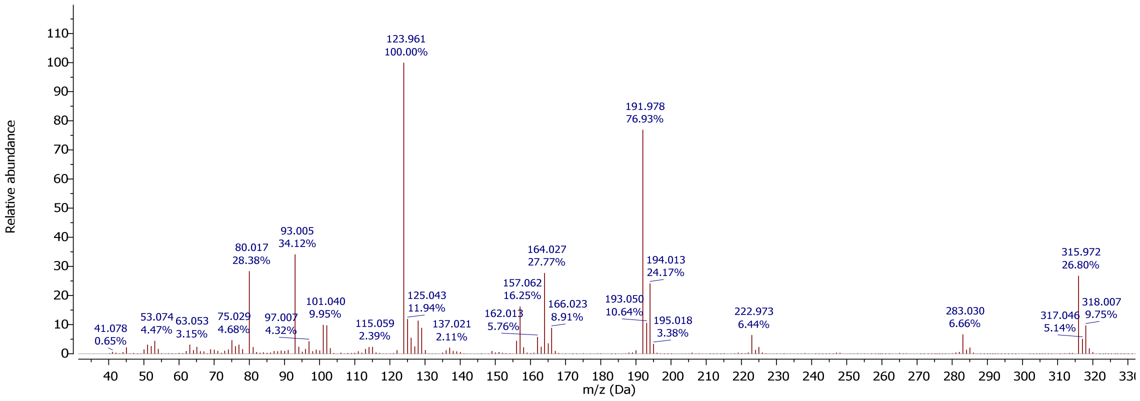

ESI-QTOF (positive ionization)

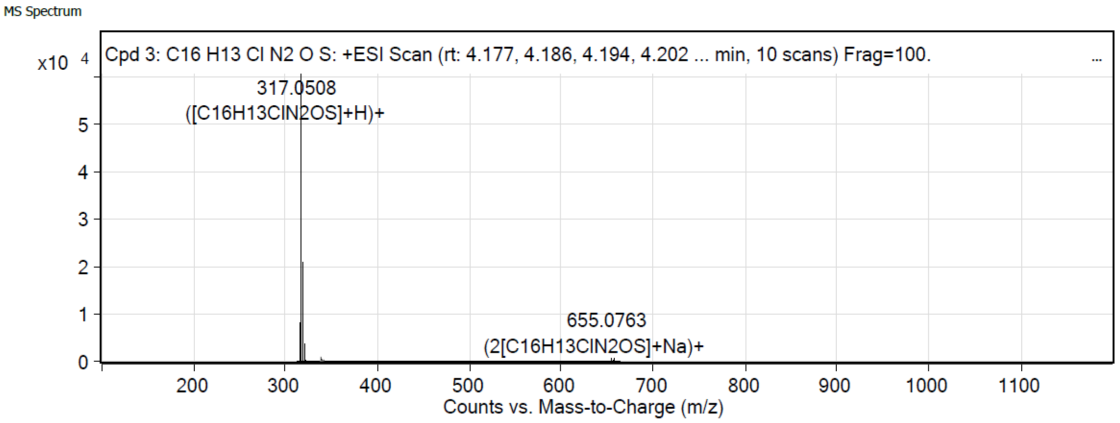

MS Spectrum Peak List

m/z	Calc *m/z*	Diff(ppm)	z	Abund	Formula	Ion
317.0508	317.051	0.45	1	60594.69	C16H13ClN2OS	(M+H)+
318.0519	318.054	6.5	1	11698.33	C16H13ClN2OS	(M+H)+
319.048	319.0483	1.08	1	21302.87	C16H13ClN2OS	(M+H)+
320.0507	320.051	1.13	1	3894.05	C16H13ClN2OS	(M+H)+
321.0456	321.0471	4.49	1	1189.33	C16H13ClN2OS	(M+H)+
339.0321	339.0329	2.45	1	937.9	C16H13ClN2OS	(M+Na)+
341.0301	341.0303	0.58	1	342.45	C16H13ClN2OS	(M+Na)+
655.0763	655.0766	0.55	1	663.34	C16H13ClN2OS	(2M+Na)+
657.0749	657.0744	-0.84	1	508.51	C16H13ClN2OS	(2M+Na)+
658.0775	658.0768	-1.07	1	251.62	C16H13ClN2OS	(2M+Na)+

4.10 Compound 23c

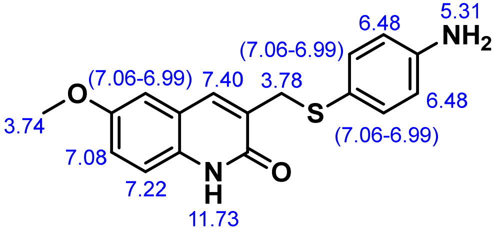

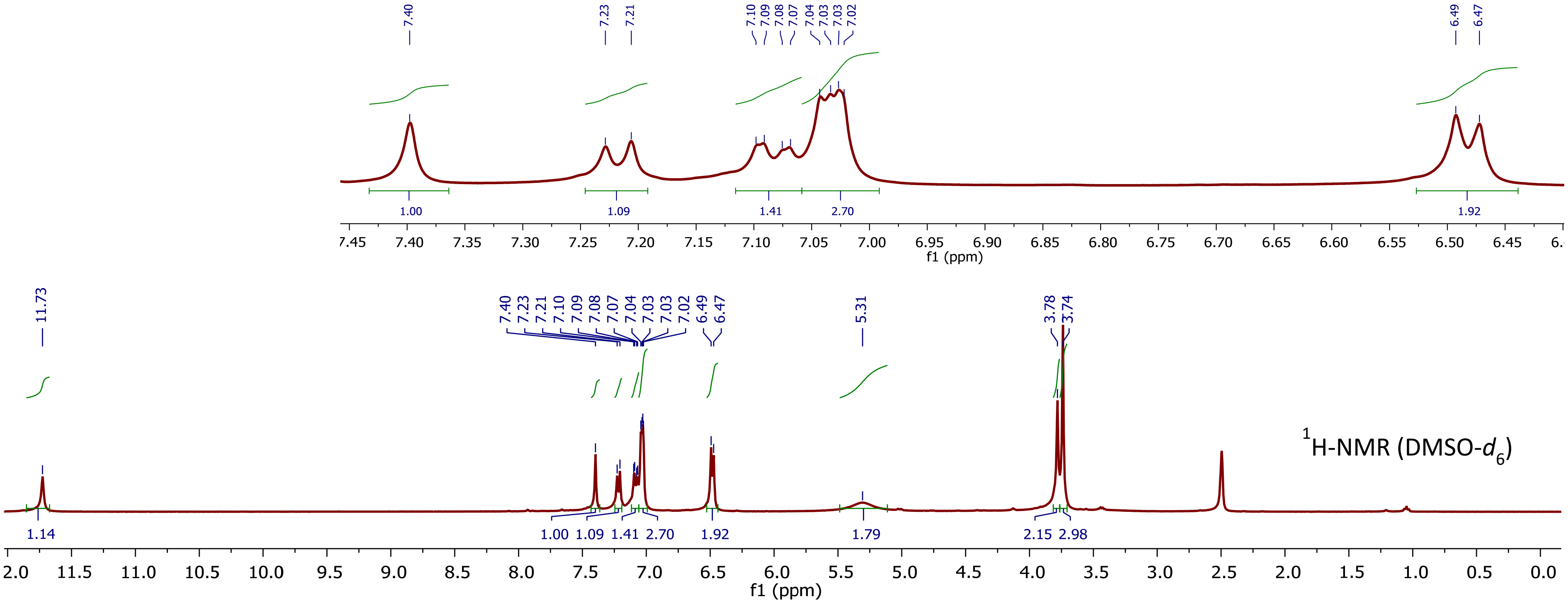

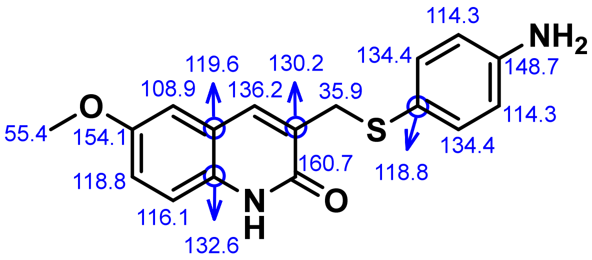
NH2
O
S
N
H
O
55.4
154.1
118.8
116.1
132.6
108.9
119.6
136.2
130.2
160.7
35.9
134.4
114.3
148.7
114.3
134.4
118.8

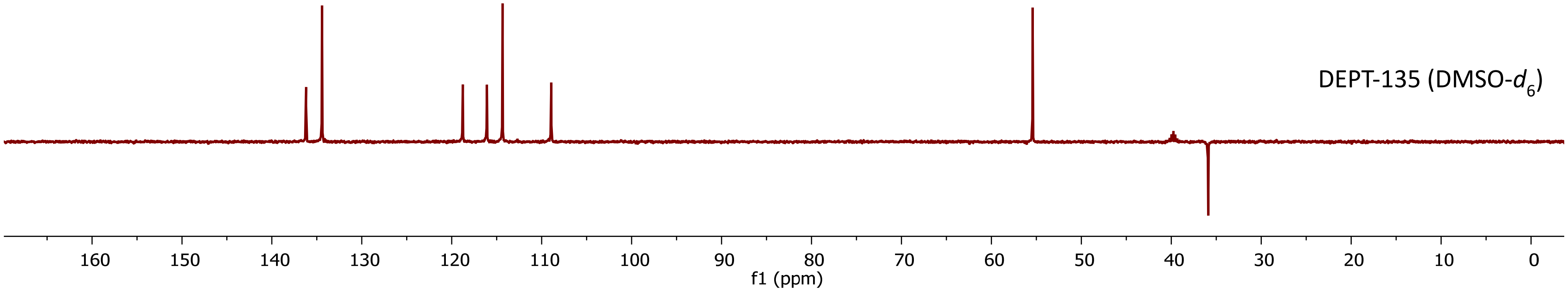
DEPT-135 (DMSO-d6)
160
150
140
130
120
110
100
90
80
70
60
50
40
30
20
10
0
f1 (ppm)

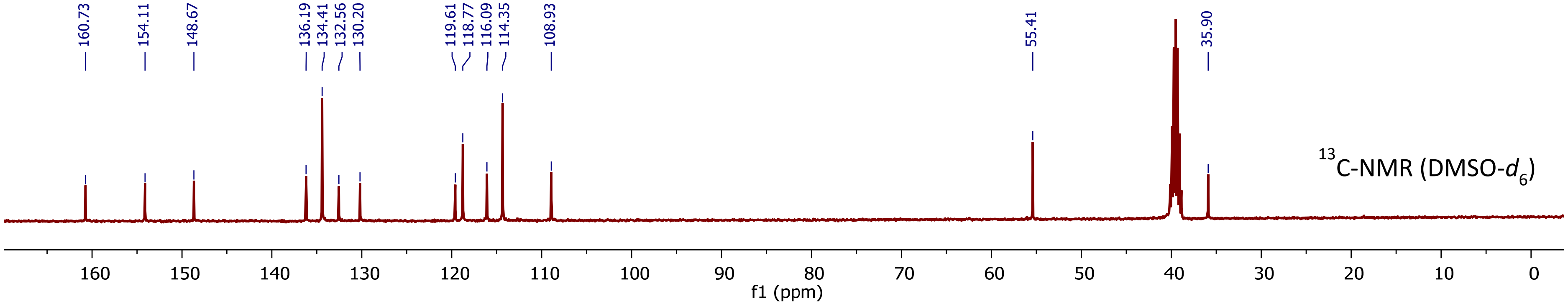
160.73
154.11
148.67
136.19
134.41
132.56
130.20
119.61
118.77
116.09
114.35
108.93
55.41
35.90
13C-NMR (DMSO-d6)
160
150
140
130
120
110
100
90
80
70
60
50
40
30
20
10
0
f1 (ppm)

EI MS (70eV)

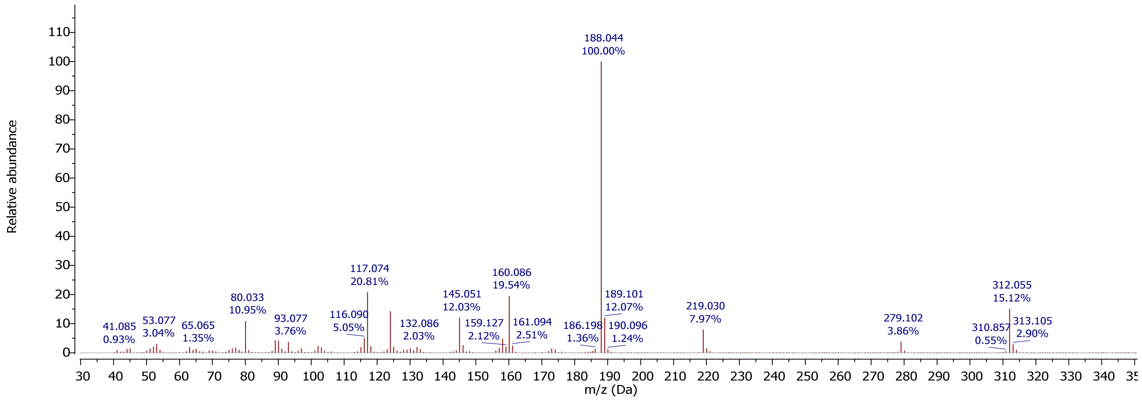

ESI-QTOF (positive ionization)

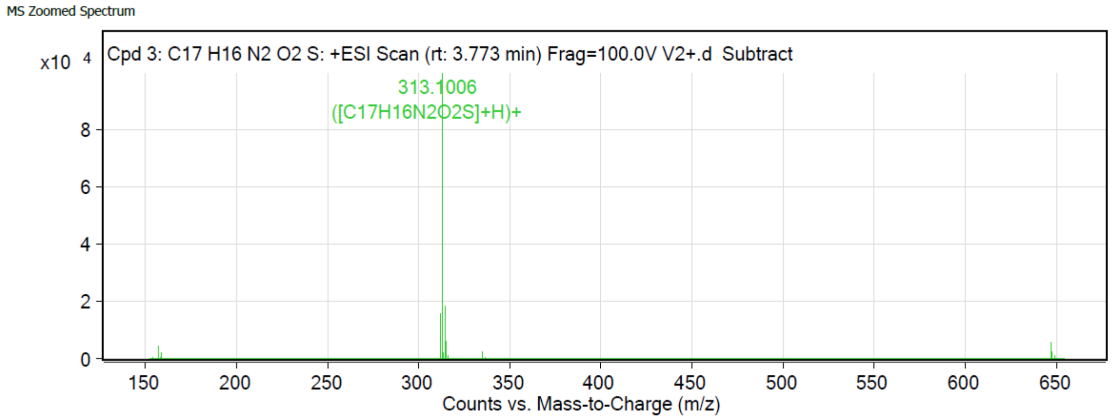

MS Spectrum Peak List

m/z	Calc *m/z*	Diff(ppm)	z	Abund	Formula	Ion
157.0524	157.0539	9.51	2	4559.77	C17H16N2O2S	(M+2H)+2
157.5549	157.5554	3.06	2	765.06	C17H16N2O2S	(M+2H)+2
313.1006	313.1005	-0.33	1	102166.45	C17H16N2O2S	(M+H)+
314.1042	314.1035	-2	1	18677.32	C17H16N2O2S	(M+H)+
315.0997	315.0997	-0.02	1	6155.71	C17H16N2O2S	(M+H)+
335.0829	335.0825	-1.31	1	2635.22	C17H16N2O2S	(M+Na)+
336.0885	336.0855	-9.12	1	518.64	C17H16N2O2S	(M+Na)+
647.177	647.1757	-2.05	1	5555	C17H16N2O2S	(2M+Na)+
648.1801	648.1787	-2.06	1	2482.27	C17H16N2O2S	(2M+Na)+
649.1823	649.1765	-8.92	1	842.61	C17H16N2O2S	(2M+Na)+

4.11 Compound 24a

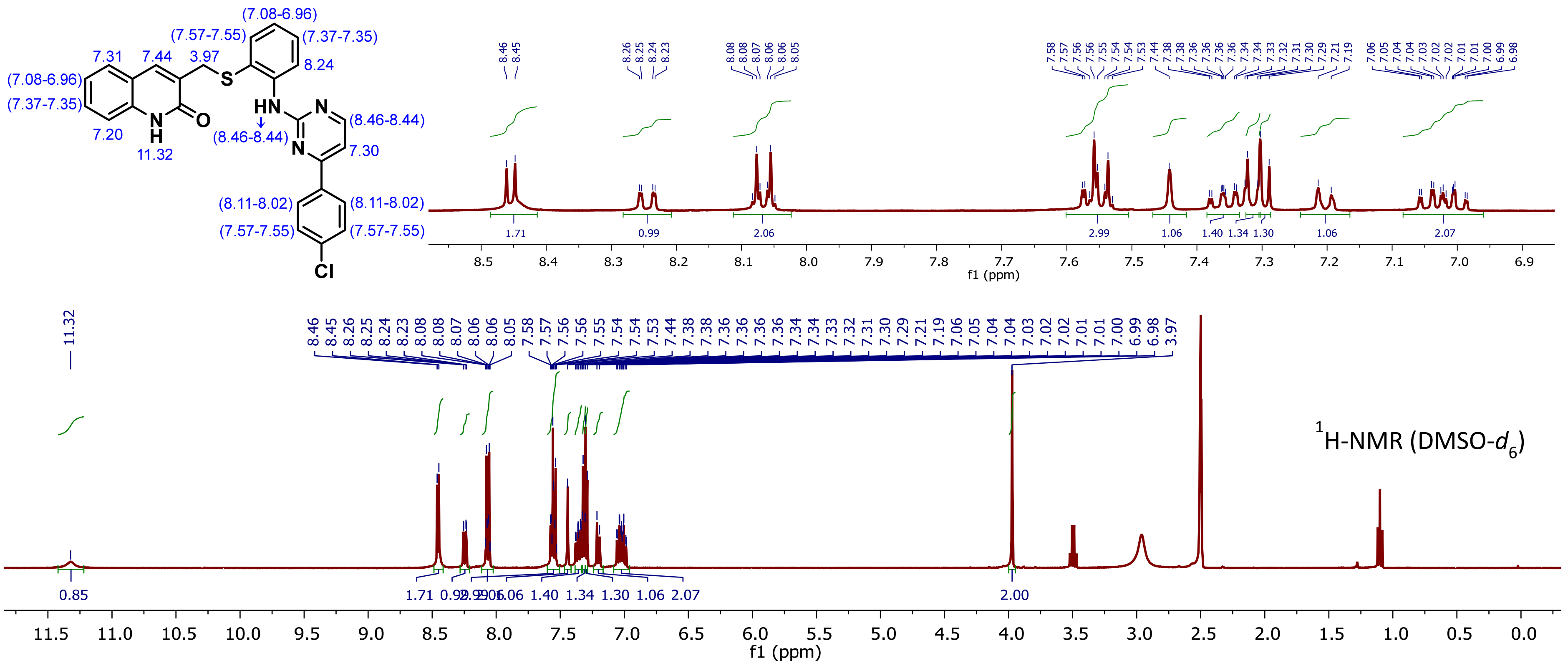

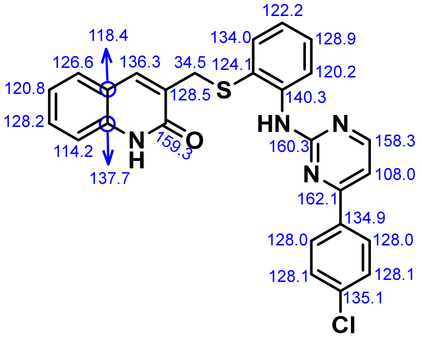
118.4
126.6
136.3
34.5
124.1
134.0
122.2
128.9
120.2
140.3
120.8
128.5
S
128.2
HN
N
158.3
160.3
114.2
N
H
O
159.3
137.7
N
108.0
162.1
134.9
128.0
128.0
128.1
128.1
135.1
Cl

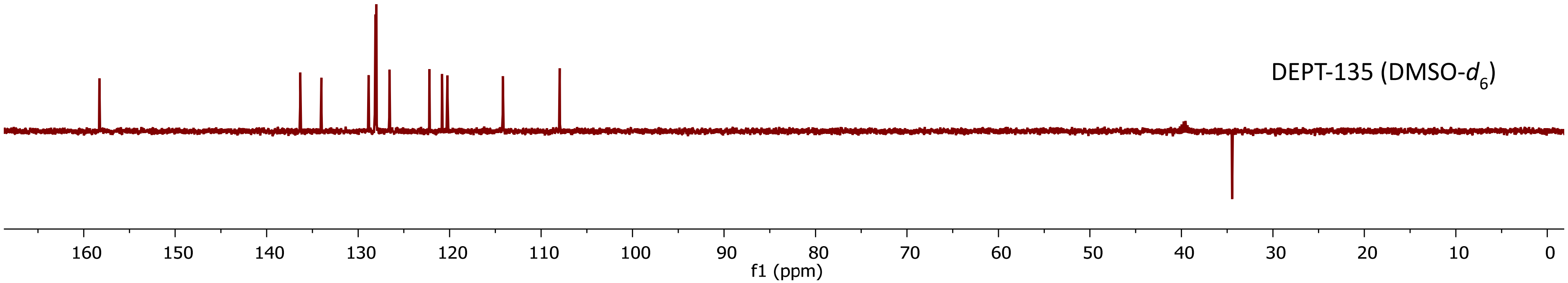
DEPT-135 (DMSO-d_6)
160
150
140
130
120
110
100
90
80
70
60
50
40
30
20
10
0
f1 (ppm)

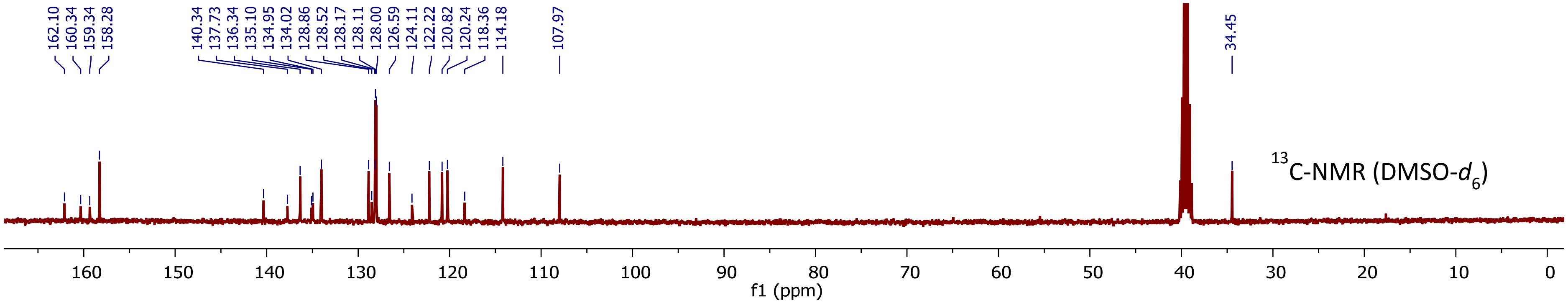
162.10
160.34
159.34
158.28
140.34
137.73
136.34
135.10
134.95
134.02
128.86
128.52
128.17
128.11
128.00
126.59
124.11
122.22
120.82
120.24
118.36
114.18
107.97
34.45
^{13}C-NMR (DMSO-d_6)
160
150
140
130
120
110
100
90
80
70
60
50
40
30
20
10
0
f1 (ppm)

EI MS (70eV)

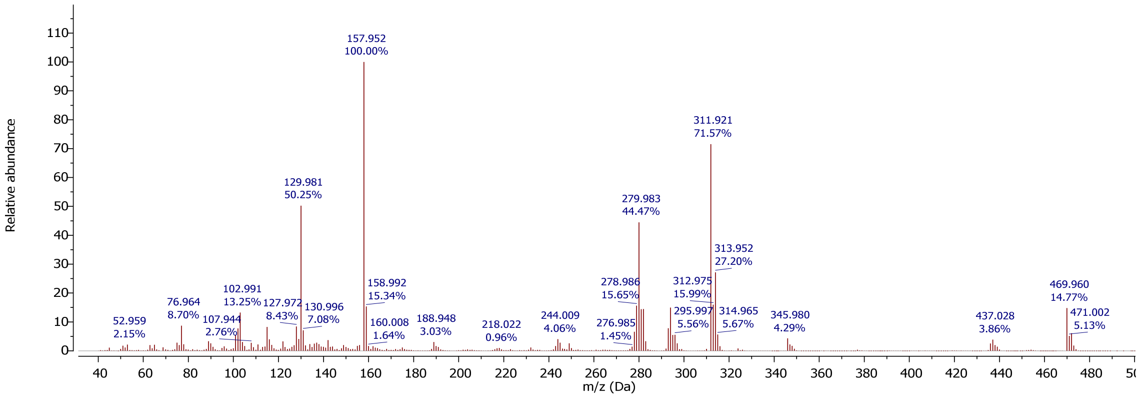

ESI-QTOF (positive ionization)

MS Spectrum

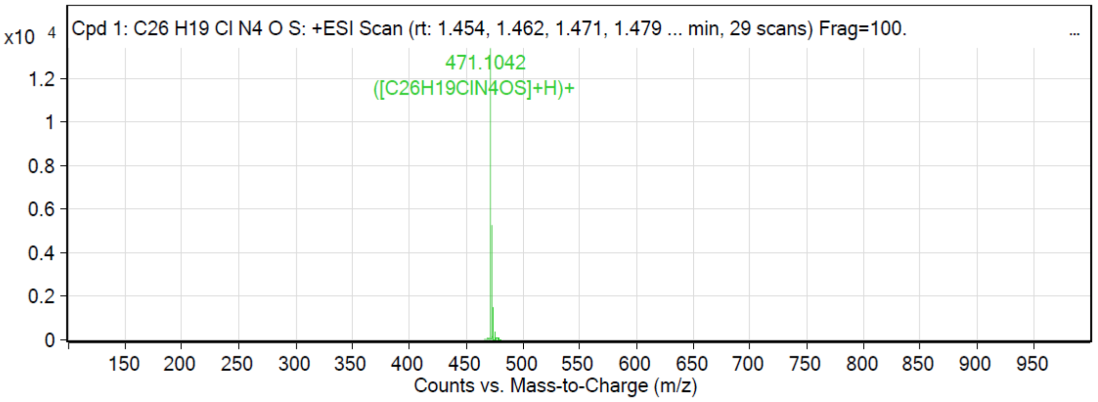

MS Spectrum Peak List

m/z	Calc *m/z*	Diff(ppm)	z	Abund	Formula	Ion
471.1042	471.1041	-0.14	1	13681.64	C26H19ClN4OS	(M+H)+
472.1075	472.1071	-0.84	1	3982.72	C26H19ClN4OS	(M+H)+
473.1018	473.102	0.35	1	5511.6	C26H19ClN4OS	(M+H)+
474.1039	474.1043	0.85	1	1595.88	C26H19ClN4OS	(M+H)+
475.1025	475.1025	-0.09	1	360.43	C26H19ClN4OS	(M+H)+
476.1051	476.1029	-4.52	1	47.53	C26H19ClN4OS	(M+H)+

4.12 Compound 24b

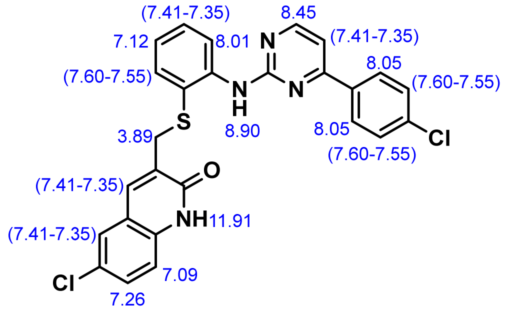

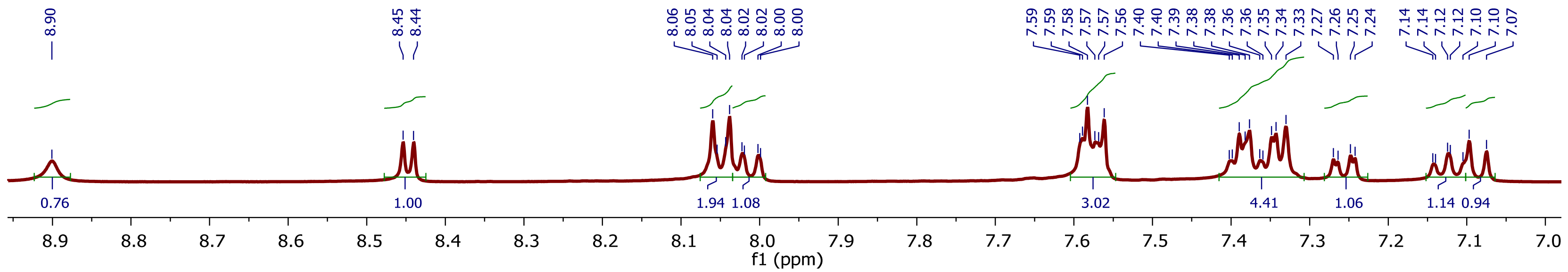

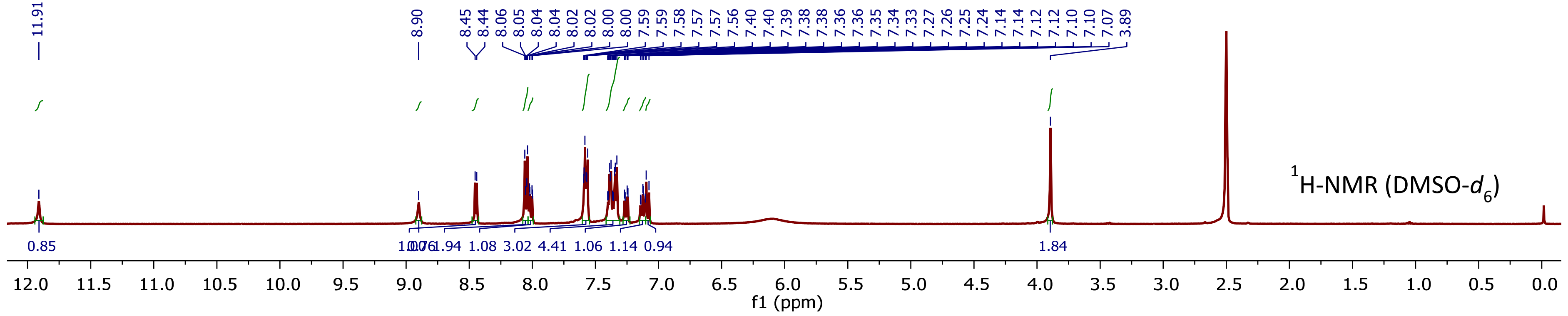

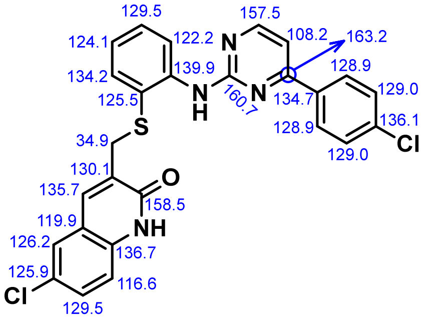
129.5
124.1
122.2
134.2
139.9
125.5
157.5
108.2
163.2
128.9
129.0
160.7
134.7
136.1
128.9
129.0
Cl
N
N
NH
S
34.9
130.1
135.7
O
158.5
119.9
NH
126.2
136.7
125.9
116.6
Cl
129.5

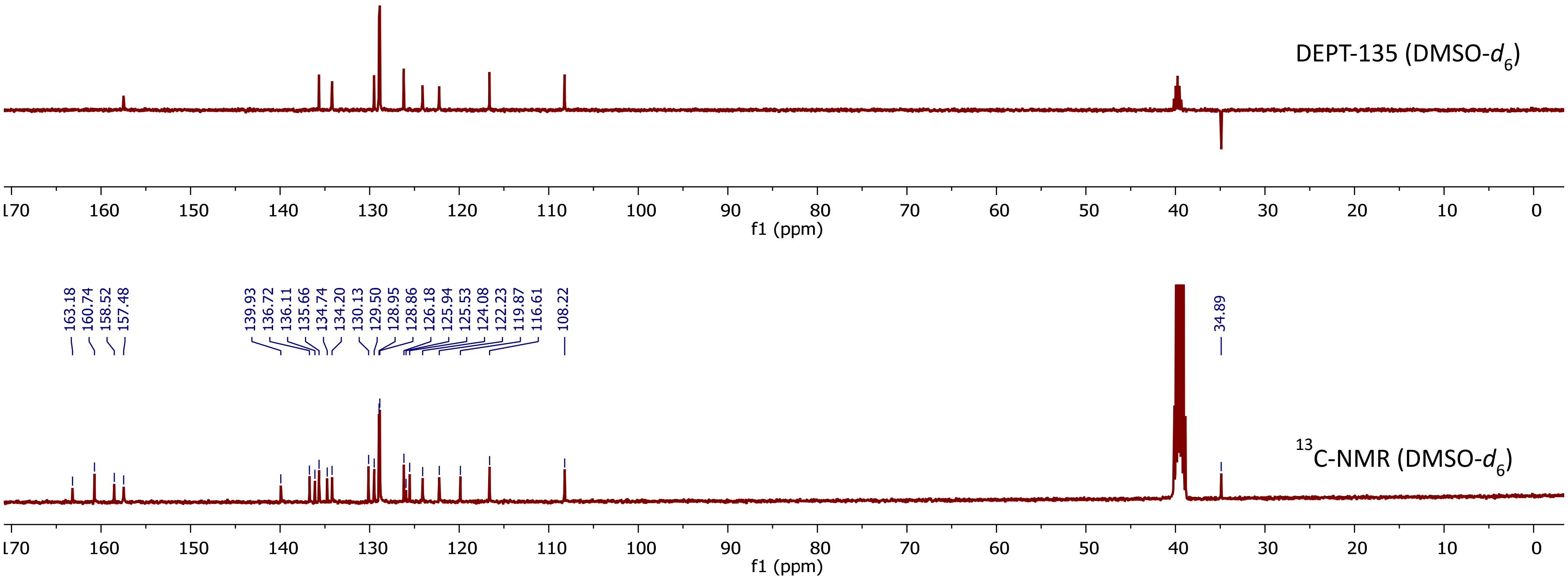
DEPT-135 (DMSO-d6)
f1 (ppm)
163.18
160.74
158.52
157.48
139.93
136.72
136.11
135.66
134.74
134.20
130.13
129.50
128.95
128.86
126.18
125.94
125.53
124.08
122.23
119.87
116.61
108.22
34.89
13C-NMR (DMSO-d6)
f1 (ppm)

EI MS (70eV)

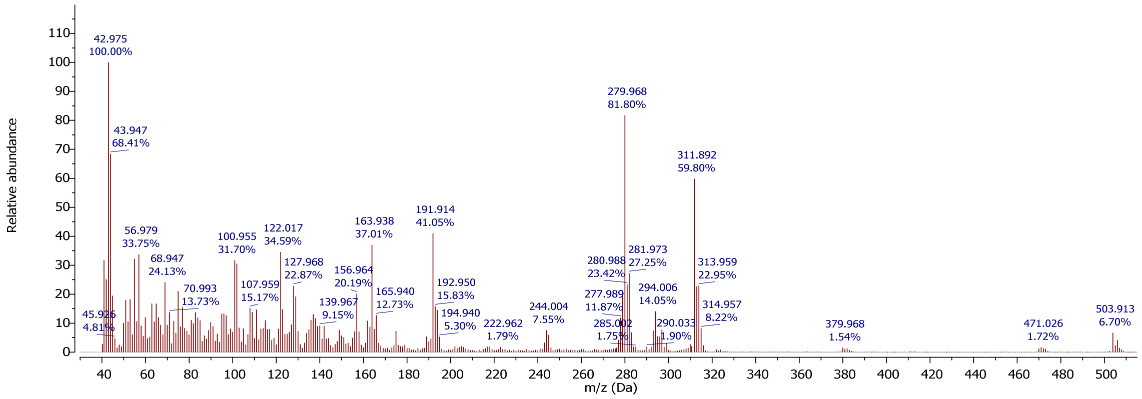

ESI-QTOF (positive ionization)

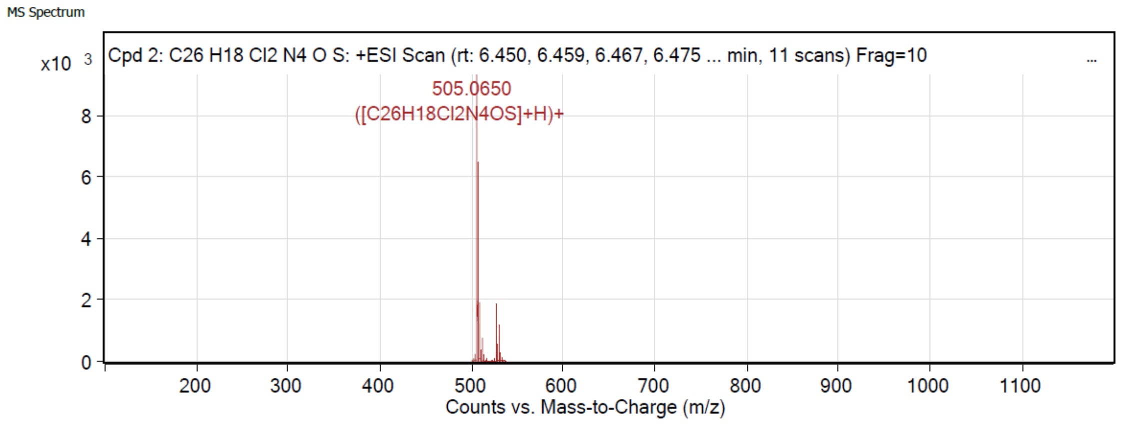

MS Spectrum Peak List

m/z	Calc *m/z*	Diff(ppm)	z	Abund	Formula	Ion
505.065	505.0651	0.24	1	9495.01	C26H18Cl2N4OS	(M+H)+
506.0685	506.0681	-0.87	1	3037.56	C26H18Cl2N4OS	(M+H)+
507.0624	507.0627	0.55	1	6658.38	C26H18Cl2N4OS	(M+H)+
508.0651	508.0653	0.41	1	1980.76	C26H18Cl2N4OS	(M+H)+
509.0605	509.0608	0.52	1	1507.94	C26H18Cl2N4OS	(M+H)+
510.0626	510.0626	0.07	1	423.29	C26H18Cl2N4OS	(M+H)+
527.0473	527.0471	-0.55	1	1897.1	C26H18Cl2N4OS	(M+Na)+
528.0515	528.05	-2.84	1	538.21	C26H18Cl2N4OS	(M+Na)+
529.0448	529.0446	-0.36	1	1290.64	C26H18Cl2N4OS	(M+Na)+
530.0491	530.0472	-3.54	1	371.94	C26H18Cl2N4OS	(M+Na)+

4.13 Compound 24c

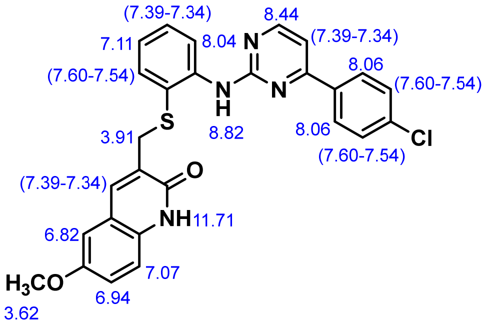

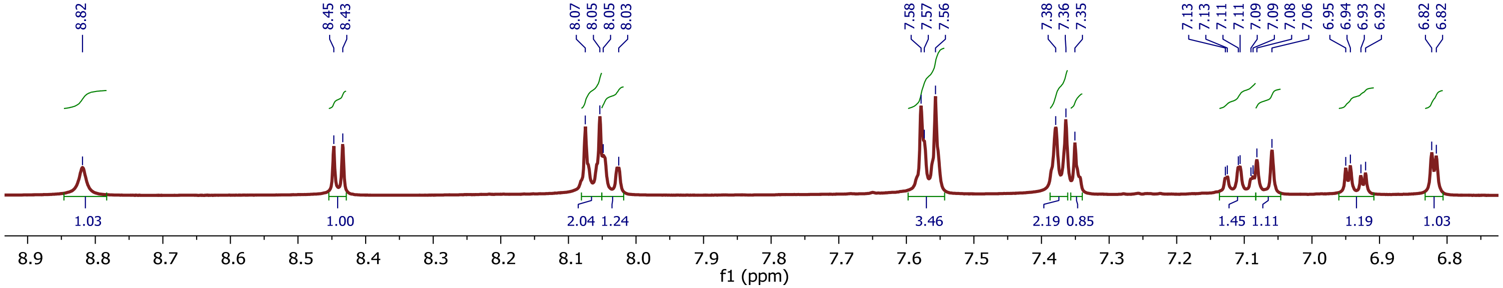

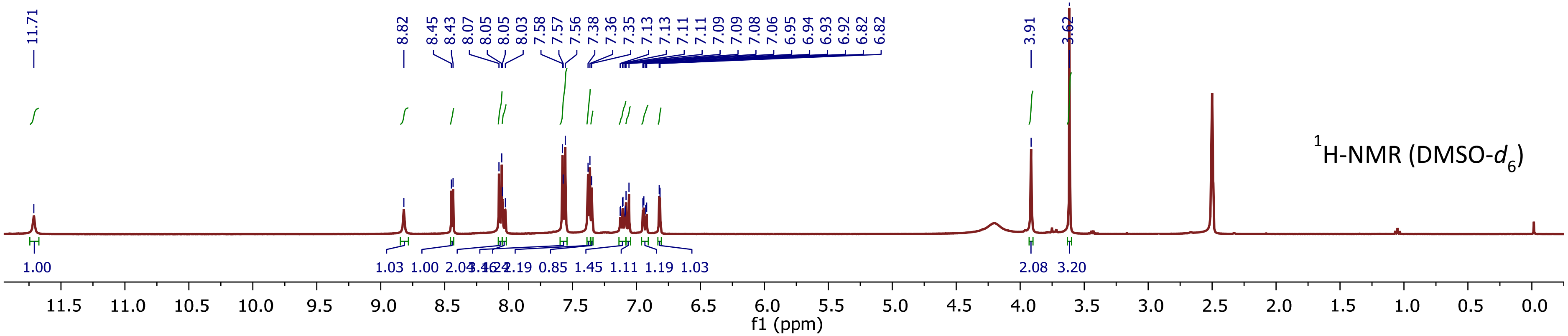

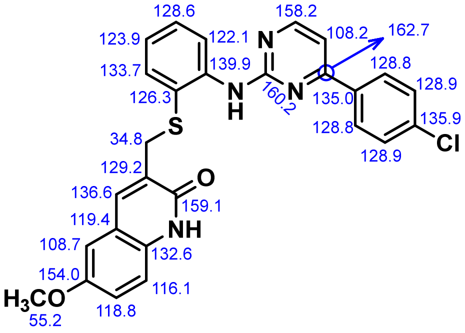

128.6
123.9
133.7
126.3
122.1
139.9
158.2
108.2
162.7
128.8
128.9
135.0
160.2
135.9
128.8
128.9
N
N
N
H
S
Cl
34.8
129.2
O
136.6
159.1
119.4
NH
108.7
132.6
154.0
116.1
H3CO
118.8
55.2

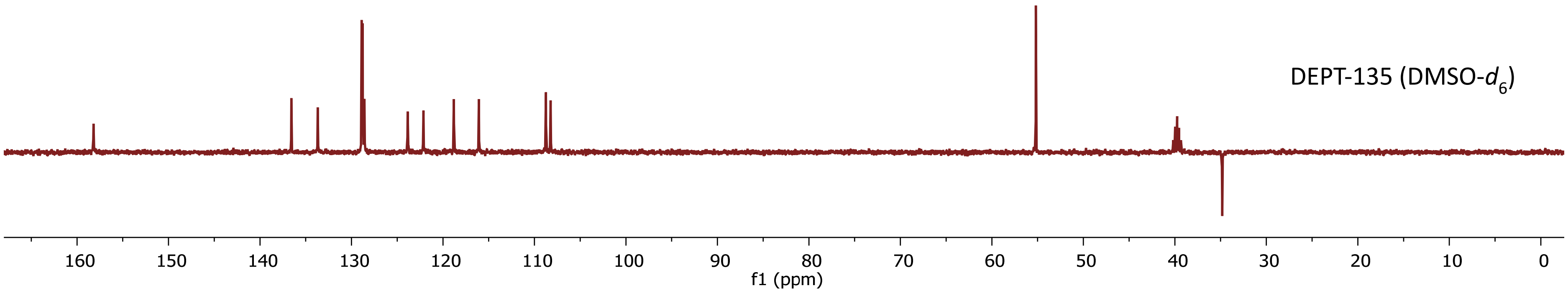

DEPT-135 (DMSO-d6)
160
150
140
130
120
110
100
90
80
70
60
50
40
30
20
10
0
f1 (ppm)

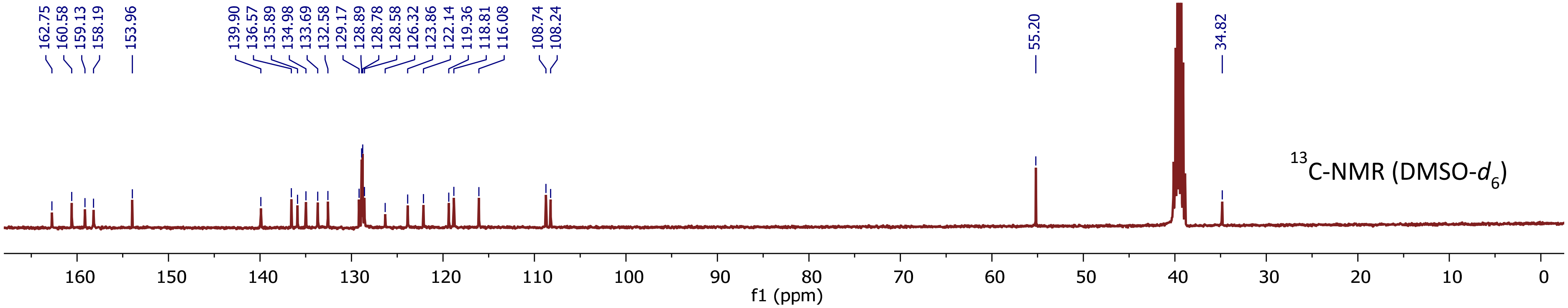

162.75
160.58
159.13
158.19
153.96
139.90
136.57
135.89
134.98
133.69
132.58
129.17
128.89
128.78
128.58
126.32
123.86
122.14
119.36
118.81
116.08
108.74
108.24
55.20
34.82
13C-NMR (DMSO-d6)
160
150
140
130
120
110
100
90
80
70
60
50
40
30
20
10
0
f1 (ppm)

EI MS (70eV)

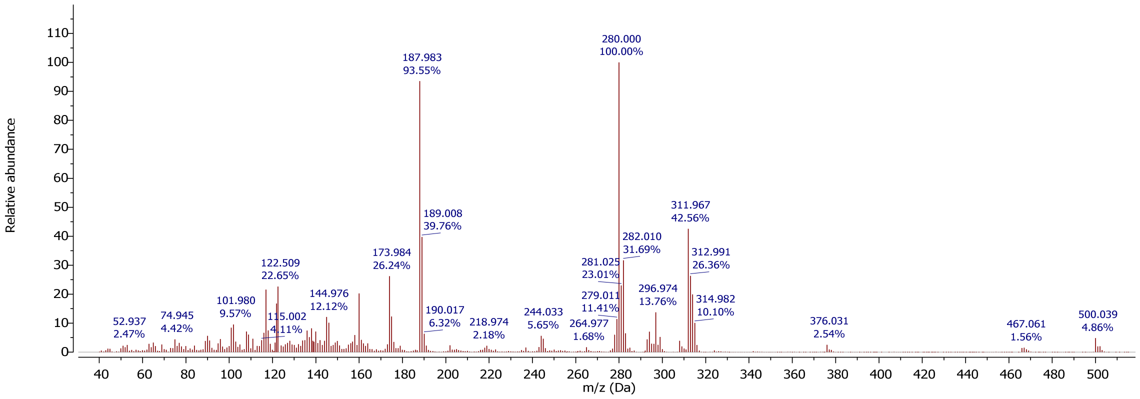

ESI-QTOF (positive ionization)

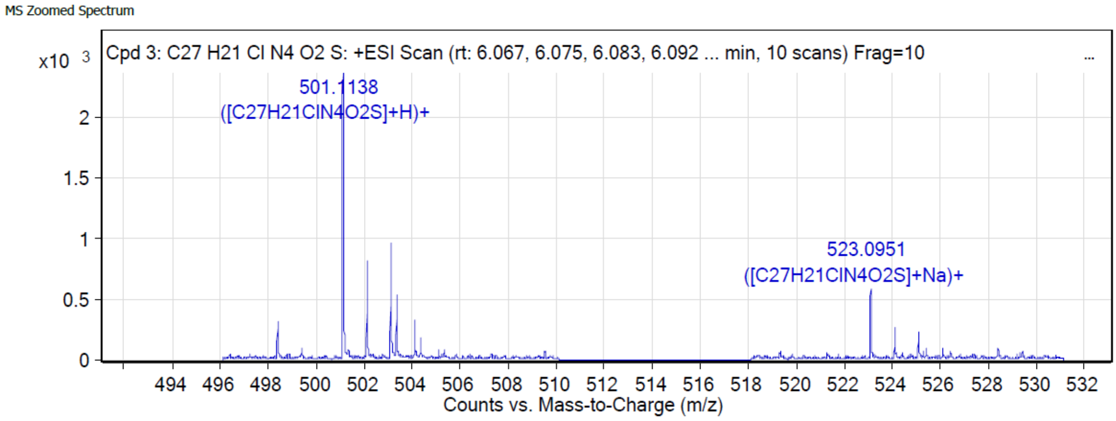

MS Spectrum Peak List

m/z	Calc *m/z*	Diff(ppm)	z	Abund	Formula	Ion
501.1138	501.1147	1.65	1	2387.34	C27H21ClN4O2S	(M+H)+
502.1158	502.1176	3.64	1	821.95	C27H21ClN4O2S	(M+H)+
503.1118	503.1127	1.77	1	1048.47	C27H21ClN4O2S	(M+H)+
504.1132	504.115	3.65	1	374.25	C27H21ClN4O2S	(M+H)+
505.1054	505.1134	15.72	1	25.44	C27H21ClN4O2S	(M+H)+
523.0951	523.0966	2.88	1	604.97	C27H21ClN4O2S	(M+Na)+
524.099	524.0996	1.06	1	291.17	C27H21ClN4O2S	(M+Na)+
525.0945	525.0946	0.31	1	265.23	C27H21ClN4O2S	(M+Na)+
526.0955	526.0969	2.63	1	46.9	C27H21ClN4O2S	(M+Na)+

4.14 Compound 25a

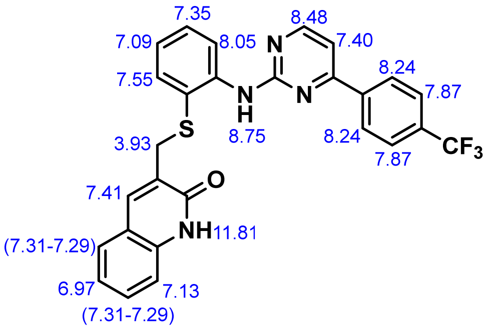

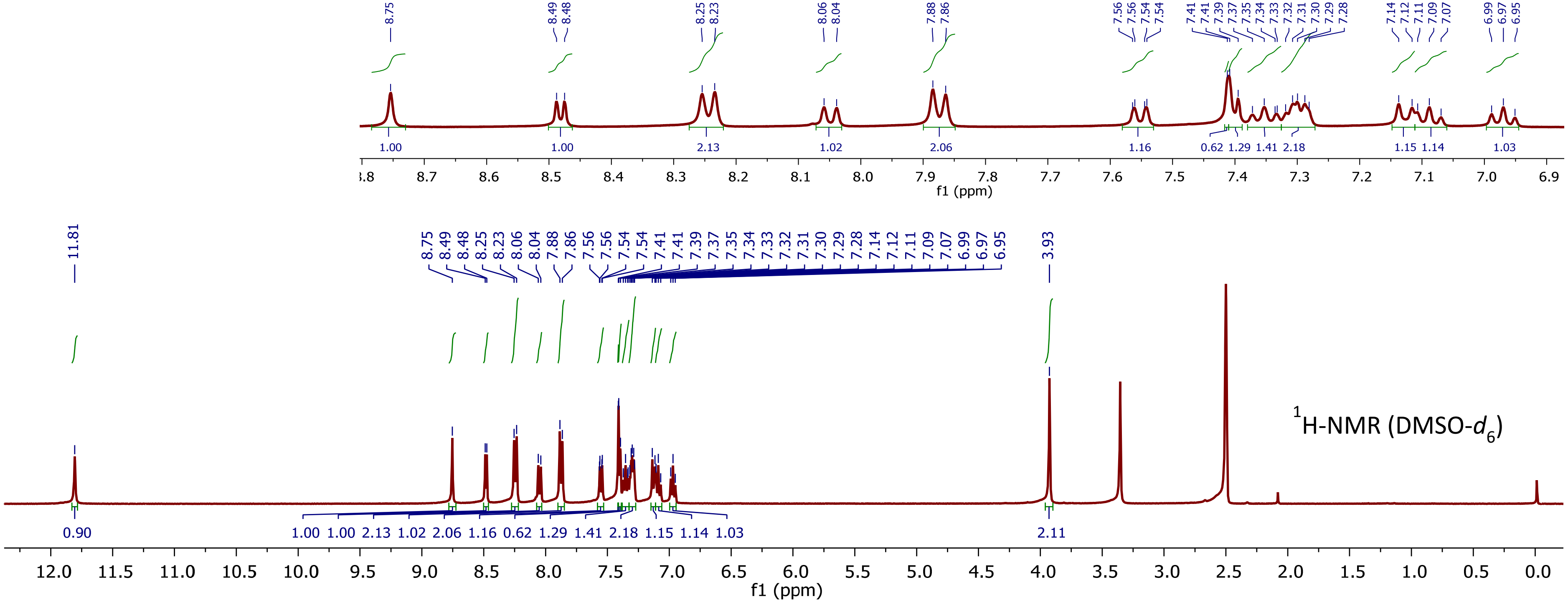

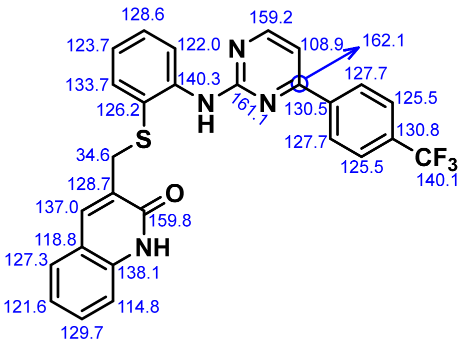
128.6
159.2
123.7
122.0
N
108.9
162.1
127.7
133.7
140.3
125.5
126.2
N
H
161.7
N
130.5
130.8
S
127.7
CF3
34.6
125.5
140.1
128.7
O
137.0
159.8
118.8
NH
127.3
138.1
121.6
114.8
129.7

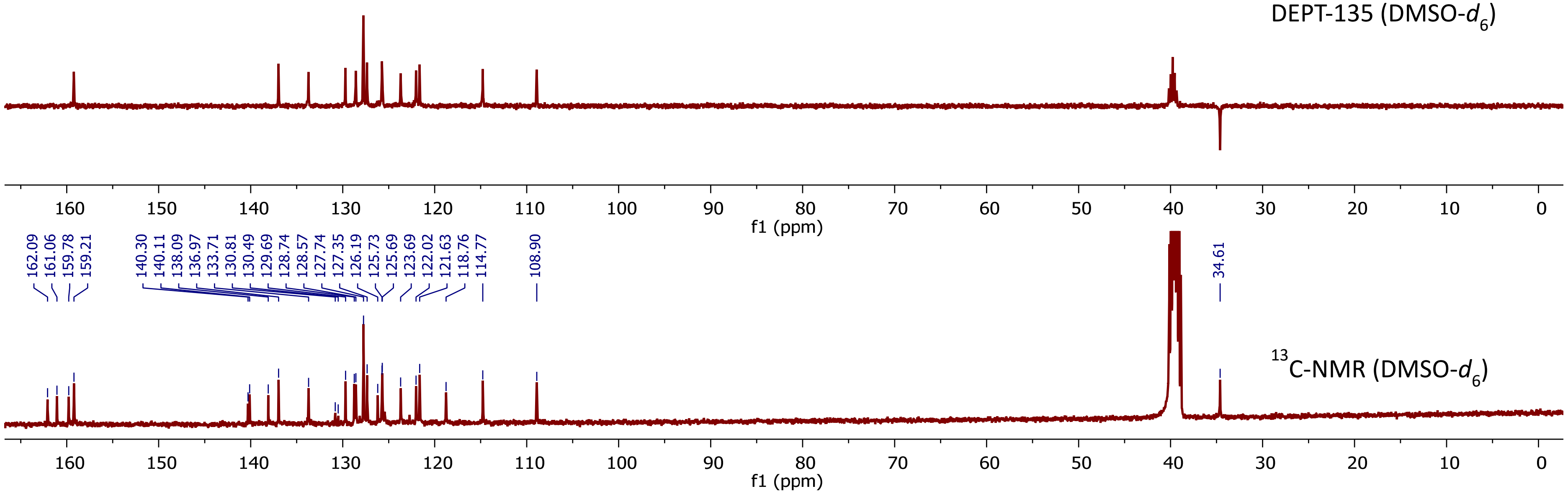
DEPT-135 (DMSO-d6)
160
150
140
130
120
110
100
90
80
70
60
50
40
30
20
10
0
f1 (ppm)
162.09
161.06
159.78
159.21
140.30
140.11
138.09
136.97
133.71
130.81
130.49
129.69
128.74
128.57
127.74
127.35
126.19
125.73
125.69
123.69
122.02
121.63
118.76
114.77
108.90
34.61
13C-NMR (DMSO-d6)
160
150
140
130
120
110
100
90
80
70
60
50
40
30
20
10
0
f1 (ppm)

EI MS (70eV)

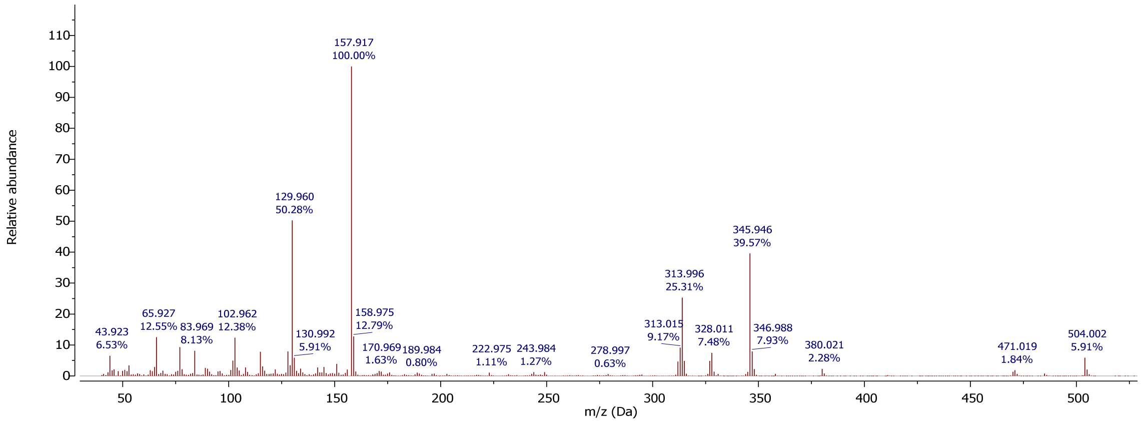

ESI-QTOF (positive ionization)

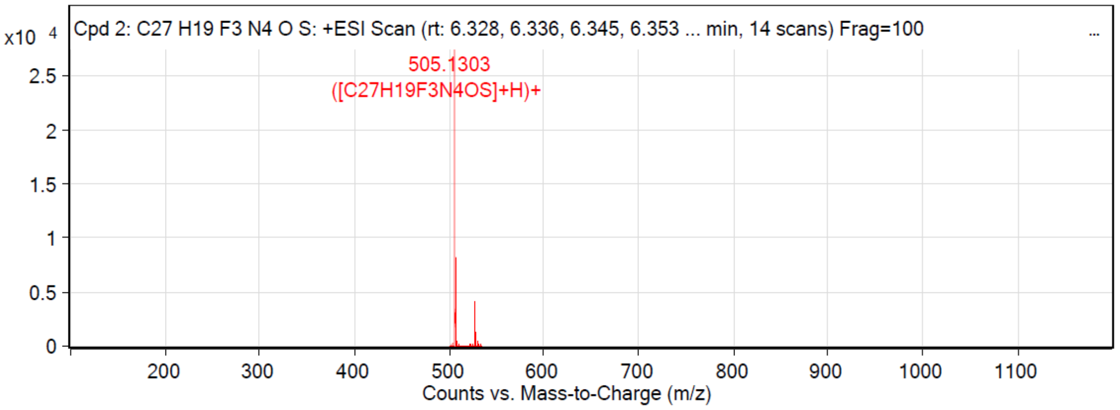

MS Spectrum Peak List

m/z	Calc *m/z*	Diff(ppm)	z	Abund	Formula	Ion
505.1303	505.1304	0.2	1	27882.64	C27H19F3N4OS	(M+H)+
506.1333	506.1334	0.34	1	8461.88	C27H19F3N4OS	(M+H)+
507.1315	507.1316	0.29	1	2272.03	C27H19F3N4OS	(M+H)+
527.1121	527.1124	0.55	1	4178.53	C27H19F3N4OS	(M+Na)+
528.1155	528.1154	-0.25	1	1381.58	C27H19F3N4OS	(M+Na)+
529.1131	529.1135	0.86	1	367.8	C27H19F3N4OS	(M+Na)+

4.15 Compound 25b

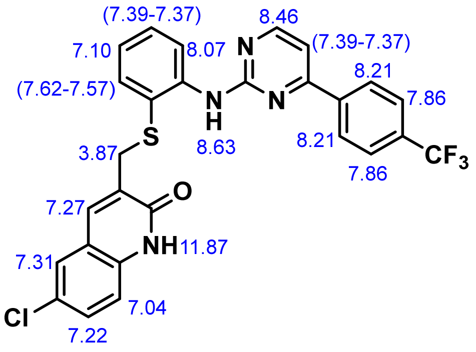

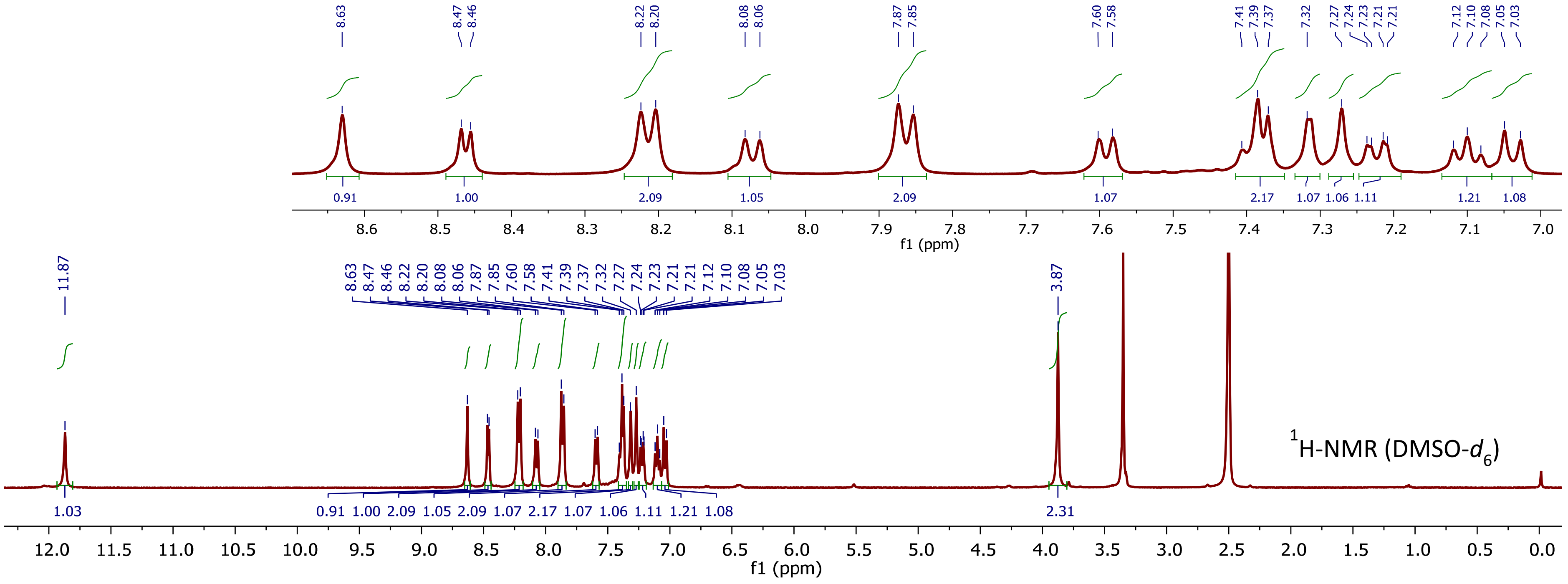

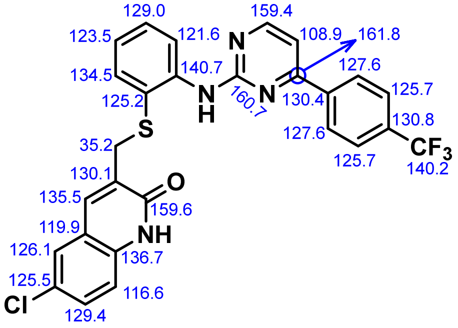
129.0
123.5
121.6
159.4
108.9
161.8
140.7
134.5
127.6
125.7
125.2
160.7
130.4
130.8
CF3
35.2
127.6
125.7
140.2
130.1
O
135.5
159.6
119.9
NH
126.1
136.7
125.5
Cl
116.6
129.4
S
N
H

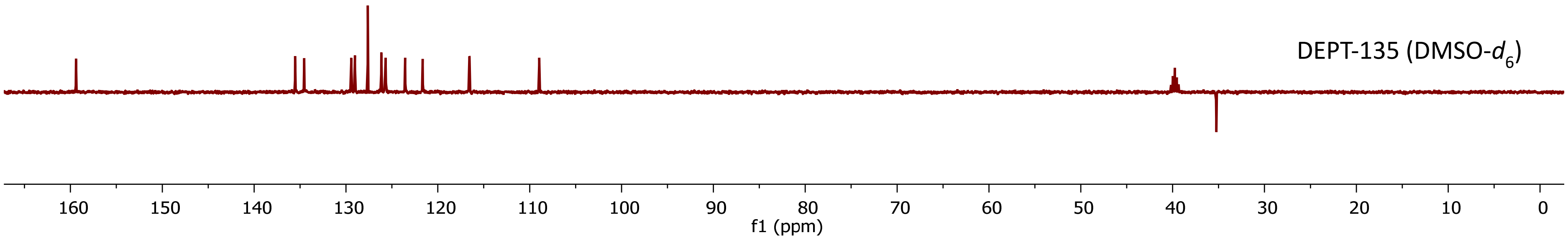
DEPT-135 (DMSO-d6)
160
150
140
130
120
110
100
90
80
70
60
50
40
30
20
10
0
f1 (ppm)

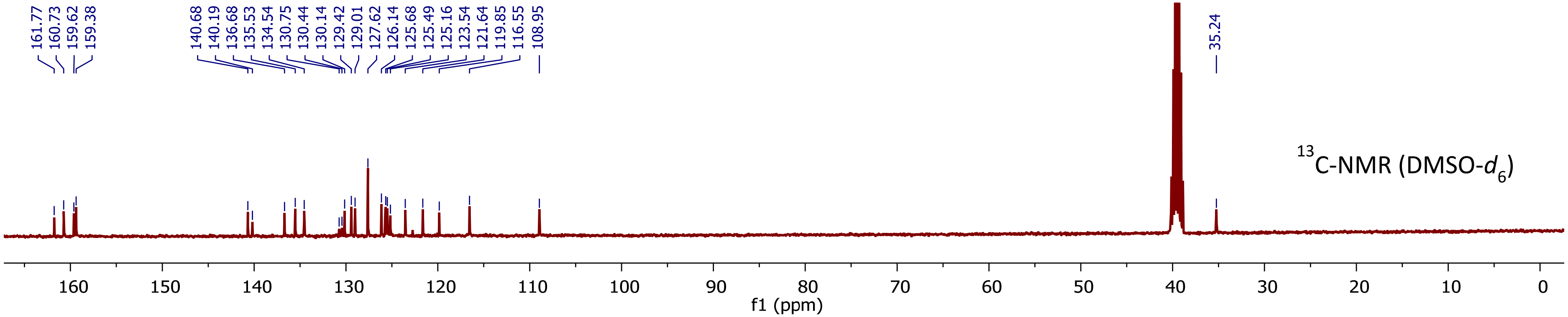
161.77
160.73
159.62
159.38
140.68
140.19
136.68
135.53
134.54
130.75
130.44
130.14
129.42
129.01
127.62
126.14
125.68
125.49
125.16
123.54
121.64
119.85
116.55
108.95
35.24
13C-NMR (DMSO-d6)
160
150
140
130
120
110
100
90
80
70
60
50
40
30
20
10
0
f1 (ppm)

EI MS (70eV)

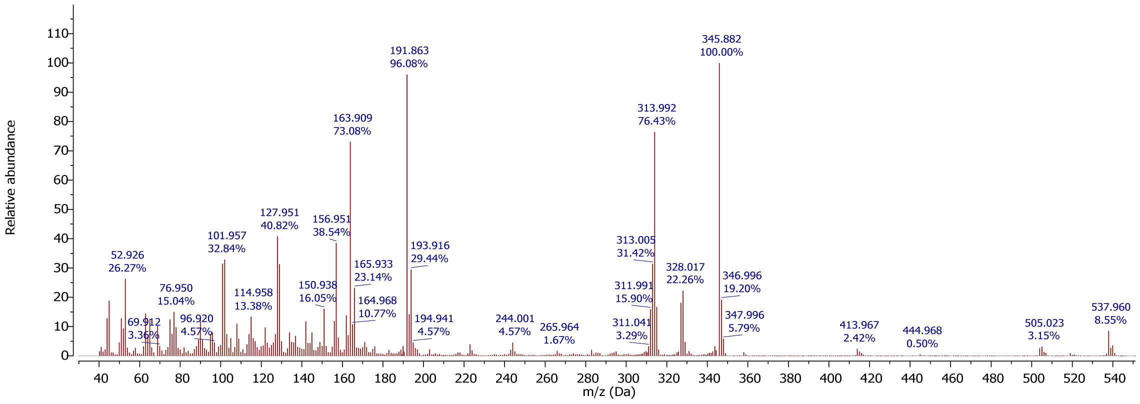

ESI-QTOF (positive ionization)

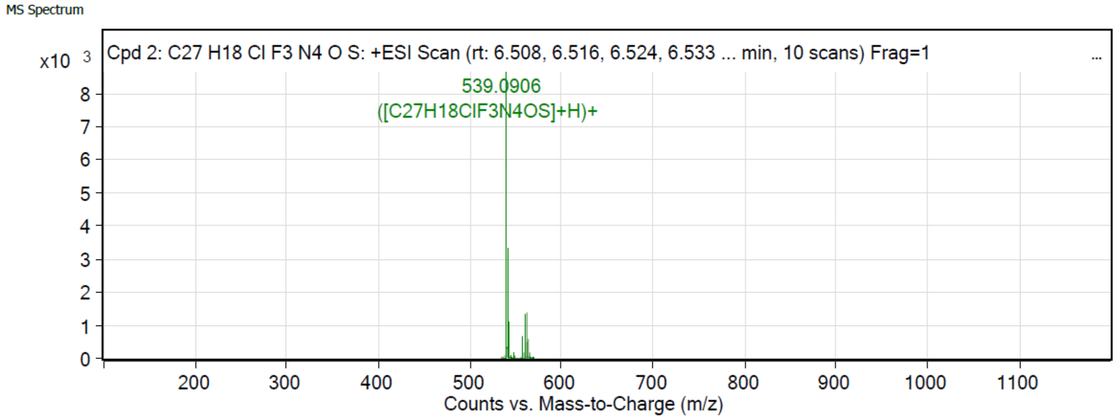

MS Spectrum Peak List

m/z	Calc *m/z*	Diff(ppm)	z	Abund	Formula	Ion
539.0906	539.0915	1.56	1	8896.48	C27H18ClF3N4OS	(M+H)+
540.093	540.0945	2.7	1	2820.27	C27H18ClF3N4OS	(M+H)+
541.0885	541.0895	1.72	1	3407.42	C27H18ClF3N4OS	(M+H)+
542.0903	542.0918	2.72	1	1135.08	C27H18ClF3N4OS	(M+H)+
543.0903	543.0901	-0.48	1	221.11	C27H18ClF3N4OS	(M+H)+
561.0729	561.0734	0.87	1	1409.61	C27H18ClF3N4OS	(M+Na)+
562.0749	562.0764	2.71	1	497.96	C27H18ClF3N4OS	(M+Na)+
563.071	563.0714	0.77	1	598.91	C27H18ClF3N4OS	(M+Na)+
564.0688	564.0737	8.76	1	130.54	C27H18ClF3N4OS	(M+Na)+
565.0657	565.072	11.26	1	21.71	C27H18ClF3N4OS	(M+Na)+

4.16 Compound 25c

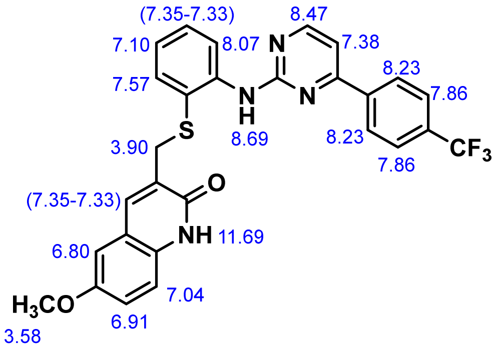

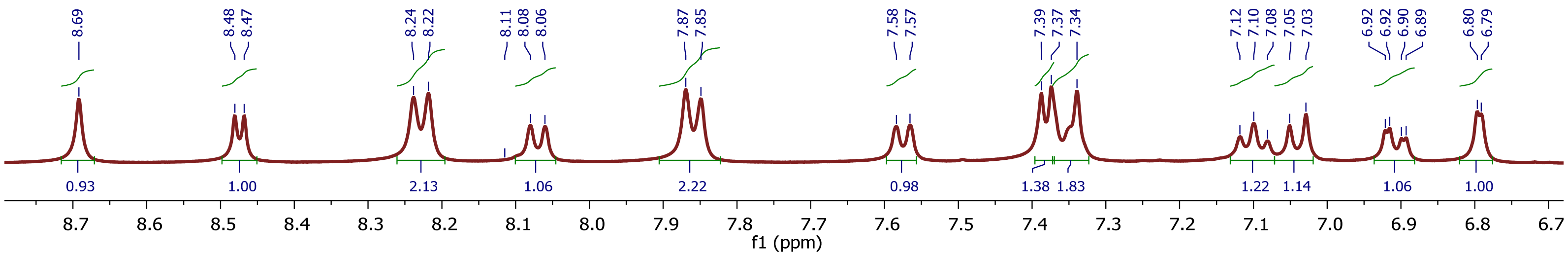

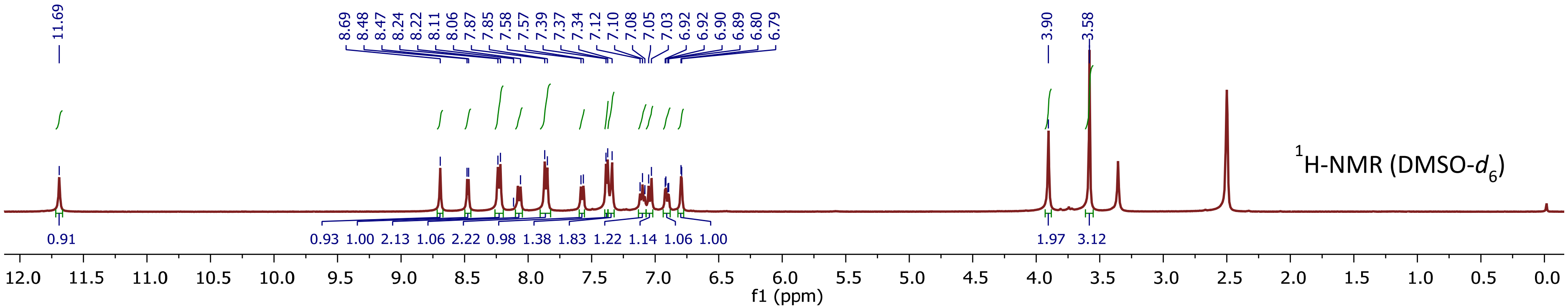

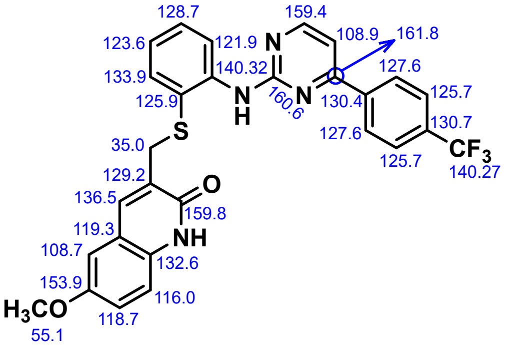

128.7
159.4
123.6
121.9
108.9
161.8
140.32
127.6
133.9
125.7
125.9
160.6
130.4
130.7
N
N
N
H
S
35.0
127.6
CF_3
125.7
140.27
129.2
O
136.5
159.8
119.3
NH
108.7
132.6
153.9
116.0
H_3CO
118.7
55.1

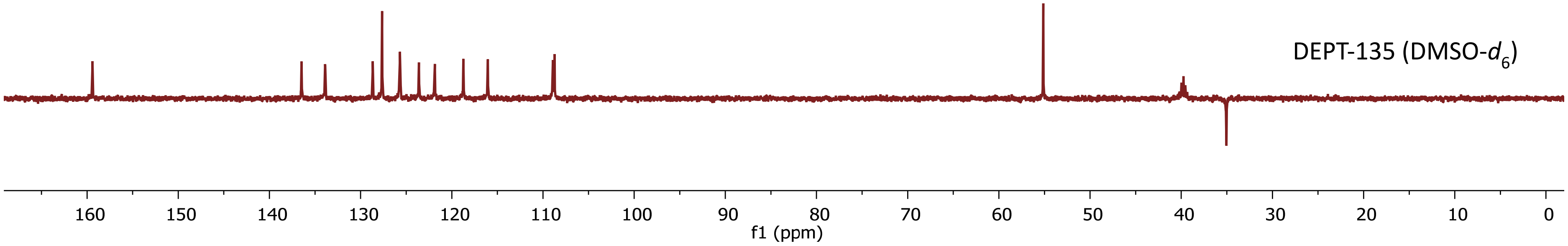

DEPT-135 (DMSO-d_6)
160
150
140
130
120
110
100
90
80
70
60
50
40
30
20
10
0
f1 (ppm)

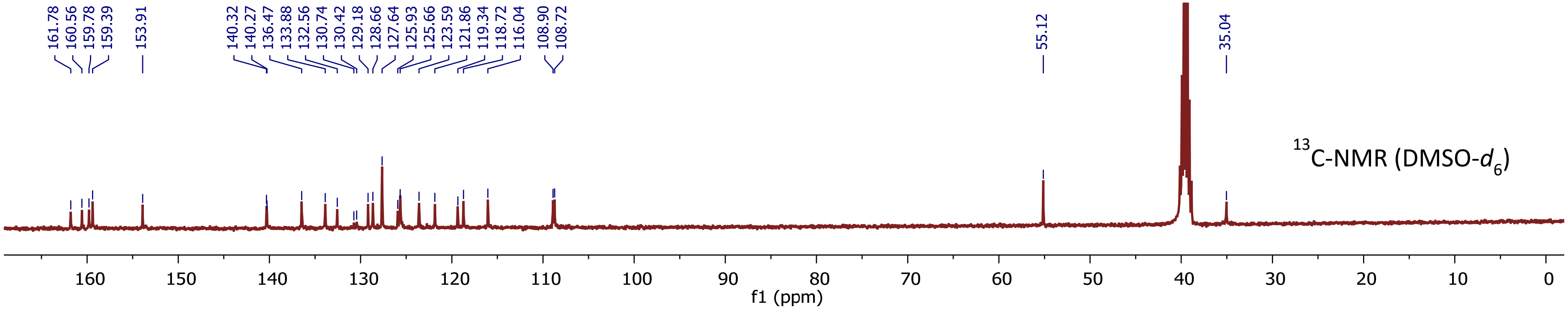

161.78
160.56
159.78
159.39
153.91
140.32
140.27
136.47
133.88
132.56
130.74
130.42
129.18
128.66
127.64
125.93
125.66
123.59
121.86
119.34
118.72
116.04
108.90
108.72
55.12
35.04
^{13}C-NMR (DMSO-d_6)
160
150
140
130
120
110
100
90
80
70
60
50
40
30
20
10
0
f1 (ppm)

EI MS (70eV)

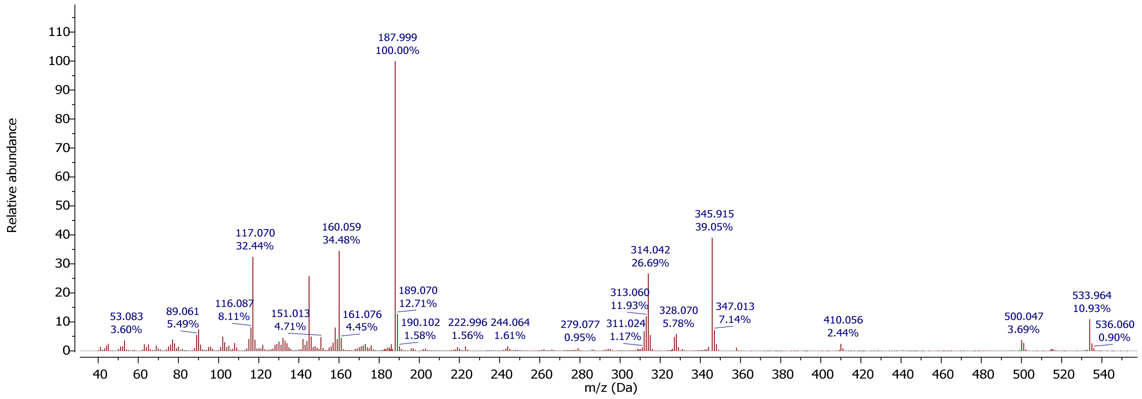

ESI-QTOF (positive ionization)

MS Zoomed Spectrum

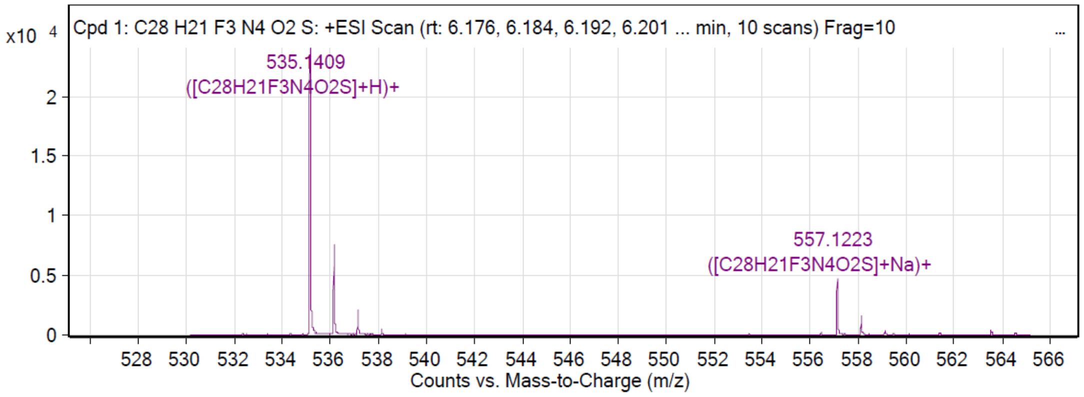

MS Spectrum Peak List

m/z	Calc *m/z*	Diff(ppm)	z	Abund	Formula	Ion
535.1409	535.141	0.18	1	24069.29	C28H21F3N4O2S	(M+H)+
536.1441	536.144	-0.1	1	7805.41	C28H21F3N4O2S	(M+H)+
537.1434	537.1424	-1.76	1	2137.66	C28H21F3N4O2S	(M+H)+
557.1223	557.123	1.13	1	4818.09	C28H21F3N4O2S	(M+Na)+
558.1259	558.126	0.08	1	1598.05	C28H21F3N4O2S	(M+Na)+
559.1254	559.1244	-1.85	1	469.72	C28H21F3N4O2S	(M+Na)+
560.1218	560.1249	5.6	1	46.38	C28H21F3N4O2S	(M+Na)+

4.17 Compound 26a

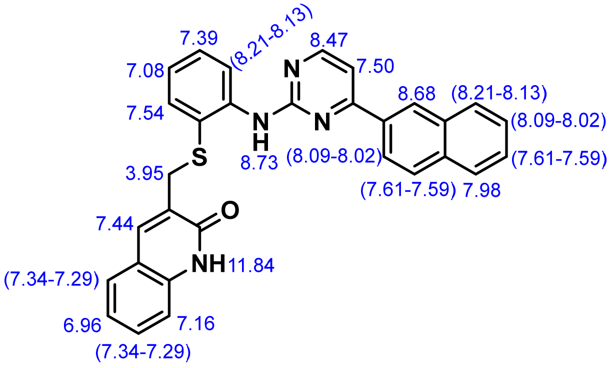

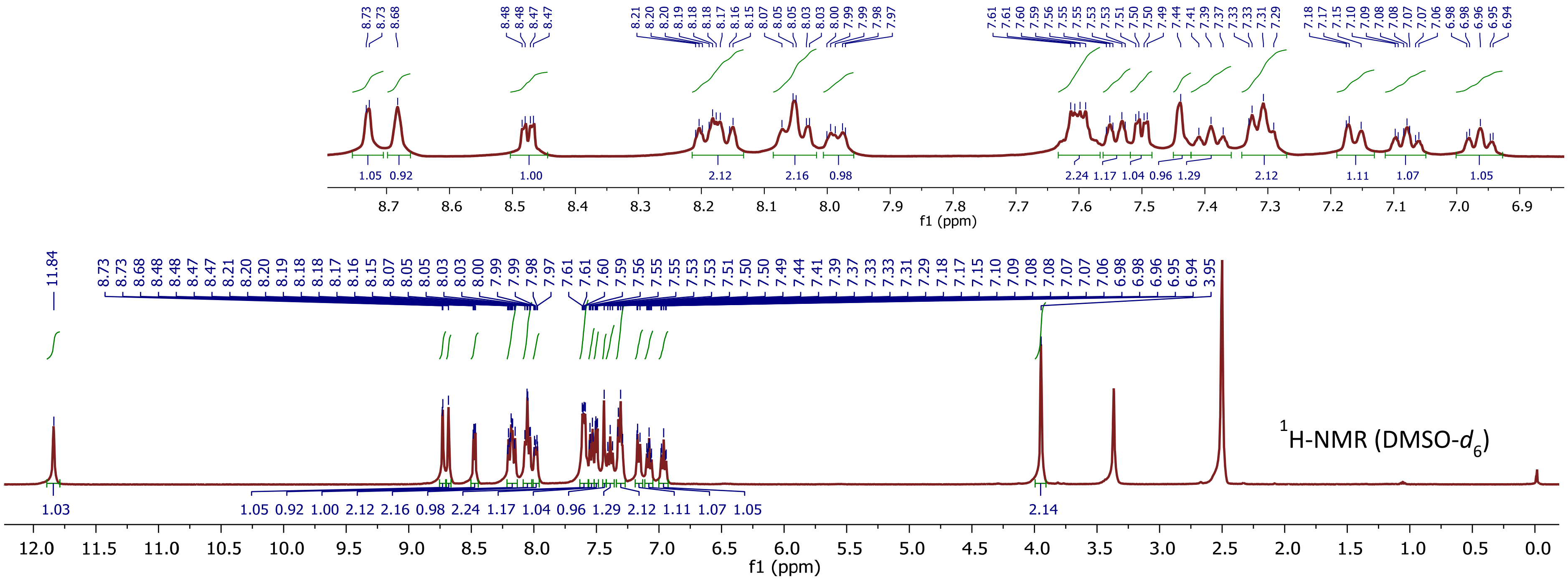

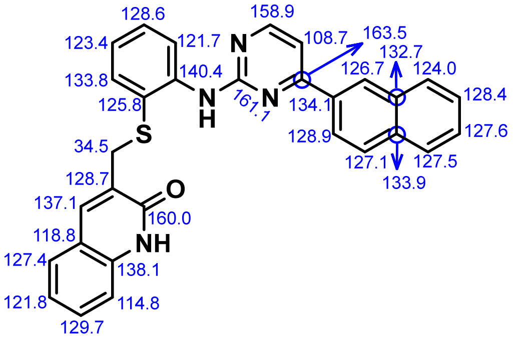

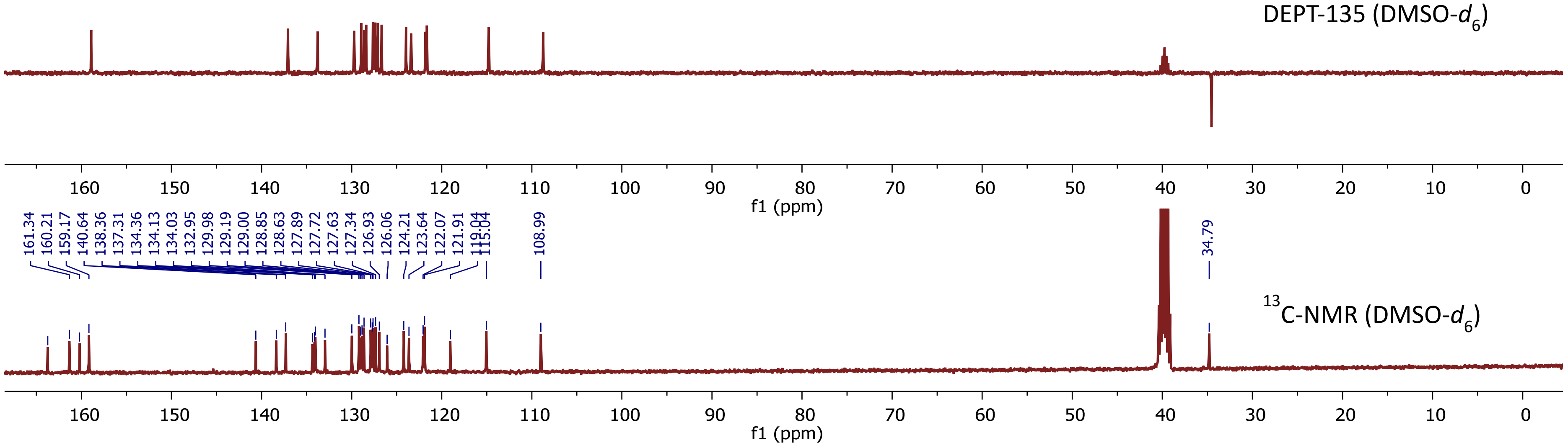
DEPT-135 (DMSO-d_6)
f1 (ppm)
161.34 160.21 159.17 140.64 138.36 137.31 134.36 134.13 134.03 132.95 129.98 129.19 129.00 128.85 128.63 127.89 127.72 127.63 127.34 126.93 126.06 124.21 123.64 122.07 121.91 119.04 115.04 108.99 34.79
^{13}C-NMR (DMSO-d_6)
f1 (ppm)

EI MS (70eV)

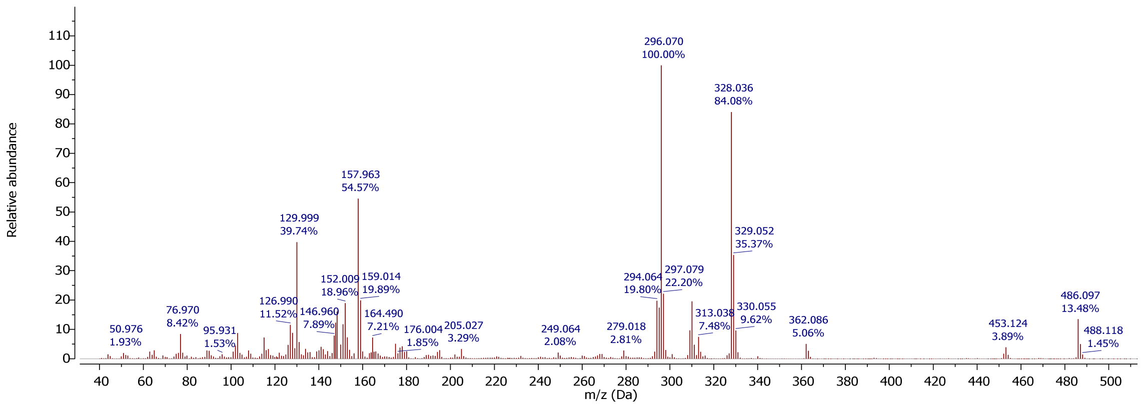

ESI-QTOF (positive ionization)

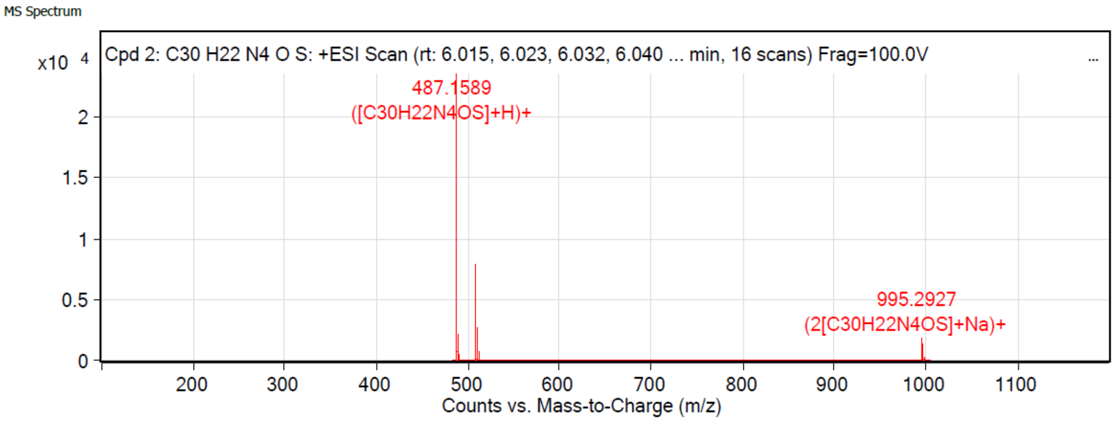

MS Spectrum Peak List

m/z	Calc *m/z*	Diff(ppm)	z	Abund	Formula	Ion
487.1589	487.1587	-0.29	1	23642.65	C30H22N4OS	(M+H)+
488.162	488.1617	-0.58	1	8337.6	C30H22N4OS	(M+H)+
489.1609	489.1604	-1.05	1	2287.91	C30H22N4OS	(M+H)+
509.1406	509.1407	0.17	1	7921.39	C30H22N4OS	(M+Na)+
510.1437	510.1437	-0.14	1	2736.4	C30H22N4OS	(M+Na)+
511.1429	511.1423	-1.19	1	797.99	C30H22N4OS	(M+Na)+
995.2927	995.2921	-0.6	1	1972.33	C30H22N4OS	(2M+Na)+
996.2959	996.2951	-0.79	1	1423.17	C30H22N4OS	(2M+Na)+
997.2974	997.2954	-2.07	1	597.2	C30H22N4OS	(2M+Na)+
998.2967	998.2958	-0.88	1	239.26	C30H22N4OS	(2M+Na)+

4.18 Compound 26b

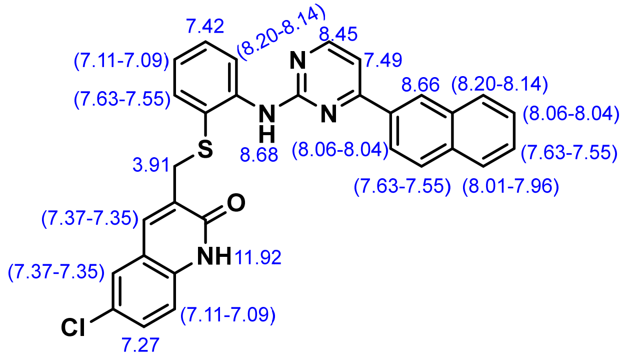

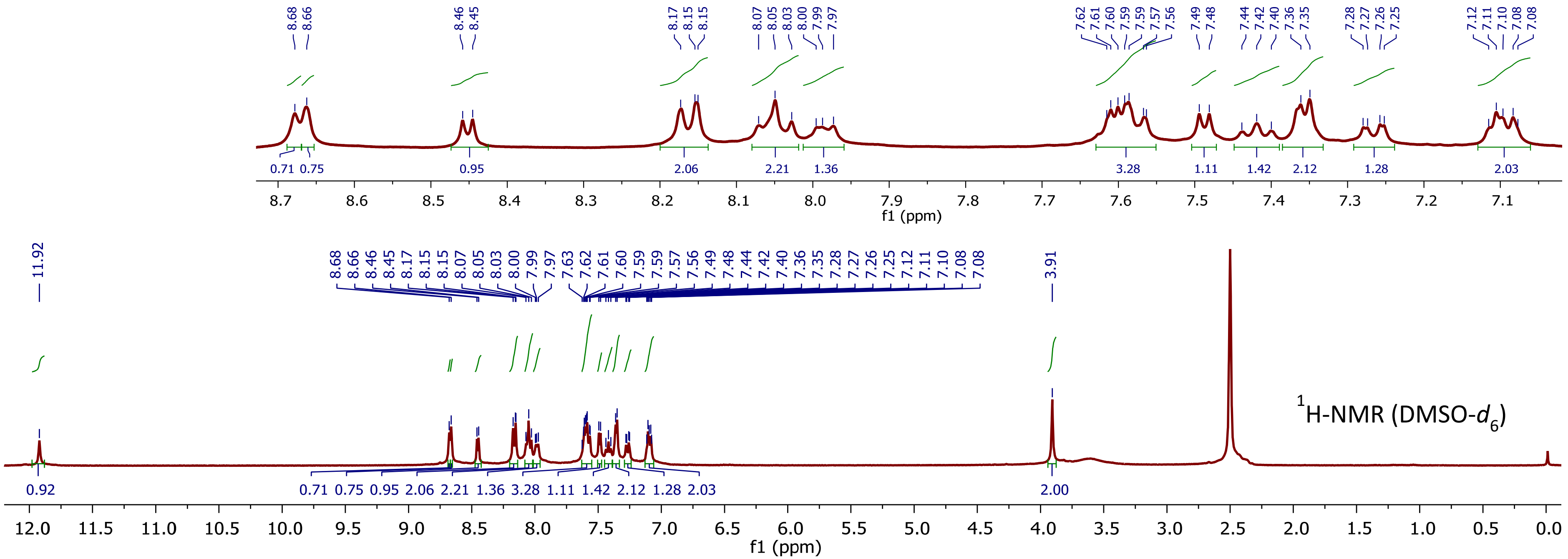

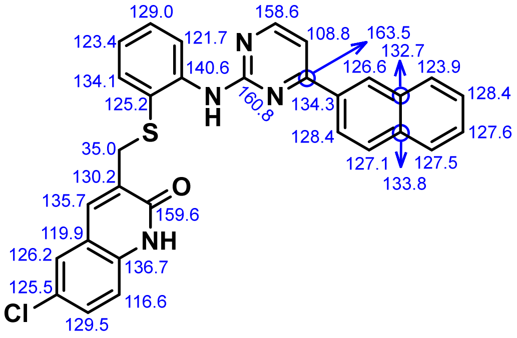
129.0
123.4
121.7
158.6
108.8
163.5
132.7
123.9
126.6
140.6
134.1
128.4
134.3
160.8
125.2
127.6
128.4
35.0
127.1
127.5
133.8
130.2
135.7
159.6
119.9
126.2
136.7
125.5
116.6
129.5
N
N
H
S
O
NH
Cl

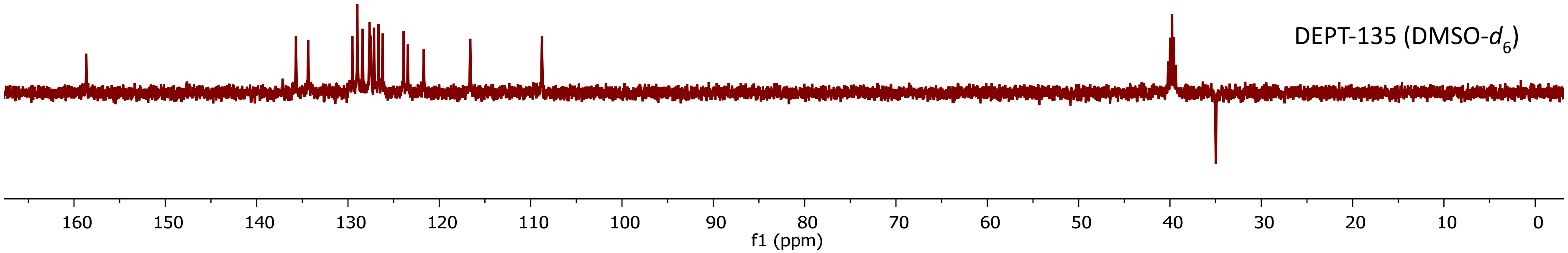
DEPT-135 (DMSO-d_6)
160 150 140 130 120 110 100 90 80 70 60 50 40 30 20 10 0
f1 (ppm)

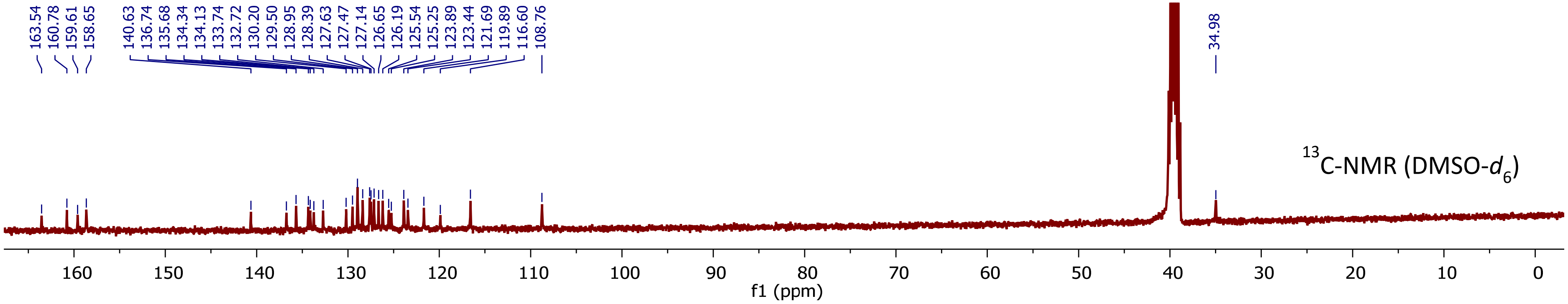
163.54
160.78
159.61
158.65
140.63
136.74
135.68
134.34
134.13
133.74
132.72
130.20
129.50
128.95
128.39
127.63
127.47
127.14
126.65
126.19
125.54
125.25
123.89
123.44
121.69
119.89
116.60
108.76
34.98
^{13}C-NMR (DMSO-d_6)
160 150 140 130 120 110 100 90 80 70 60 50 40 30 20 10 0
f1 (ppm)

EI MS (70eV)

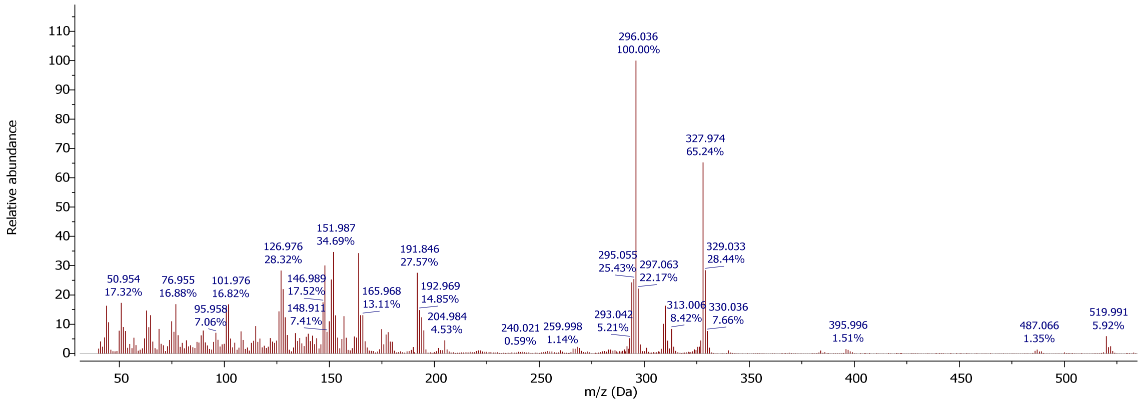

ESI-QTOF (positive ionization)

MS Spectrum

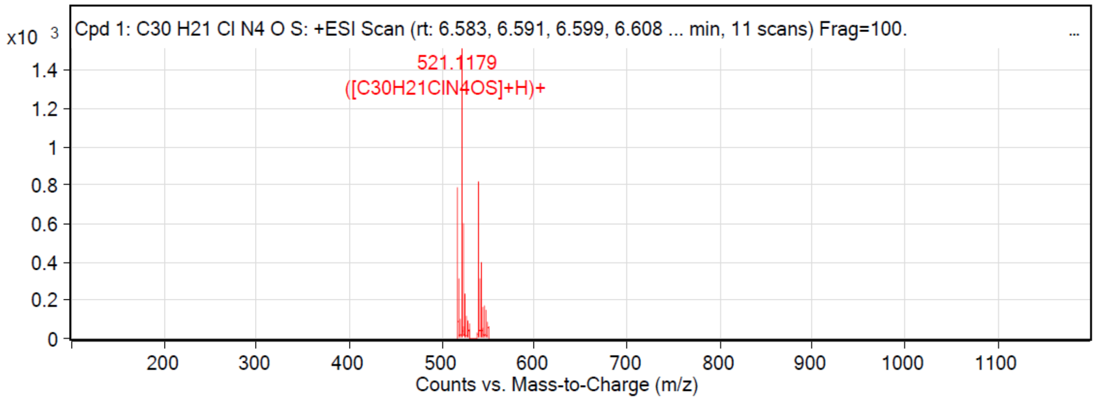

MS Spectrum Peak List

m/z	Calc *m/z*	Diff(ppm)	z	Abund	Formula	Ion
521.1179	521.1197	3.47	1	1600.01	C30H21ClN4OS	(M+H)+
522.1209	522.1228	3.62	1	533.33	C30H21ClN4OS	(M+H)+
523.1181	523.1179	-0.37	1	610.94	C30H21ClN4OS	(M+H)+
524.1182	524.1201	3.75	1	212.57	C30H21ClN4OS	(M+H)+
525.1234	525.1189	-8.57	1	48.47	C30H21ClN4OS	(M+H)+
543.1007	543.1017	1.8	1	407.77	C30H21ClN4OS	(M+Na)+
544.1089	544.1047	-7.63	1	128.08	C30H21ClN4OS	(M+Na)+
545.0977	545.0999	4.04	1	142.36	C30H21ClN4OS	(M+Na)+

4.19 Compound 26c

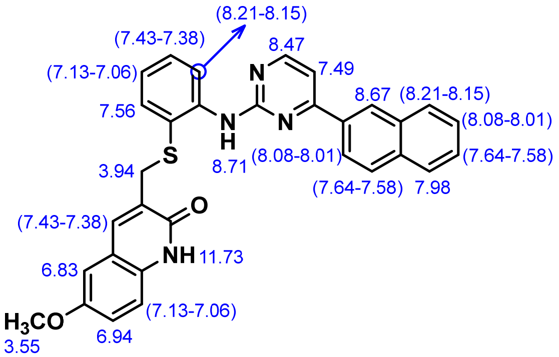

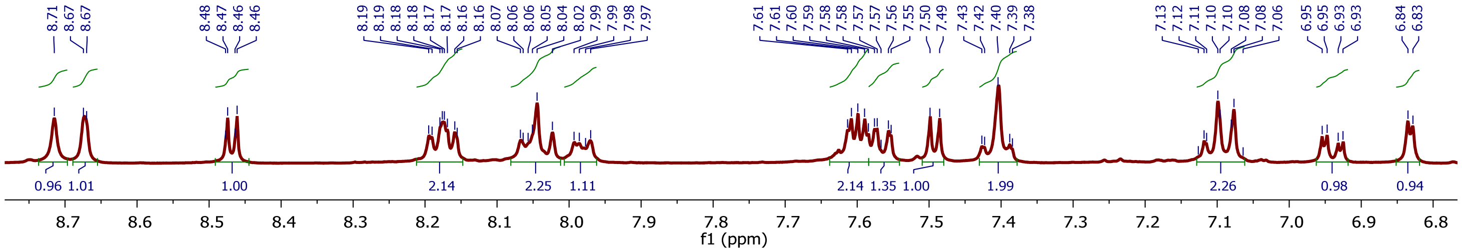

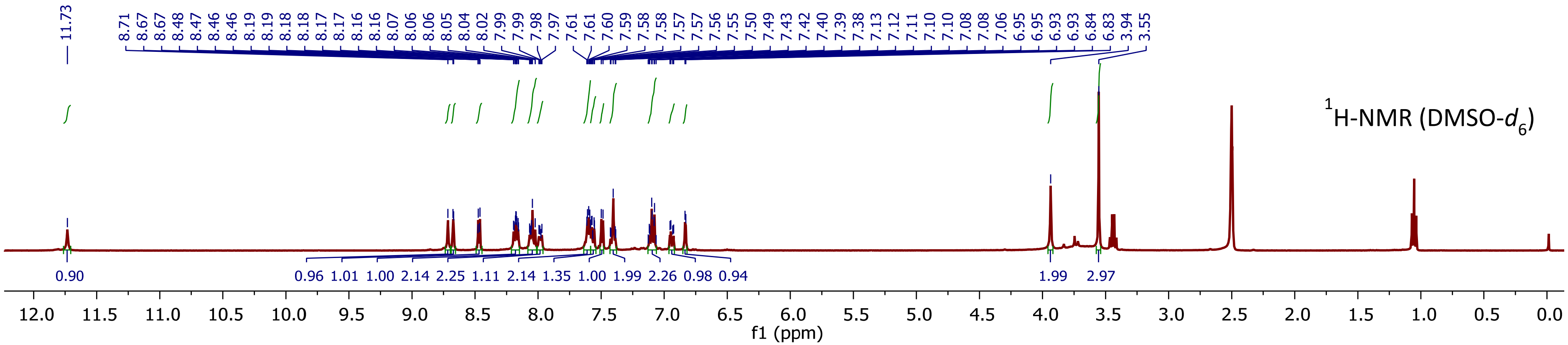

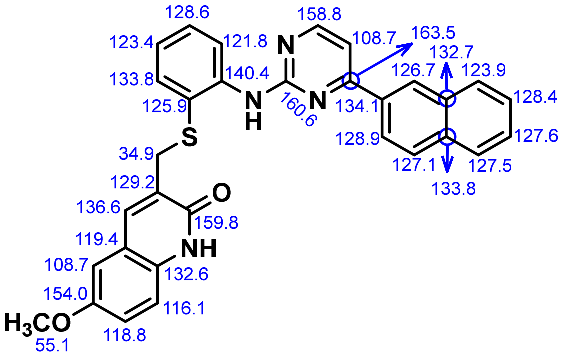

N
NH
H
S
O
NH
H3CO
128.6
123.4
121.8
140.4
133.8
125.9
158.8
108.7
163.5
132.7
123.9
126.7
128.4
160.6
134.1
128.9
127.6
127.1
127.5
133.8
34.9
129.2
136.6
159.8
119.4
108.7
132.6
154.0
116.1
118.8
55.1

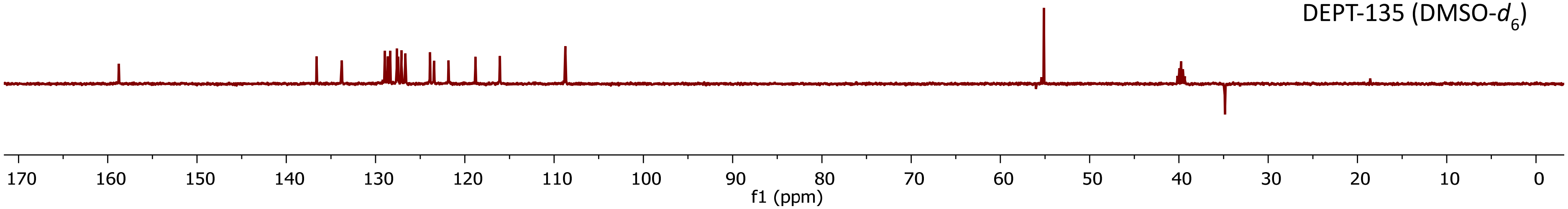

DEPT-135 (DMSO-d6)
170 160 150 140 130 120 110 100 90 80 70 60 50 40 30 20 10 0
f1 (ppm)

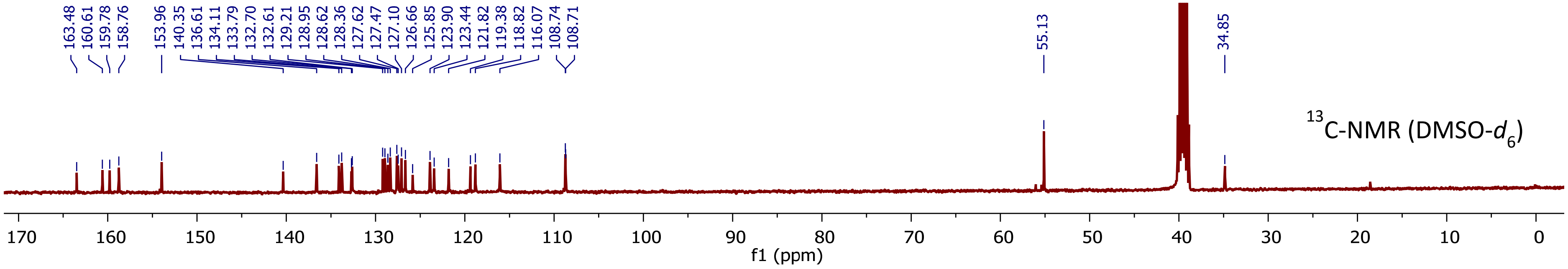

163.48
160.61
159.78
158.76
153.96
140.35
136.61
134.11
133.79
132.70
132.61
129.21
128.95
128.62
128.36
127.62
127.47
127.10
126.66
125.85
123.90
123.44
121.82
119.38
118.82
116.07
108.74
108.71
55.13
34.85
13C-NMR (DMSO-d6)
170 160 150 140 130 120 110 100 90 80 70 60 50 40 30 20 10 0
f1 (ppm)

EI MS (70eV)

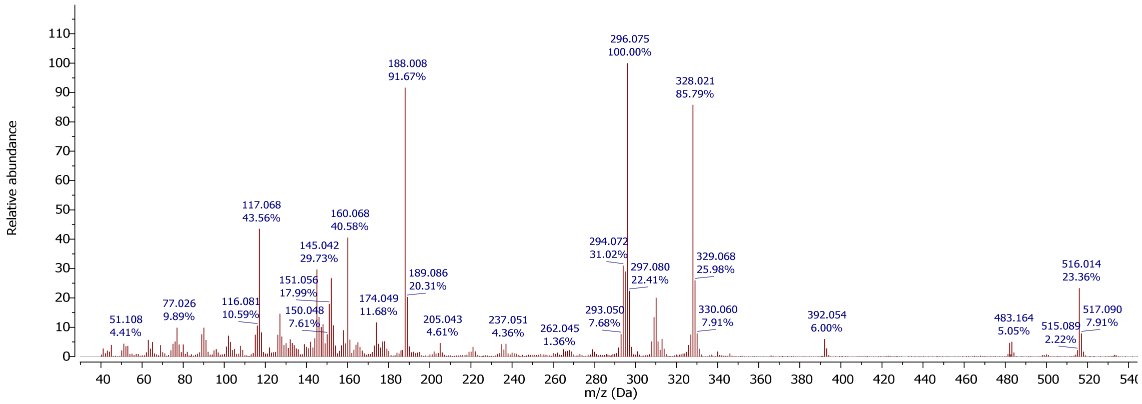

ESI-QTOF (positive ionization)

MS Zoomed Spectrum

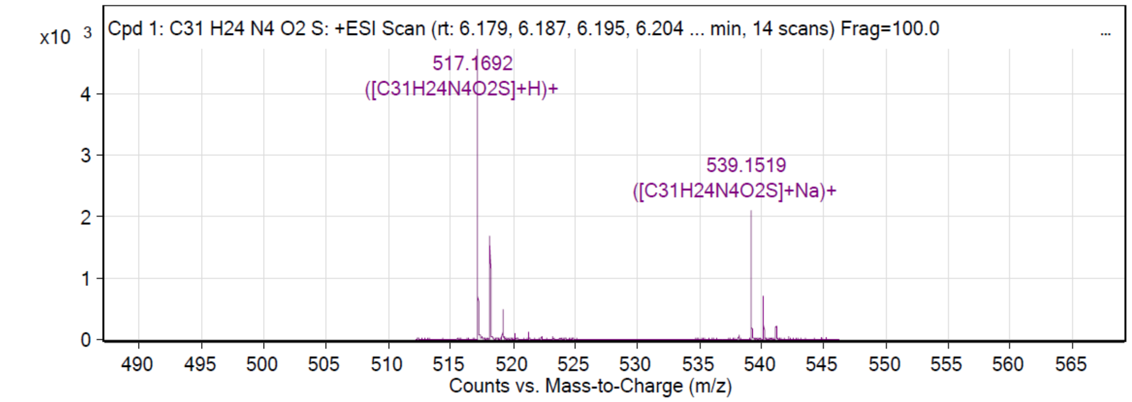

MS Spectrum Peak List

m/z	Calc *m/z*	Diff(ppm)	z	Abund	Formula	Ion
517.1692	517.1693	0.18	1	4806.15	C31H24N4O2S	(M+H)+
518.1721	518.1723	0.37	1	1741.63	C31H24N4O2S	(M+H)+
519.172	519.1712	-1.67	1	551.25	C31H24N4O2S	(M+H)+
520.1747	520.1717	-5.78	1	59.42	C31H24N4O2S	(M+H)+
539.1519	539.1512	-1.17	1	2227.44	C31H24N4O2S	(M+Na)+
540.1547	540.1543	-0.79	1	725.25	C31H24N4O2S	(M+Na)+
541.1539	541.1531	-1.47	1	174.48	C31H24N4O2S	(M+Na)+

4.20 Compound 27a

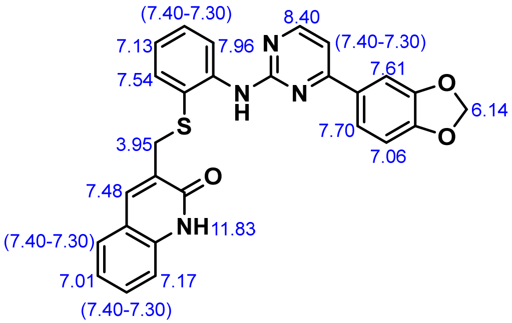

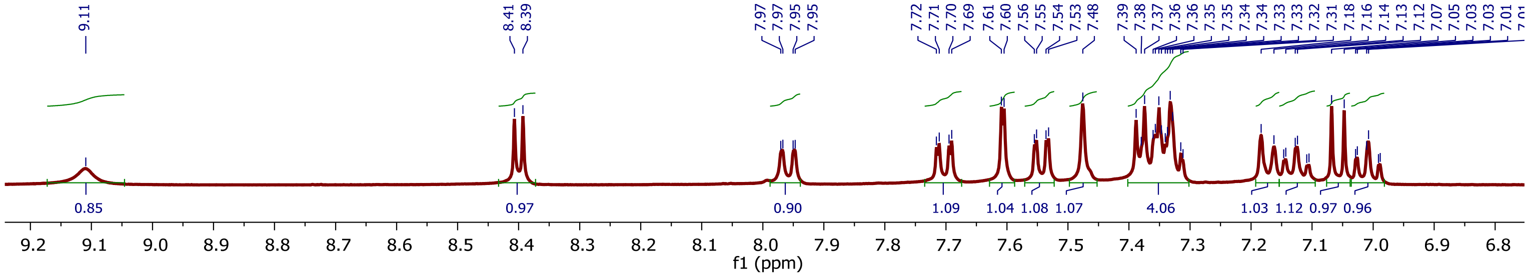

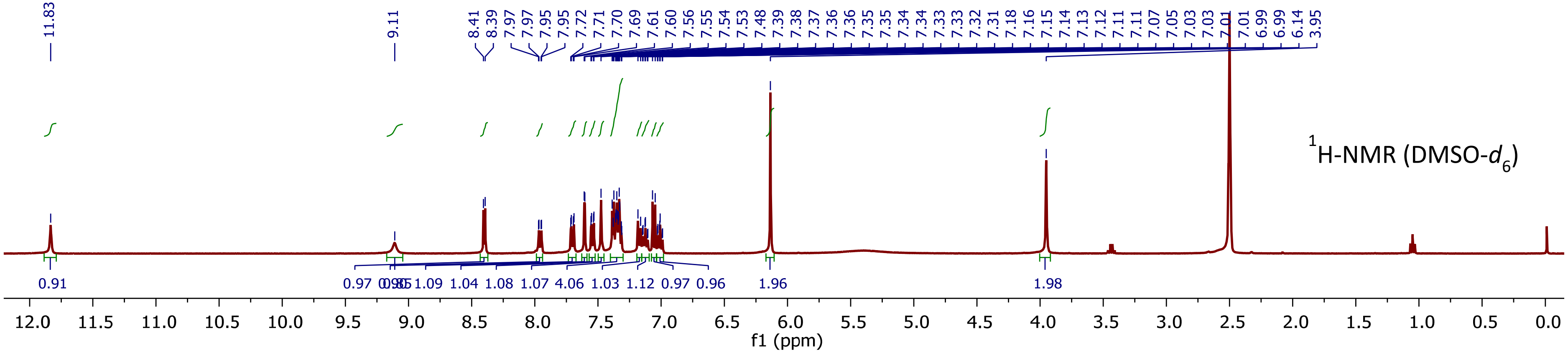

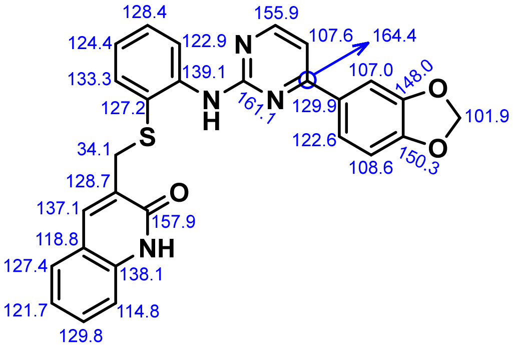
128.4
124.4
122.9
133.3
139.1
127.2
155.9
107.6
164.4
107.0
148.0
129.9
101.9
161.1
122.6
108.6
150.3
34.1
128.7
137.1
157.9
118.8
127.4
138.1
121.7
114.8
129.8
N
N
NH
H
S
O
O
O
NH

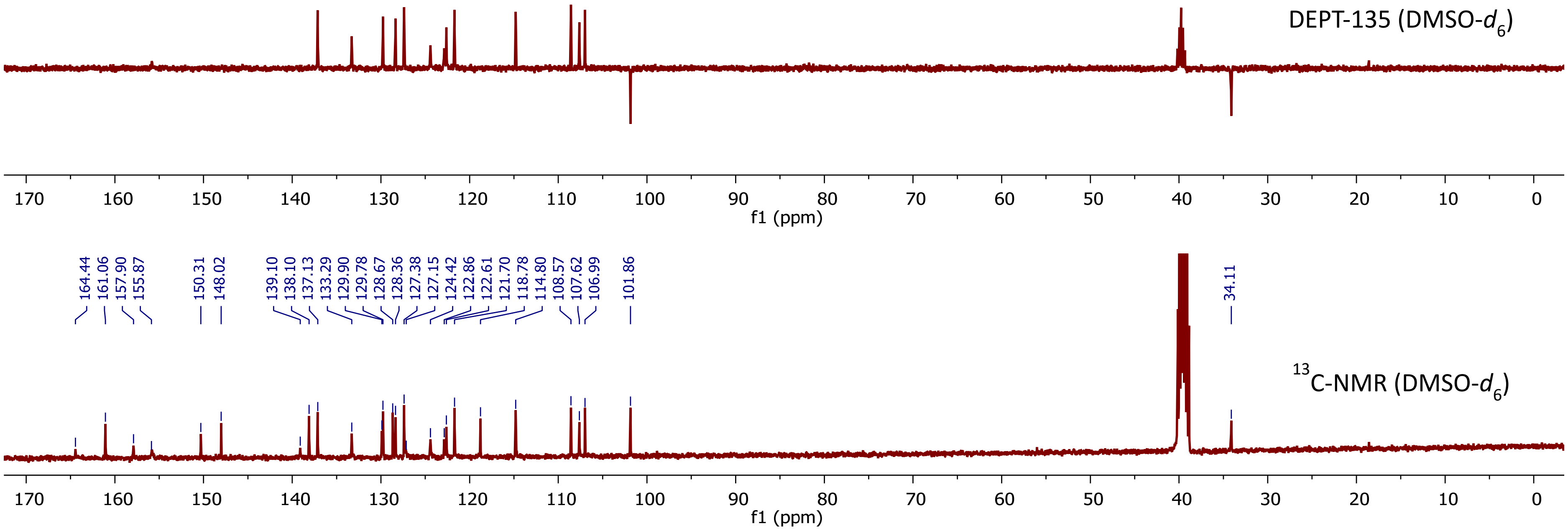
DEPT-135 (DMSO-d6)
170 160 150 140 130 120 110 100 90 80 70 60 50 40 30 20 10 0
f1 (ppm)
164.44
161.06
157.90
155.87
150.31
148.02
139.10
138.10
137.13
133.29
129.90
129.78
128.67
128.36
127.38
127.15
124.42
122.86
122.61
121.70
118.78
114.80
108.57
107.62
106.99
101.86
34.11
13C-NMR (DMSO-d6)
170 160 150 140 130 120 110 100 90 80 70 60 50 40 30 20 10 0
f1 (ppm)

EI MS (70eV)

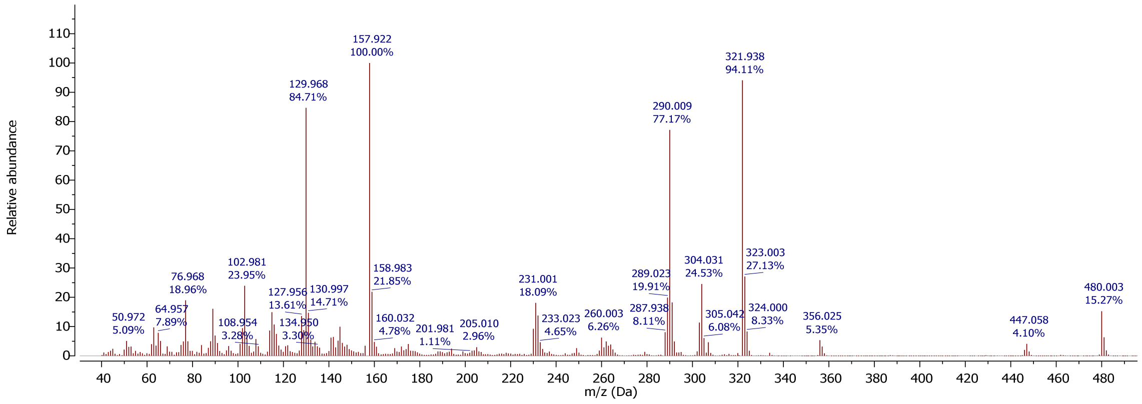

ESI-QTOF (positive ionization)

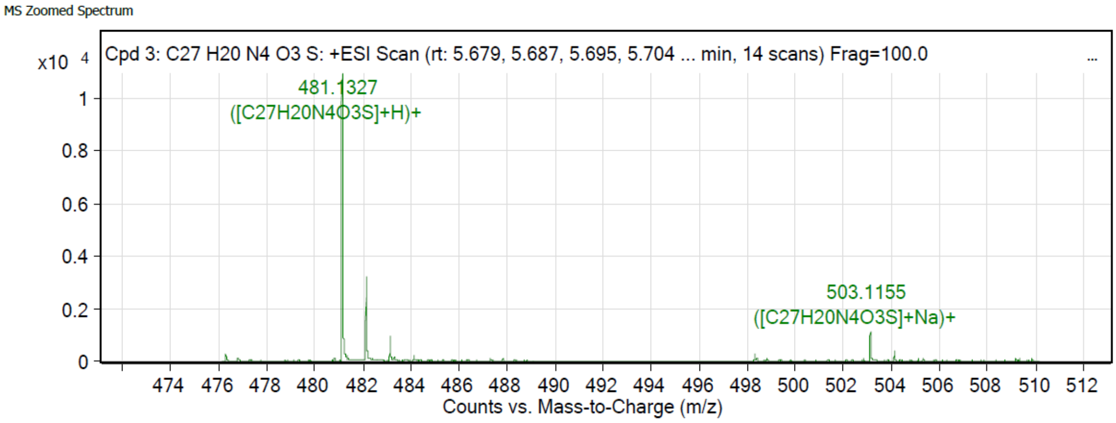

MS Spectrum Peak List

m/z	Calc *m/z*	Diff(ppm)	z	Abund	Formula	Ion
481.1327	481.1329	0.32	1	10997.75	C27H20N4O3S	(M+H)+
482.1356	482.1359	0.5	1	3385.77	C27H20N4O3S	(M+H)+
483.1344	483.1342	-0.48	1	1002.74	C27H20N4O3S	(M+H)+
484.1349	484.1349	0.03	1	191.21	C27H20N4O3S	(M+H)+
503.1155	503.1148	-1.41	1	1172.9	C27H20N4O3S	(M+Na)+
504.1165	504.1178	2.71	1	371.69	C27H20N4O3S	(M+Na)+
505.1164	505.1161	-0.45	1	64.05	C27H20N4O3S	(M+Na)+

4.21 Compound 27b

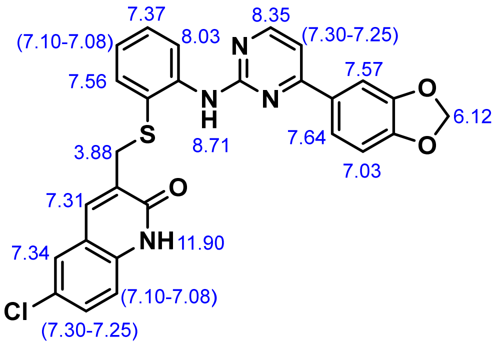

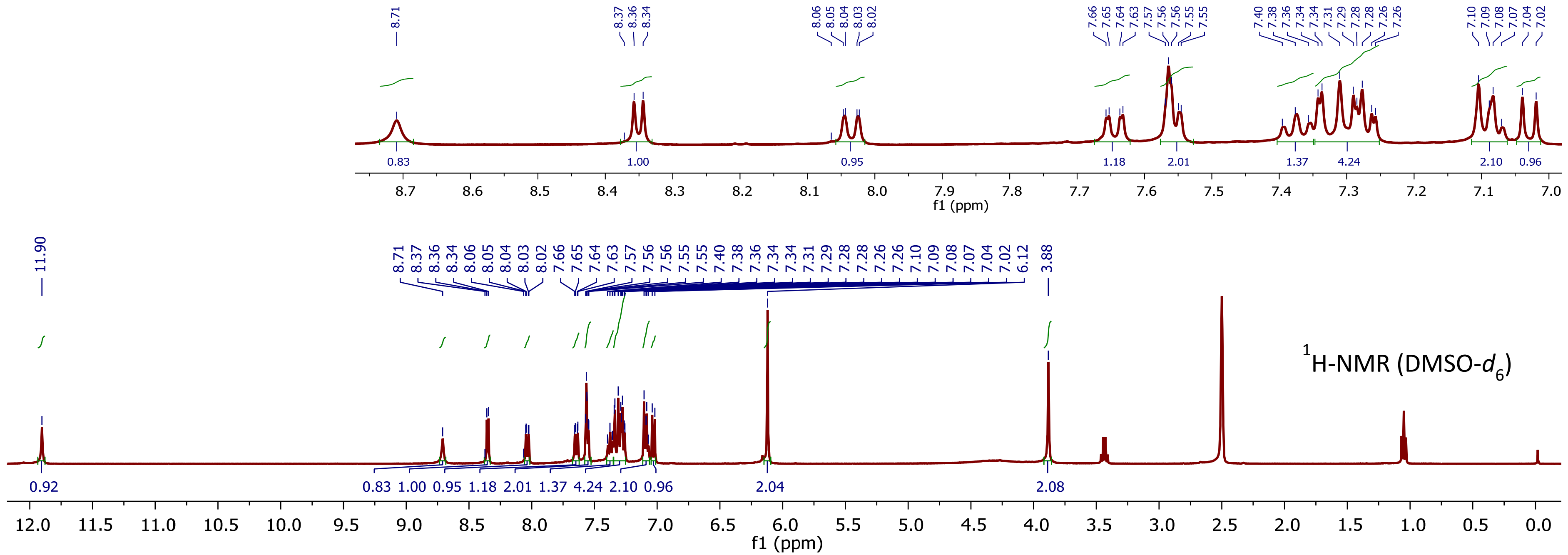

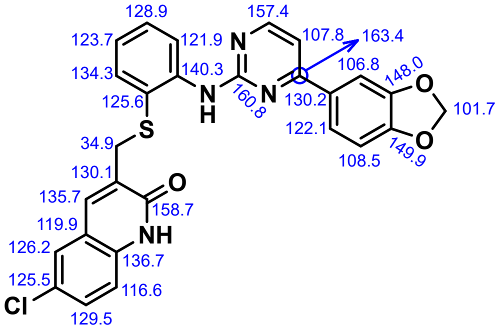

128.9
157.4
123.7
121.9
107.8
163.4
140.3
106.8
148.0
134.3
125.6
160.8
130.2
101.7
122.1
34.9
130.1
108.5
149.9
135.7
158.7
119.9
126.2
136.7
125.5
116.6
129.5
Cl
NH
O
S
N
H

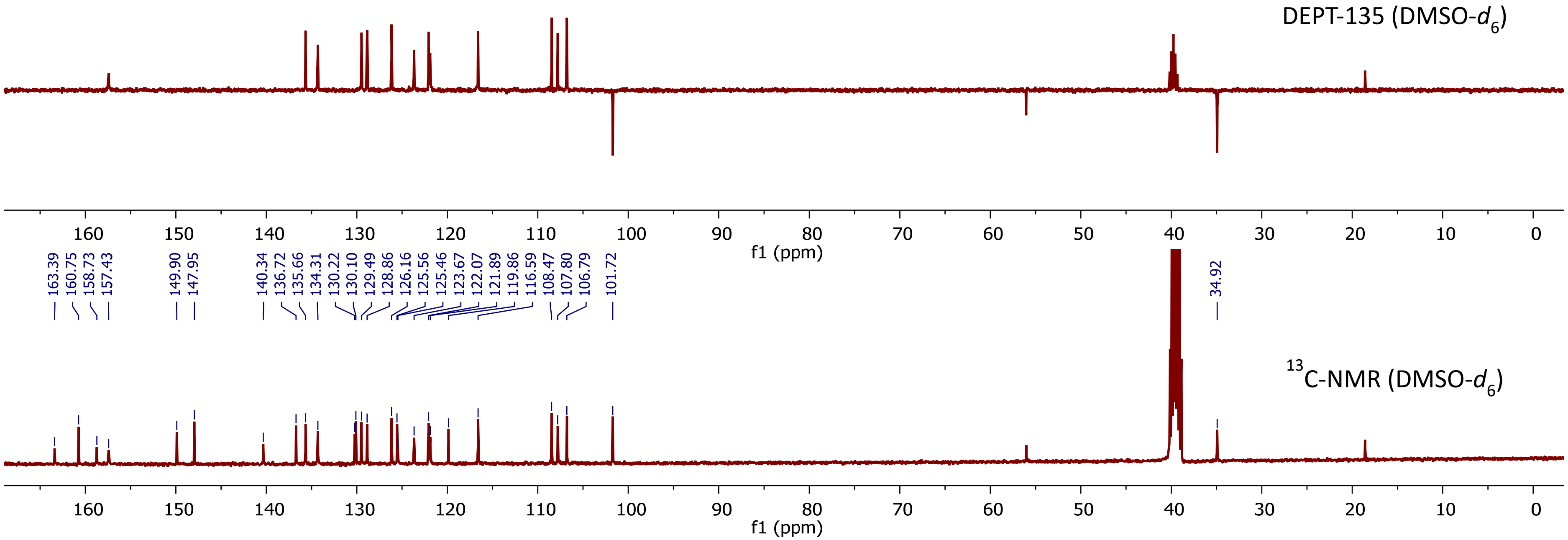

DEPT-135 (DMSO-d6)
f1 (ppm)
163.39
160.75
158.73
157.43
149.90
147.95
140.34
136.72
135.66
134.31
130.22
130.10
129.49
128.86
126.16
125.56
125.46
123.67
122.07
121.89
119.86
116.59
108.47
107.80
106.79
101.72
34.92
13C-NMR (DMSO-d6)
f1 (ppm)

EI MS (70eV)

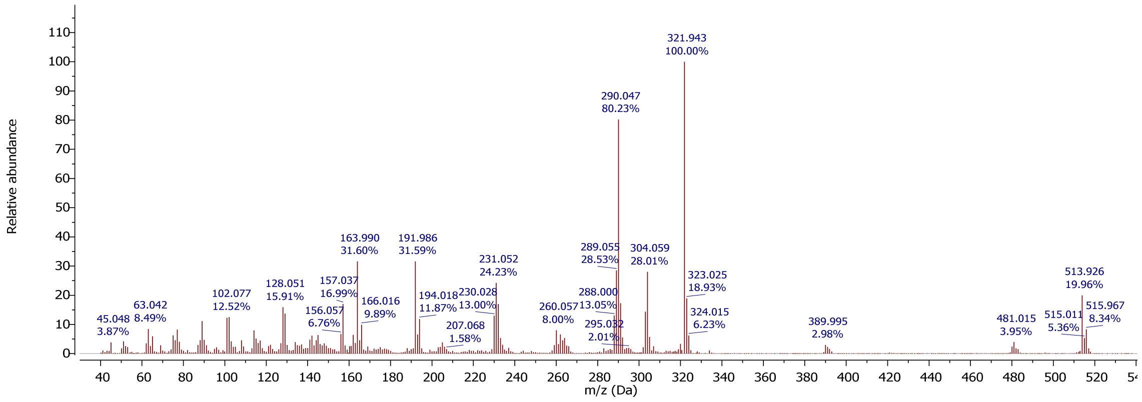

ESI-QTOF (positive ionization)

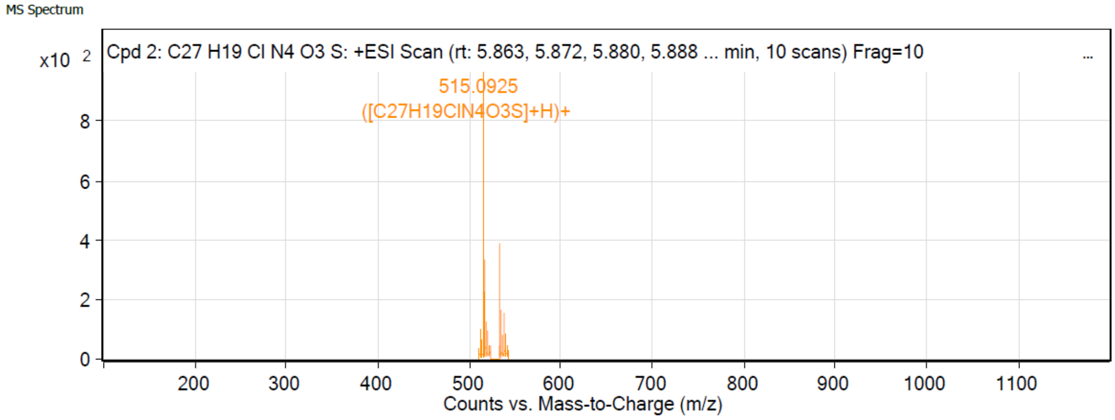

MS Spectrum Peak List

m/z	Calc *m/z*	Diff(ppm)	z	Abund	Formula	Ion
515.0925	515.0939	2.66	1	991.42	C27H19ClN4O3S	(M+H)+
516.0936	516.0969	6.48	1	197.51	C27H19ClN4O3S	(M+H)+
517.0902	517.092	3.48	1	309.58	C27H19ClN4O3S	(M+H)+
518.0905	518.0943	7.26	1	47.33	C27H19ClN4O3S	(M+H)+
537.0721	537.0759	6.91	1	67.66	C27H19ClN4O3S	(M+Na)+

4.22 Compound 27c

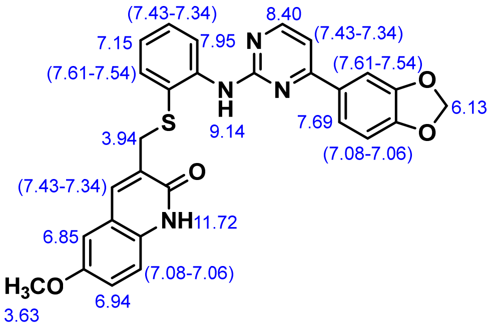

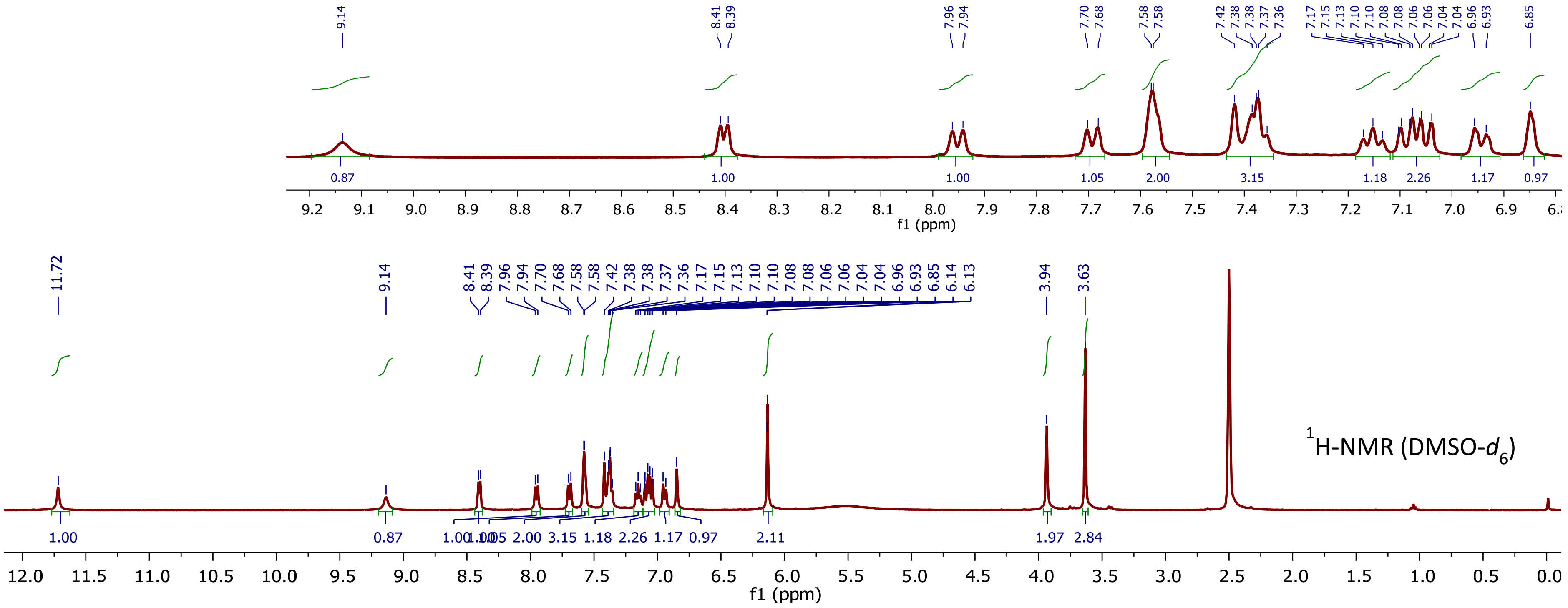

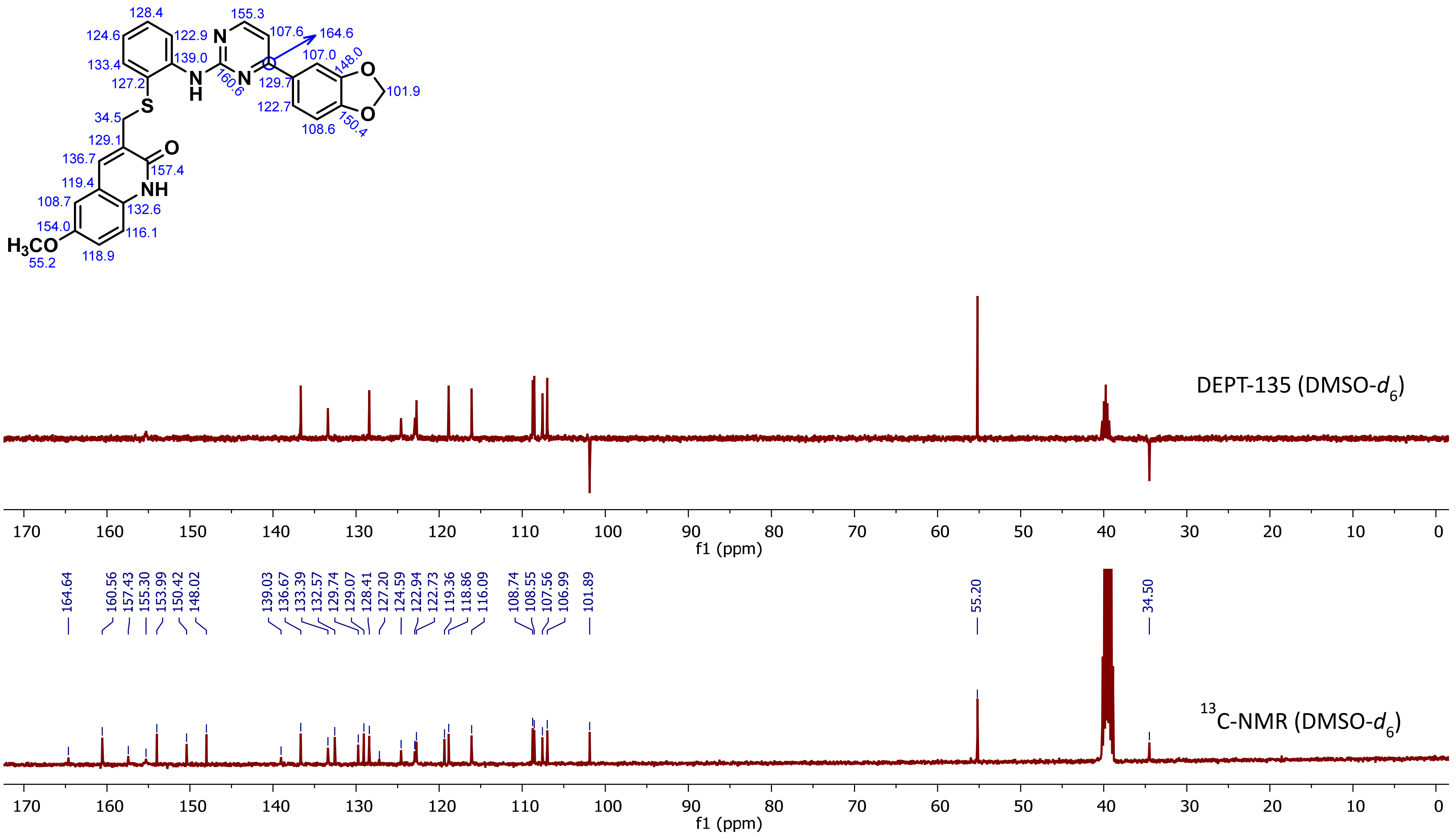
128.4
155.3
124.6
122.9
107.6
164.6
139.0
107.0
148.0
133.4
160.6
129.7
101.9
127.2
122.7
34.5
108.6
150.4
129.1
136.7
157.4
119.4
108.7
132.6
154.0
116.1
55.2
118.9
DEPT-135 (DMSO-d6)
170 160 150 140 130 120 110 100 90 80 70 60 50 40 30 20 10 0
f1 (ppm)
164.64
160.56
157.43
155.30
153.99
150.42
148.02
139.03
136.67
133.39
132.57
129.74
129.07
128.41
127.20
124.59
122.94
122.73
119.36
118.86
116.09
108.74
108.55
107.56
106.99
101.89
55.20
34.50
13C-NMR (DMSO-d6)
170 160 150 140 130 120 110 100 90 80 70 60 50 40 30 20 10 0
f1 (ppm)

EI MS (70eV)

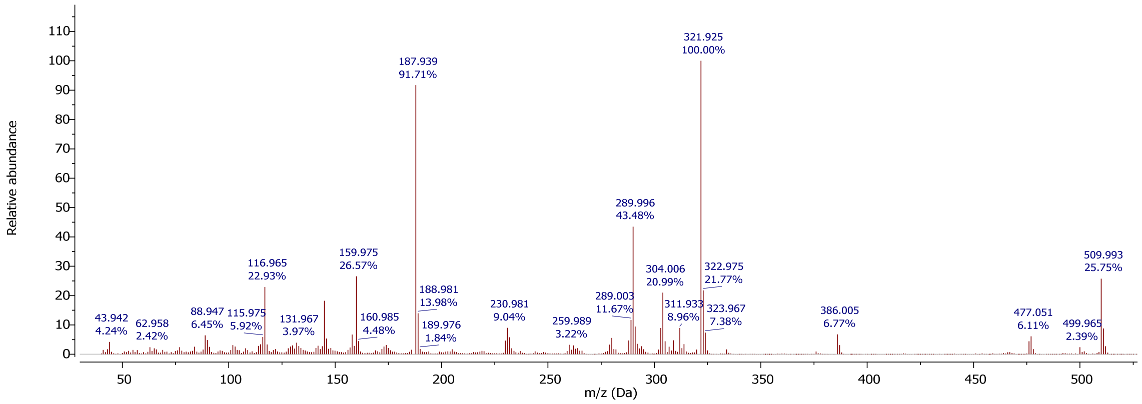

ESI-QTOF (positive ionization)

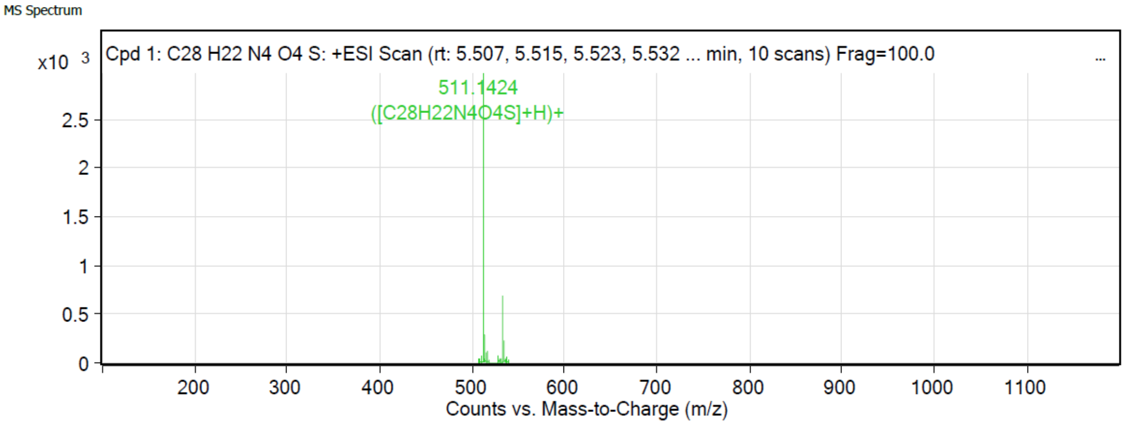

MS Spectrum Peak List

m/z	Calc *m/z*	Diff(ppm)	z	Abund	Formula	Ion
511.1424	511.1435	2.08	1	3015.56	C28H22N4O4S	(M+H)+
512.1468	512.1465	-0.68	1	976.06	C28H22N4O4S	(M+H)+
513.1478	513.145	-5.53	1	260.68	C28H22N4O4S	(M+H)+
533.124	533.1254	2.63	1	706.64	C28H22N4O4S	(M+Na)+
534.1277	534.1284	1.32	1	240.8	C28H22N4O4S	(M+Na)+
535.1246	535.1269	4.35	1	21.18	C28H22N4O4S	(M+Na)+

4.23 Compound 28a

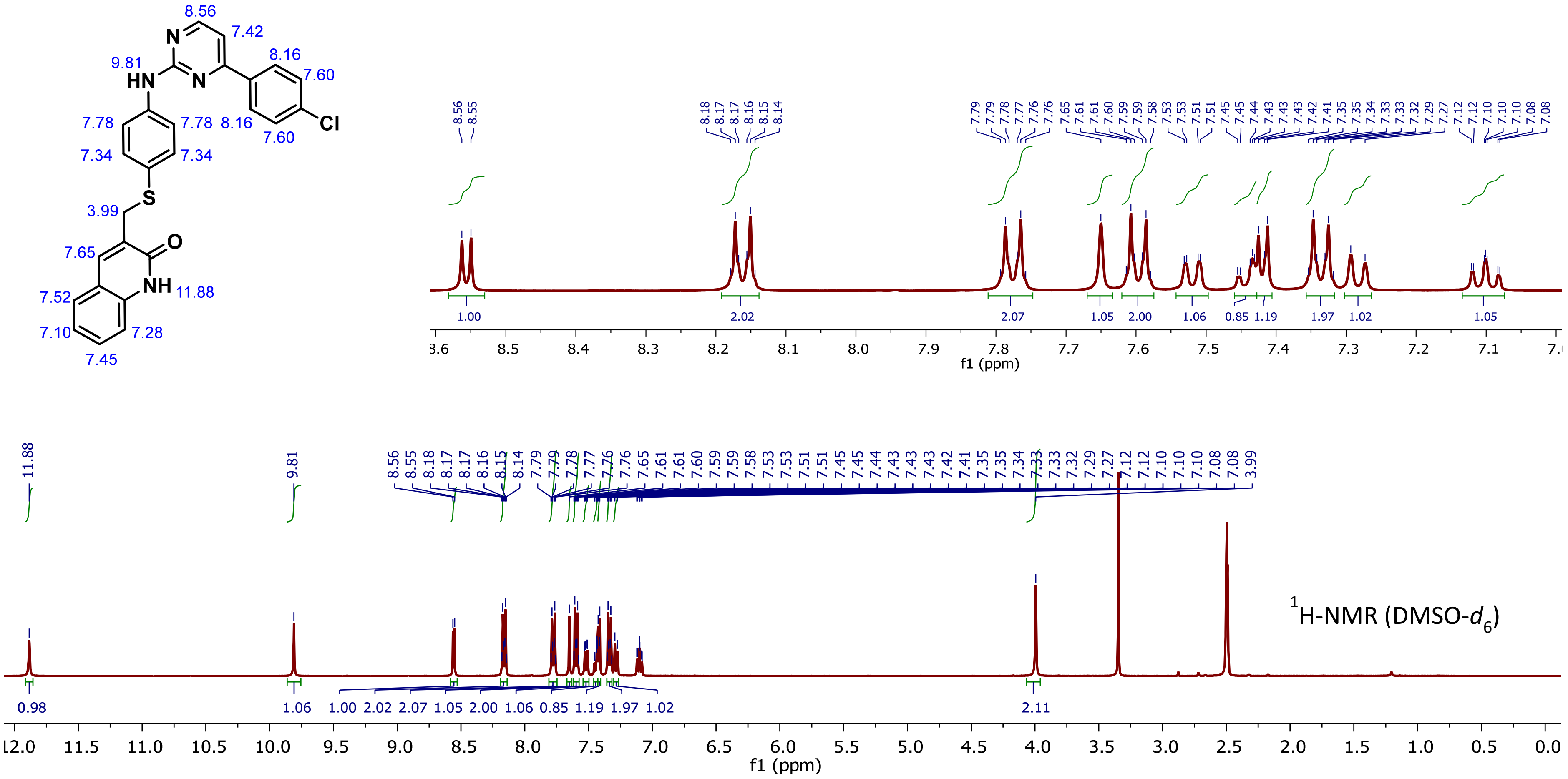

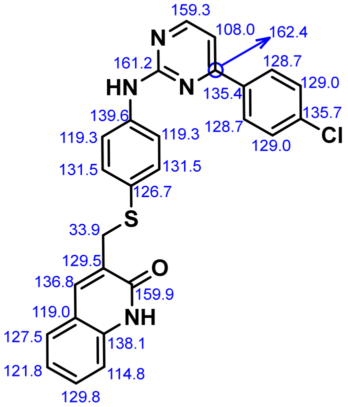

159.3
N
108.0
162.4
128.7
161.2
HN
N
135.4
129.0
139.6
135.7
128.7
Cl
119.3
119.3
129.0
131.5
131.5
126.7
33.9
S
129.5
O
136.8
159.9
119.0
NH
127.5
138.1
121.8
114.8
129.8

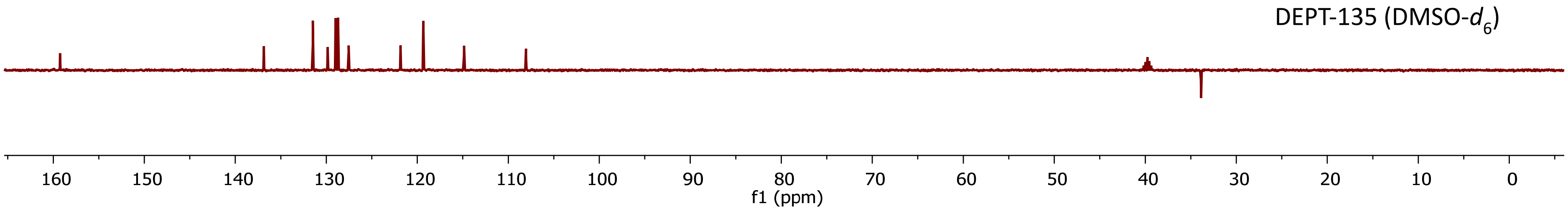

DEPT-135 (DMSO-d_6)
160
150
140
130
120
110
100
90
80
70
60
50
40
30
20
10
0
f1 (ppm)

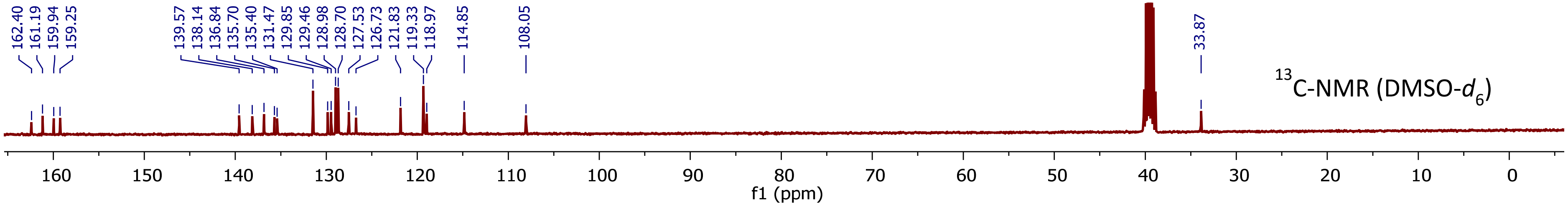

162.40
161.19
159.94
159.25
139.57
138.14
136.84
135.70
135.40
131.47
129.85
129.46
128.98
128.70
127.53
126.73
121.83
119.33
118.97
114.85
108.05
33.87
^{13}C-NMR (DMSO-d_6)
160
150
140
130
120
110
100
90
80
70
60
50
40
30
20
10
0
f1 (ppm)

EI MS (70eV)

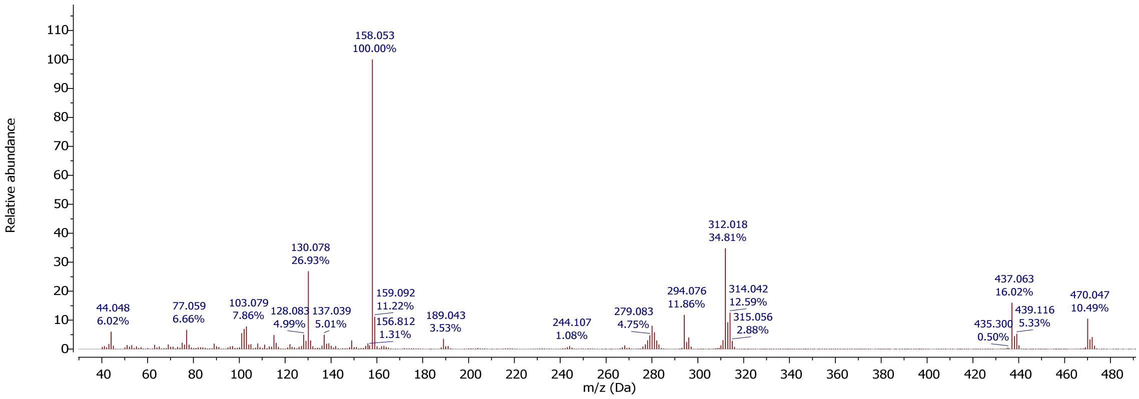

ESI-QTOF (positive ionization)

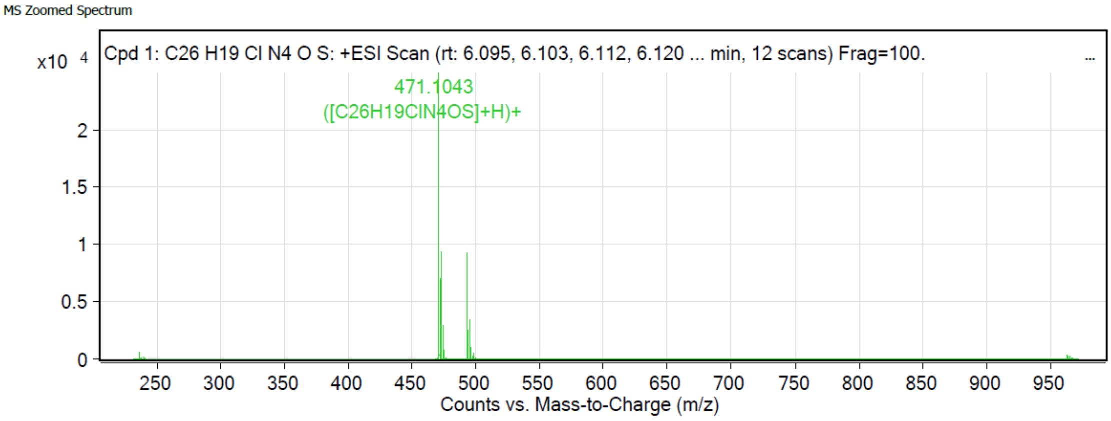

MS Spectrum Peak List

m/z	Calc *m/z*	Diff(ppm)	z	Abund	Formula	Ion
236.0563	236.0557	-2.45	2	703.69	C26H19ClN4OS	(M+2H)+2
471.1043	471.1041	-0.42	1	25044.35	C26H19ClN4OS	(M+H)+
472.107	472.1071	0.09	1	7196.44	C26H19ClN4OS	(M+H)+
473.1022	473.102	-0.36	1	9531.86	C26H19ClN4OS	(M+H)+
474.1047	474.1043	-0.84	1	3006.96	C26H19ClN4OS	(M+H)+
493.086	493.086	0.09	1	9473.16	C26H19ClN4OS	(M+Na)+
494.0895	494.089	-0.97	1	2629.7	C26H19ClN4OS	(M+Na)+
495.0836	495.084	0.67	1	3647.4	C26H19ClN4OS	(M+Na)+
496.0859	496.0863	0.7	1	1086.21	C26H19ClN4OS	(M+Na)+
963.1817	963.1828	1.18	1	354.06	C26H19ClN4OS	(2M+Na)+

4.24 Compound 28b

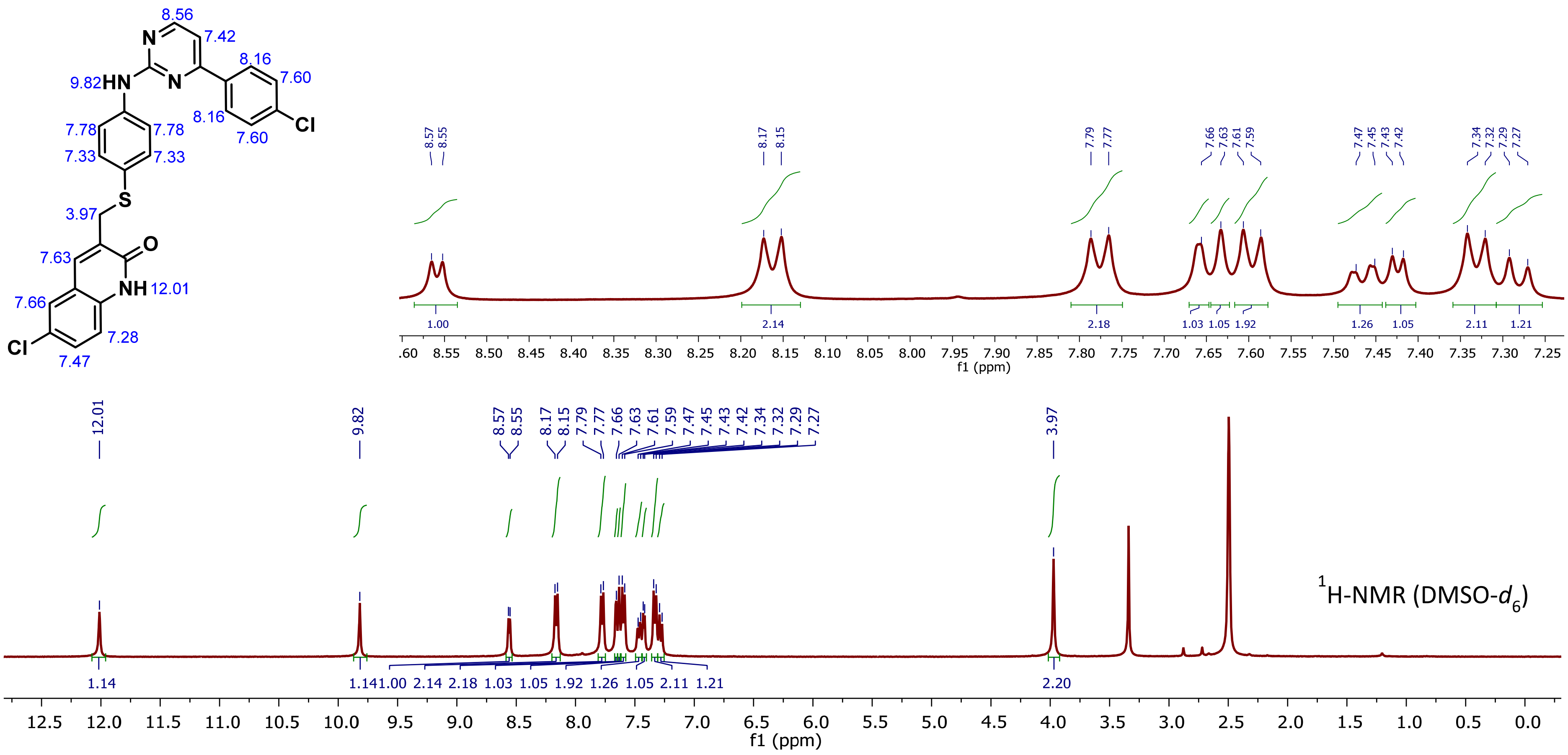

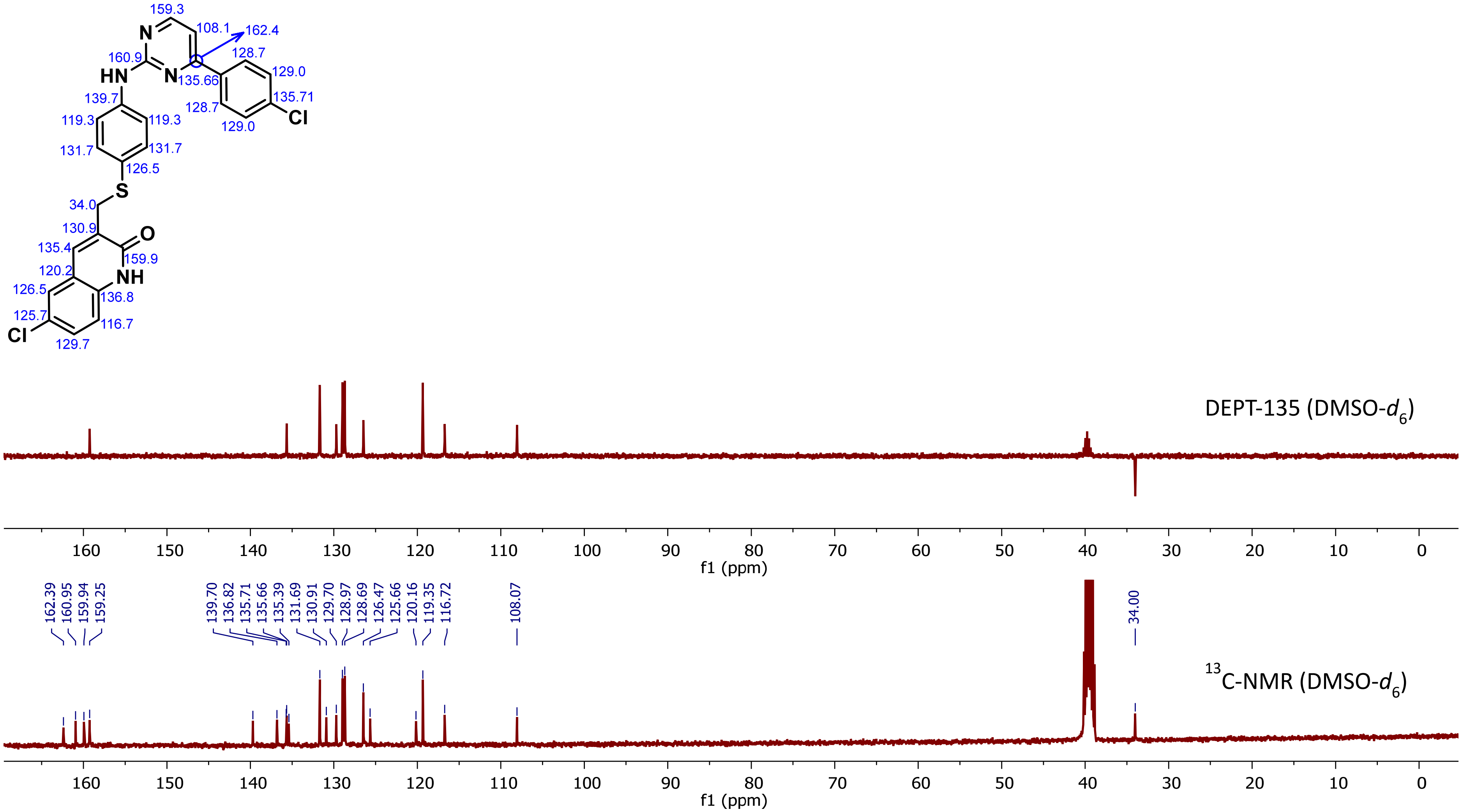

159.3
108.1
162.4
128.7
129.0
160.9
HN
N
135.66
135.71
Cl
139.7
128.7
129.0
119.3
119.3
131.7
131.7
126.5
S
34.0
130.9
O
135.4
159.9
120.2
NH
126.5
136.8
125.7
116.7
Cl
129.7
DEPT-135 (DMSO-d6)
160 150 140 130 120 110 100 90 80 70 60 50 40 30 20 10 0
f1 (ppm)
162.39
160.95
159.94
159.25
139.70
136.82
135.71
135.66
135.39
131.69
130.91
129.70
128.97
128.69
126.47
125.66
120.16
119.35
116.72
108.07
34.00
13C-NMR (DMSO-d6)
160 150 140 130 120 110 100 90 80 70 60 50 40 30 20 10 0
f1 (ppm)

EI MS (70eV)

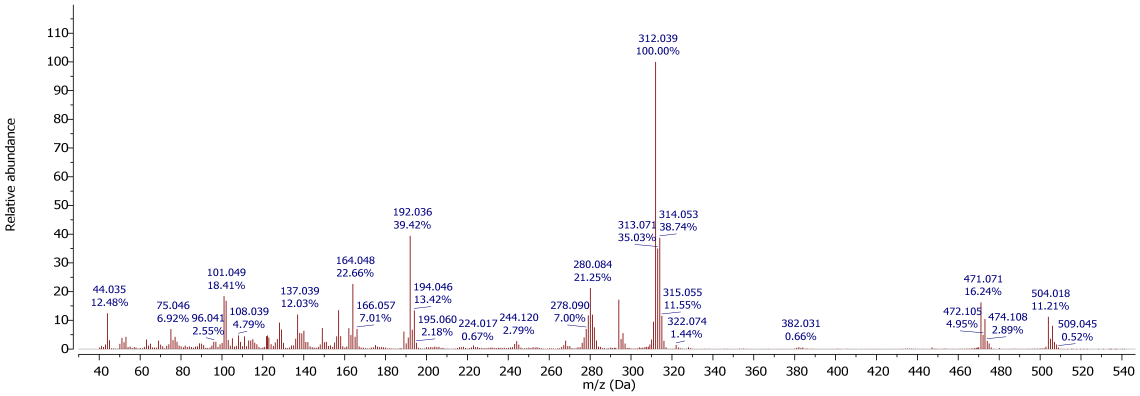

ESI-QTOF (positive ionization)

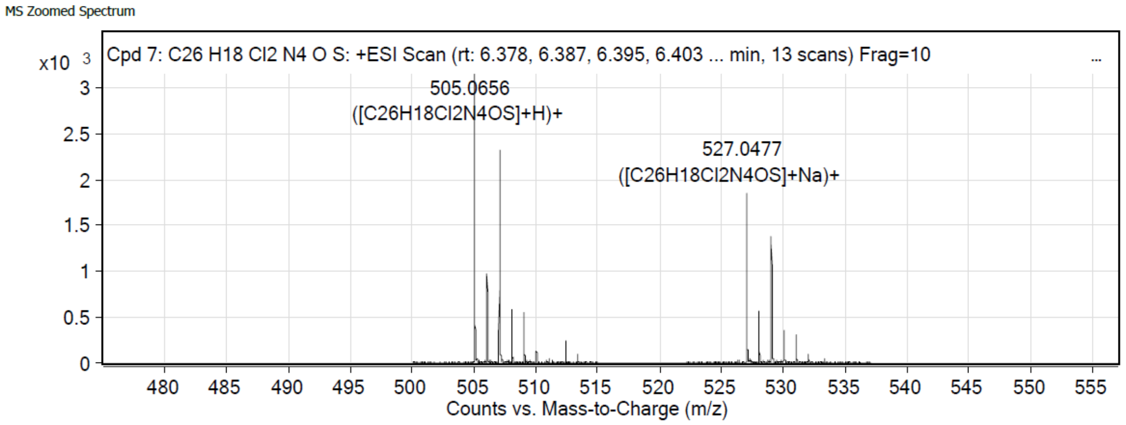

MS Spectrum Peak List

m/z	Calc *m/z*	Diff(ppm)	z	Abund	Formula	Ion
505.0656	505.0651	-0.89	1	3256.15	C26H18Cl2N4OS	(M+H)+
506.0683	506.0681	-0.35	1	1040.6	C26H18Cl2N4OS	(M+H)+
507.0631	507.0627	-0.92	1	2374.85	C26H18Cl2N4OS	(M+H)+
508.0668	508.0653	-3	1	635.36	C26H18Cl2N4OS	(M+H)+
509.0606	509.0608	0.34	1	581.17	C26H18Cl2N4OS	(M+H)+
527.0477	527.0471	-1.2	1	1915.42	C26H18Cl2N4OS	(M+Na)+
528.0503	528.05	-0.5	1	572.96	C26H18Cl2N4OS	(M+Na)+
529.0442	529.0446	0.76	1	1465.6	C26H18Cl2N4OS	(M+Na)+
530.0473	530.0472	-0.16	1	357.7	C26H18Cl2N4OS	(M+Na)+
531.0453	531.0427	-4.88	1	308.78	C26H18Cl2N4OS	(M+Na)+

4.25 Compound 28c

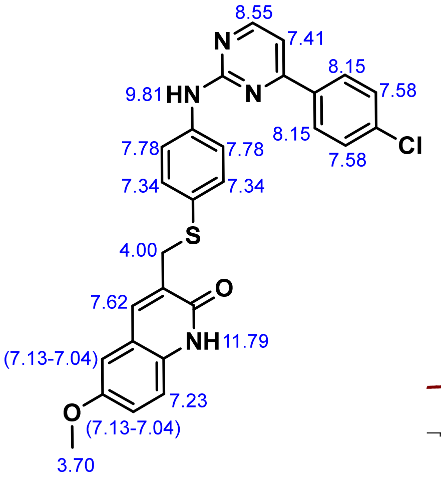

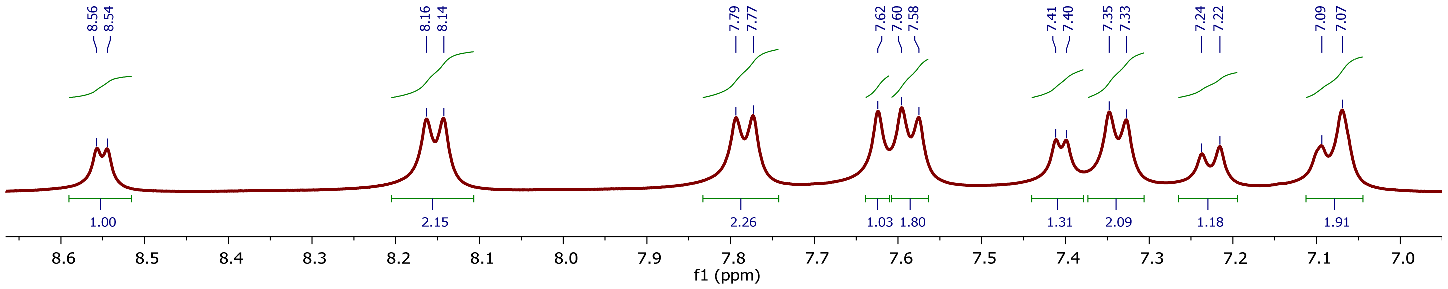

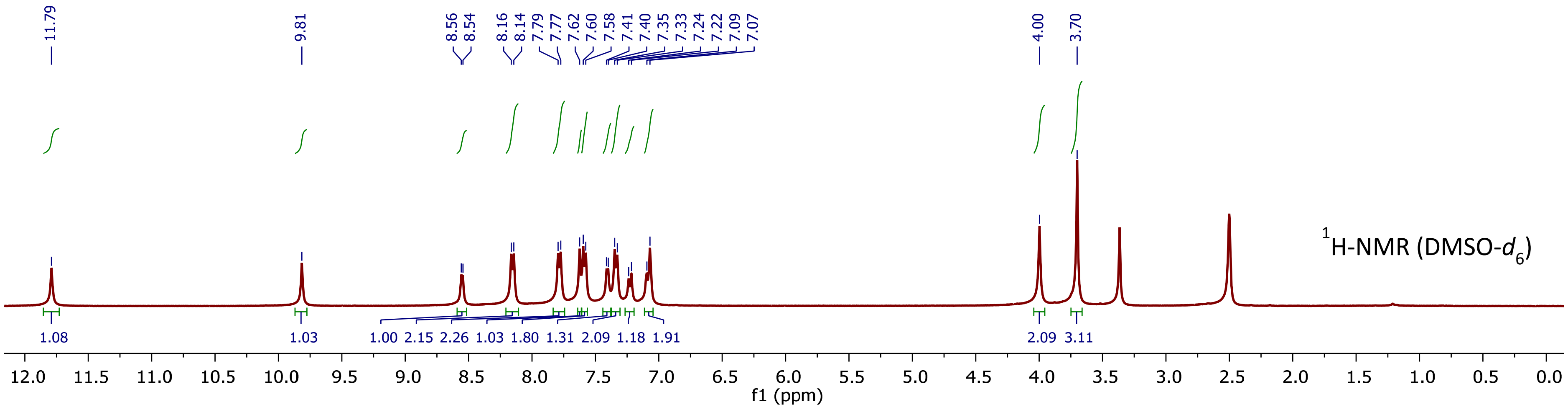

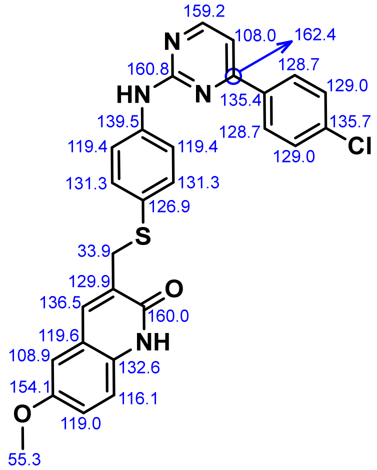
159.2
108.0
162.4
128.7
129.0
160.8
HN
N
135.4
135.7
Cl
139.5
119.4
119.4
128.7
129.0
131.3
131.3
126.9
S
33.9
129.9
O
136.5
160.0
119.6
NH
108.9
132.6
154.1
116.1
O
119.0
55.3

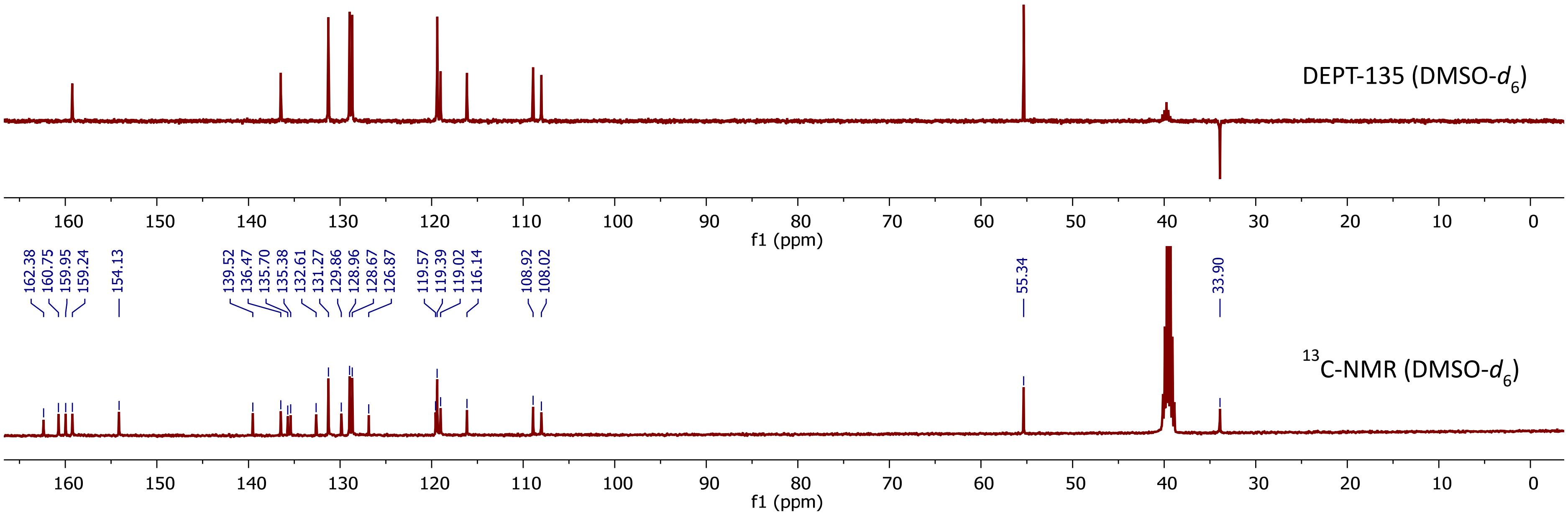
DEPT-135 (DMSO-d6)
f1 (ppm)
162.38
160.75
159.95
159.24
154.13
139.52
136.47
135.70
135.38
132.61
131.27
129.86
128.96
128.67
126.87
119.57
119.39
119.02
116.14
108.92
108.02
55.34
33.90
13C-NMR (DMSO-d6)
f1 (ppm)

EI MS (70eV)

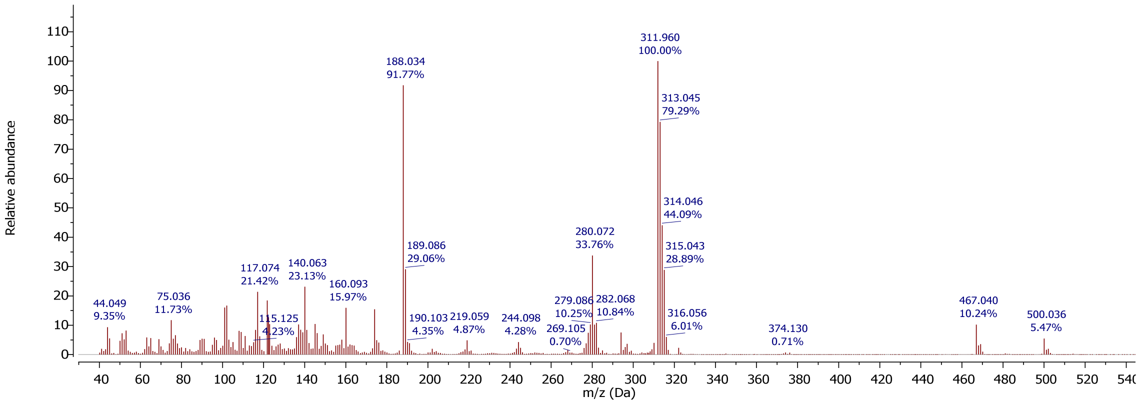

ESI-QTOF (positive ionization)

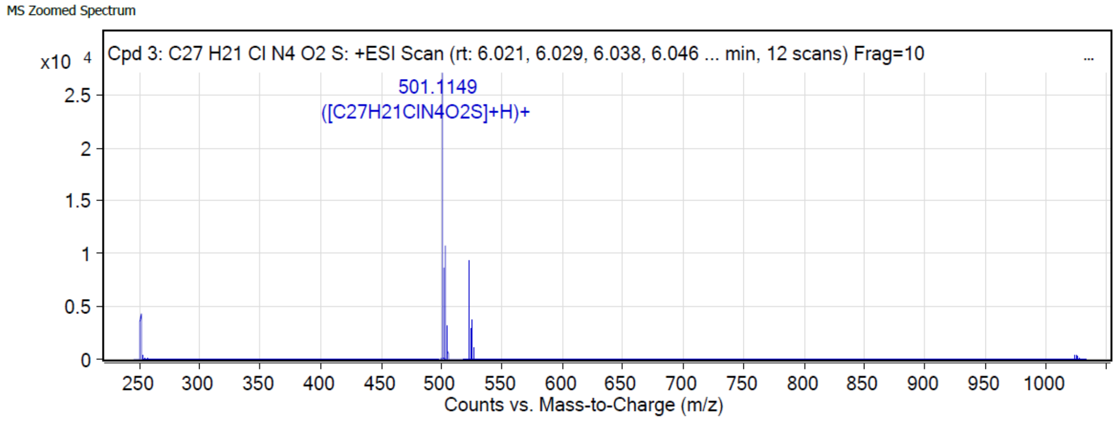

MS Spectrum Peak List

m/z	Calc *m/z*	Diff(ppm)	z	Abund	Formula	Ion
251.0607	251.061	0.87	2	4426.26	C27H21ClN4O2S	(M+2H)+2
252.0598	252.06	0.68	2	1891.7	C27H21ClN4O2S	(M+2H)+2
501.1149	501.1147	-0.58	1	27915.04	C27H21ClN4O2S	(M+H)+
502.1182	502.1176	-1.03	1	8698.28	C27H21ClN4O2S	(M+H)+
503.113	503.1127	-0.71	1	10863.01	C27H21ClN4O2S	(M+H)+
504.1153	504.115	-0.68	1	3341.83	C27H21ClN4O2S	(M+H)+
523.0968	523.0966	-0.44	1	9575.11	C27H21ClN4O2S	(M+Na)+
524.0994	524.0996	0.33	1	3051.01	C27H21ClN4O2S	(M+Na)+
525.0949	525.0946	-0.46	1	3856.37	C27H21ClN4O2S	(M+Na)+
1023.2084	1023.204	-4.34	1	341.57	C27H21ClN4O2S	(2M+Na)+

4.26 Compound 29a

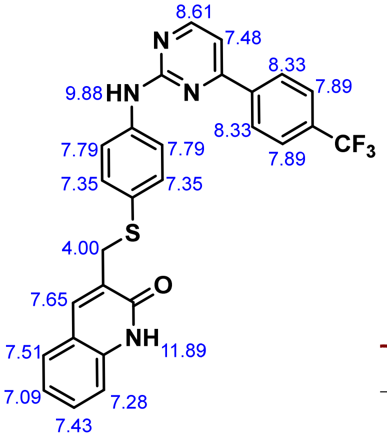

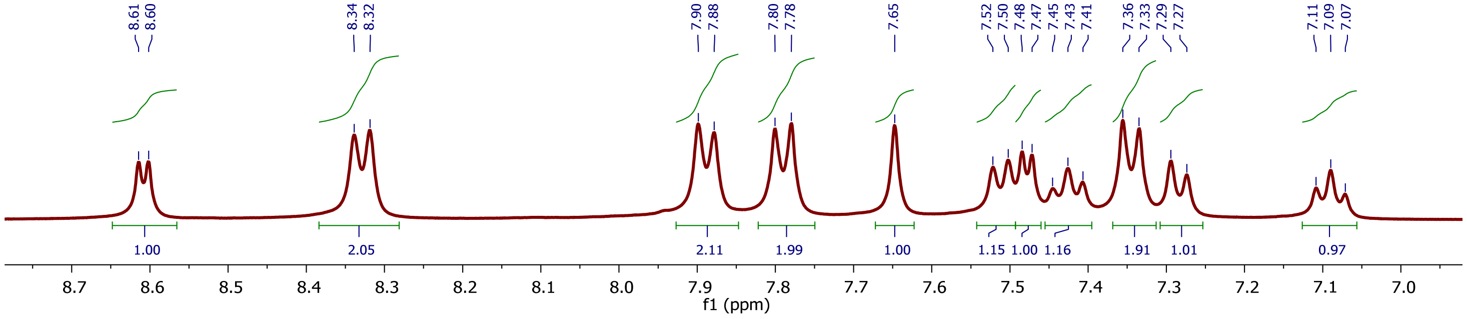

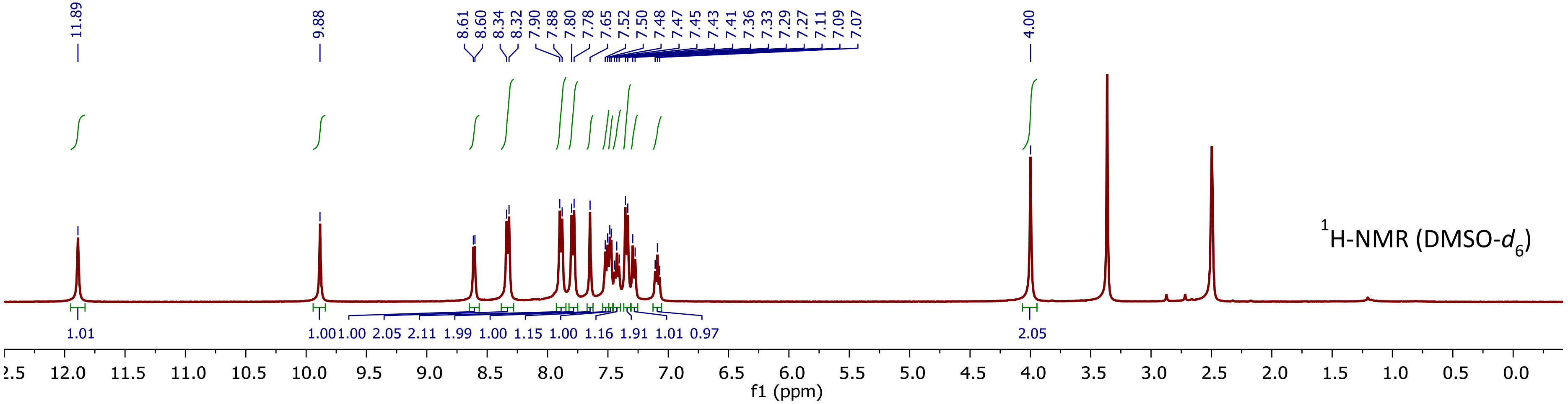

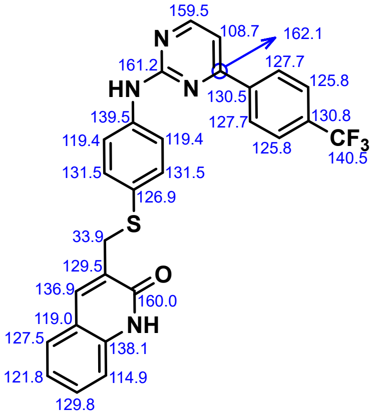
159.5
108.7
162.1
N
161.2
127.7
HN
N
130.5
125.8
139.5
127.7
130.8
119.4
119.4
CF_3
125.8
140.5
131.5
131.5
126.9
S
33.9
129.5
O
136.9
160.0
119.0
NH
127.5
138.1
121.8
114.9
129.8

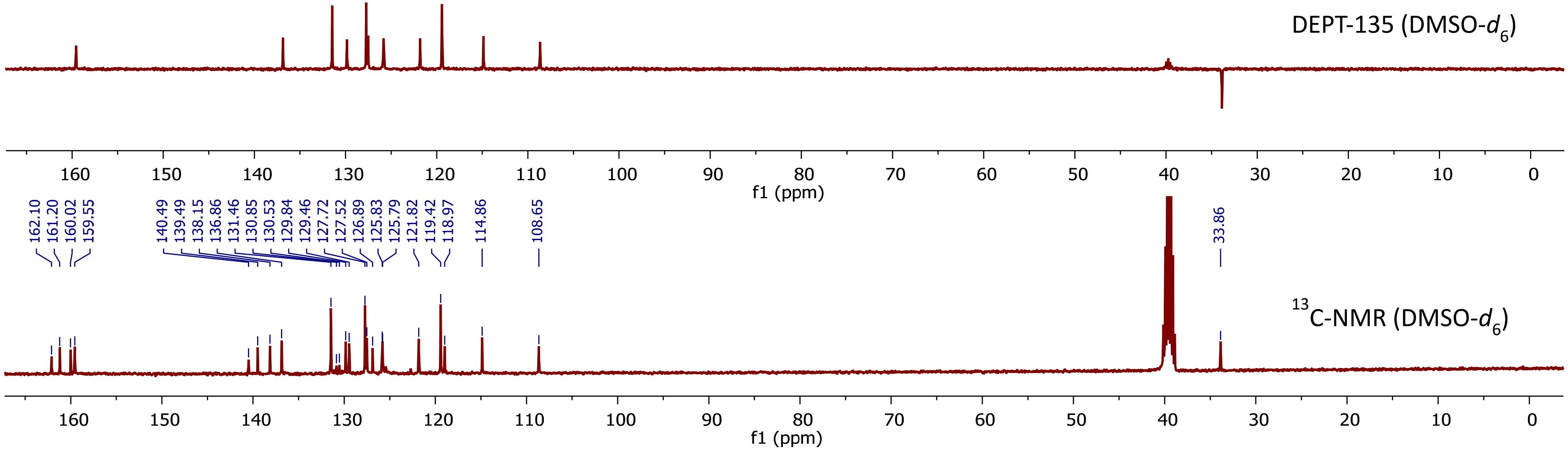
DEPT-135 (DMSO-d_6)
160 150 140 130 120 110 100 90 80 70 60 50 40 30 20 10 0
f1 (ppm)
162.10
161.20
160.02
159.55
140.49
139.49
138.15
136.86
131.46
130.85
130.53
129.84
129.46
127.72
127.52
126.89
125.83
125.79
121.82
119.42
118.97
114.86
108.65
33.86
^{13}C-NMR (DMSO-d_6)
160 150 140 130 120 110 100 90 80 70 60 50 40 30 20 10 0
f1 (ppm)

EI MS (70eV)

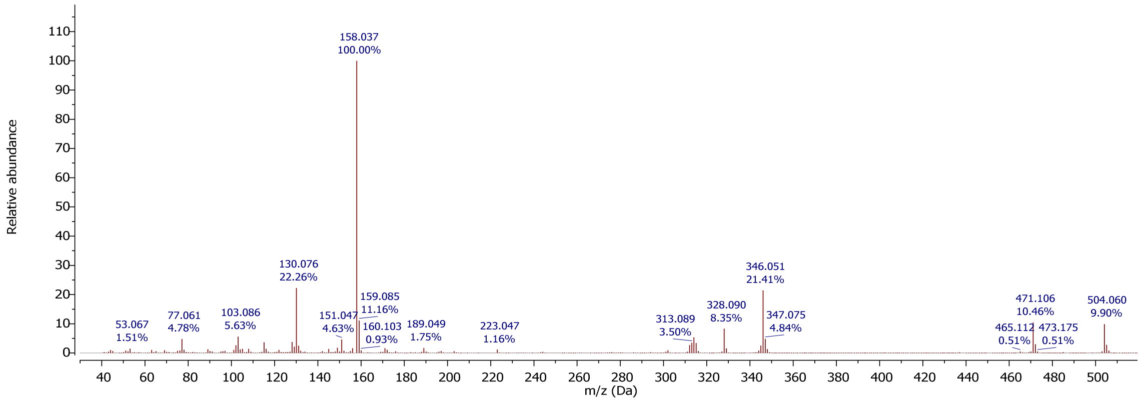

ESI-QTOF (positive ionization)

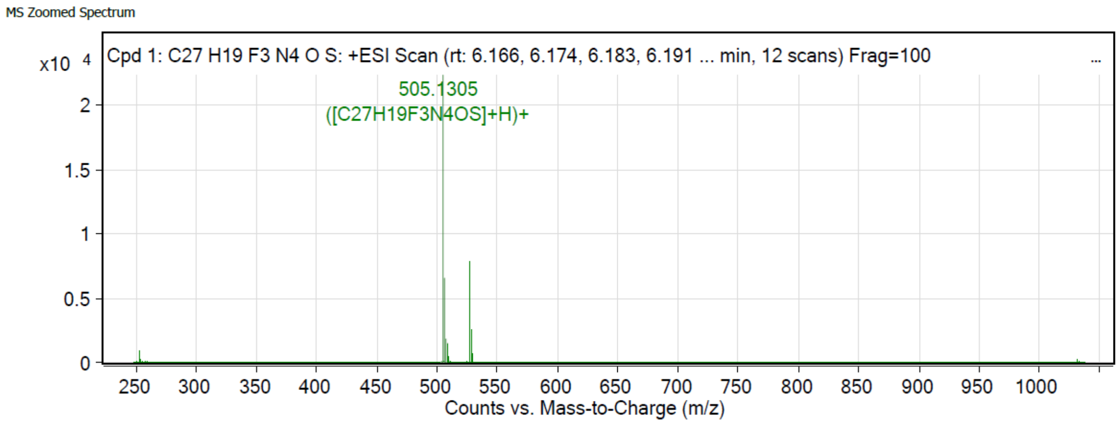

MS Spectrum Peak List

m/z	Calc *m/z*	Diff(ppm)	z	Abund	Formula	Ion
253.0686	253.0689	0.93	2	999.09	C27H19F3N4OS	(M+2H)+2
253.5731	253.5704	-10.91	2	251.84	C27H19F3N4OS	(M+2H)+2
505.1305	505.1304	-0.15	1	22389.96	C27H19F3N4OS	(M+H)+
506.1334	506.1334	0.06	1	6679.36	C27H19F3N4OS	(M+H)+
507.132	507.1316	-0.69	1	1904.2	C27H19F3N4OS	(M+H)+
508.1332	508.1321	-2.15	1	355.41	C27H19F3N4OS	(M+H)+
527.1125	527.1124	-0.2	1	7999.77	C27H19F3N4OS	(M+Na)+
528.1149	528.1154	0.85	1	2662.99	C27H19F3N4OS	(M+Na)+
529.113	529.1135	0.99	1	829.77	C27H19F3N4OS	(M+Na)+
1031.2355	1031.2356	0.09	1	264.18	C27H19F3N4OS	(2M+Na)+

4.27 Compound 29b

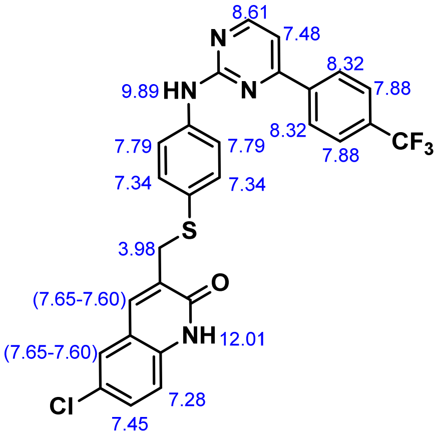

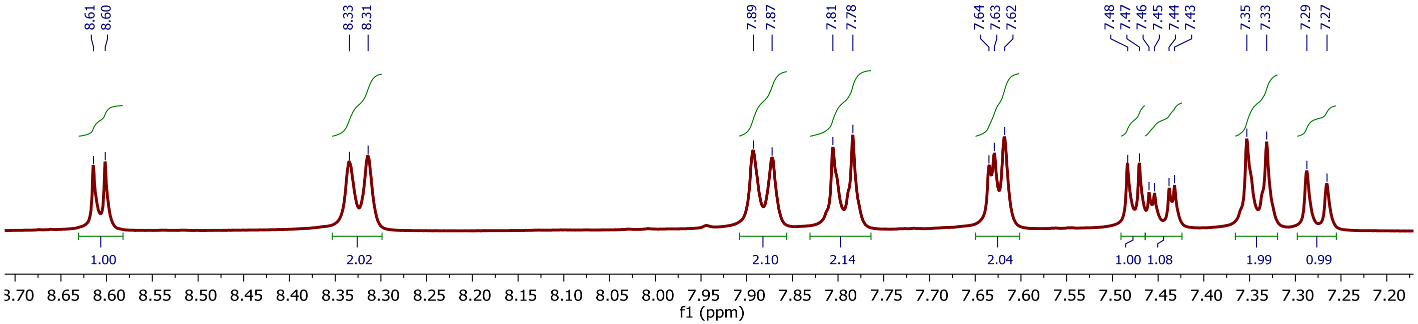

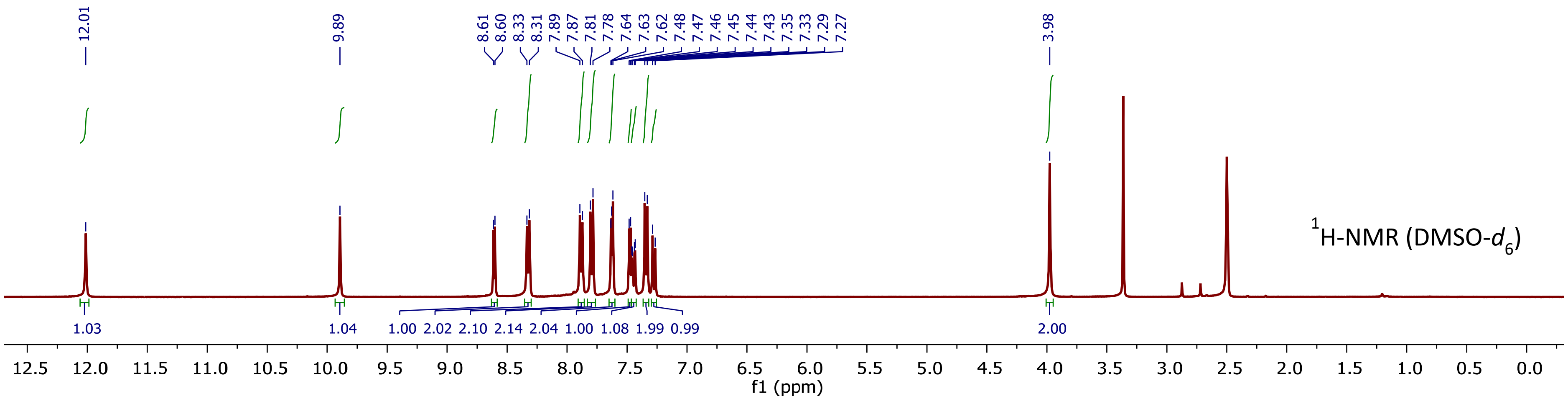

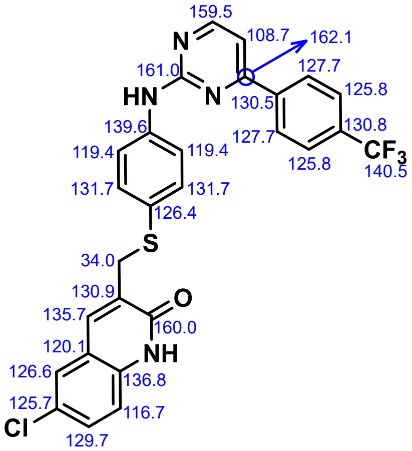
159.5
108.7
162.1
N
161.0
127.7
125.8
HN
N
130.5
130.8
139.6
127.7
CF3
119.4
119.4
125.8
140.5
131.7
131.7
126.4
34.0
S
130.9
O
135.7
160.0
120.1
NH
126.6
136.8
125.7
116.7
Cl
129.7

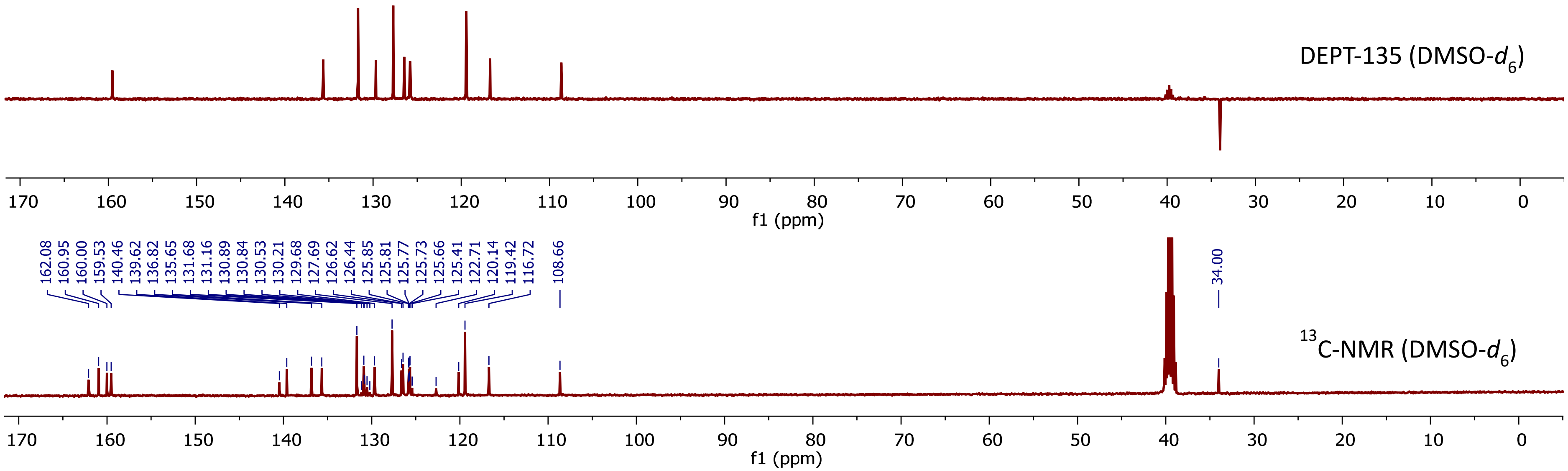
DEPT-135 (DMSO-d6)
170 160 150 140 130 120 110 100 90 80 70 60 50 40 30 20 10 0
f1 (ppm)
162.08 160.95 160.00 159.53 140.46 139.62 136.82 135.65 131.68 131.16 130.89 130.84 130.53 130.21 129.68 127.69 126.62 126.44 125.85 125.81 125.77 125.73 125.66 125.41 122.71 120.14 119.42 116.72 108.66 34.00
13C-NMR (DMSO-d6)
170 160 150 140 130 120 110 100 90 80 70 60 50 40 30 20 10 0
f1 (ppm)

EI MS (70eV)

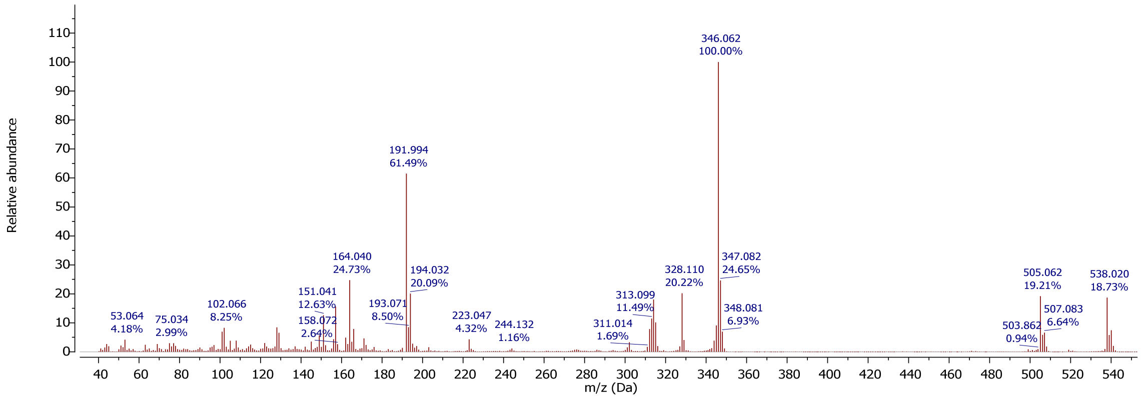

ESI-QTOF (positive ionization)

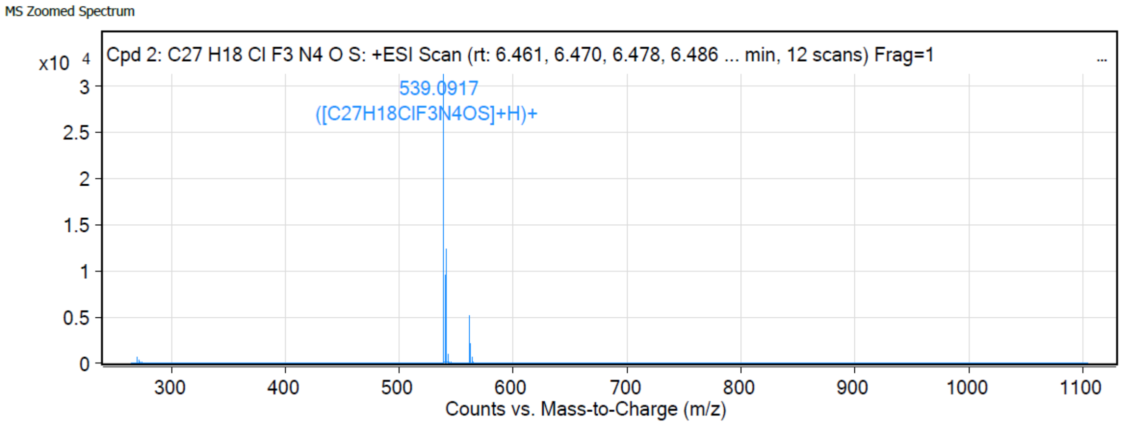

MS Spectrum Peak List

m/z	Calc *m/z*	Diff(ppm)	z	Abund	Formula	Ion
270.0488	270.0494	1.95	2	644.48	C27H18ClF3N4OS	(M+2H)+2
539.0917	539.0915	-0.46	1	31643.5	C27H18ClF3N4OS	(M+H)+
540.0945	540.0945	-0.16	1	9793.26	C27H18ClF3N4OS	(M+H)+
541.0895	541.0895	-0.09	1	12503.29	C27H18ClF3N4OS	(M+H)+
542.0922	542.0918	-0.74	1	3917.52	C27H18ClF3N4OS	(M+H)+
543.0892	543.0901	1.66	1	997.55	C27H18ClF3N4OS	(M+H)+
561.0737	561.0734	-0.48	1	5293.78	C27H18ClF3N4OS	(M+Na)+
562.077	562.0764	-1.14	1	1791.7	C27H18ClF3N4OS	(M+Na)+
563.0708	563.0714	1.05	1	2218.61	C27H18ClF3N4OS	(M+Na)+
1099.1553	1099.1576	2.07	1	60.47	C27H18ClF3N4OS	(2M+Na)+

4.28 Compound 29c

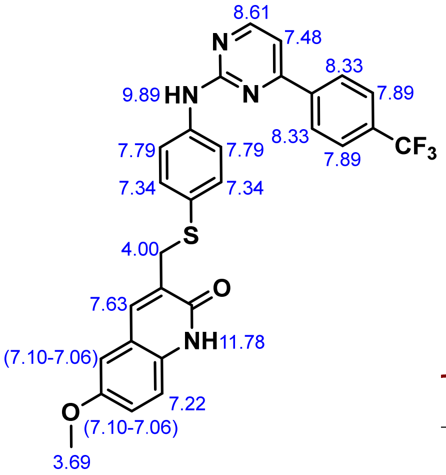

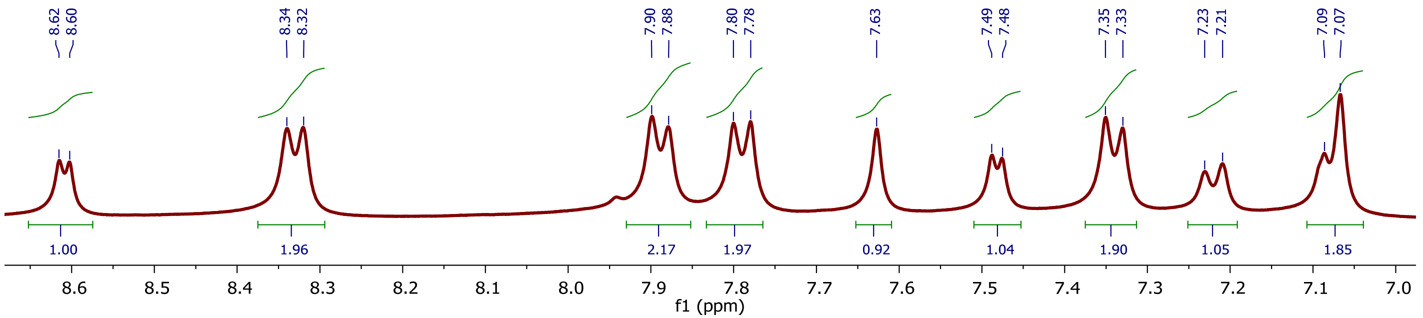

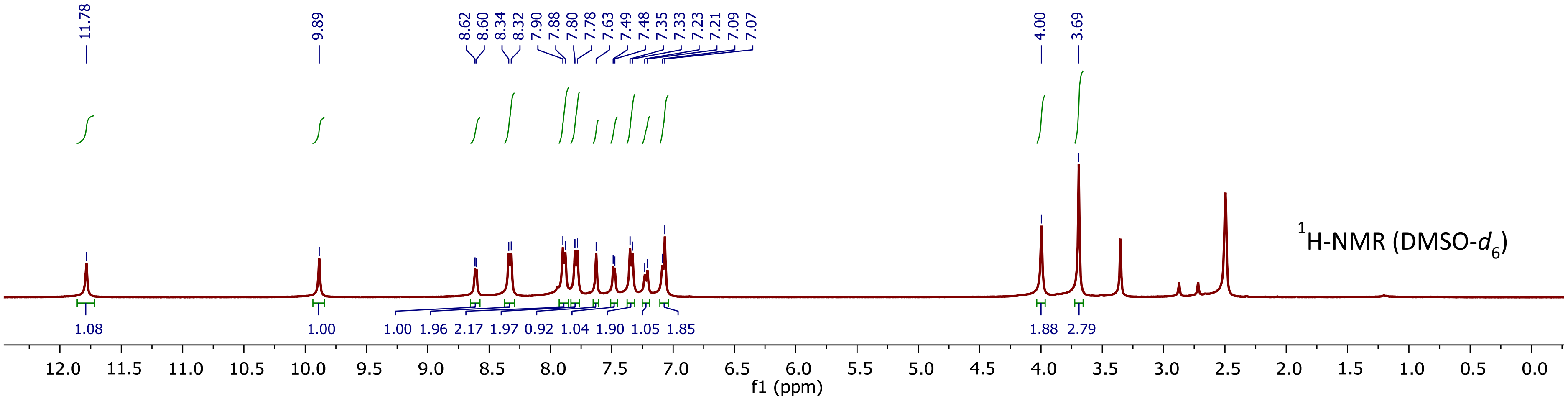

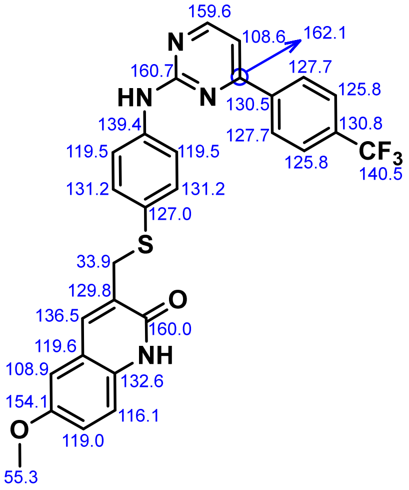
159.6
108.6
162.1
127.7
125.8
160.7
HN
N
N
130.5
130.8
139.4
127.7
CF3
119.5
119.5
125.8
140.5
131.2
131.2
127.0
S
33.9
129.8
O
136.5
160.0
119.6
NH
108.9
132.6
154.1
116.1
O
119.0
55.3

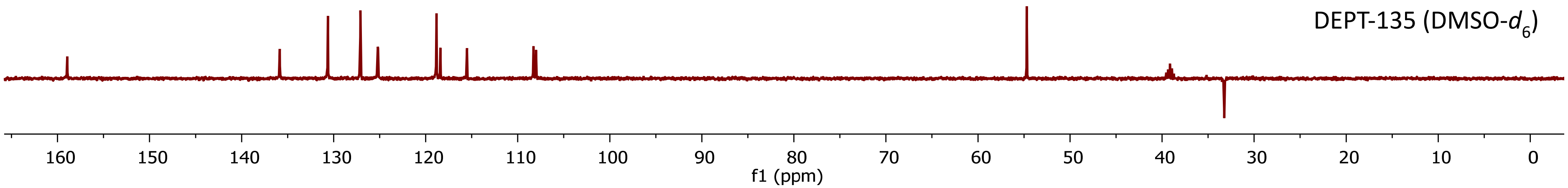
DEPT-135 (DMSO-d6)
160 150 140 130 120 110 100 90 80 70 60 50 40 30 20 10 0
f1 (ppm)

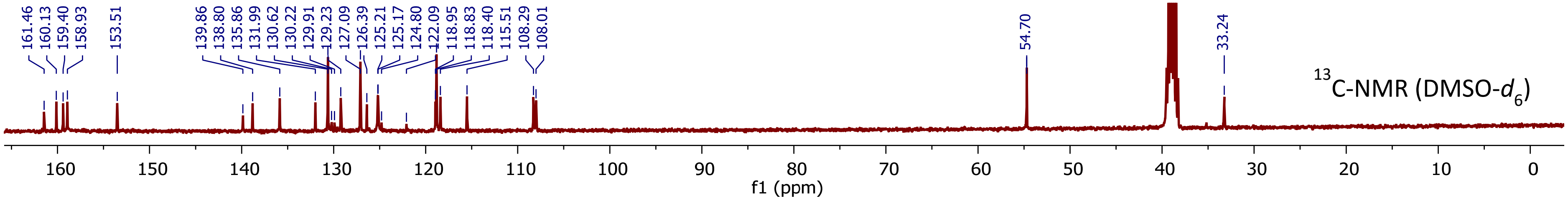
161.46
160.13
159.40
158.93
153.51
139.86
138.80
135.86
131.99
130.62
130.22
129.91
129.23
127.09
126.39
125.21
125.17
124.80
122.09
118.95
118.83
118.40
115.51
108.29
108.01
54.70
33.24
13C-NMR (DMSO-d6)
160 150 140 130 120 110 100 90 80 70 60 50 40 30 20 10 0
f1 (ppm)

EI MS (70eV)

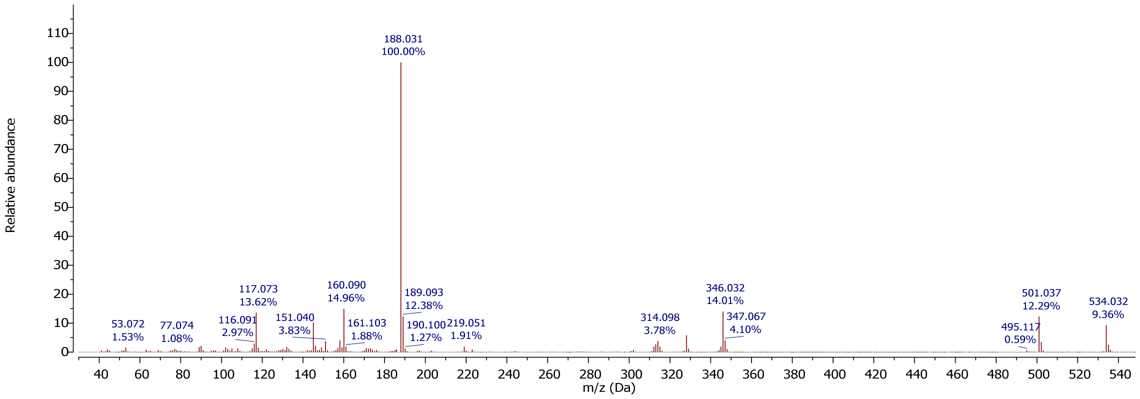

ESI-QTOF (positive ionization)

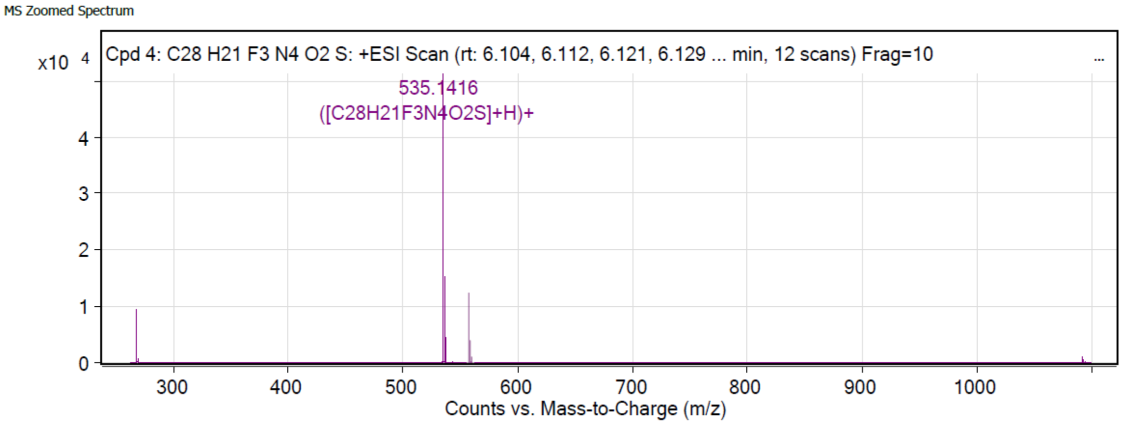

MS Spectrum Peak List

m/z	Calc *m/z*	Diff(ppm)	z	Abund	Formula	Ion
268.0741	268.0741	0.27	2	9462.26	C28H21F3N4O2S	(M+2H)+2
268.5756	268.5756	0.15	2	3198.57	C28H21F3N4O2S	(M+2H)+2
269.0752	269.0748	-1.14	2	934.05	C28H21F3N4O2S	(M+2H)+2
535.1416	535.141	-1.04	1	51348.98	C28H21F3N4O2S	(M+H)+
536.1445	536.144	-0.92	1	16004.3	C28H21F3N4O2S	(M+H)+
537.1433	537.1424	-1.55	1	4650.11	C28H21F3N4O2S	(M+H)+
557.1231	557.123	-0.22	1	12574.94	C28H21F3N4O2S	(M+Na)+
558.1263	558.126	-0.7	1	4061.59	C28H21F3N4O2S	(M+Na)+
559.1251	559.1244	-1.35	1	1216.16	C28H21F3N4O2S	(M+Na)+
1091.2563	1091.2567	0.31	1	1051.75	C28H21F3N4O2S	(2M+Na)+

4.29 Compound 30a

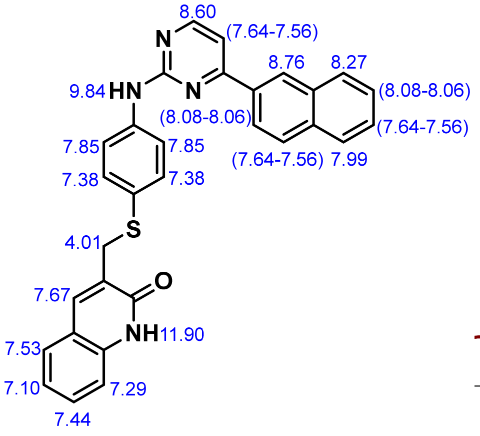

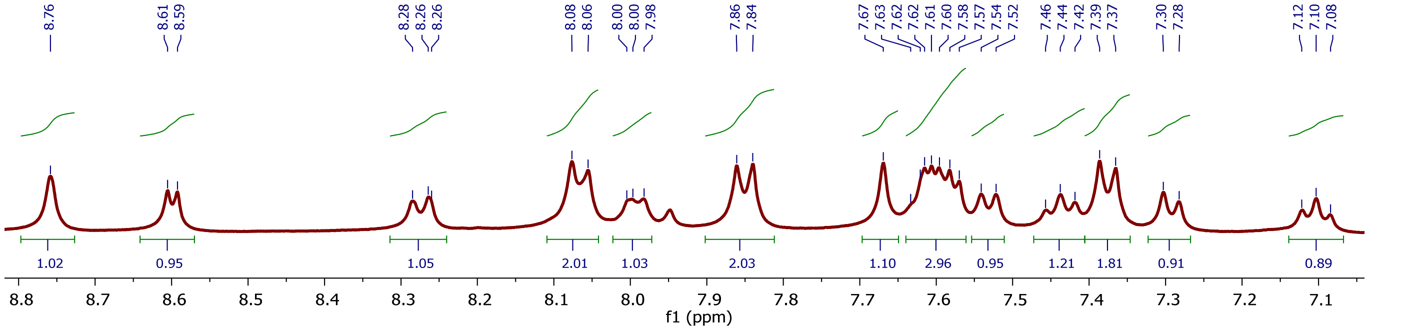

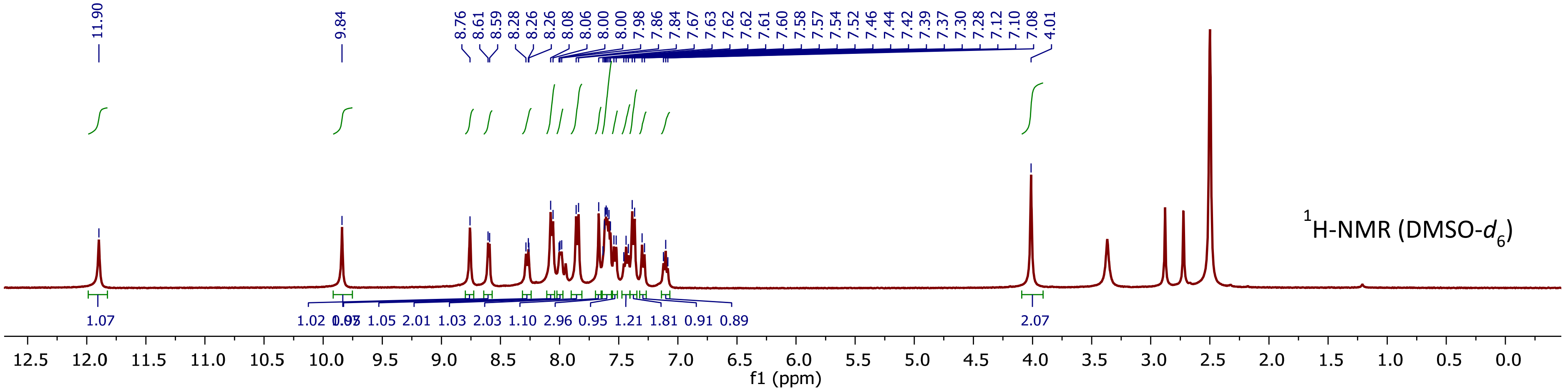

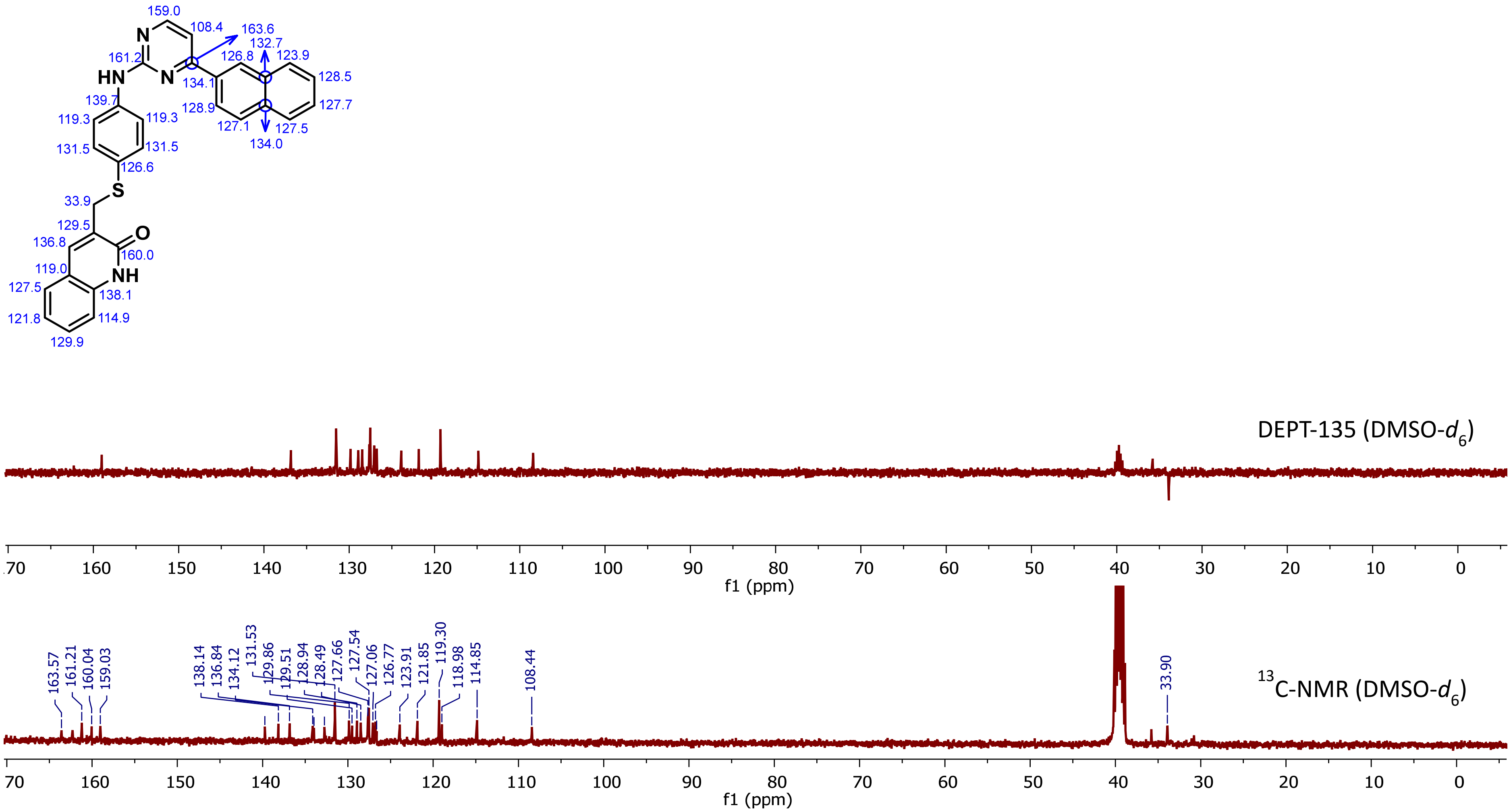
DEPT-135 (DMSO-d6)
f1 (ppm)
13C-NMR (DMSO-d6)
163.57
161.21
160.04
159.03
138.14
136.84
134.12
131.53
129.86
129.51
128.94
128.49
127.66
127.54
127.06
126.77
123.91
121.85
119.30
118.98
114.85
108.44
33.90
f1 (ppm)

EI MS (70eV)

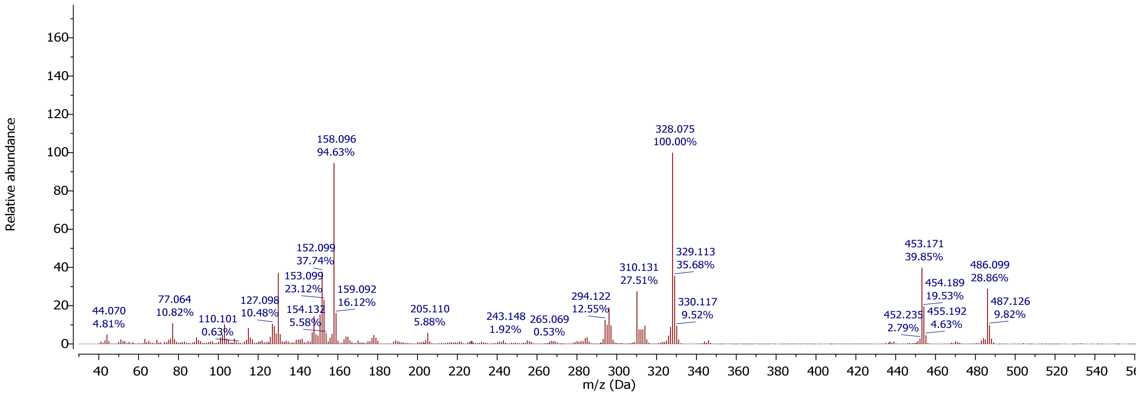

ESI-QTOF (positive ionization)

MS Zoomed Spectrum

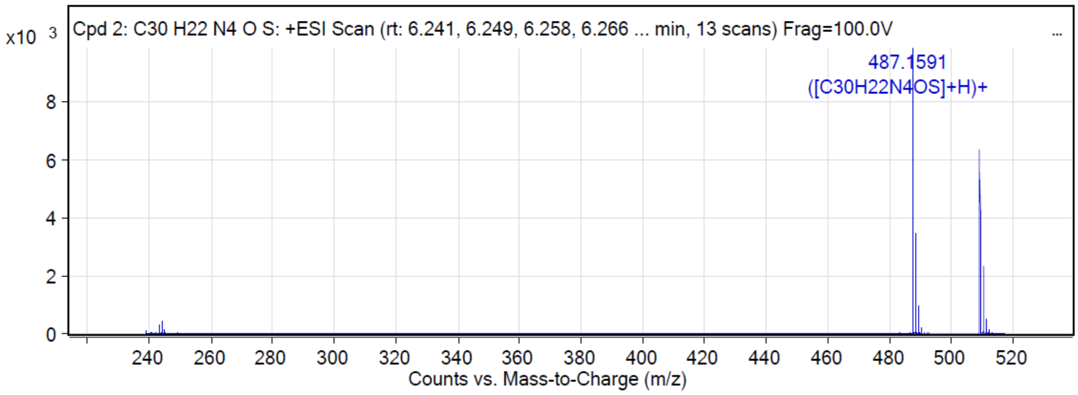

MS Spectrum Peak List

m/z	Calc *m/z*	Diff(ppm)	z	Abund	Formula	Ion
244.0825	244.083	2	2	497.6	C30H22N4OS	(M+2H)+2
244.5853	244.5845	-3.32	2	121.62	C30H22N4OS	(M+2H)+2
245.0794	245.0838	18.26	2	54.57	C30H22N4OS	(M+2H)+2
487.1591	487.1587	-0.73	1	9911.09	C30H22N4OS	(M+H)+
488.1618	488.1617	-0.18	1	3507.15	C30H22N4OS	(M+H)+
489.161	489.1604	-1.3	1	998.93	C30H22N4OS	(M+H)+
509.1411	509.1407	-0.95	1	6441.07	C30H22N4OS	(M+Na)+
510.1432	510.1437	1	1	2417.24	C30H22N4OS	(M+Na)+
511.1432	511.1423	-1.77	1	575.29	C30H22N4OS	(M+Na)+
512.1463	512.1428	-6.91	1	102.76	C30H22N4OS	(M+Na)+

4.30 Compound 30b

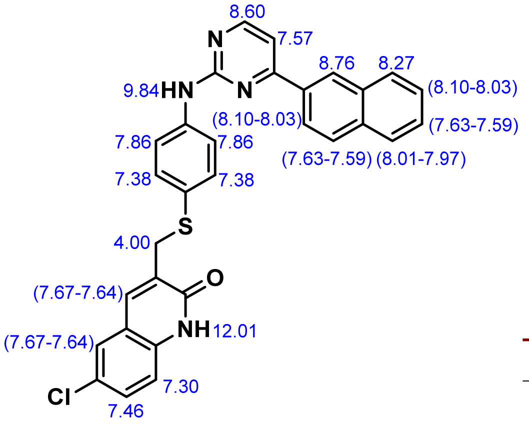

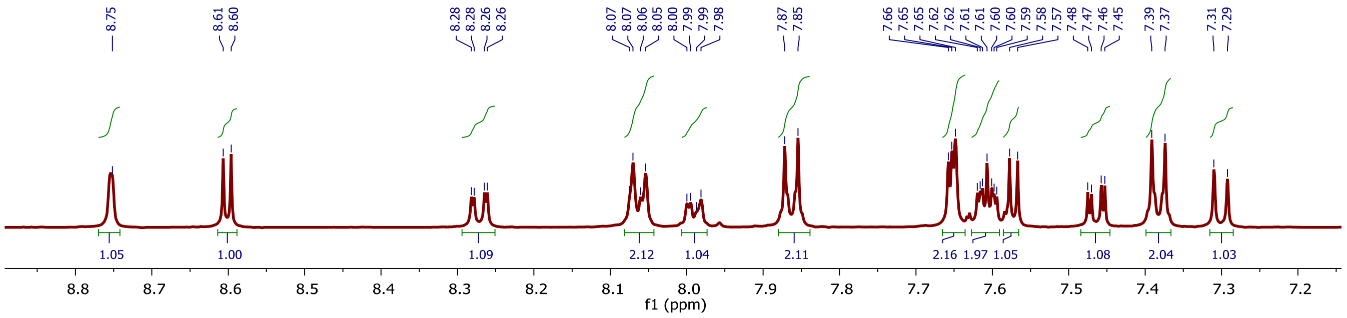

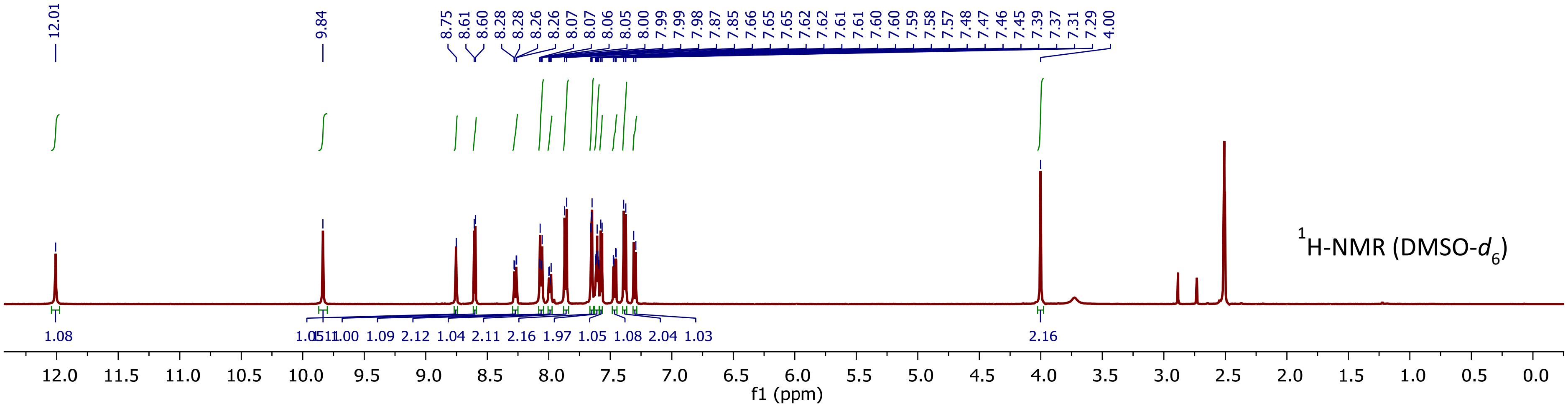

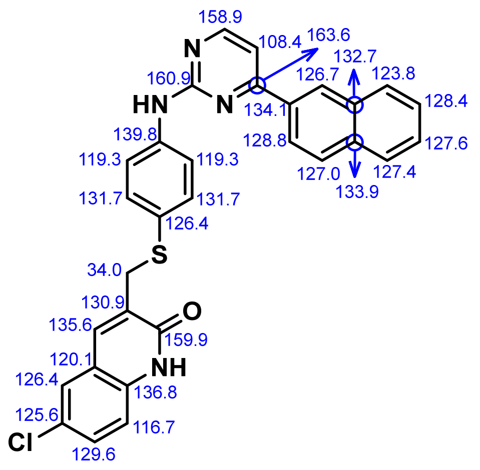

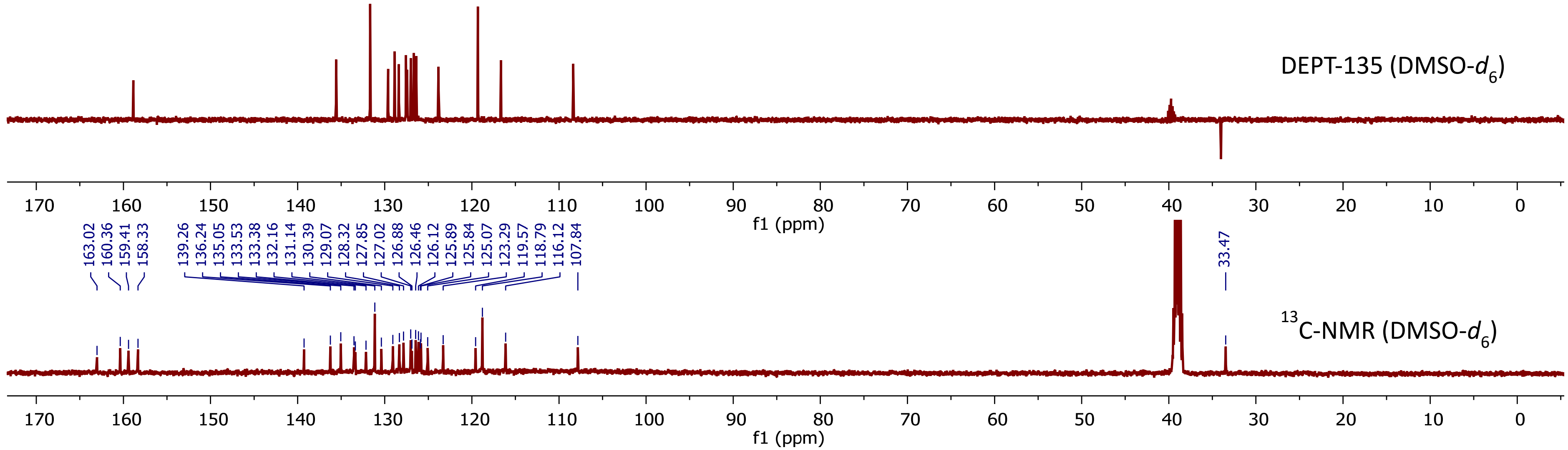

DEPT-135 (DMSO-d_6)
f1 (ppm)
163.02 160.36 159.41 158.33 139.26 136.24 135.05 133.53 133.38 132.16 131.14 130.39 129.07 128.32 127.85 127.02 126.88 126.46 126.12 125.89 125.84 125.07 123.29 119.57 118.79 116.12 107.84 33.47
^{13}C-NMR (DMSO-d_6)
f1 (ppm)

EI MS (70eV)

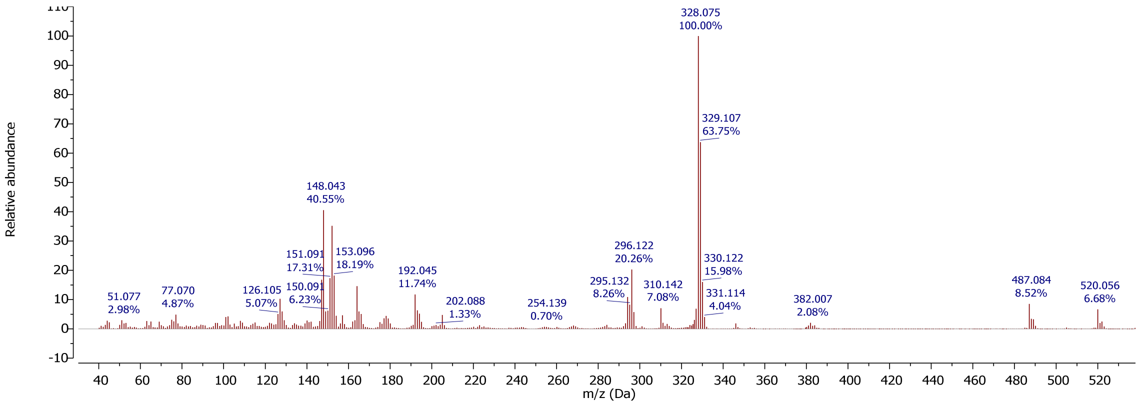

ESI-QTOF (positive ionization)

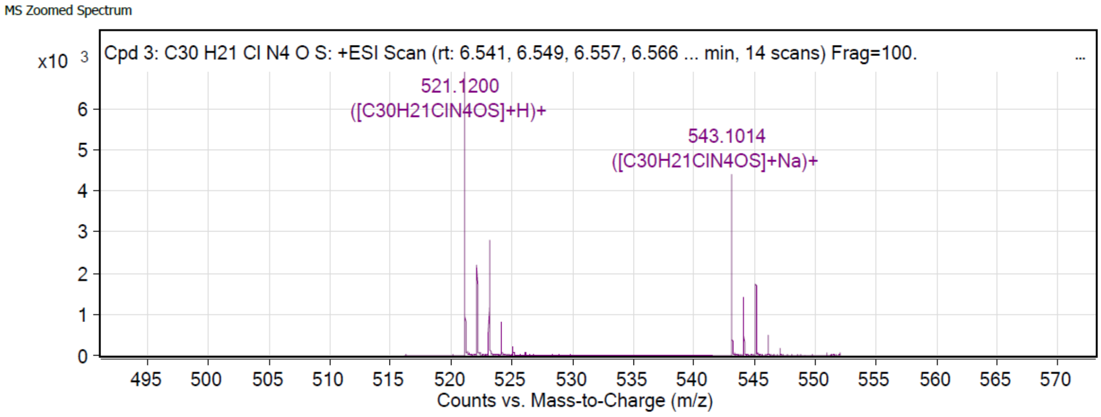

MS Spectrum Peak List

m/z	Calc *m/z*	Diff(ppm)	z	Abund	Formula	Ion
521.12	521.1197	-0.43	1	6953.13	C30H21ClN4OS	(M+H)+
522.1225	522.1228	0.41	1	2321.15	C30H21ClN4OS	(M+H)+
523.1184	523.1179	-0.9	1	2869.15	C30H21ClN4OS	(M+H)+
524.1206	524.1201	-0.91	1	840.5	C30H21ClN4OS	(M+H)+
525.1218	525.1189	-5.56	1	235.49	C30H21ClN4OS	(M+H)+
543.1014	543.1017	0.59	1	4442.43	C30H21ClN4OS	(M+Na)+
544.1049	544.1047	-0.44	1	1485.43	C30H21ClN4OS	(M+Na)+
545.0992	545.0999	1.25	1	1813.92	C30H21ClN4OS	(M+Na)+
546.1036	546.1021	-2.75	1	545.87	C30H21ClN4OS	(M+Na)+
547.101	547.1009	-0.21	1	175.92	C30H21ClN4OS	(M+Na)+

4.31 Compound 30c

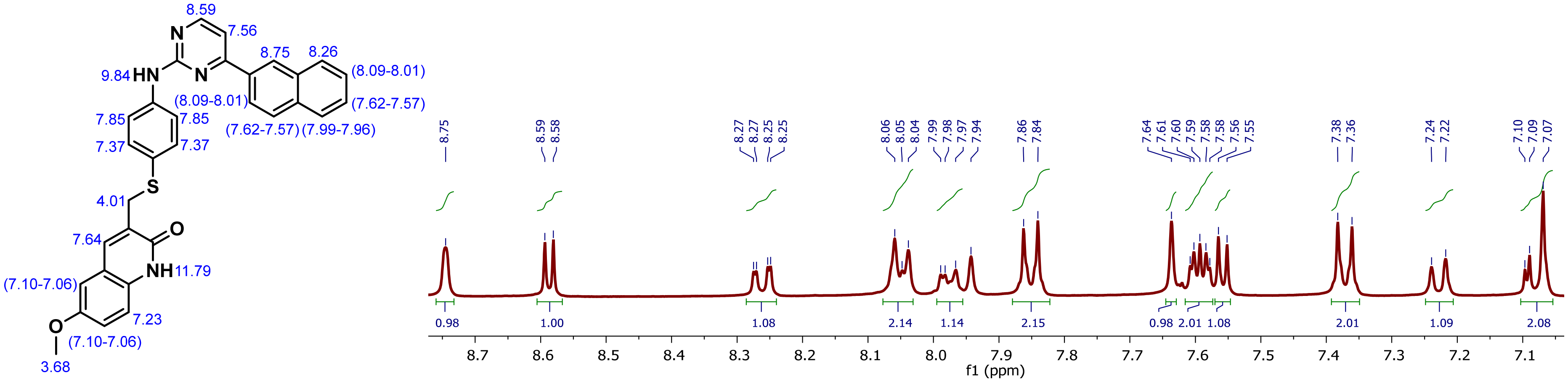

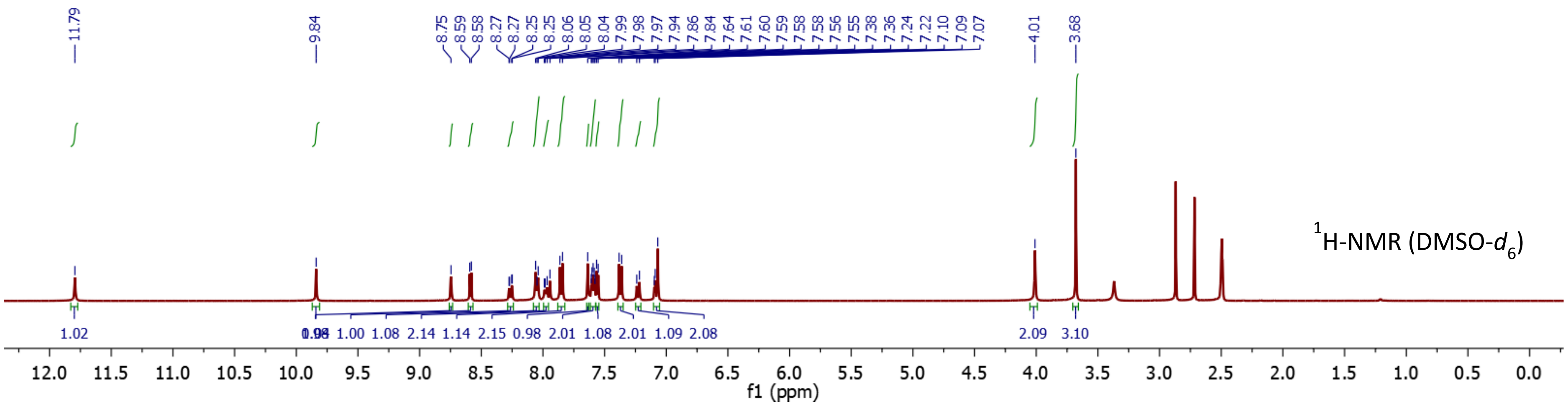

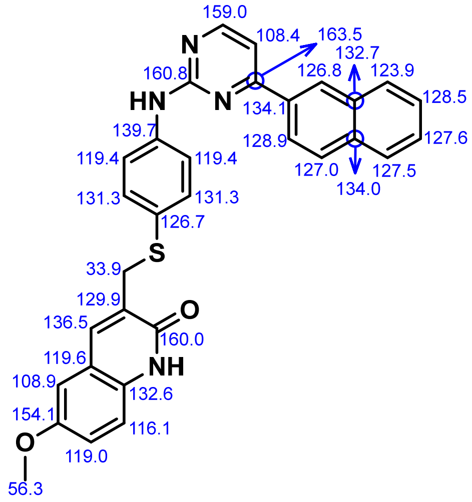
159.0
108.4
163.5
132.7
126.8
123.9
128.5
160.8
134.1
128.9
127.6
127.0
127.5
134.0
139.7
119.4
119.4
131.3
131.3
126.7
33.9
129.9
136.5
160.0
119.6
108.9
132.6
154.1
116.1
119.0
56.3

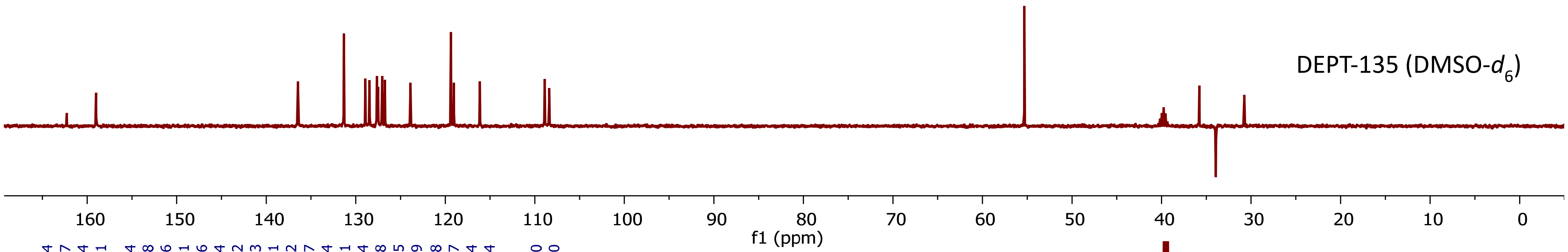
DEPT-135 (DMSO-d_6)
f1 (ppm)

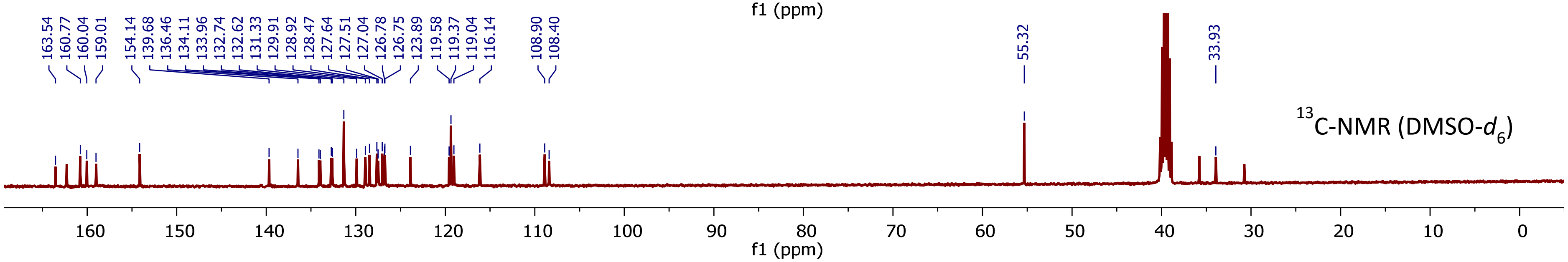
163.54
160.77
160.04
159.01
154.14
139.68
136.46
134.11
133.96
132.74
132.62
131.33
129.91
128.92
128.47
127.64
127.51
127.04
126.78
126.75
123.89
119.58
119.37
119.04
116.14
108.90
108.40
55.32
33.93
^{13}C-NMR (DMSO-d_6)
f1 (ppm)

EI MS (70eV)

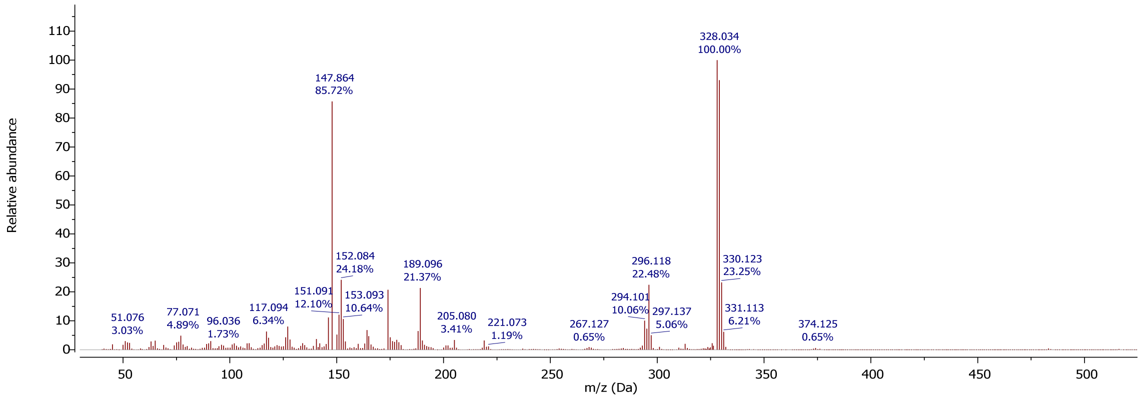

ESI-QTOF (positive ionization)

MS Zoomed Spectrum

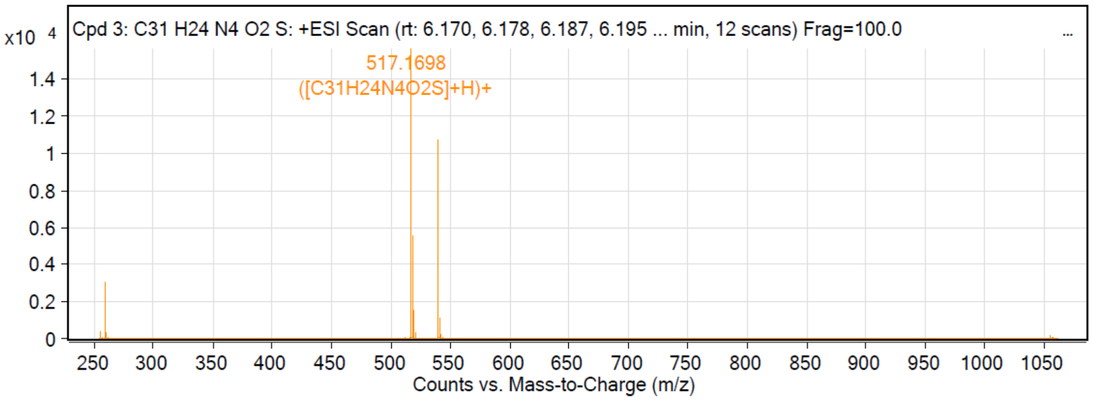

MS Spectrum Peak List

m/z	Calc *m/z*	Diff(ppm)	z	Abund	Formula	Ion
259.0884	259.0883	-0.48	2	3151.79	C31H24N4O2S	(M+2H)+2
259.5893	259.5898	1.98	2	1104.27	C31H24N4O2S	(M+2H)+2
260.0897	260.0892	-1.67	2	368.27	C31H24N4O2S	(M+2H)+2
517.1698	517.1693	-1.06	1	15760.97	C31H24N4O2S	(M+H)+
518.1728	518.1723	-0.94	1	5645.06	C31H24N4O2S	(M+H)+
519.1722	519.1712	-1.98	1	1609.85	C31H24N4O2S	(M+H)+
539.1517	539.1512	-0.93	1	11045.04	C31H24N4O2S	(M+Na)+
540.1551	540.1543	-1.52	1	3947.27	C31H24N4O2S	(M+Na)+
541.155	541.1531	-3.5	1	1184.69	C31H24N4O2S	(M+Na)+
1055.3142	1055.3132	-0.95	1	210.75	C31H24N4O2S	(2M+Na)+

4.32 Compound 31a

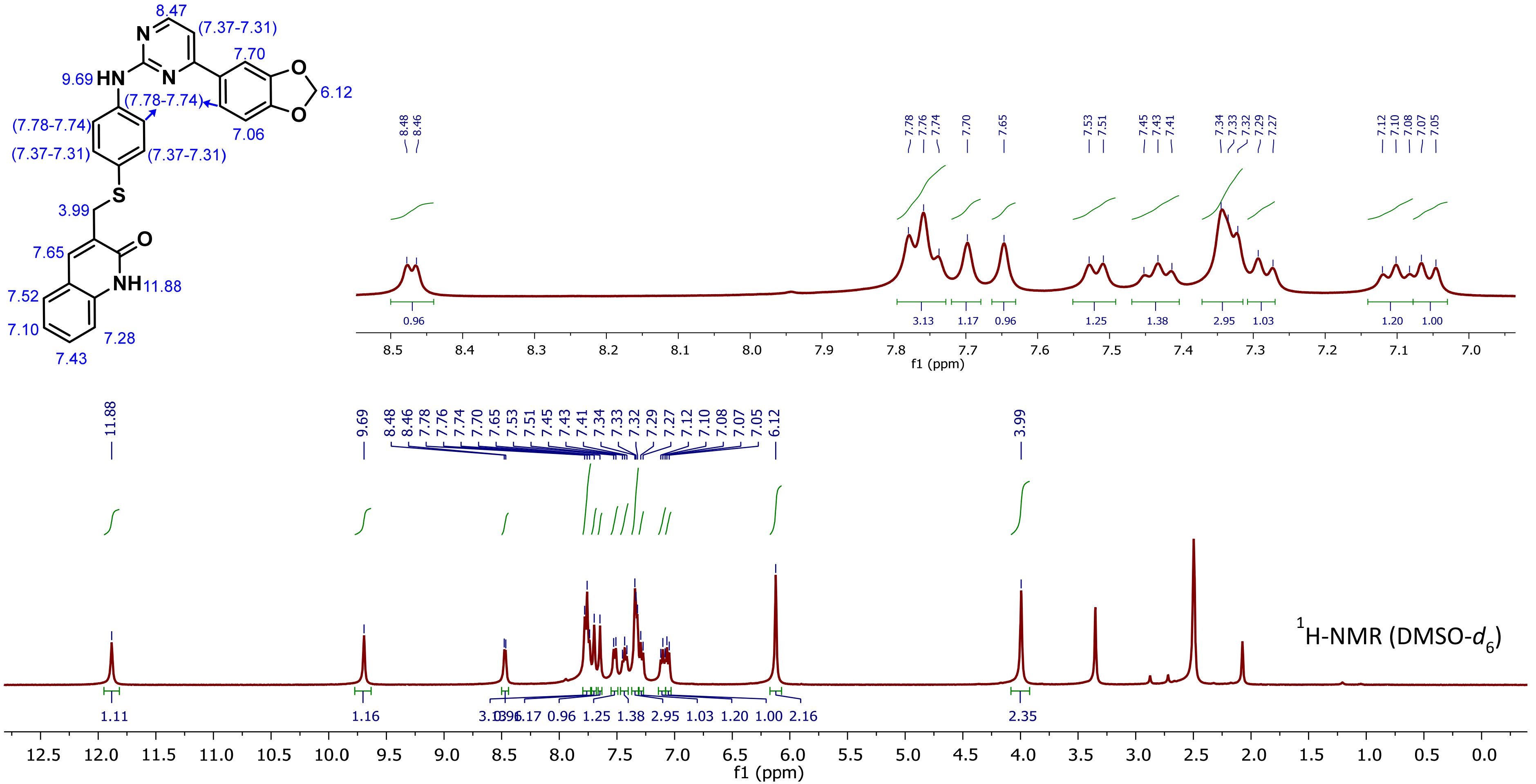

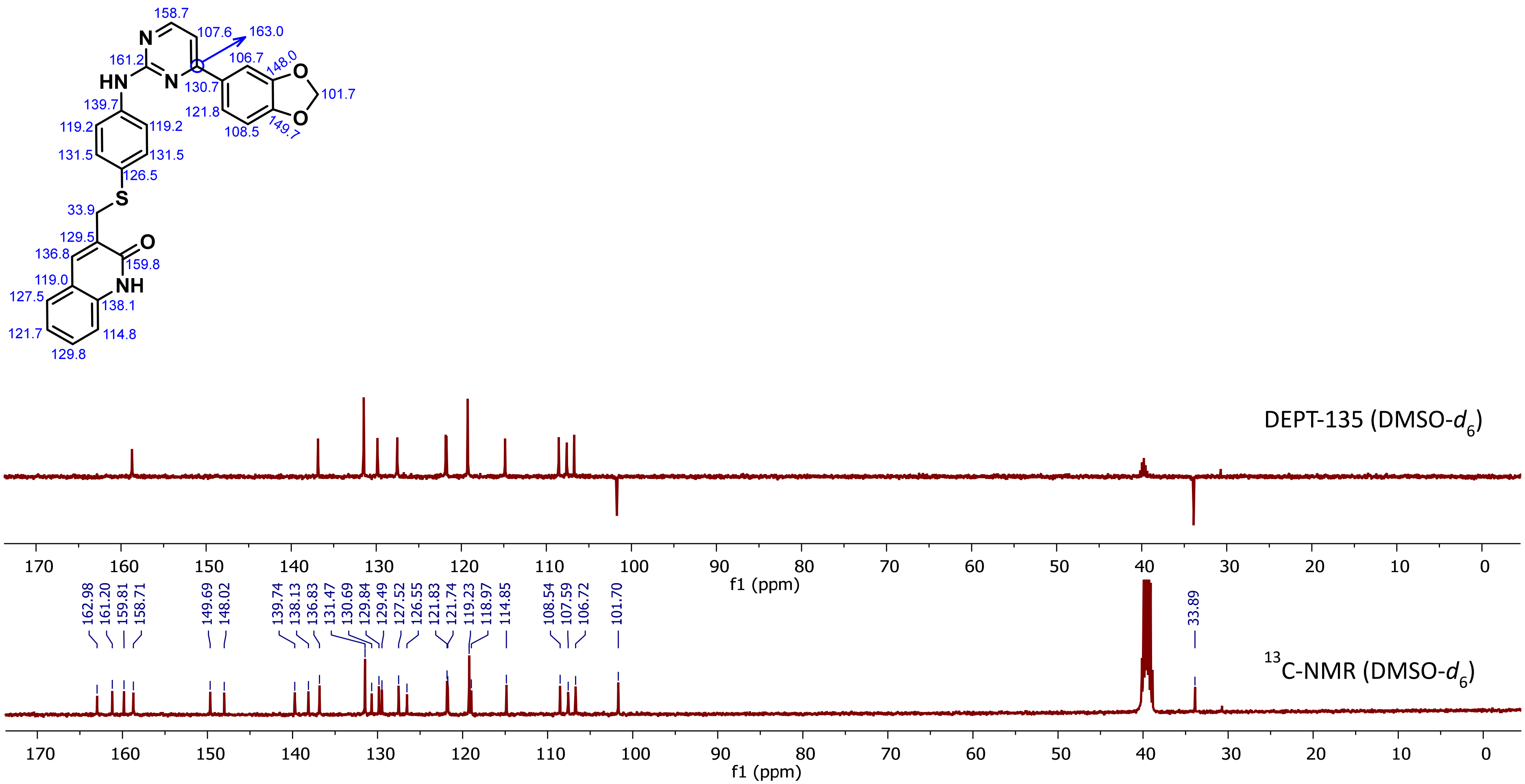
158.7
107.6
163.0
161.2
106.7
148.0
HN
130.7
101.7
139.7
121.8
119.2
119.2
108.5
149.7
131.5
131.5
126.5
33.9
S
129.5
O
136.8
159.8
119.0
NH
127.5
138.1
121.7
114.8
129.8
DEPT-135 (DMSO-d_6)
f1 (ppm)
162.98
161.20
159.81
158.71
149.69
148.02
139.74
138.13
136.83
131.47
130.69
129.84
129.49
127.52
126.55
121.83
121.74
119.23
118.97
114.85
108.54
107.59
106.72
101.70
33.89
^{13}C-NMR (DMSO-d_6)
f1 (ppm)

EI MS (70eV)

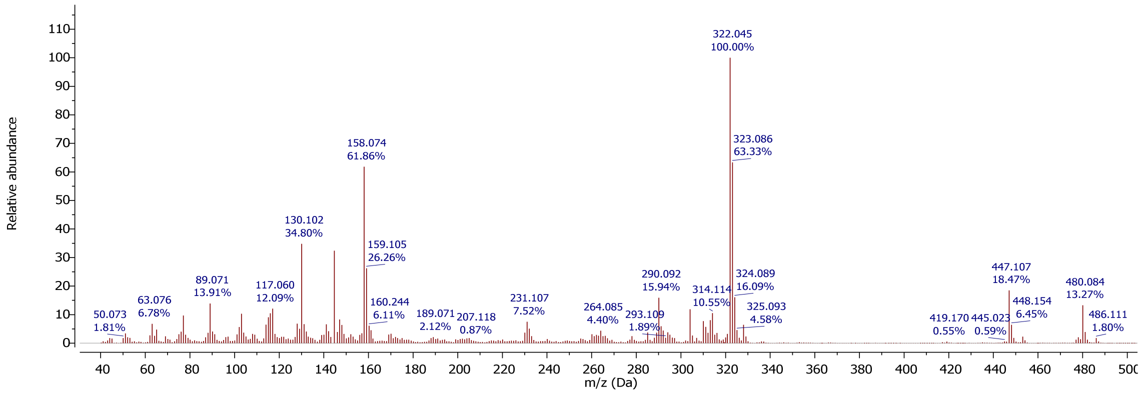

ESI-QTOF (positive ionization)

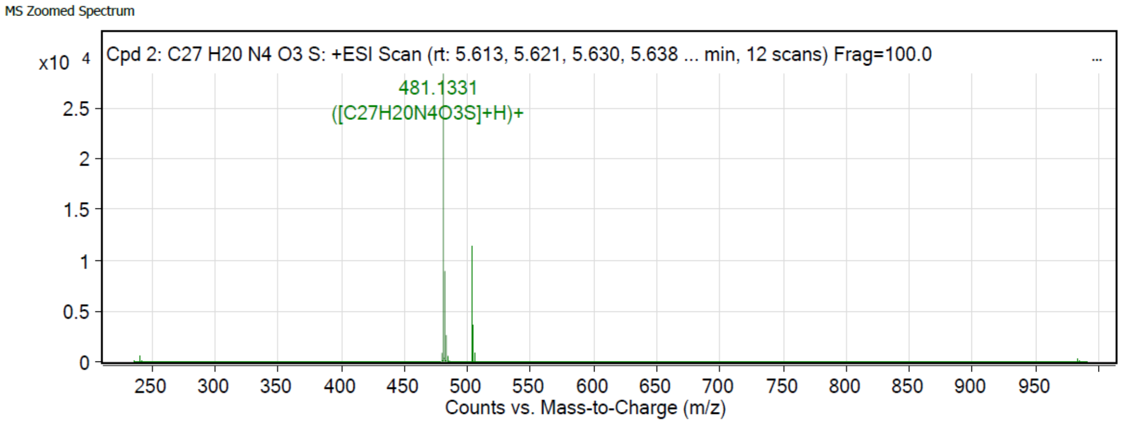

MS Spectrum Peak List

m/z	Calc *m/z*	Diff(ppm)	z	Abund	Formula	Ion
241.0696	241.0701	1.93	2	654.72	C27H20N4O3S	(M+2H)+2
481.1331	481.1329	-0.47	1	29280.21	C27H20N4O3S	(M+H)+
482.1361	482.1359	-0.51	1	8975.86	C27H20N4O3S	(M+H)+
483.1347	483.1342	-1.13	1	2638.49	C27H20N4O3S	(M+H)+
503.115	503.1148	-0.28	1	11622.33	C27H20N4O3S	(M+Na)+
504.1176	504.1178	0.4	1	3718.28	C27H20N4O3S	(M+Na)+
505.1169	505.1161	-1.49	1	1042.77	C27H20N4O3S	(M+Na)+
506.1125	506.1169	8.6	1	186.36	C27H20N4O3S	(M+Na)+
983.2422	983.2404	-1.77	1	352.25	C27H20N4O3S	(2M+Na)+
984.2449	984.2434	-1.44	1	200.29	C27H20N4O3S	(2M+Na)+

4.33 Compound 31b

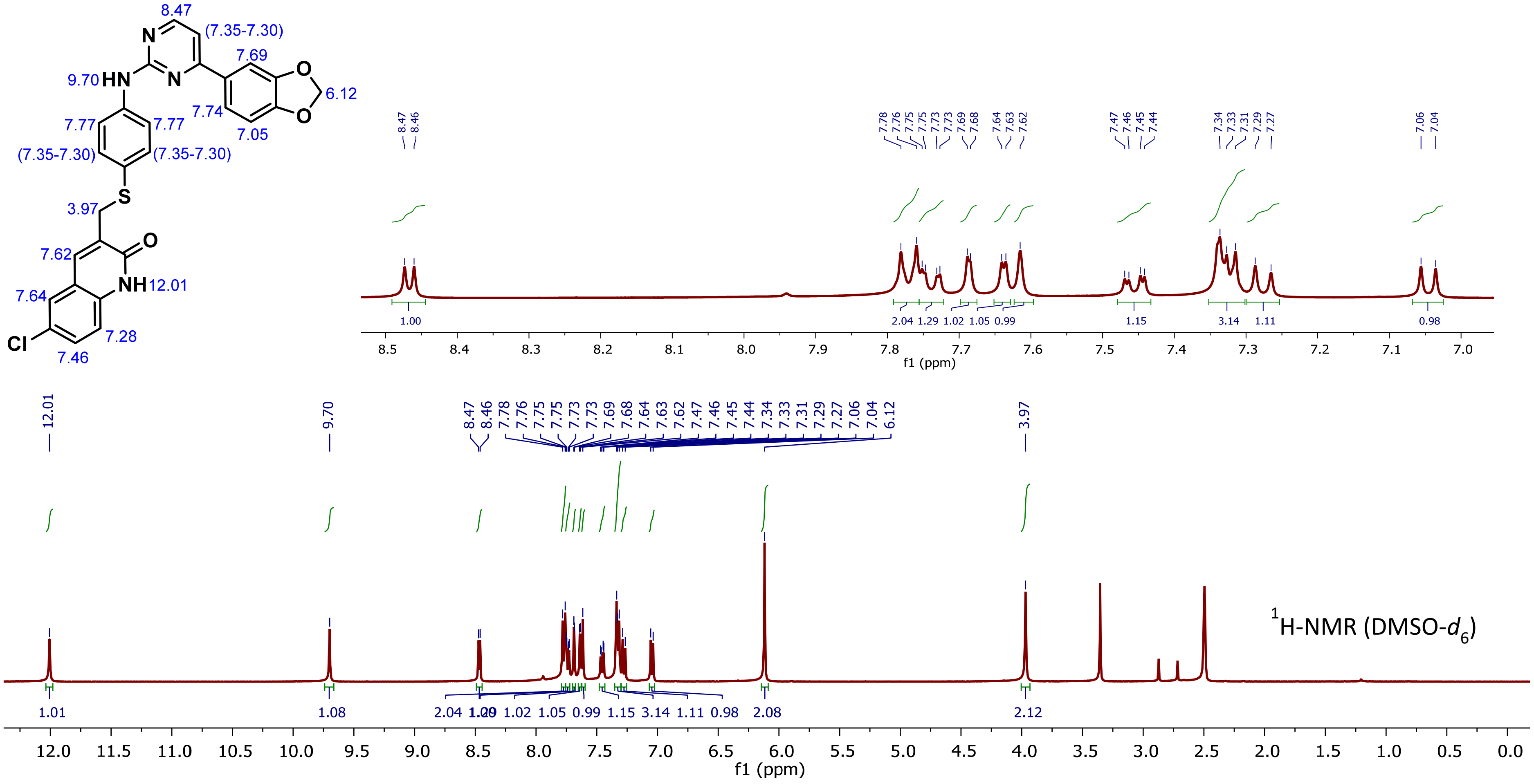

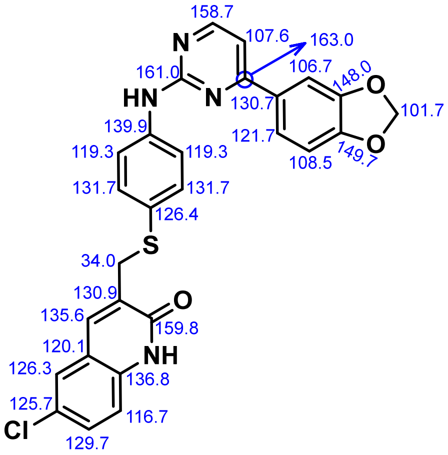
158.7
N
107.6
163.0
161.0
106.7
148.0
O
HN
N
130.7
101.7
139.9
121.7
O
119.3
119.3
108.5
149.7
131.7
131.7
126.4
34.0
S
130.9
O
135.6
159.8
120.1
NH
126.3
136.8
125.7
116.7
Cl
129.7

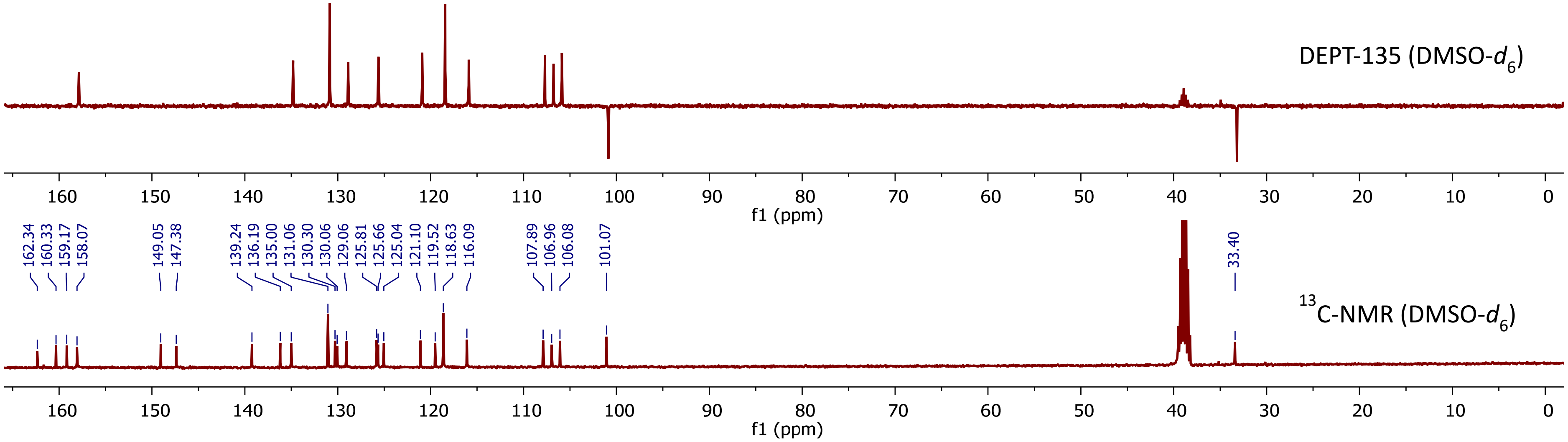
DEPT-135 (DMSO-d_6)
160
150
140
130
120
110
100
90
80
70
60
50
40
30
20
10
0
f1 (ppm)
162.34
160.33
159.17
158.07
149.05
147.38
139.24
136.19
135.00
131.06
130.30
130.06
129.06
125.81
125.66
125.04
121.10
119.52
118.63
116.09
107.89
106.96
106.08
101.07
33.40
^{13}C-NMR (DMSO-d_6)
160
150
140
130
120
110
100
90
80
70
60
50
40
30
20
10
0
f1 (ppm)

EI MS (70eV)

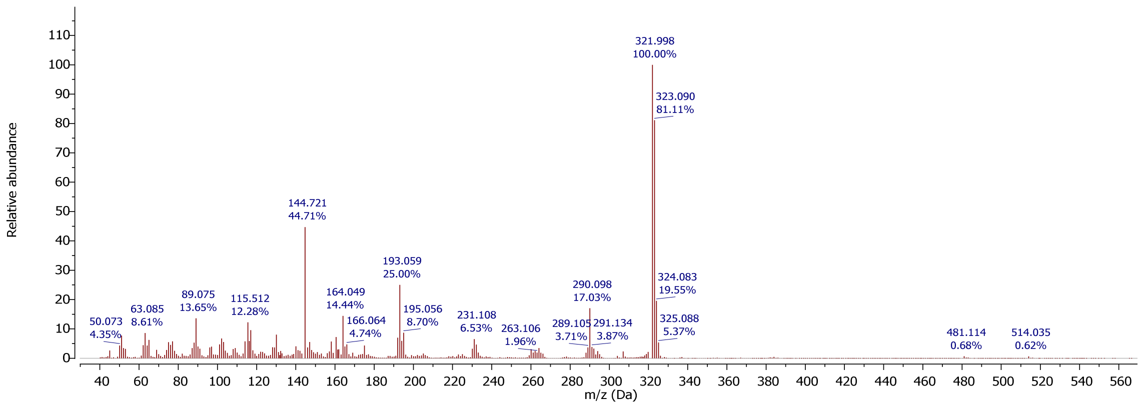

ESI-QTOF (positive ionization)

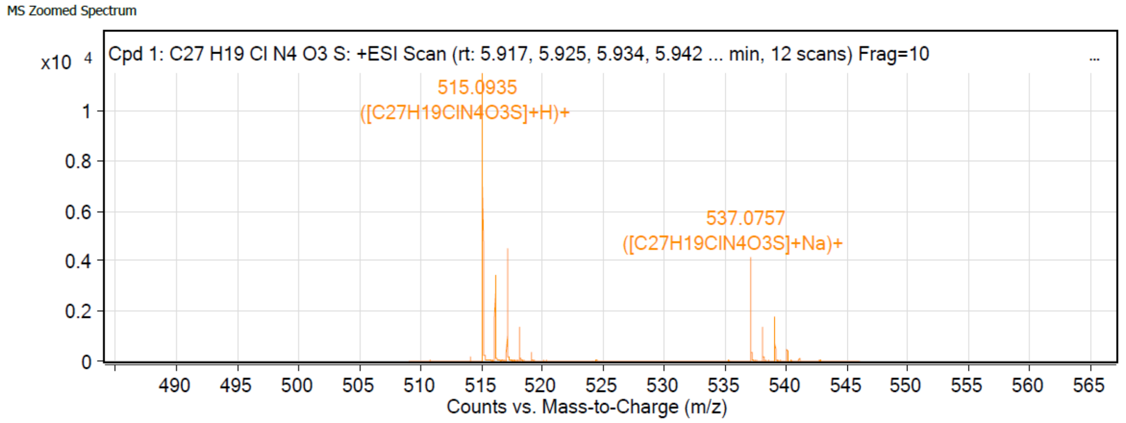

MS Spectrum Peak List

m/z	Calc *m/z*	Diff(ppm)	z	Abund	Formula	Ion
514.085	514.0861	2.22	1	145.65	C27H19ClN4O3S	M+
515.0935	515.0939	0.84	1	11522.77	C27H19ClN4O3S	(M+H)+
516.0969	516.0969	0.05	1	3560.53	C27H19ClN4O3S	(M+H)+
517.0919	517.092	0.16	1	4642.79	C27H19ClN4O3S	(M+H)+
518.0935	518.0943	1.54	1	1356.07	C27H19ClN4O3S	(M+H)+
519.0937	519.0927	-1.93	1	351.39	C27H19ClN4O3S	(M+H)+
537.0757	537.0759	0.23	1	4185.63	C27H19ClN4O3S	(M+Na)+
538.0784	538.0788	0.85	1	1410.28	C27H19ClN4O3S	(M+Na)+
539.0735	539.0739	0.8	1	1793.18	C27H19ClN4O3S	(M+Na)+
540.0759	540.0762	0.64	1	533.48	C27H19ClN4O3S	(M+Na)+

4.34 Compound 31c

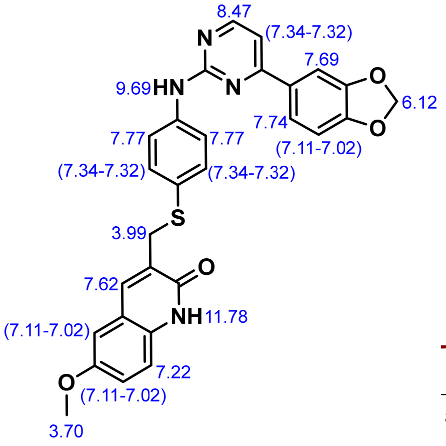

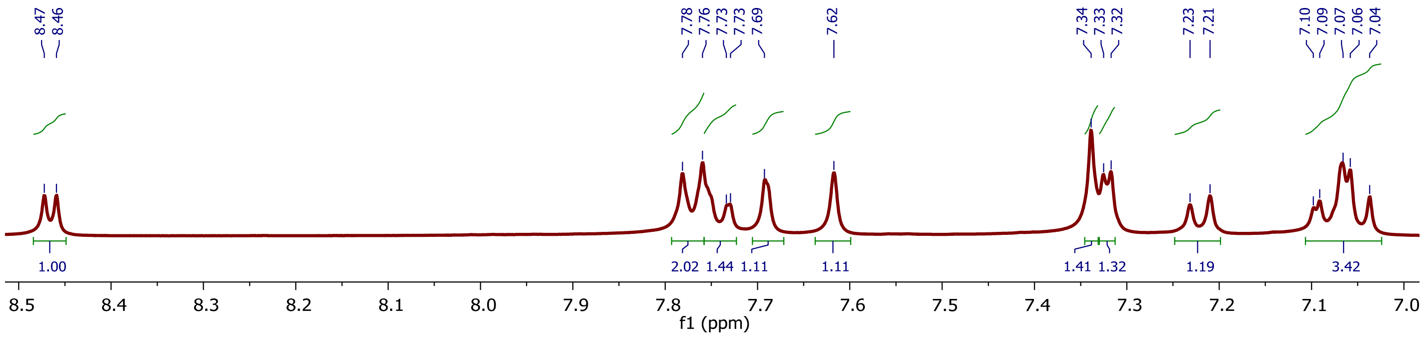

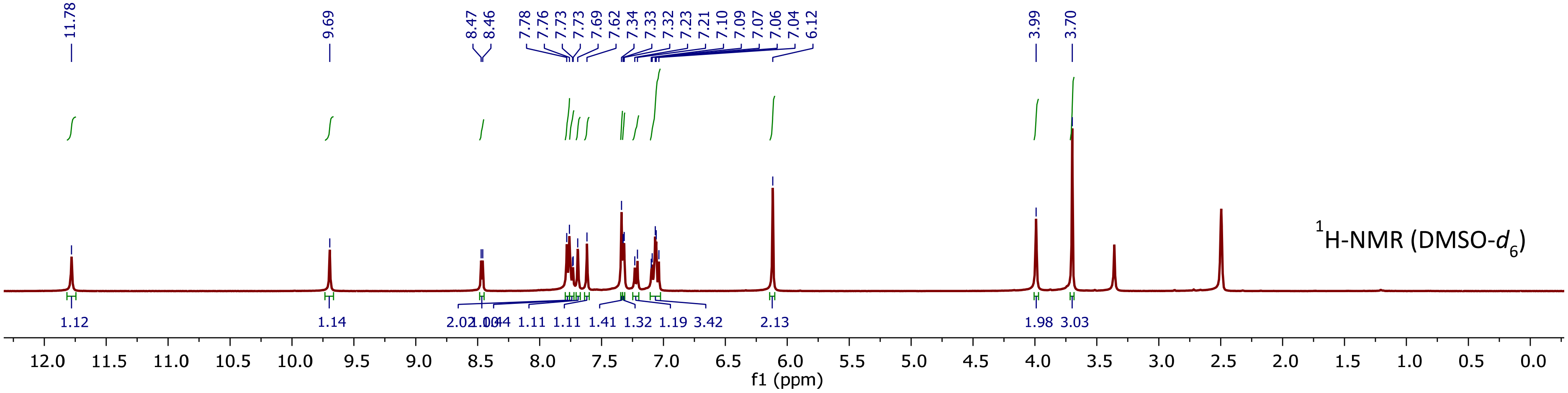

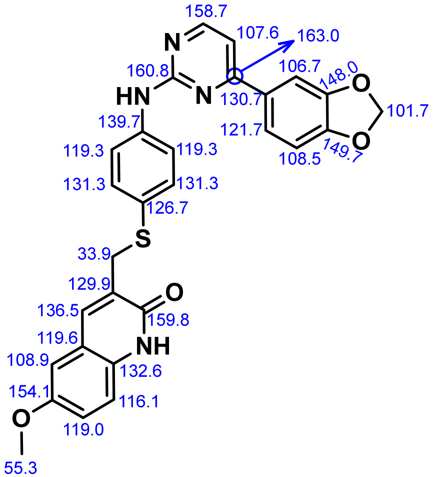
158.7
107.6
163.0
106.7
148.0
160.8
130.7
101.7
139.7
121.7
119.3
119.3
108.5
149.7
131.3
131.3
126.7
33.9
129.9
136.5
159.8
119.6
108.9
132.6
154.1
116.1
119.0
55.3

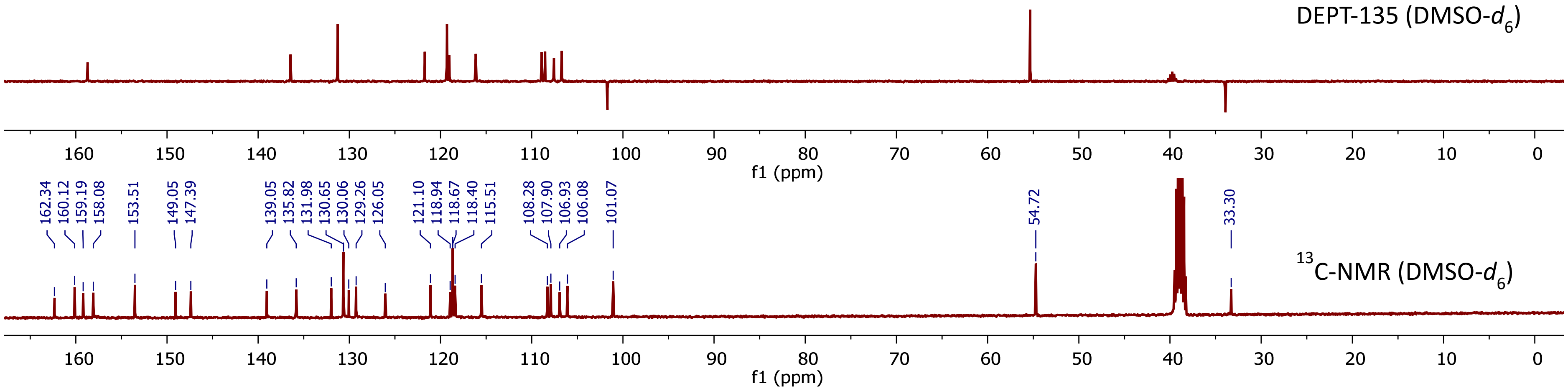
DEPT-135 (DMSO-d_6)
f1 (ppm)
162.34
160.12
159.19
158.08
153.51
149.05
147.39
139.05
135.82
131.98
130.65
130.06
129.26
126.05
121.10
118.94
118.67
118.40
115.51
108.28
107.90
106.93
106.08
101.07
54.72
33.30
^{13}C-NMR (DMSO-d_6)
f1 (ppm)

EI MS (70eV)

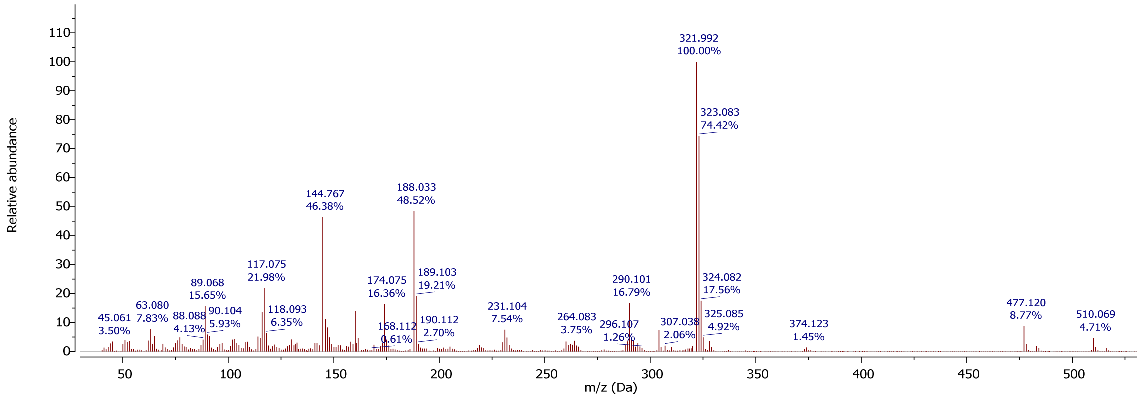

ESI-QTOF (positive ionization)

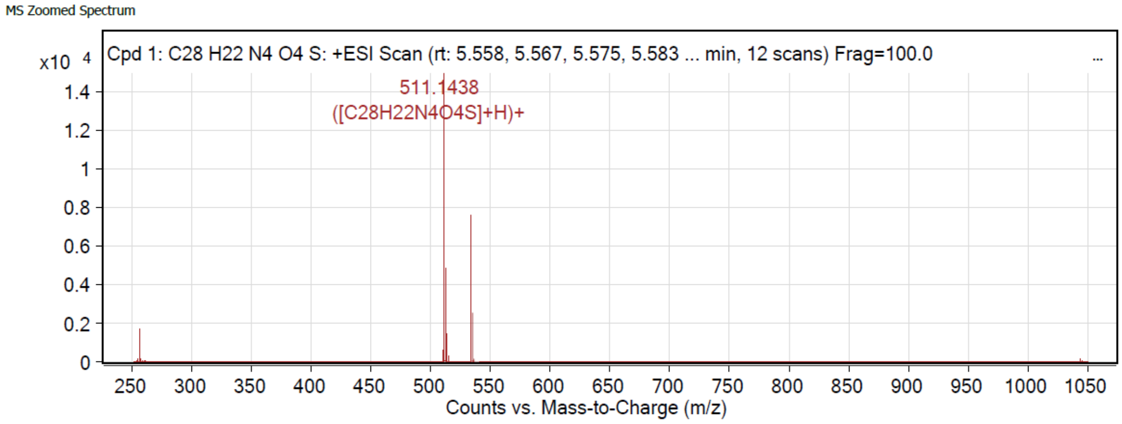

MS Spectrum Peak List

m/z	Calc *m/z*	Diff(ppm)	z	Abund	Formula	Ion
256.0746	256.0754	2.93	2	1763.84	C28H22N4O4S	(M+2H)+2
256.5772	256.5769	-1.17	2	685.52	C28H22N4O4S	(M+2H)+2
257.0777	257.0761	-6.04	2	183.36	C28H22N4O4S	(M+2H)+2
511.1438	511.1435	-0.71	1	14971.63	C28H22N4O4S	(M+H)+
512.1462	512.1465	0.55	1	4953.16	C28H22N4O4S	(M+H)+
513.1455	513.145	-0.93	1	1537.28	C28H22N4O4S	(M+H)+
533.1252	533.1254	0.42	1	7665.06	C28H22N4O4S	(M+Na)+
534.1284	534.1284	0.01	1	2610.76	C28H22N4O4S	(M+Na)+
535.1272	535.1269	-0.46	1	736.35	C28H22N4O4S	(M+Na)+
1043.2579	1043.2616	3.53	1	83.51	C28H22N4O4S	(2M+Na)+

4.35 Compound 32a

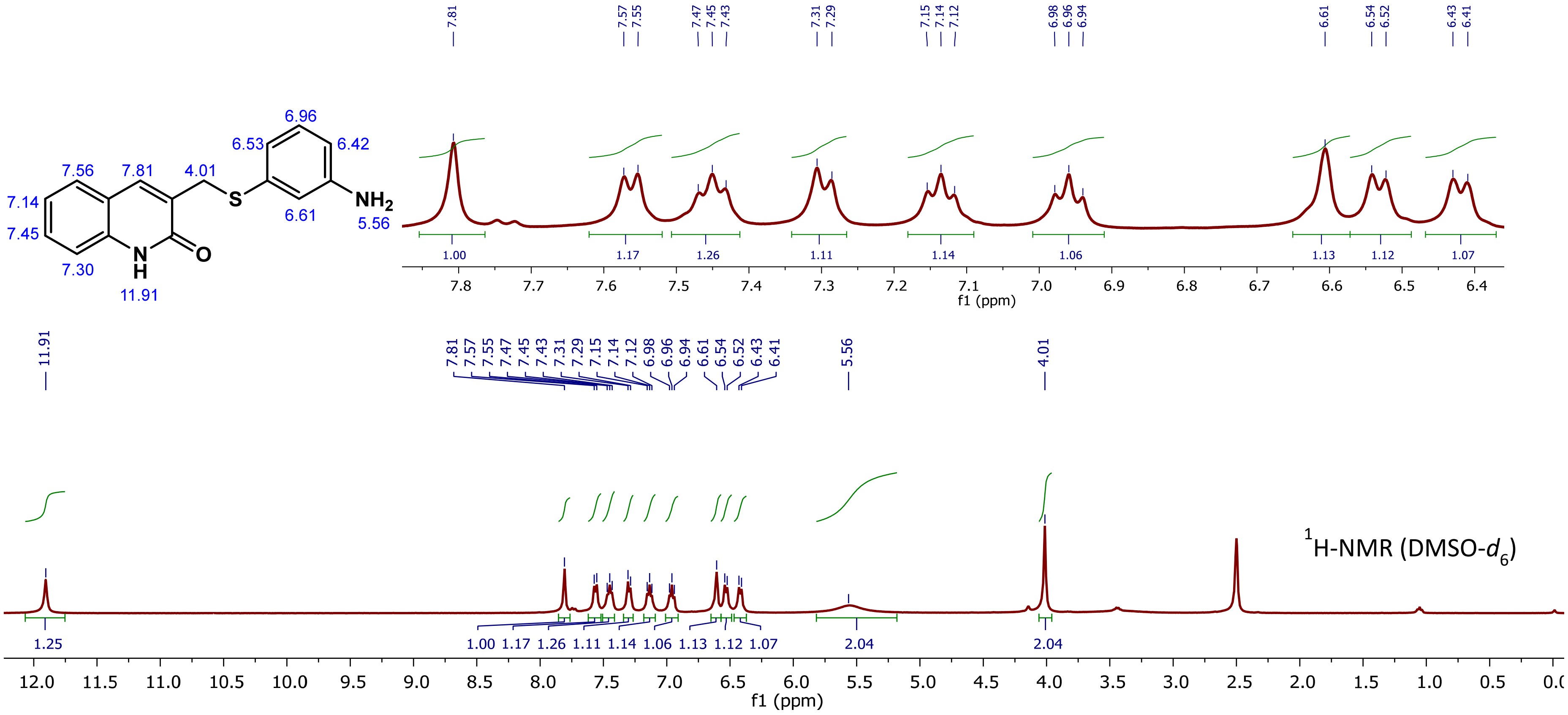

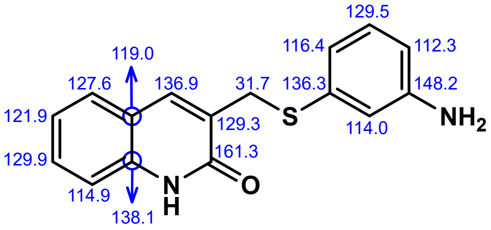
129.5
116.4
112.3
119.0
127.6
136.9
31.7
136.3
148.2
121.9
129.3
S
NH2
114.0
161.3
129.9
O
114.9
N
H
138.1

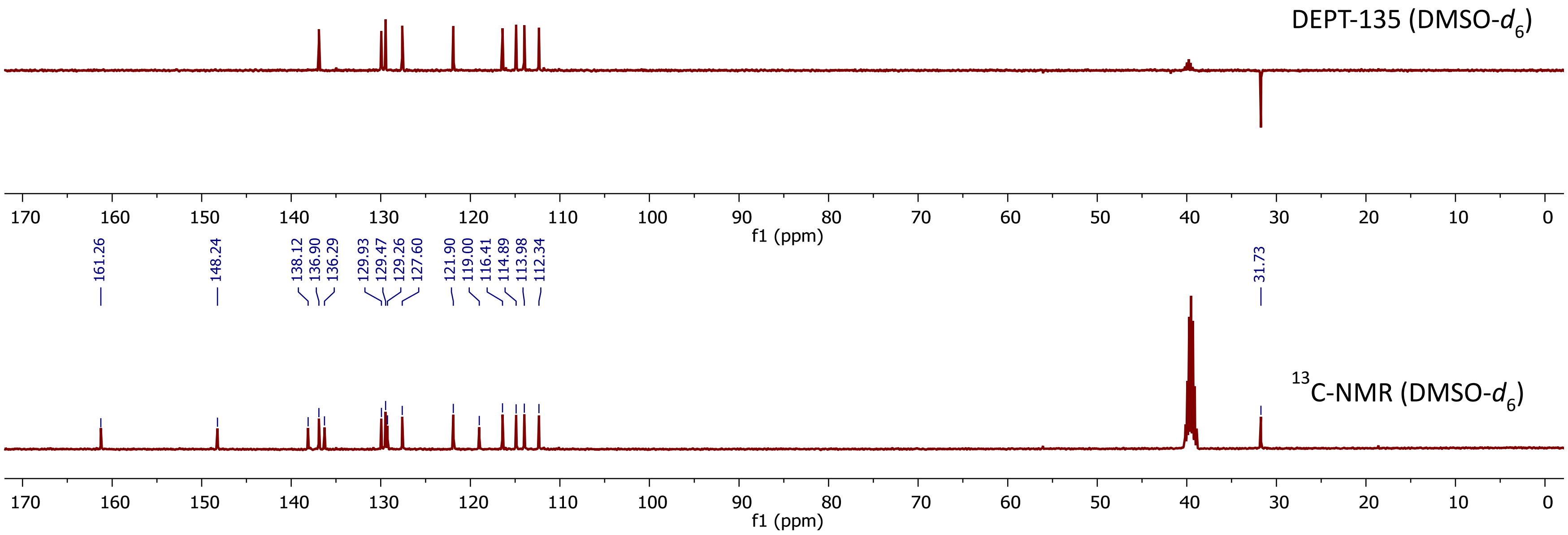
DEPT-135 (DMSO-d6)
f1 (ppm)
161.26
148.24
138.12
136.90
136.29
129.93
129.47
129.26
127.60
121.90
119.00
116.41
114.89
113.98
112.34
31.73
13C-NMR (DMSO-d6)
f1 (ppm)

EI MS (70eV)

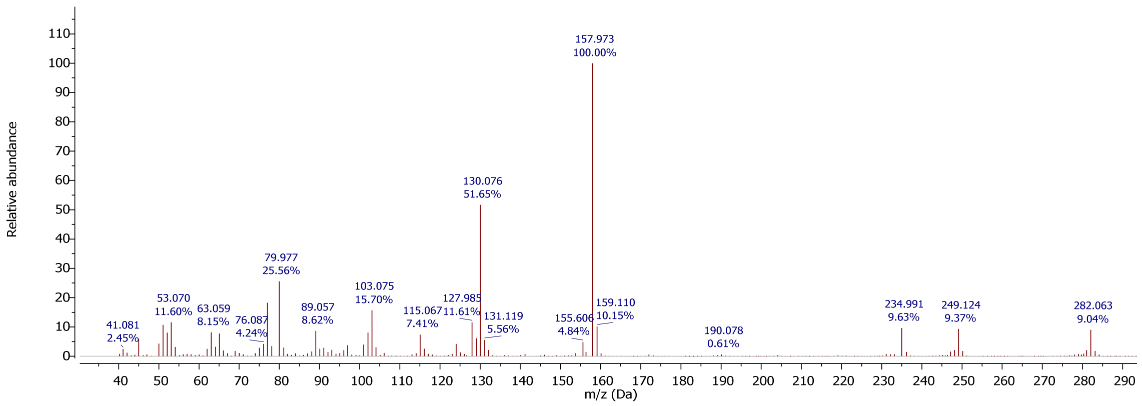

ESI-QTOF (positive ionization)

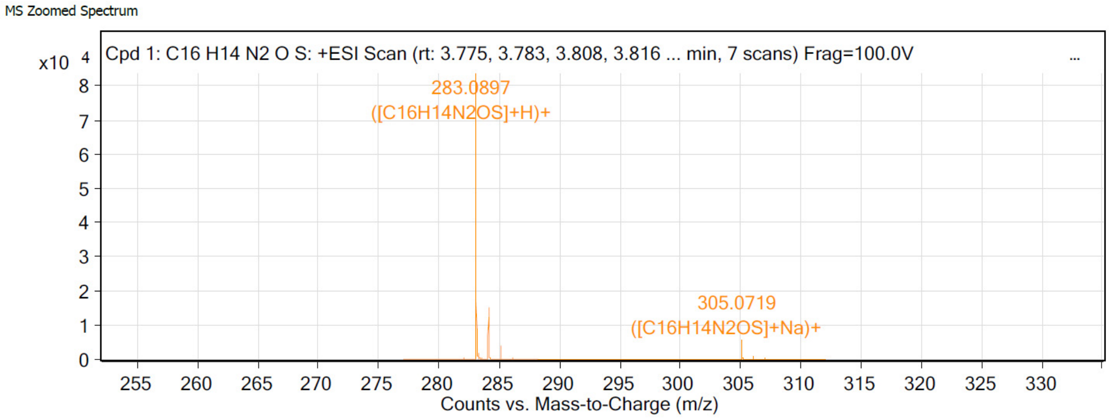

MS Spectrum Peak List

m/z	Calc *m/z*	Diff(ppm)	z	Abund	Formula	Ion
282.0827	282.0821	-1.93	1	538.14	C16H14N2OS	M+
283.0897	283.09	0.8	1	84261.67	C16H14N2OS	(M+H)+
284.0929	284.0929	0.02	1	15045.32	C16H14N2OS	(M+H)+
285.0885	285.0887	0.89	1	4221.72	C16H14N2OS	(M+H)+
305.0719	305.0719	0.16	1	5652.01	C16H14N2OS	(M+Na)+
306.0753	306.0749	-1.28	1	1184.37	C16H14N2OS	(M+Na)+
307.0701	307.0707	1.83	1	367.66	C16H14N2OS	(M+Na)+

4.36 Compound 32b

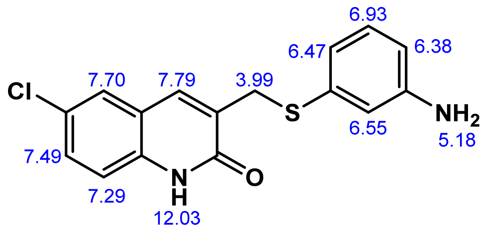

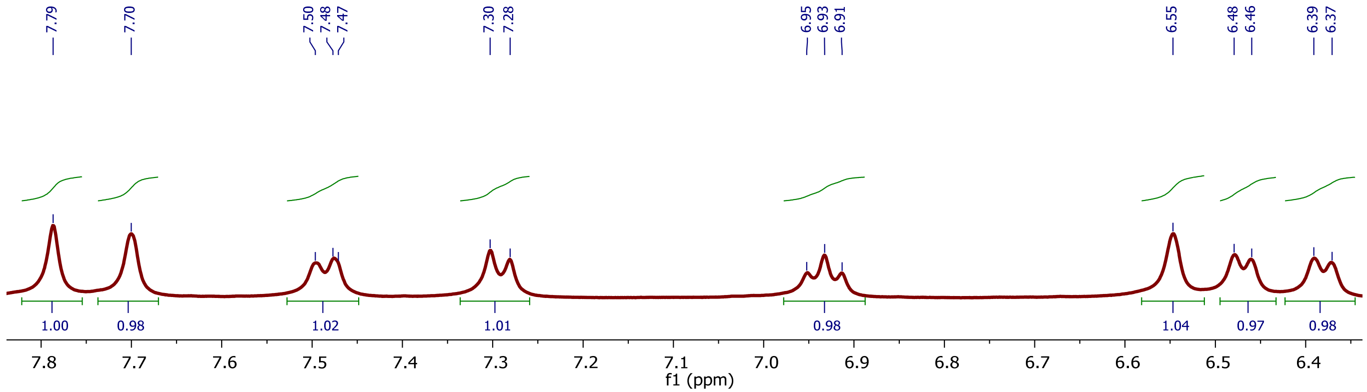

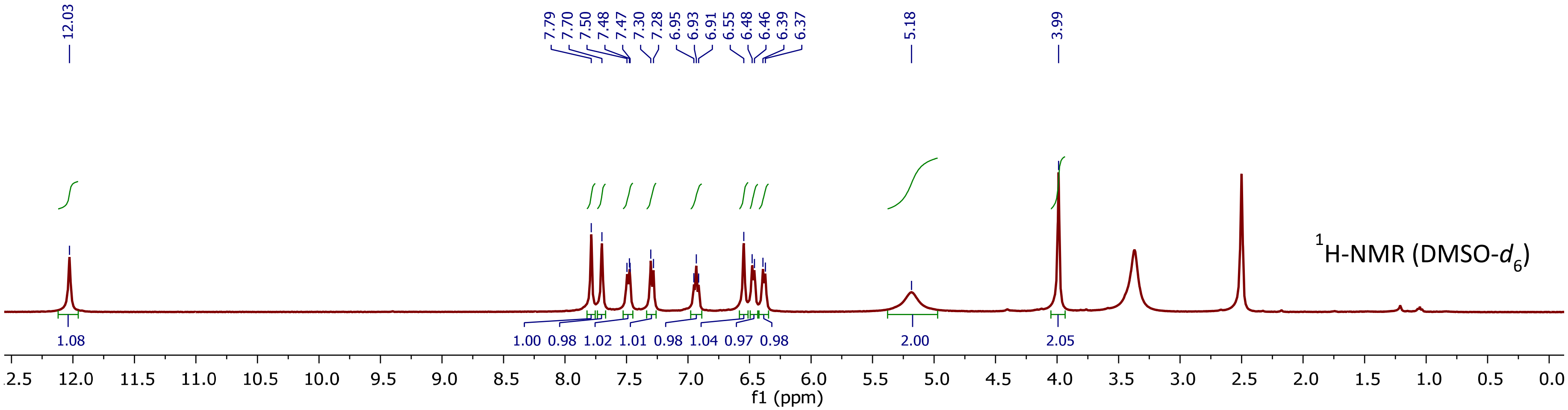

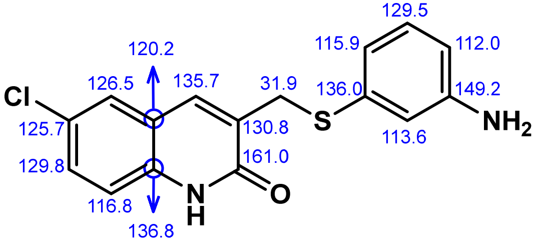
Cl
NH$_2$
S
N
H
O
120.2
126.5
135.7
31.9
136.0
115.9
129.5
112.0
149.2
113.6
125.7
130.8
161.0
129.8
116.8
136.8

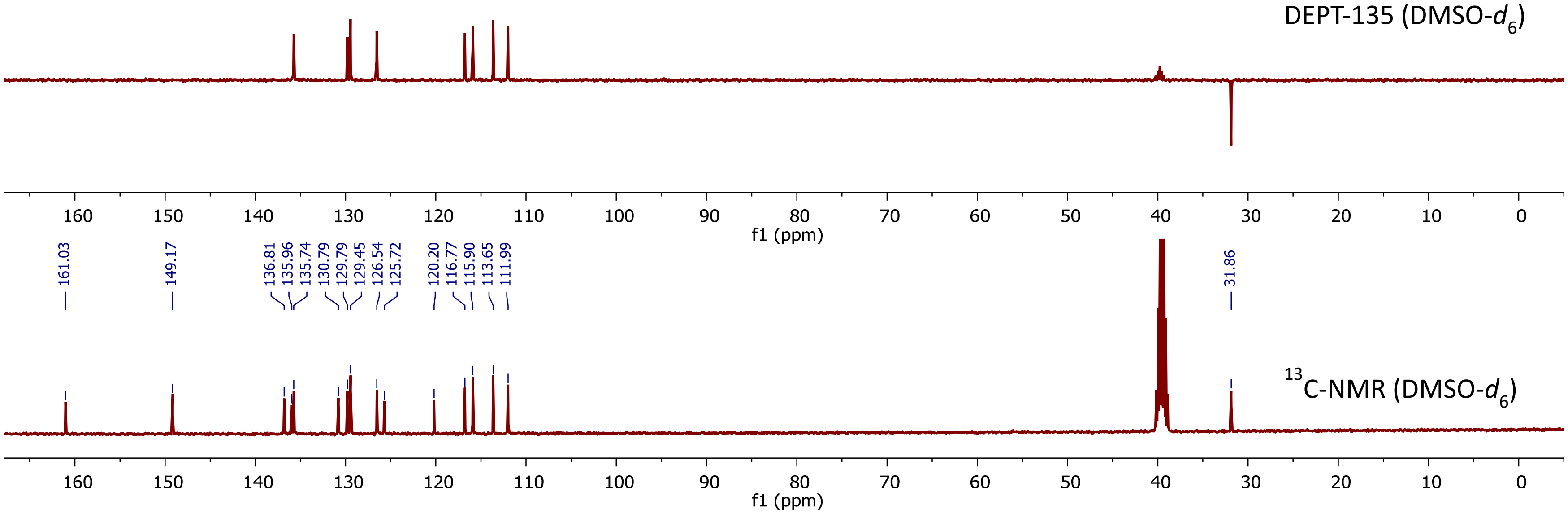
DEPT-135 (DMSO-d_6)
^{13}C-NMR (DMSO-d_6)
f1 (ppm)
161.03
149.17
136.81
135.96
135.74
130.79
129.79
129.45
126.54
125.72
120.20
116.77
115.90
113.65
111.99
31.86

EI MS (70eV)

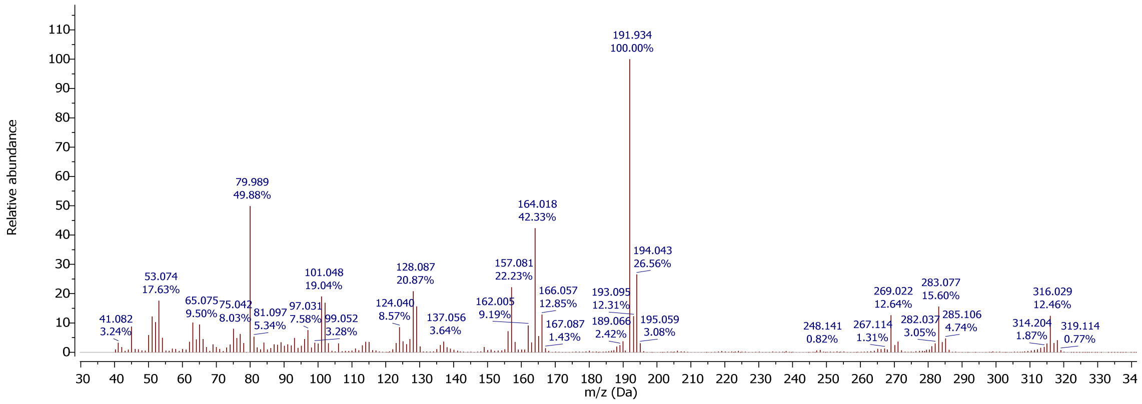

ESI-QTOF (positive ionization)

MS Zoomed Spectrum

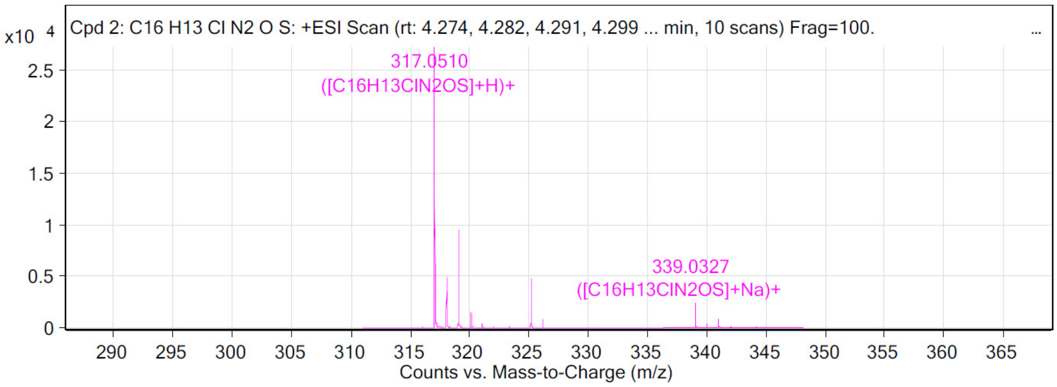

MS Spectrum Peak List

m/z	Calc *m/z*	Diff(ppm)	z	Abund	Formula	Ion
316.045	316.0432	-5.68	1	103.78	C16H13ClN2OS	M+
317.051	317.051	0	1	27328.75	C16H13ClN2OS	(M+H)+
318.0537	318.054	0.83	1	5062.02	C16H13ClN2OS	(M+H)+
319.0482	319.0483	0.49	1	9851.85	C16H13ClN2OS	(M+H)+
320.0515	320.051	-1.44	1	1715.77	C16H13ClN2OS	(M+H)+
321.0484	321.0471	-4.17	1	507.61	C16H13ClN2OS	(M+H)+
339.0327	339.0329	0.79	1	2483.84	C16H13ClN2OS	(M+Na)+
[illegible]	[illegible]	[illegible]	1	[illegible]	[illegible]	[illegible]
341.03	341.0303	0.81	1	915.04	C16H13ClN2OS	(M+Na)+
342.0322	342.033	2.33	1	175.25	C16H13ClN2OS	(M+Na)+

4.37 Compound 32c

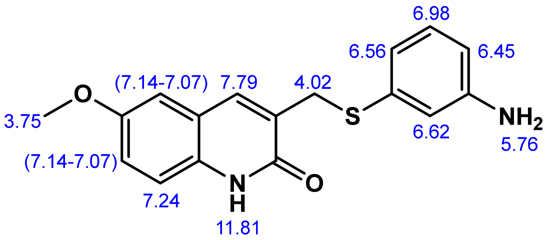

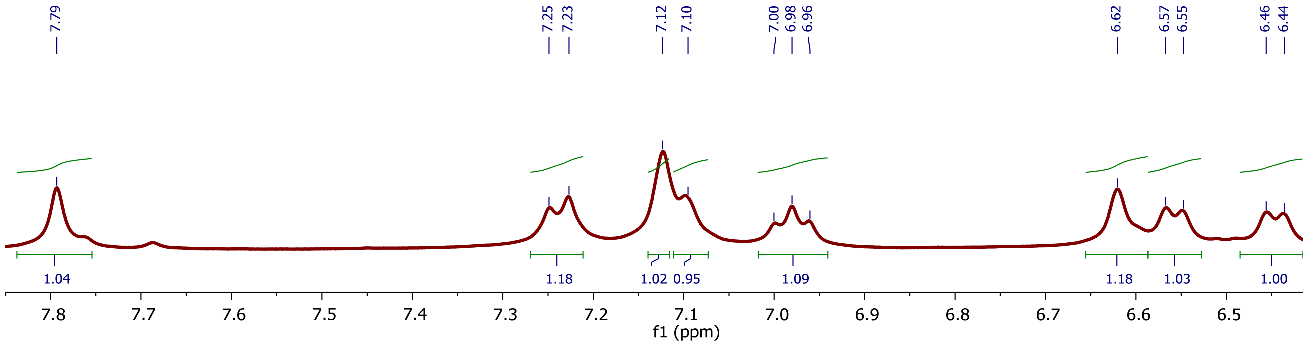

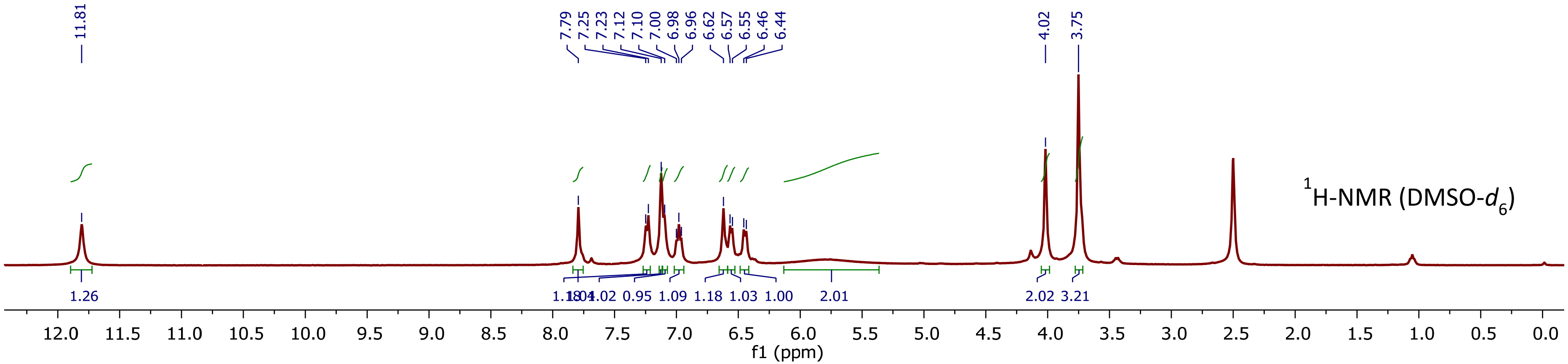

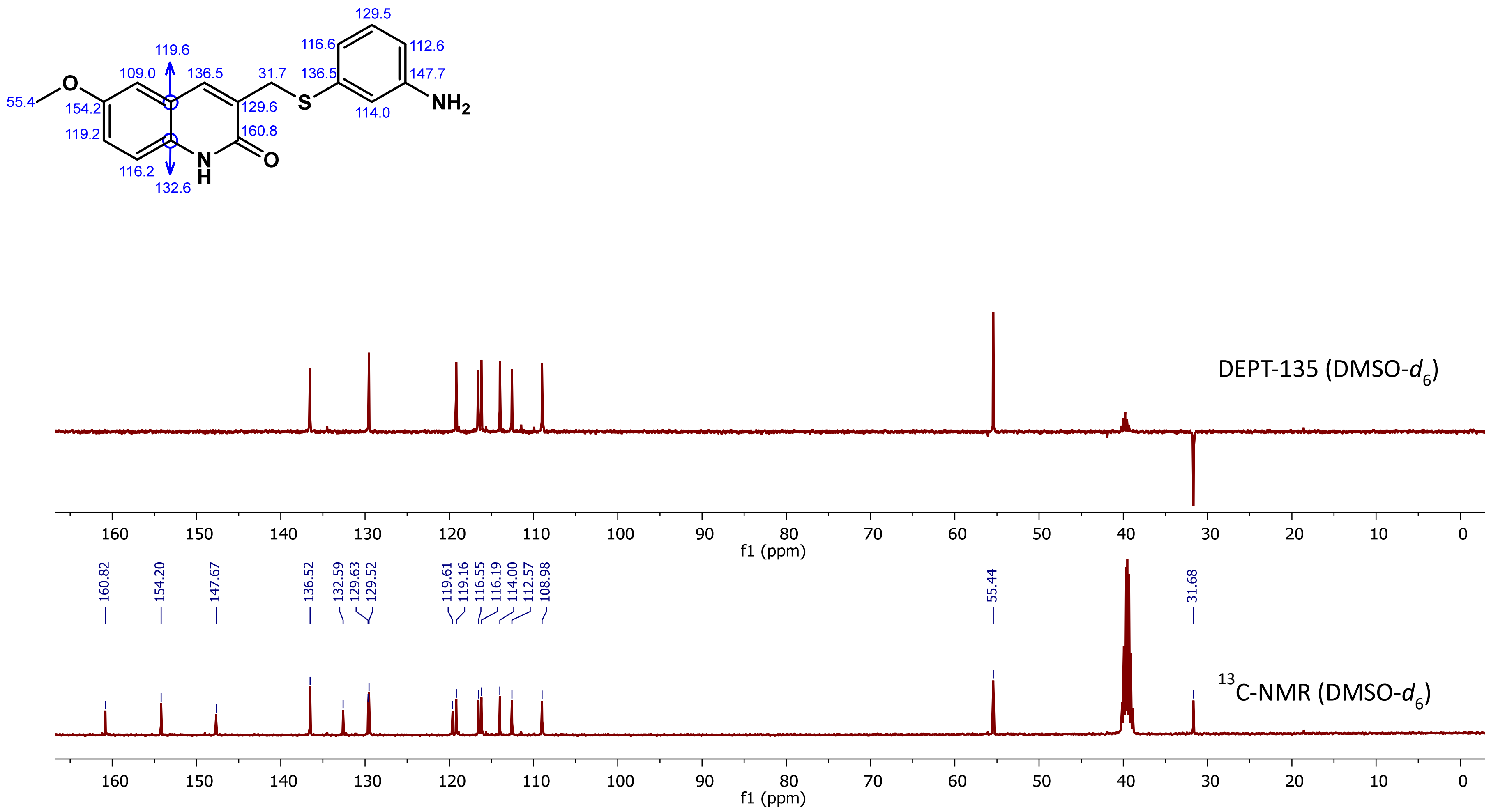

55.4
O
154.2
119.2
116.2
109.0
119.6
136.5
132.6
N
H
O
160.8
129.6
31.7
S
136.5
116.6
129.5
112.6
147.7
NH2
114.0
DEPT-135 (DMSO-d6)
f1 (ppm)
160.82
154.20
147.67
136.52
132.59
129.63
129.52
119.61
119.16
116.55
116.19
114.00
112.57
108.98
55.44
31.68
13C-NMR (DMSO-d6)
f1 (ppm)

EI MS (70eV)

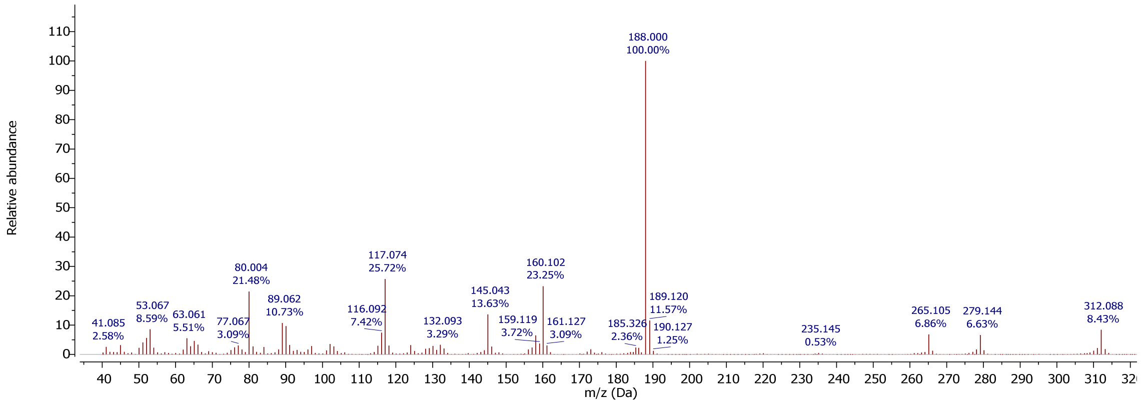

ESI-QTOF (positive ionization)

MS Zoomed Spectrum

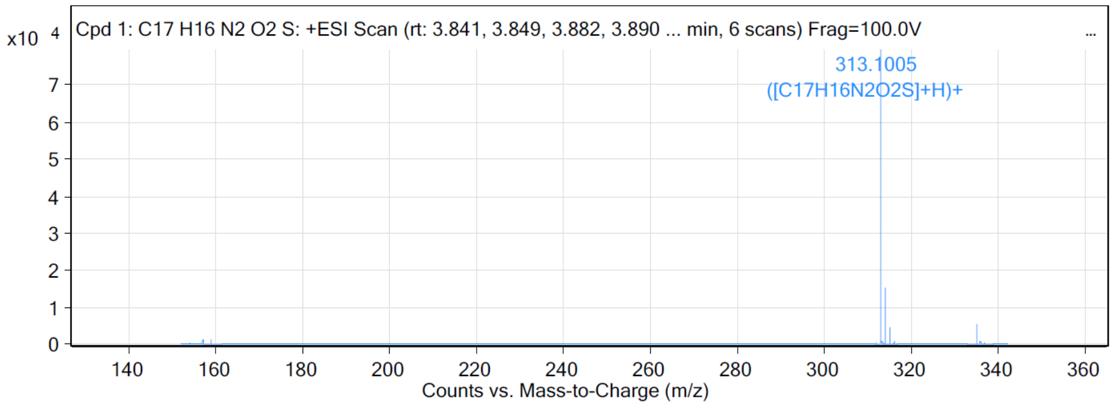

MS Spectrum Peak List

m/z	Calc *m/z*	Diff(ppm)	z	Abund	Formula	Ion
157.0534	157.0539	3.1	2	1327.52	C17H16N2O2S	(M+2H)+2
157.5539	157.5554	9.41	2	217.53	C17H16N2O2S	(M+2H)+2
158.057	158.0535	-22.44	2	103.37	C17H16N2O2S	(M+2H)+2
312.0924	312.0927	0.81	1	601.84	C17H16N2O2S	M+
313.1005	313.1005	-0.04	1	81732.62	C17H16N2O2S	(M+H)+
314.1036	314.1035	-0.32	1	15167.51	C17H16N2O2S	(M+H)+
315.0996	315.0997	0.27	1	4440.54	C17H16N2O2S	(M+H)+
335.0826	335.0825	-0.42	1	5570.1	C17H16N2O2S	(M+Na)+
336.0856	336.0855	-0.26	1	1072.04	C17H16N2O2S	(M+Na)+
337.081	337.0817	2.09	1	358.39	C17H16N2O2S	(M+Na)+

4.38 Compound 35a

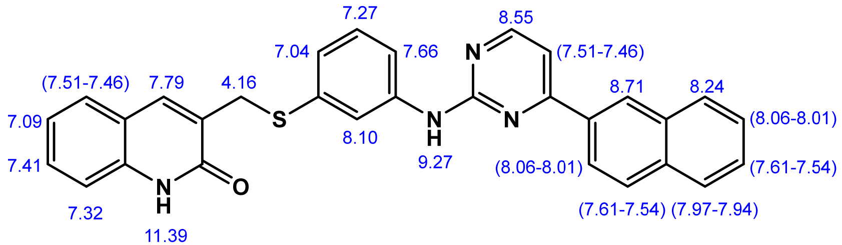

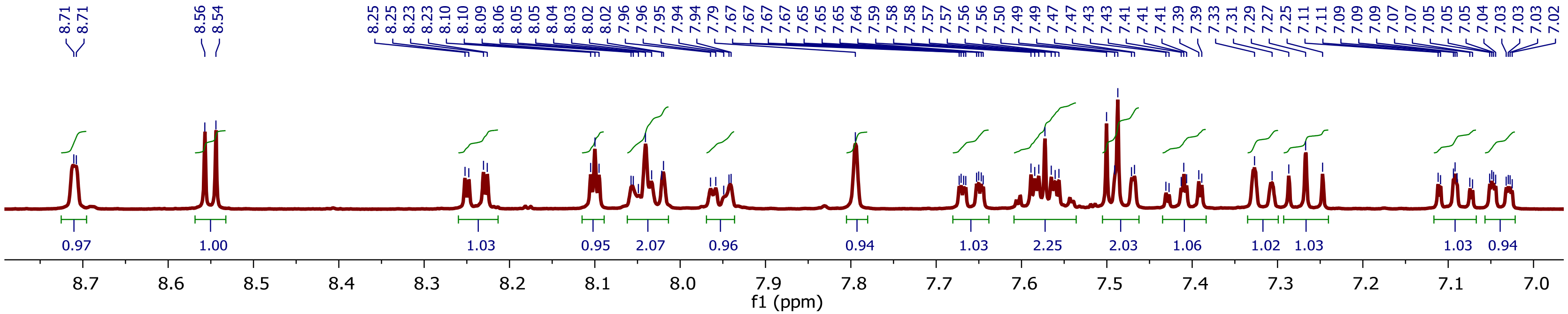

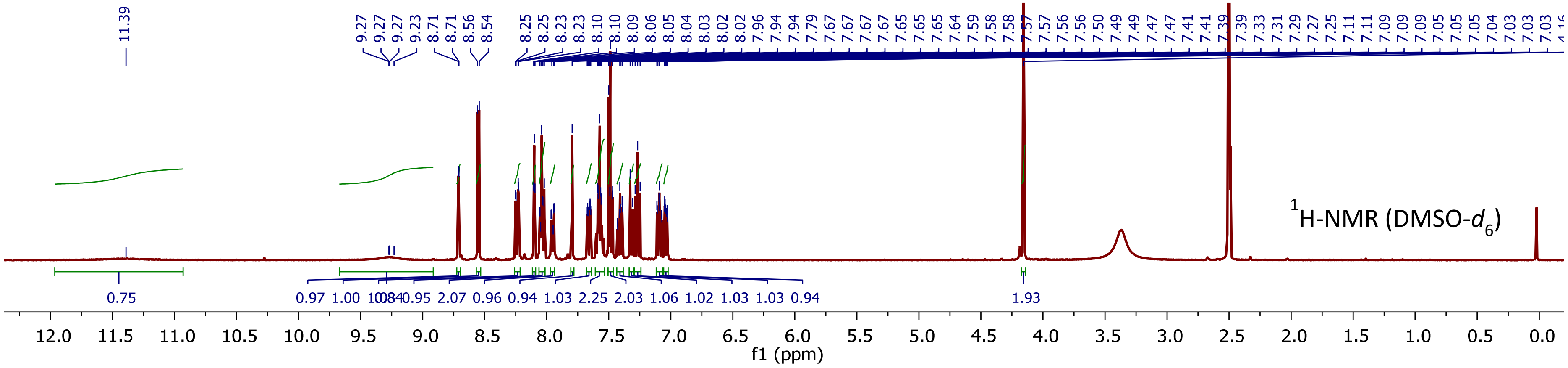

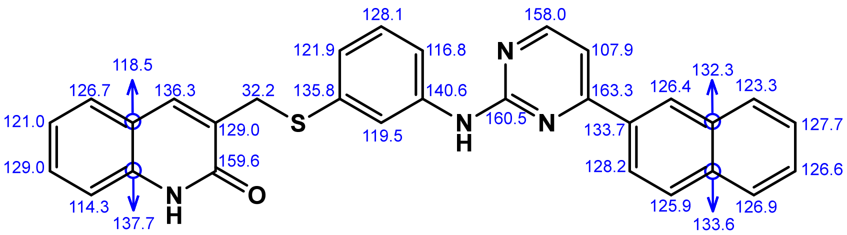
118.5
126.7
136.3
32.2
135.8
128.1
121.9
116.8
158.0
107.9
121.0
129.0
159.6
129.0
114.3
137.7
S
140.6
160.5
119.5
N
H
N
N
163.3
126.4
132.3
123.3
133.7
127.7
128.2
126.6
125.9
133.6
126.9
O

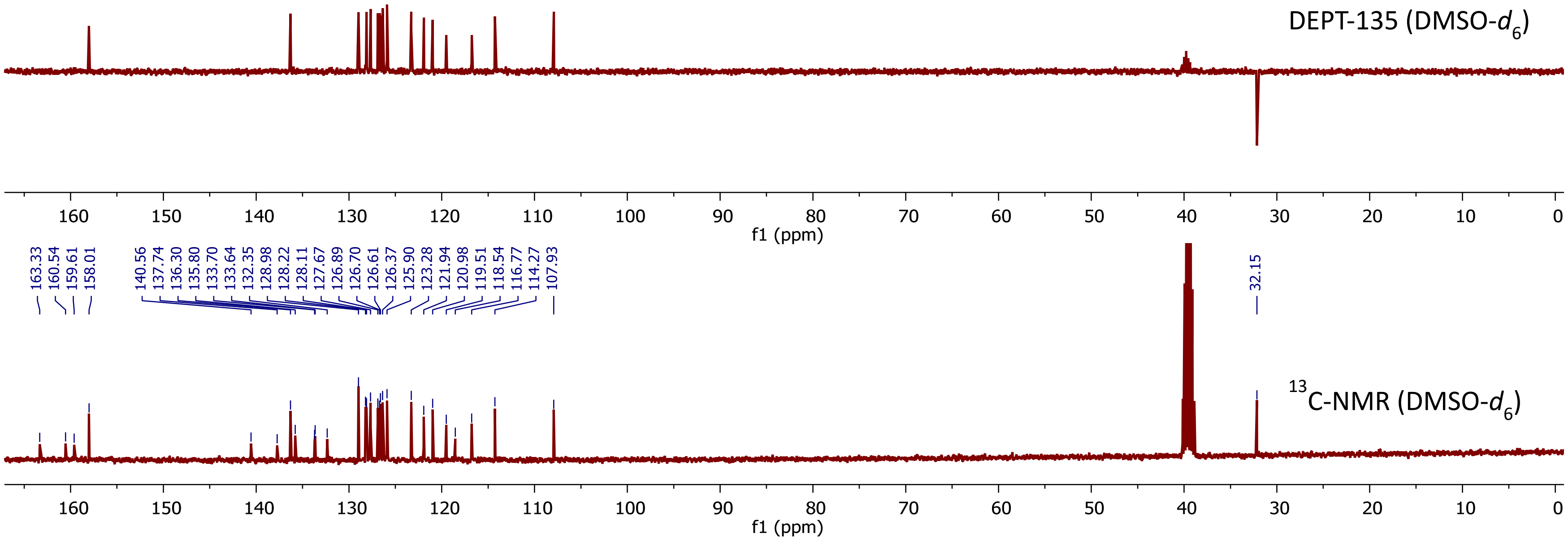
DEPT-135 (DMSO-d6)
160 150 140 130 120 110 100 90 80 70 60 50 40 30 20 10 0
f1 (ppm)
163.33
160.54
159.61
158.01
140.56
137.74
136.30
135.80
133.70
133.64
132.35
128.98
128.22
128.11
127.67
126.89
126.70
126.61
126.37
125.90
123.28
121.94
120.98
119.51
118.54
116.77
114.27
107.93
32.15
13C-NMR (DMSO-d6)
160 150 140 130 120 110 100 90 80 70 60 50 40 30 20 10 0
f1 (ppm)

EI MS (70eV)

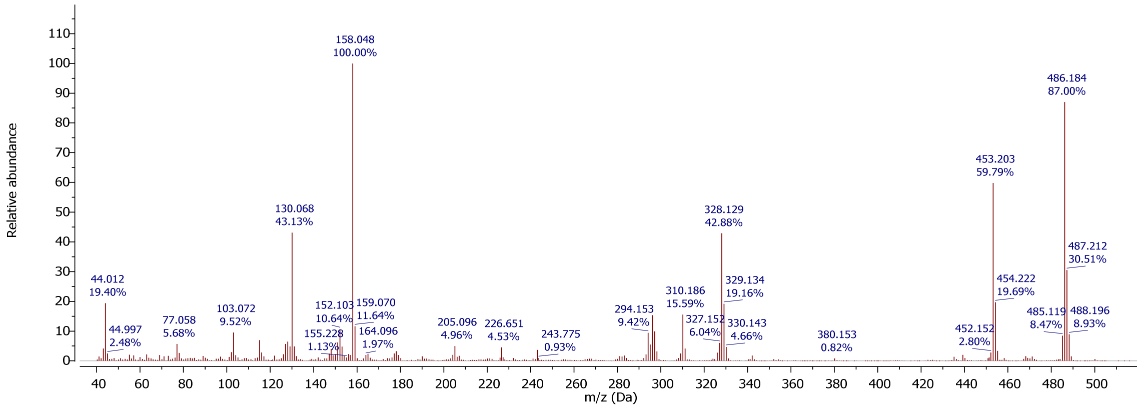

ESI-QTOF (positive ionization)

MS Zoomed Spectrum

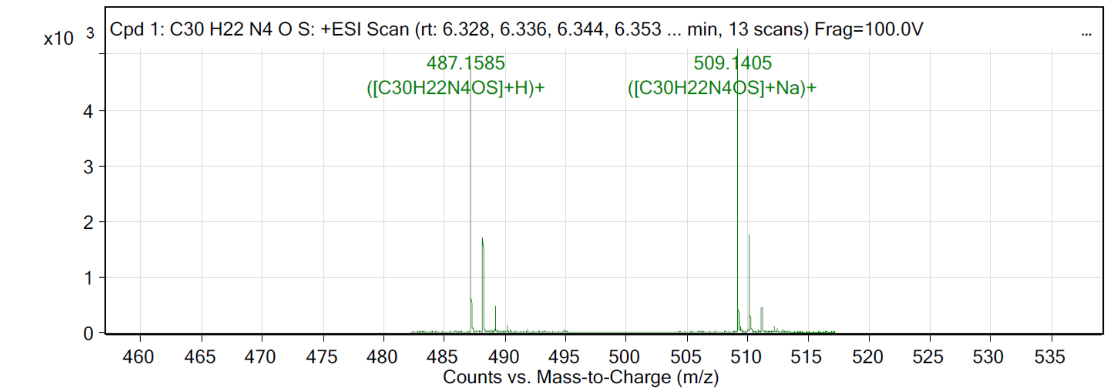

MS Spectrum Peak List

m/z	Calc *m/z*	Diff(ppm)	z	Abund	Formula	Ion
487.1585	487.1587	0.48	1	4914.18	C30H22N4OS	(M+H)+
488.1618	488.1617	-0.13	1	1750.32	C30H22N4OS	(M+H)+
489.1595	489.1604	1.83	1	516.34	C30H22N4OS	(M+H)+
490.1622	490.1608	-2.83	1	78.4	C30H22N4OS	(M+H)+
509.1405	509.1407	0.35	1	5170.75	C30H22N4OS	(M+Na)+
510.1434	510.1437	0.59	1	1867.81	C30H22N4OS	(M+Na)+
511.1418	511.1423	1.13	1	513.31	C30H22N4OS	(M+Na)+
512.1422	512.1428	1	1	82.34	C30H22N4OS	(M+Na)+

4.39 Compound 35b

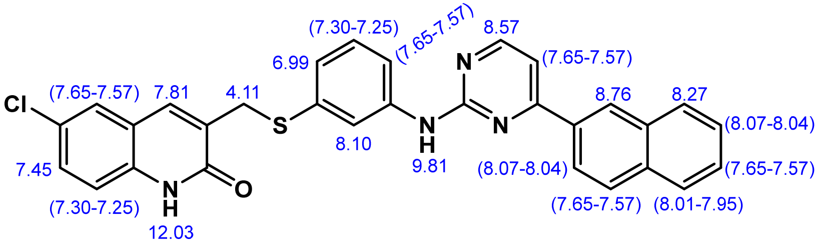

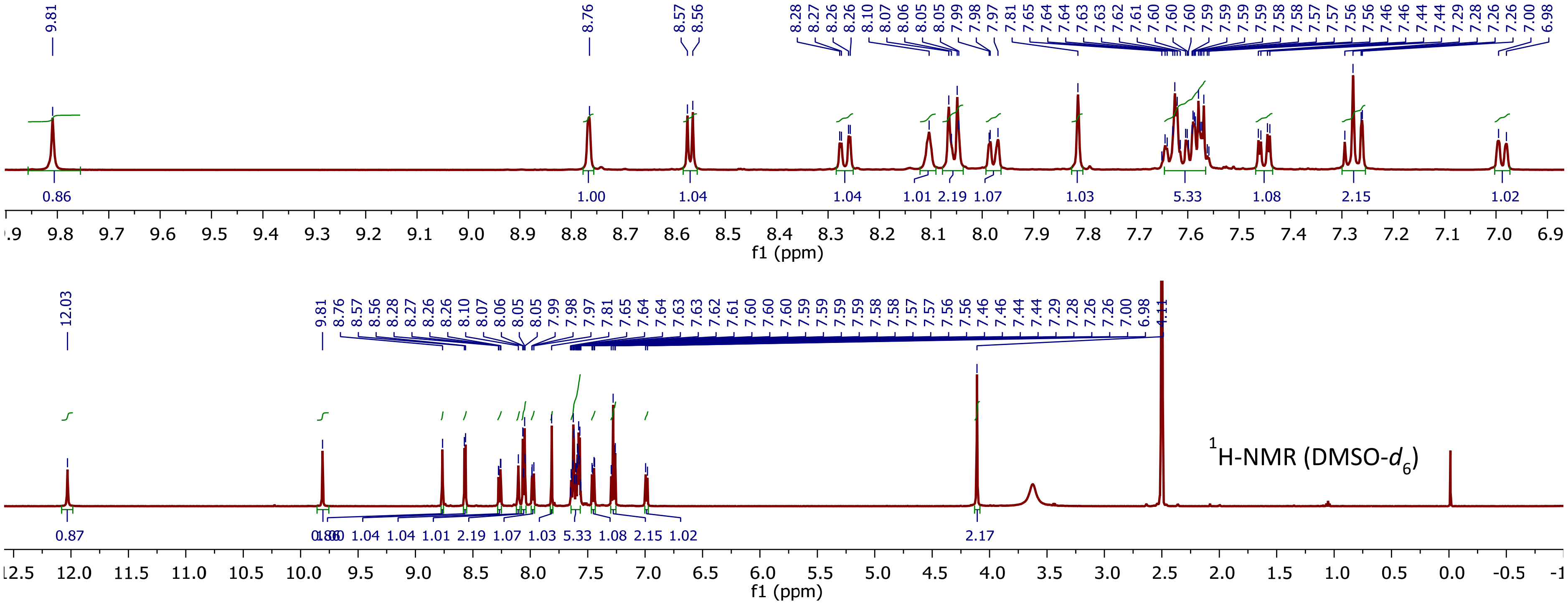

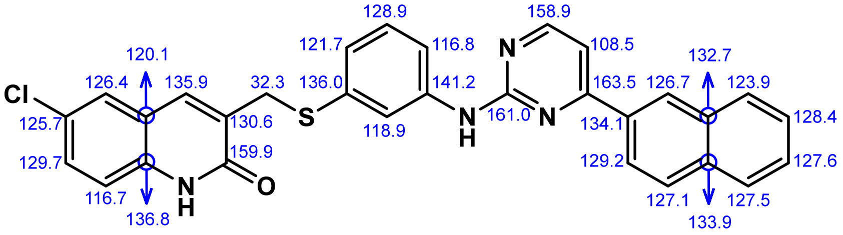

Cl
125.7
129.7
116.7
126.4
120.1
135.9
136.8
N
H
O
130.6
159.9
32.3
S
136.0
121.7
128.9
116.8
141.2
118.9
N
H
161.0
N
N
158.9
108.5
163.5
134.1
129.2
126.7
132.7
123.9
128.4
127.6
127.5
127.1
133.9

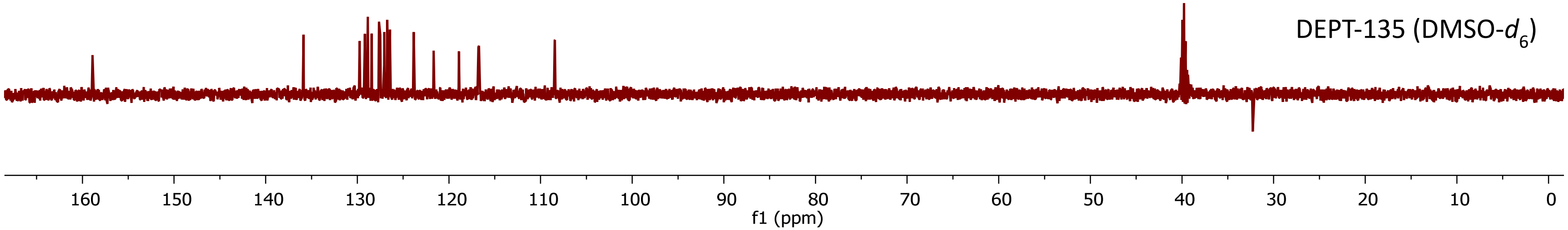

DEPT-135 (DMSO-d_6)
160 150 140 130 120 110 100 90 80 70 60 50 40 30 20 10 0
f1 (ppm)

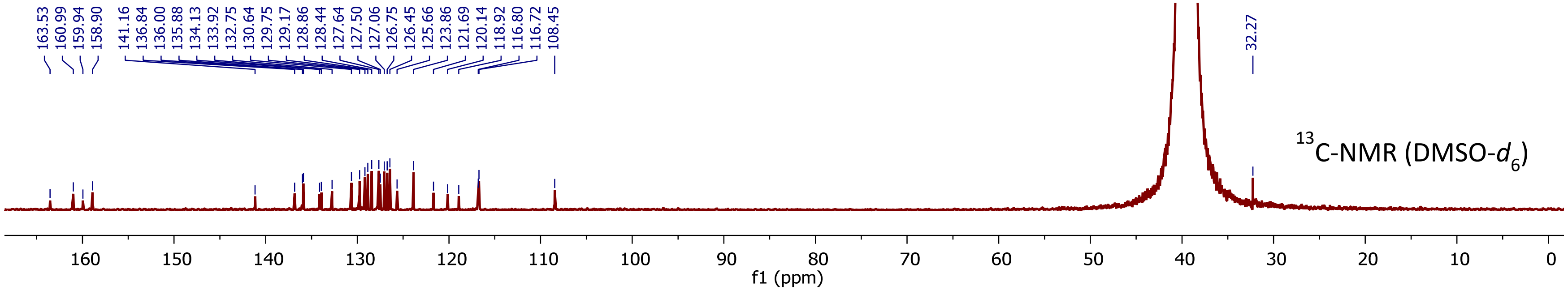

163.53
160.99
159.94
158.90
141.16
136.84
136.00
135.88
134.13
133.92
132.75
130.64
129.75
129.17
128.86
128.44
127.64
127.50
127.06
126.75
126.45
125.66
123.86
121.69
120.14
118.92
116.80
116.72
108.45
32.27
^{13}C-NMR (DMSO-d_6)
160 150 140 130 120 110 100 90 80 70 60 50 40 30 20 10 0
f1 (ppm)

EI MS (70eV)

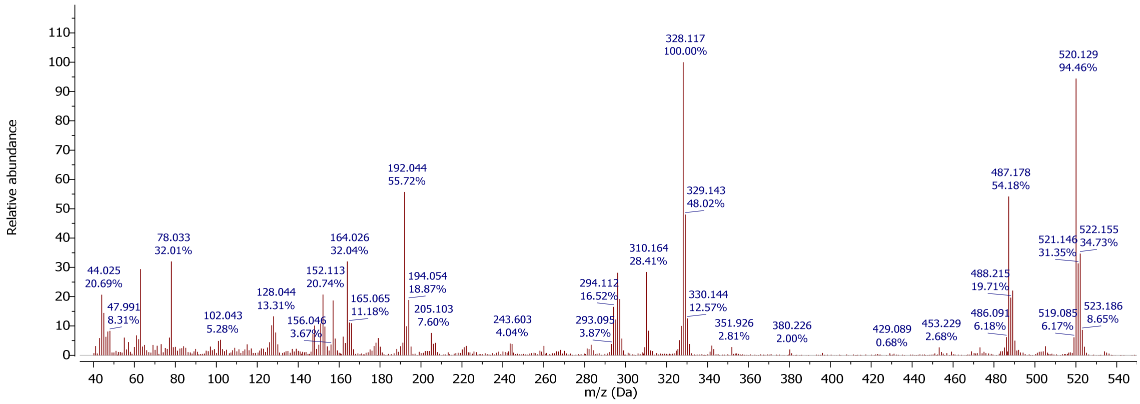

ESI-QTOF (positive ionization)

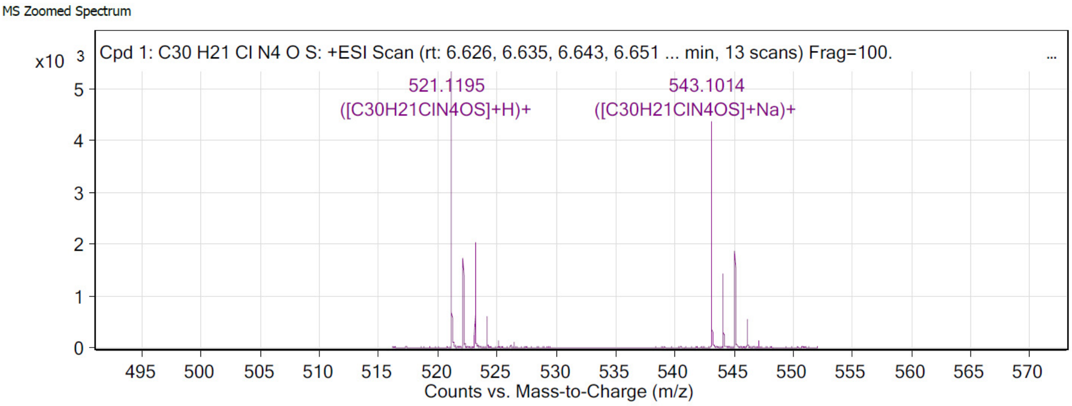

MS Spectrum Peak List

m/z	Calc *m/z*	Diff(ppm)	z	Abund	Formula	Ion
521.1195	521.1197	0.48	1	5458.62	C30H21ClN4OS	(M+H)+
522.1225	522.1228	0.56	1	1815.21	C30H21ClN4OS	(M+H)+
523.117	523.1179	1.75	1	2090.69	C30H21ClN4OS	(M+H)+
524.1203	524.1201	-0.34	1	619.73	C30H21ClN4OS	(M+H)+
525.1183	525.1189	1.13	1	126.98	C30H21ClN4OS	(M+H)+
543.1014	543.1017	0.45	1	4458.7	C30H21ClN4OS	(M+Na)+
544.1047	544.1047	0.02	1	1534.79	C30H21ClN4OS	(M+Na)+
545.1003	545.0999	-0.83	1	1950.36	C30H21ClN4OS	(M+Na)+
[illegible]	[illegible]	[illegible]	1	[illegible]	C30H21ClN4OS	(M+Na)+
547.1014	547.1009	-0.92	1	126.62	C30H21ClN4OS	(M+Na)+

4.40 Compound 35c

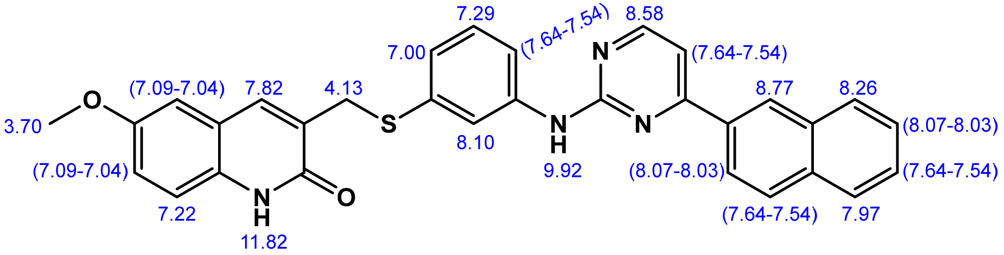

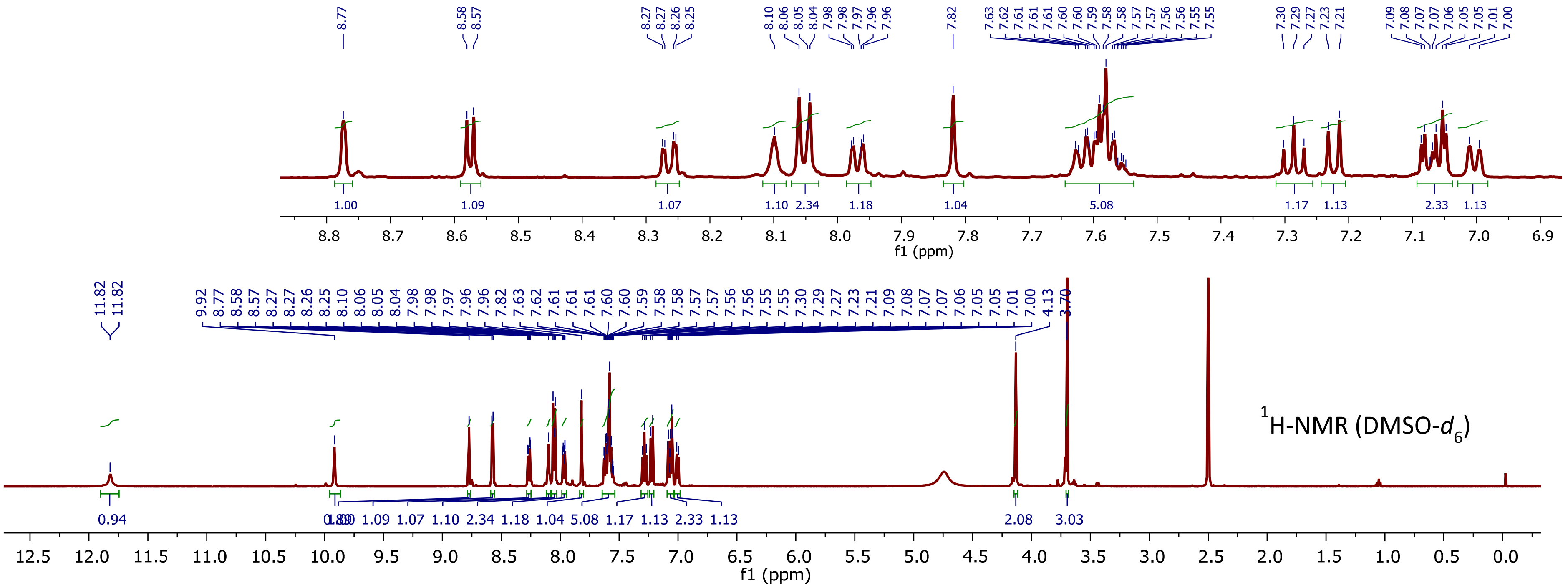

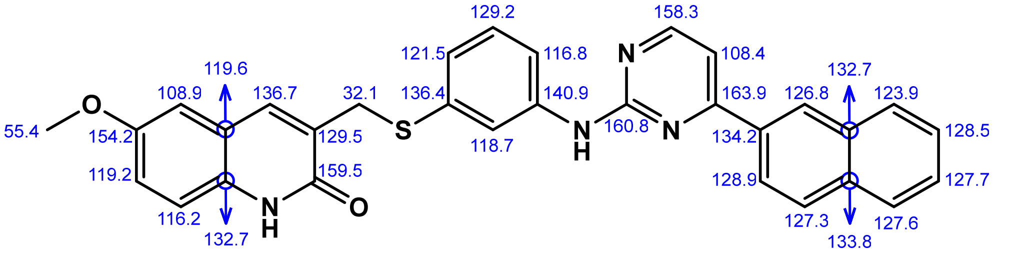
55.4
O
154.2
119.2
116.2
108.9
119.6
132.7
136.7
129.5
159.5
N
H
O
32.1
S
136.4
121.5
129.2
116.8
140.9
118.7
N
H
160.8
N
158.3
108.4
163.9
N
134.2
128.9
126.8
127.3
132.7
133.8
123.9
128.5
127.7
127.6

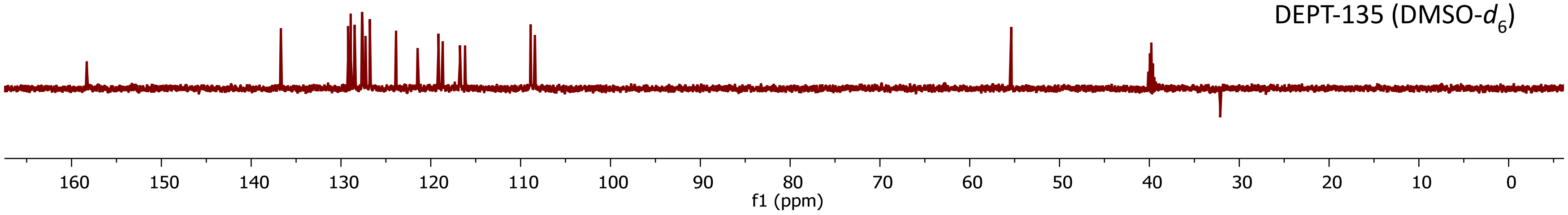
DEPT-135 (DMSO-d_6)
160
150
140
130
120
110
100
90
80
70
60
50
40
30
20
10
0
f1 (ppm)

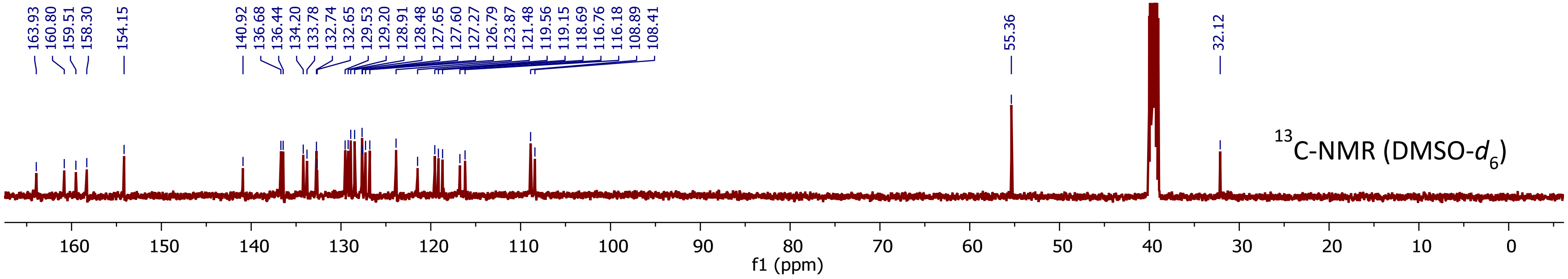
163.93
160.80
159.51
158.30
154.15
140.92
136.68
136.44
134.20
133.78
132.74
132.65
129.53
129.20
128.91
128.48
127.65
127.60
127.27
126.79
123.87
121.48
119.56
119.15
118.69
116.76
116.18
108.89
108.41
55.36
32.12
^{13}C-NMR (DMSO-d_6)
160
150
140
130
120
110
100
90
80
70
60
50
40
30
20
10
0
f1 (ppm)

EI MS (70eV)

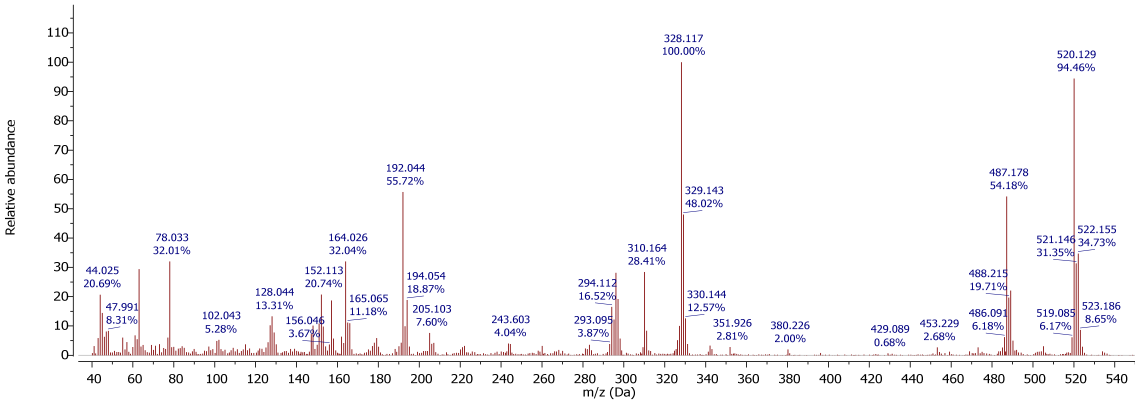

ESI-QTOF (positive ionization)

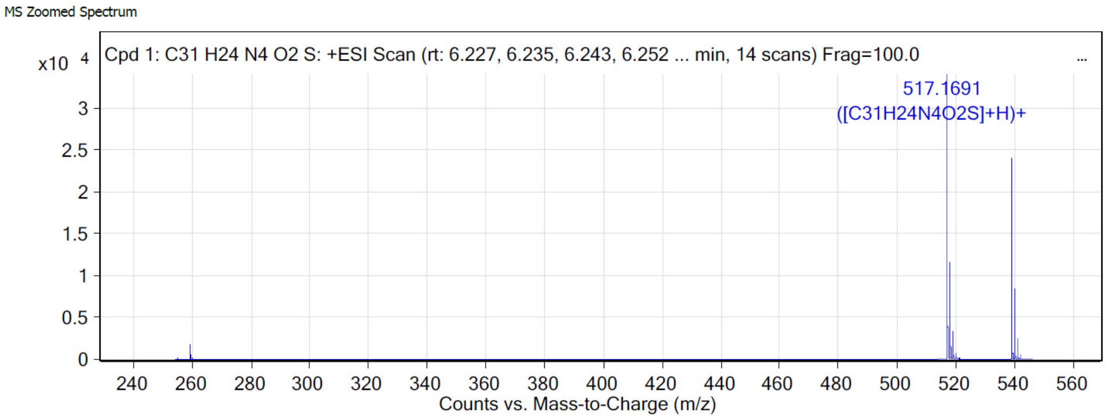

MS Spectrum Peak List

m/z	Calc *m/z*	Diff(ppm)	z	Abund	Formula	Ion
259.0882	259.0883	0.29	2	1807.07	C31H24N4O2S	(M+2H)+2
259.5901	259.5898	-1.32	2	601.35	C31H24N4O2S	(M+2H)+2
260.0892	260.0892	-0.06	2	184.95	C31H24N4O2S	(M+2H)+2
517.1691	517.1693	0.43	1	35187.08	C31H24N4O2S	(M+H)+
518.1726	518.1723	-0.53	1	12105.11	C31H24N4O2S	(M+H)+
519.1719	519.1712	-1.29	1	3356.03	C31H24N4O2S	(M+H)+
539.1511	539.1512	0.2	1	24291.18	C31H24N4O2S	(M+Na)+
540.1542	540.1543	0.13	1	8693.75	C31H24N4O2S	(M+Na)+
541.1534	541.1531	-0.5	1	2476.71	C31H24N4O2S	(M+Na)+
